# The Reactions of N,N′-Diphenyldithiomalondiamide with Arylmethylidene Meldrum’s Acids

**DOI:** 10.3390/ijms232415997

**Published:** 2022-12-15

**Authors:** Victor V. Dotsenko, Alexander V. Aksenov, Anna E. Sinotsko, Ekaterina A. Varzieva, Alena A. Russkikh, Arina G. Levchenko, Nicolai A. Aksenov, Inna V. Aksenova

**Affiliations:** 1 Department of Organic Chemistry and Technologies, Kuban State University, 149 Stavropolskaya St., 350040 Krasnodar, Russia; 2 Department of Organic Chemistry, North Caucasus Federal University, 1a Pushkin St., 355017 Stavropol, Russia

**Keywords:** active methylene compounds, Michael adducts, dithiomalonamides, heterocyclization, Meldrum’s acid

## Abstract

The Michael addition reaction between dithiomalondianilide (N,N′-diphenyldithiomalondiamide) and arylmethylidene Meldrum’s acids, accompanied by subsequent heterocyclization, was investigated along with factors affecting the mixture composition of the obtained products. The plausible mechanism includes the formation of stable Michael adducts which, under the studied conditions, undergo further transformations to yield corresponding N-methylmorpholinium 4-aryl-6-oxo-3-(N-phenylthio-carbamoyl)-1,4,5,6-tetrahydropyridin-2-thiolates and their oxidation derivatives, 4,5-dihydro-3H-[1,2]dithiolo[3,4-b]pyridin-6(7H)-ones. The structure of one such product, N-methylmorpholinium 2,2-dimethyl-5-(1-(2-nitrophenyl)-3-(phenylamino)-2-(N-phenylthiocarbamoyl)-3-thioxopropyl)-4-oxo-4H-1,3-dioxin-6-olate, was confirmed via X-ray crystallography.

## 1. Introduction

Active methylene compounds bearing a thioamide group are routinely used for the synthesis of various functionalized N,S-heterocycles such as 2-mercapto(2-thioxo)-pyridines [1,2,3,4,5,6], 4H-thiopyrans [7,8] and thiochromones [9], thiazolines [10], isothiazoles [11,12], 1,2,4-dithiazoles [13], 1,2,3-thiadiazoles and 1,2,3-triazoles [14,15], thieno[2,3-b]pyridines [16,17,18,19,20,21], and 1,3,5-thiadiazines [22,23], many of which are shown to be promising organic semi-conductors, fluorescent dyes, drugs, agrochemicals, etc. 

It is worth noting that while cyanothioacetamide NCCH_2_C(S)NH_2_ [24,25,26,27] and β-ketothioamides [27,28] are among the most common reagents in such types of heterocyclization reactions, the use of active methylene thio- and dithiomalonamides is far less studied [28], despite the fact that the latter often show quite different behavior, resulting in unique products. 

**Scheme 1:**
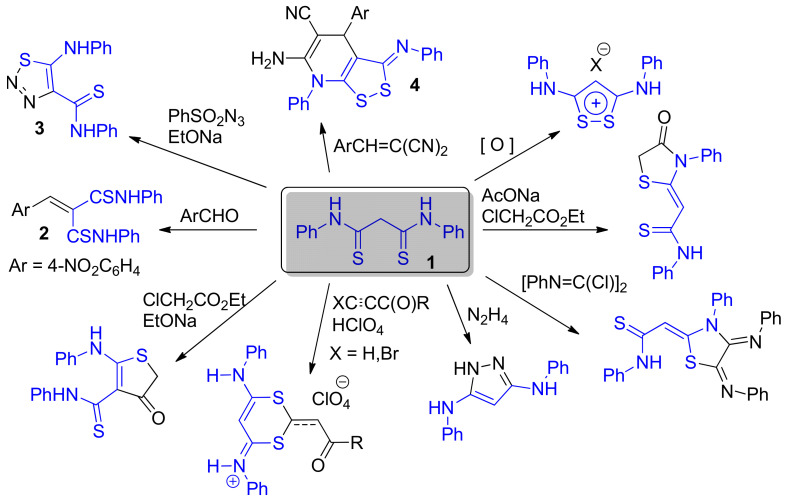
The diversity of reported dithiomalondianilide reactions.

Thus, for example, an application scope of the readily available N,N′-diphenyldithiomalondiamide (dithiomalondianilide) **1** [29,30] (Scheme 1) includes the synthesis of S,S- or S,N-bidentate ligands capable of heavy metal binding [31,32,33,34,35,36,37,38,39], or its use as a lubricant additive [40,41] or steel corrosion inhibitor [42]. In heterocyclic synthesis, dithioamide **1** was used to prepare 1,2-dithiolium salts [43,44], 1,3-dithiinium perchlorates [45,46,47,48], functionalized thiazolidines [49,50], 3,5-*bis*(phenylamino)pyrazole [51,52], and 2,3-dihydrothiophenes [51] (Scheme 1).

Nevertheless, despite its rather high CH-acidity (p*K*_a_ = 10.3) [30], there are only few articles reporting use of malondithiodianilide **1** as an active methylene reagent. Thus, the Knoevenagel condensation of compound **1** with 4-nitro benzaldehyde leads to the corresponding unsaturated thioanilide **2**, albeit in a very low yield (14%) [51]. The diazo-transfer reaction with phenylsulfonyl azide gives 1,2,3-thiadiazole **3** [53], while the tandem Michael addition–oxidation process with arylmethylidene malononitriles in the presence of morpholine results in [1,2]dithiolo[3,4-b]pyridines **4** [54].

**Scheme 2:**
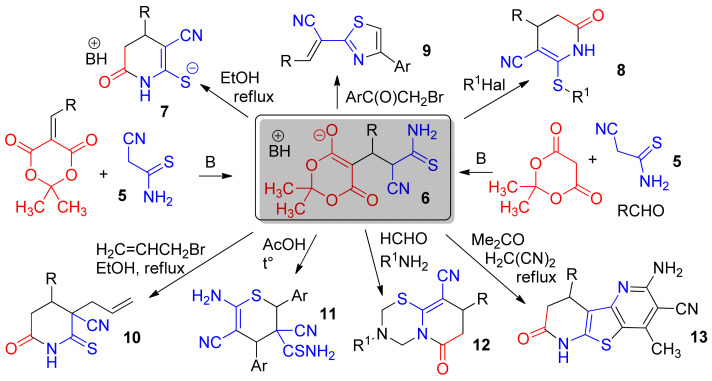
Synthesis and reactions of the Michael adducts **6**.

The preparation of labile (though isolable) Michael adducts **6** via the reaction of another active methylene compound, cyanothioacetamide **5**, with arylmethylidene Meldrum’s acids (or with Meldrum’s acid and aldehydes) is well documented [55,56,57,58,59,60,61,62,63,64,65,66,67,68] (Scheme 2). The adducts **6** (Scheme 2) are widely used as convenient precursors for further transformations into partially saturated nicotinonitriles **7**,**8** [56,57,58,59,60,62,63,67,68], (2-thiazolyl)acrylonitriles **9** [55], thioglutarimides **10** [69], functionalized 4H-thiopyrans **11** [55], pyrido[2,1-b][1,3,5]thiadiazines **12** [64,65,66,67,68,70], or polycyclic thienopyridine ensembles **13** [61]. Many of these compounds show promising biological activity [65,66,67,68,71,72,73,74,75]. However, no successful attempts to isolate similar Michael adducts derived from Meldrum’s acid and other active methylene thioamide-based reagents are known today [76,77].

Therefore, here, we would like to present the results of our study on the Michael addition reaction between N,N′-diphenyldithiomalondiamide **1** and various arylmethylidene Meldrum’s acids.

## 2. Results and Discussion

**Scheme 3:**
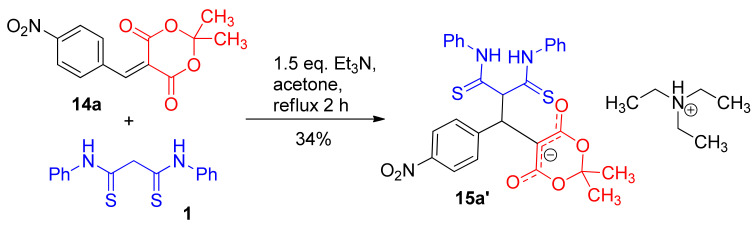
Synthesis of the Michael adduct **15a**′.

Initially, we choose 4-nitrobenzylidene Meldrum’s acid (2,2-dimethyl-5-(4-nitrobenzylidene)-1,3-dioxane-4,6-dione, **14a**) as a model substrate, only to find out that its reaction with N,N′-diphenyldithiomalondiamide **1** proceeds smoothly in refluxing acetone in the presence of excess Et_3_N, yielding 34% of the stable Michael adduct **15a**′ (Scheme 3) (Table 1, Entry 1):

At this point, some efforts to optimize the reaction conditions have been made. Thus, acetone was found to be the solvent of choice because it easily dissolves the starting reagents **1** and **14**, whereas the salt-like Michael adducts **15** are insoluble and precipitate during the reaction. The reaction in EtOH gives lower yields and the products are often contaminated with the starting N,N′-diphenyldithiomalondiamide **1**, which is poorly soluble in alcohols (Table 1, entry 2). In the same way, we checked some other solvents. MeCN, EtOAc, pyridine, and THF are not suitable solvents due to the bad solubility of dithiomalondianilide **1**. Halogenated hydrocarbons such as CH_2_Cl_2_ and ClCH_2_CH_2_Cl partially dissolve **1**, but cannot be used because of their alkylating effects.

**Scheme 4:**
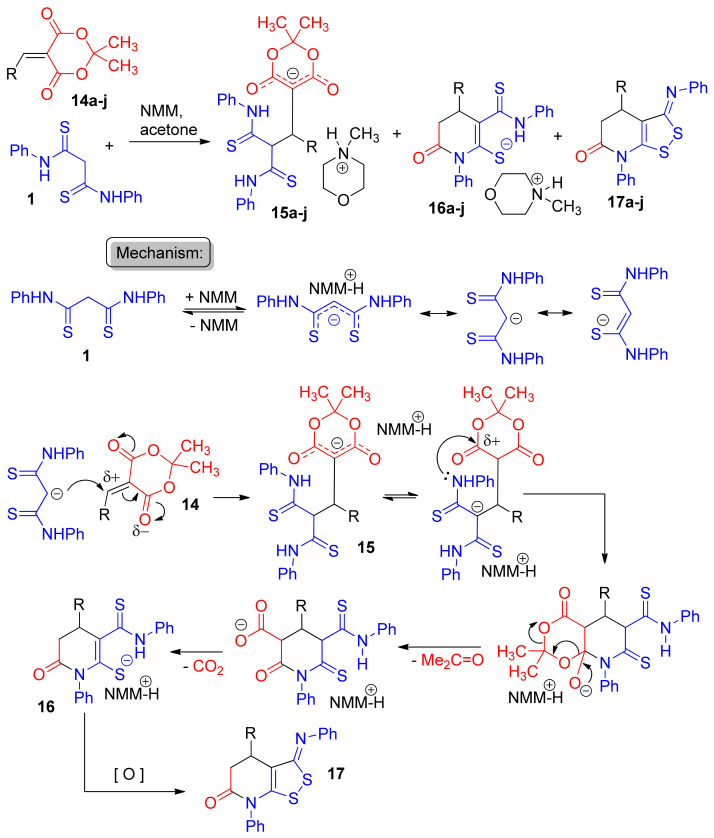
The reaction between dithiomalondianilide **1** and arylmethylidene Meldrum acids **14**: products and possible mechanistic pathway.

The presence of somewhat weaker base N-methylmorpholine instead of triethylamine improved the yield significantly, probably due to the lower solubility of the N-methylmorpholinium salts (Table 1, entry 3) and from further on, N-methylmorpholine was used as the preferable base. To evaluate the substrate scope, we introduced the Michael acceptors **14b**,**c** (R = 2-NO_2_C_6_H_4_ (**b**), 2-ClC_6_H_4_ (**c**)) as the model ones, in addition to the original **14a** (R = 4-NO_2_C_6_H_4_). As was found, the reaction time plays a significant role here as with its increase, the yields of Michael adducts **15** also increased. However, along with this, the formation of two side products—1,4,5,6-tetrahydropyridine-2-thiolates **16** originating from the cyclization of adducts **15** and the oxidation products of pyridine-2-thiolates **16**, 4,5-dihydro-3H-[1,2]dithiolo[3,4-b]pyridin-6(7H)-ones **17** (Scheme 4)—was also observed. Empirically, the best yields of the Michael adducts **15** can be achieved upon 40–80 min of gentle reflux in acetone, while further heating leads to noticeable subsequent heterocyclization of Michael adducts **15** into tetrahydropyridin-2-thiolates **16**. Still, the complete conversion of **15** → **16** does not happen under prolonged heating due to the ease of oxidation of thiolates **16** to[1,2]dithiolo[3,4-b]pyridines **17**. It should be pointed out that the mild oxidation of 2-mercaptopyridin-3-thiocarboxamides (or related-thiones or -thiolates) into[1,2]dithiolo[3,4-b]pyridine or isothiazolo[5,4-b]pyridine derivatives is well known and documented in the literature [54,78,79,80,81,82,83,84,85].

Notably, partially saturated [1,2]dithiolo[3,4-b]pyridines are a rather rare class of heterocyclic compounds and very little work has been conducted on their chemistry and preparation [54,86,87]. Preliminary experiments under an inert atmosphere (nitrogen) show that dithiolopyridines **17** do not form under these conditions. However, the mechanism of oxidation **16 → 17** is unclear and requires further study. 

The cyclization ability of Michael adducts **15** seems to be determined by their solubility, which depends largely on the structure of an aromatic substituent. Thus, the refluxing of dithiomalondianilide **1** and arylmethylidene compound **14a** in acetone for 2.5 h results in the formation of a mixture of Michael adduct **15a** and dithiolopyridine **17a** in a ~54:46 ratio (Table 1, entry 5); meanwhile, pure **15a**, although with a low yield (9%), can be isolated upon running the reaction at room temperature (Table 1, entry 4). On the other hand, the brief heating of an acetone solution of dithiodianilide **1**, N-methylmorpholine, and the Michael acceptor **14b** (Ar = 2-NO_2_C_6_H_4_) gives the adduct **15b** alone, while extended 2.5 h refluxing leads to a mixture of adduct **15b** and its cyclization product, thiolate **16b**, in a ~3:1 ratio (Table 1, entries 6,7) (Scheme 4). 

Meanwhile, the NMR spectra of Michael adduct **15c** prepared by heating **1** with 2,2-dimethyl-5-(2-chlorobenzylidene)-1,3-dioxane-4,6-dione **14c** in acetone for 3h reveals only traces of the corresponding thiolate **16c** (Table 1, entry 10). Similar results are observed in the case of some other arylmethylidene compounds **14d**–**j**, likely as a consequence of the better reactivity of Michael acceptors **14** bearing strong electron-withdrawing groups compared to those with strong electron-donor ones. Thus, according to the TLC, 40 min of reaction time is enough for the full conversion of the starting materials **14d** (Ar = 4-ClC_6_H_4_) and **14e** (Ar = 2,4-Cl_2_C_6_H_3_) to the corresponding adducts **15** and cyclization products **16** (Table 1, entries 11, 12). At the same time, according to the TLC and NMR data, the reaction of compound **14f** with dithiomalondianilide **1** occurs only with ~50% conversion after 80 min, giving ~33% of adduct **15f** and ~2% of **16f** (NMR yield) (Table 1, entry 13). Complete conversion of the starting reagents **14f** and **1** was achieved in only ~3 h and the oxidation product **17f** was detected via NMR among the compounds **15f** and **16f** (Table 1, entry 14).

**Table 1:** The products, conditions and yields in the reactions between **1** and Michael acceptors **14**.

Entries	Starting Reagents 14	Reaction Conditions	Products (Yields) ^1^		
			Michael Adducts 15	Pyridine-2-Thiolates 16	Dithiolo-Pyridines 17
entry 1	**14a** 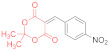	1.5 eq. Et_3_N, acetone, reflux 2 h	**15a**′ (34%)	ND ^2^	ND
entry 2	**14a** 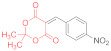	1.5 eq. NMM, EtOH, reflux 2 h	**15a** (42%)	traces	ND
entry 3	**14a** 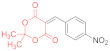	1.5 eq. NMM, acetone, reflux 1 h	**15a** (56%)	traces	ND
entry 4	**14a** 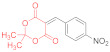	1.5 eq. NMM, acetone, 25 °C	**15a** (9%)	ND	ND
entry 5	**14a** 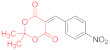	1.5 eq. NMM, acetone, reflux 2.5 h	**15a** (24%)	ND	**17a** (21%)
entry 6	**14b** 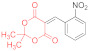	1.5 eq. NMM, acetone, reflux 1 h	**15b** (57%)	traces	ND
entry 7	**14b** 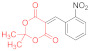	1.5 eq. NMM, acetone, reflux 2.5 h	**15b** (36%)	**16b** (13%)	ND
entry 8	**14c** 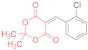	1.5 eq. NMM, acetone, reflux 20 min	**15c** (43%)	ND	ND
entry 9	**14c** 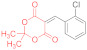	1.5 eq. NMM, acetone, reflux 40 min	**15c** (60%)	traces	ND
entry 10	**14c** 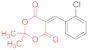	1.5 eq. NMM, acetone, reflux 3 h	**15c** (58%)	**16b** (<5%)	ND
entry 11	**14d** 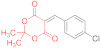	1.5 eq. NMM, acetone, reflux 40 min	**15d** (19%)	**16d** (19%)	ND
entry 12	**14e** 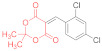	1.5 eq. NMM, acetone, reflux 35 min	**15e** (44%)	**16e** (22%)	ND
entry 13	**14f** 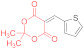	1.5 eq. NMM, acetone, reflux 80 min	**15f** (33%)	**16f** (2%)	ND
entry 14	**14f** 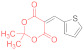	1.5 eq. NMM, acetone, reflux 3 h	**15f** (35%)	**16f** (20%)	**17f** (8%)
entry 15	**14g** 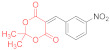	1.5 eq. NMM, acetone, reflux 80 min	**15g** (18%)	**16g** (14%)	**17g** (12%)
entry 16	**14h** 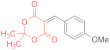	1.5 eq. NMM, acetone, reflux 1 h	**15h** (27%)	**16h** (7%)	traces
entry 17	**14h** 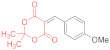	1.5 eq. NMM, acetone, reflux 4 h	**15h** (19%)	**16h** (4%)	**17h** (13%)
entry 18	**14i** 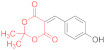	1.5 eq. NMM, acetone, reflux 1.5 h	**15i** (20%)	**16h** (~2%)	ND
entry 19	**14j** 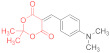	1.5 eq. NMM, acetone, reflux 80 min	ND	ND	**17j** (29%)

^1^ In entries 5,7,11–18, NMR yields are given; the products were not isolated in individual state. ^2^ ND—not detected.

Interestingly, unlike 2-nitro and 4-nitro, Michael acceptors **14a,b**, and 2,2-dimethyl-5-(3-nitrobenzylidene)-1,3-dioxane-4,6-dione **14g** under the same conditions (gentle heating for 80 min) give a complex mixture of the Michael addition/heterocyclization products **15g**, **16g**, and **17g** (in a molar ratio of ~42:32:26) along with detectable amounts of starting thioamide **1** (Table 1, entry 15).

In the case of 4-methoxybenzylidene Meldrum’s acid **14h**, the reaction does not proceed to completion after 1 h, resulting in a mixture of Michael adduct **15h** (27%), small amounts (~7%) of pyridine-2-thiolate **16h**, and plenty of unreacted starting material (Table 1, entry 16). According to the NMR, starting dithiomalondianilide **1** (about 7%) is still present in the reaction mixture even after 4 h along with adduct **15h** (~19%), thiolate **16h** (~4%), and dithiolopyridine **17h** (~13%) (Table 1, entry 17).

Similarly, only ~25% conversion is observed in the reaction with 4-hydroxybenzylidene Meldrum’s acid **14i** after 1.5 h. The NMR spectra reveal signals of the Michael adduct **15i** with a small admixture of thiolate **16i** (Table 1, entry 18). Moreover, even after 6 h of heating, the starting materials remain in the reaction mixture and full conversion does not occur due to, presumably, the reduced reactivity of compound **14i** caused by the strong donor effect of a substituent (OH or O^−^) at the para-position of the benzene ring.

In this regard, it is somewhat surprising that 5-[4-(dimethylamino)benzylidene]-2,2-dimethyl-1,3-dioxane-4,6-dione **14j** reacts smoothly with thioamide **1** to give a sole isolated product, 4-(4-(dimethylamino)phenyl)-7-phenyl-3-(phenylimino)-4,5-dihydro-3H-[1,2]dithiolo[3,4-b]pyridin-6(7H)-one **17j**, albeit in a modest yield (Table 1, entry 19). 

The structure of all the prepared compounds was confirmed via FT-IR and NMR spectroscopy (including DEPTQ ^13^C, 2D NMR ^1^H-^13^C HSQC and ^1^H-^13^C HMBC experiments) (See Electronic Supplementary Materials File). 1,4,5,6-Tetrahydropyridine-2-thiolates **16** and 4,5-dihydro-3H-[1,2]dithiolo[3,4-b]pyridin-6(7H)-ones **17** can be clearly identified through characteristic [56,57,58,59,60,62,63,67,68] ABX-patterns of C(O)-CH_2_-CHR protons in the ^1^H NMR spectra. In addition, the ^1^H NMR spectra of 1,4,5,6-tetrahydropyridine-2-thiolates **16** exhibit the signals of an N-methylmorpholinium cation and a broadened singlet of a C(S)NH proton, strongly shifted downfield to δ 15.81–15.92 ppm due to the intramolecular hydrogen bond S^−^…H-N(C=S). In the FTIR spectra of [1,2]dithiolo[3,4-b]pyridines **17**, absorption bands corresponding to C=N vibrations are detected, and the ^13^C DEPTQ spectra reveal no C=S signals; however, only C=N carbon signals appear at δ = 163–164 ppm.

**Figure 1:**
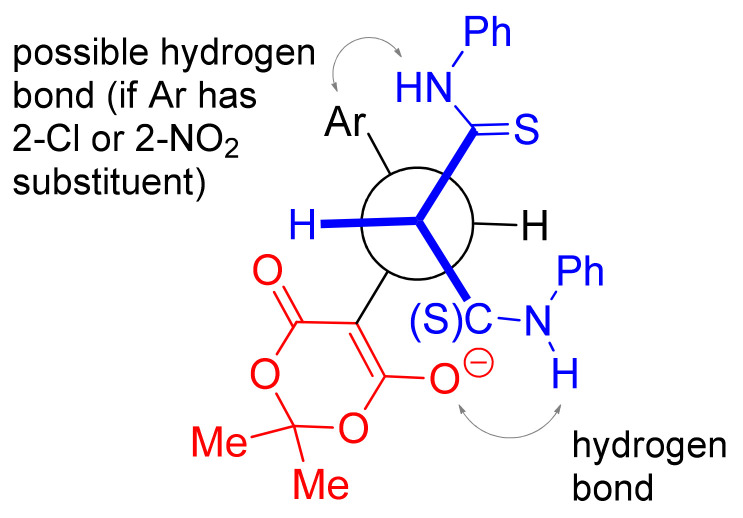
Hydrogen bonding in Michael adducts **15**.

In turn, the ^1^H and ^13^C NMR spectra of the Michael adducts **15** show non-equivalence of the signals of two different C(S)NHPh fragments. Thus, the difference in the ^1^H chemical shifts between C(S)NH peaks reaches Δ 1.05–1.07 ppm (e.g., adducts **15a,d**) and could be explained by hydrogen bonding between the negatively charged oxygen atom of the anionic part of the molecule and the proton of one of the C(S)NH groups (–O^−^…H-N(C=S) (Figure 1). The difference in chemical shifts between two C(S)NHPh protons decreases to Δ 0.3–0.5 ppm (e.g., adducts **15b**,**c**,**e**) when an aromatic ring bears a substituent capable of hydrogen bond formation at ortho-position (e.g., Cl, NO_2_).

**Figure 2:**
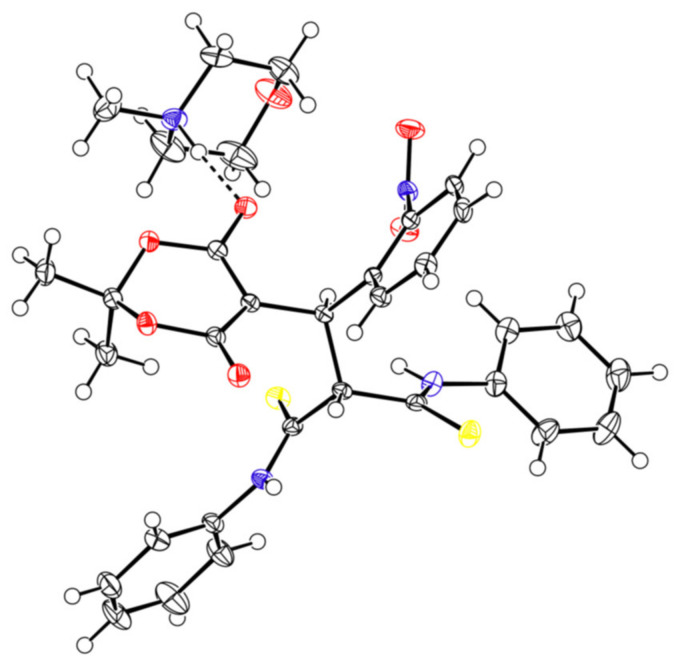
ORTEP drawing of X-ray structure for N-methylmorpholinium 2,2-dimethyl-5-(1-(2-nitrophenyl)-3-(phenylamino)-2-(N-phenylthiocarbamoyl)-3-thioxopropyl)-4-oxo-4H-1,3-dioxin-6-olate **15b** (CCDC deposition number **2218133**).

Another characteristic feature of the ^1^H NMR spectra of the Michael adducts **15** is that the signals of CH–CH protons appear as a pair of doublets with coupling constants, *^3^J* = 12.0–12.1 Hz. An approximate estimate of the CH–CH dihedral angle (ϕ) by the Haasnoot–DeLeeuw–Altona [88] and Altona–Donders [89] equations gives a range of 169°…177°, indicating the anti-periplanar orientation of CH–CH protons (Figure 1). These values are in a good agreement with the results of X-ray diffraction studies of a single crystal of the Michael adduct **15b**, according to which the CH–CH dihedral angle ϕ is 179.8° (Figure 2). 

**Scheme 5:**
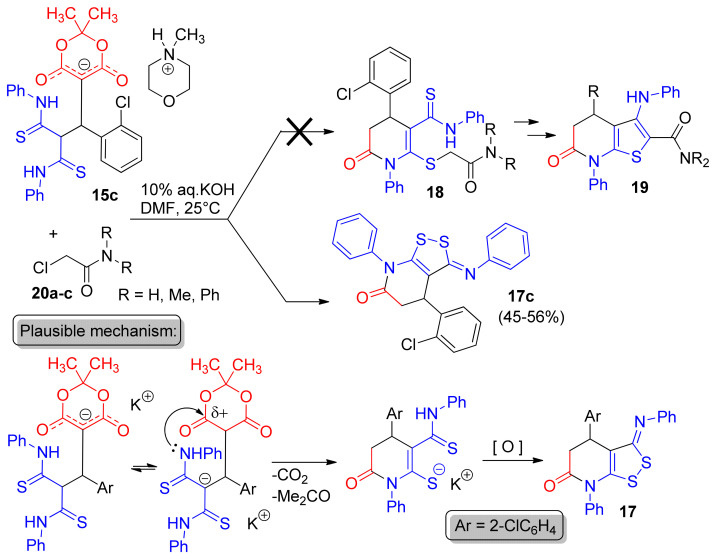
Synthesis and plausible mechanism of formation of 4-(2-chlorophenyl)-7-phenyl-3-(phenylimino)-4,5-dihydro-3H-[1,2]dithiolo[3,4-b]pyridin-6(7H)-one **17c**.

Next, we decided to study the reactivity of the studied Michael adducts **15** towards alkylating agents. The structurally similar thioamide-based Michael adducts **6** are known [55,60,62,69] to react with alkylating agents, with the formation of a variety heterocyclization products (Scheme 2). We speculated (Scheme 5) that the alkylation of adducts **15** would lead to pyridines **18**, which could be cyclized further into thieno[2,3-b]pyridines **19**, known for their pharmacological importance [16,17,18,19,20,21]. 

Therefore, we examined the reactions of the Michael adduct **15c** with certain α-chloroacetamides **20a**–**c** (Scheme 5) in DMF solution in the presence of 1 eq. KOH at 25 °C. To our surprise, no alkylation products were formed and the dithiolopyridine **17c** was the only isolated product in all cases. The most plausible mechanism involves intramolecular cyclization of the Michael adduct **15c** to form 3-(N-phenylthiocarbamoyl)-1,4,5,6-tetrahydropyridin-2-thiolate, which is then rapidly oxidized (most likely, by air). 

Considering all of the above, we envision two main directions for our future studies. First is the finding of direct and selective oxidation-free cyclization of the Michael adducts **15** to the corresponding 3-(N-phenylthiocarbamoyl)tetrahydropyridin-2-thiolates **16**, which are valuable reagents with great synthetic potential. Secondly, the mechanism of oxidation **16** → **17** and the development a new preparative transformation of adducts **15** into dithiolopyridines **17** will also be subjects of our further research efforts.

## 3. Materials and Methods

^1^H and ^13^C DEPTQ NMR spectra were recorded and 2D NMR experiments conducted in solutions of DMSO-*d*_6_ using a Bruker AVANCE-III HD instrument (at 400.40 or 100.61 MHz, respectively). Residual solvent signals were used as internal standards, in DMSO-*d*_6_ (2.49 ppm for ^1^H, and 39.50 ppm for ^13^C nuclei). Single-crystal X-ray diffraction analysis of compound **15b** was performed using an automatic four-circle diffractometer (Agilent Super Nova, Dual, Cu at zero, Atlas S2). High-resolution mass spectra (HRMS) were registered using a Bruker MaXis Impact spectrometer (electrospray ionization, using HCO_2_Na–HCO_2_H for calibration). The samples were dissolved in MeOH under moderate heating (37–38 °C) and ultrasonication. See the Supplementary Materials File for NMR, FTIR, HRMS spectral charts, and X-ray analysis data. 

FT-IR spectra were measured using a Bruker Vertex 70 instrument equipped with an ATR sampling module. Elemental analyses were carried out using a Carlo Erba 1106 Elemental Analyzer. The reaction progress and purity of isolated compounds were controlled via TLC on Sorbfil-A plates, eluent—acetone:hexane 1:1 or ethyl acetate, and the spots were visualized using UV-light or iodine vapors. Starting arylmethylidene Meldrum’s acids **14** were synthesized according to known procedures [90,91,92] and were identical to those described. N,N′-Diphenyldithiomalondiamide (dithiomalondianilide) **1** was prepared from acetylacetone and phenyl isothiocyanate as described earlier [29,30]. All other reagents and solvents were purchased from commercial vendors and used as received. A preliminary report on the reaction of **1** with arylmethylidene Meldrum’s acids was published as a proceeding paper [93].

*Triethylammonium 2,2-dimethyl-5-(1-(4-nitrophenyl)-3-(phenylamino)-2-(N-phenylthio-carbamoyl)-3-thioxopropyl)-4-oxo-4H-1,3-dioxin-6-olate* (**15a**′). A round-bottom flask was charged with anhydrous acetone (50 mL), 500 mg (1.80 mmol) of 4-nitrobenzylidene Meldrum’s acid (2,2-dimethyl-5-(4-nitrobenzylidene)-1,3-dioxane-4,6-dione, **14a**), 480 mg (1.80 mmol) of dithiomalondianilide **1**, and triethylamine (0.4 mL, 2.9 mmol). The solution was refluxed under vigorous stirring, and the crystals of adduct **15a**′ started to precipitate. The mixture was refluxed for 2 h and monitored via TLC until the starting reagents were consumed. The mixture was left overnight and the crystals were filtered off and washed with acetone and light petroleum. The yield was 400 mg (34%), bright yellow crystals. ^1^H NMR (400 MHz, DMSO-*d*_6_): 1.15 (t, ^3^*J* = 7.3 Hz, 9H, 3 CH_3_CH_2_), 1.27–1.31 (m, 6H, 2 Me), 3.07 (q, ^3^*J* = 7.3 Hz, 6H, 3 CH_3_CH_2_), 5.27 (d, ^3^*J* = 12.1 Hz, 1H, CH), 5.95 (d, ^3^*J* = 12.1 Hz, 1H, CH), 7.15 (AB_2_-t, ^3^*J* = 7.3 Hz, 1H, H-4 Ph), 7.21 (AB_2_-t, ^3^*J* = 7.3 Hz, 1H, H-4 Ph), 7.26–7.31 (m, 2H, H-Ph), 7.36–7.40 (m, 2H, H-Ph), 7.55 (d, ^3^*J* = 7.8 Hz, 2H, H-Ph), 7.80–7.84 (m, 4H, H-Ar), 7.98 (d, ^3^*J* = 8.7 Hz, 2H, H-Ar), 8.86 (br s, 1H, HN^+^), 10.89 (s, 1H, C(S)NH), 11.96 (s, 1H, C(S)NH). ^13^C DEPTQ NMR (101 MHz, DMSO-*d*_6_): 8.7* (3 CH_3_), 25.8* (2 CH_3_), 45.2* (CH–Ar), 45.7 (3 CH_2_N), 71.5* (CH–CSNHPh), 74.8 (C–C=O), 99.7 (O–CMe_2_–O), 122.1* (2 CH Ar), 123.1* (2 CH Ph), 123.3* (2 CH Ph), 125.9* (C^4^H Ph), 126.0* (C^4^H Ph), 128.3* (2 CH Ph), 128.4* (2 CH Ph), 129.6* (2 CH Ar), 139.4 (C^1^ Ph), 139.6 (C^1^ Ph), 144.9 (C Ar), 152.5 (C Ar), 197.0 (C=S), 198.8 (C=S). *Signals with a negative phase. The signal of C-O^−^ carbons was not observed, probably due to its low intensity. FTIR, ν_max_, cm^−1^: 3256, 3196, 3142, 2991, 2941, 2636 (N-H, C-H); 1516 (NO_2asym_); 1342 (NO_2sym_). HRMS (ESI) m/z: calculated for C_34_H_41_N_4_O_6_S_2_ [M + H]^+^: 665.2468; found: 665.2466 (Δ 0.3 ppm). Elemental Analysis (C_34_H_40_N_4_O_6_S_2,_ M 664.83): calculated (%): C, 61.42; H, 6.06; N, 8.43; found (%): C, 61.41; H, 6.15; N, 8.40.

*N-Methylmorpholinium 2,2-dimethyl-5-(1-(4-nitrophenyl)-3-(phenylamino)-2-(N-phenyl-thiocarbamoyl)-3-thioxopropyl)-4-oxo-4H-1,3-dioxin-6-olate* (**15a**) (Table 1, entry 3). To a clear solution of 2,2-dimethyl-5-(4-nitrobenzylidene)-1,3-dioxane-4,6-dione **14a** (369 mg, 1.33 mmol) and dithiomalondianilide **1** (381 mg, 1.33 mmol) in anhydrous acetone (15 mL), an excess (0.6 mL, 5.45 mmol) of N-methylmorpholine was added. The mixture was refluxed under vigorous stirring and bright yellow crystals of adduct **15a** started to precipitate within 5 min. The mixture was refluxed for 1 h and monitored via TLC until the starting reagents were consumed. The crystals were filtered off and washed with acetone and light petroleum. The yield was 491 mg (56%), bright yellow crystals. ^1^H NMR (400 MHz, DMSO-*d*_6_): 1.28 (br s, 3H, Me), 1.33 (br s, 3H, Me), 2.78 (s, 3H, NMe), 3.15–3.19 (m, 4H, CH_2_NCH_2_), 3.73–3.78 (m, 4H, CH_2_OCH_2_), 5.27 (d, ^3^*J* = 12.1 Hz, 1H, CH), 5.97 (d, ^3^*J* = 12.1 Hz, 1H, CH), 7.13–7.16 (m, 1H, H-4 Ph), 7.19–7.23 (m, 1H, H-4 Ph), 7.26–7.30 (m, 2H, H-3, H-5 Ph), 7.36–7.40 (m, 2H, H-3, H-5 Ph), 7.54 (d, ^3^*J* = 7.8 Hz, 2H, H-2, H-6 Ph), 7.82–7.85 (m, 4H, H-2, H-6 Ph and H-2, H-6 4-NO_2_C_6_H_4_ overlapped), 7.98 (d, ^3^*J* = 8.6 Hz, 2H, H-3, H-5 4-NO_2_C_6_H_4_), 9.68 (br s, 1H, HN^+^), 10.92 (s, 1H, C(S)NH), 11.97 (s, 1H, C(S)NH). ^13^C DEPTQ NMR (101 MHz, DMSO-*d*_6_): 25.6* (CH_3_), 25.9* (CH_3_), 42.6* (N–CH_3_), 45.2* (CH–Ar), 52.6 (CH_2_NCH_2_), 63.5 (CH_2_OCH_2_), 71.3* (CH–CSNHPh), 74.9 (C–C=O), 99.8 (O–CMe_2_–O), 122.1* (C-3 C-5 4-NO_2_C_6_H_4_), 123.0* (C-2, C-6 Ph), 123.3* (C-2, C-6 Ph), 125.8* (C-4 Ph), 126.0* (C-4 Ph), 128.28* (C-3, C-5 Ph), 128.34* (C-3, C-5 Ph), 129.5* (C-2 C-6 4-NO_2_C_6_H_4_), 139.4 (C-1 Ph), 139.6 (C-1 Ph), 144.9 (C-4 4-NO_2_C_6_H_4_), 152.5 (C–1 4-NO_2_C_6_H_4_), 164.8 (C–O^−^), 197.0 (C=S), 198.8 (C=S). *Signals with a negative phase. FTIR, ν_max_, cm^−1^: 3175, 2987, 2863 (N–H, C–H); 1516 (NO_2 asym_); 1346 (NO_2 sym_). Elemental Analysis (C_33_H_36_N_4_O_7_S_2,_ M 664.81): calculated (%): C, 59.62; H, 5.46; N, 8.43; found (%): C, 59.70; H, 5.55; N, 8.42.

When a mixture of dithiomalondianilide **1** (240 mg, 0.838 mmol), Michael acceptor **14a** (369 mg, 1.33 mmol), and N-methylmorpholine (0.13 mL, 1.18 mmol) was refluxed in acetone (15 mL) for 2.5 h, and then, left to stand overnight, the initially formed crystals of adduct **15a** disappeared; acetone was evaporated under reduced pressure, and the residue was treated with cold *n*-BuOH. The crystalline solid was filtered off and washed with BuOH and light petroleum to give 226 mg of yellow powder. According to ^1^H NMR, the product consisted of adduct **15a** and 4-(4-nitrophenyl)-3-(phenylimino)-4,5-dihydro-3H-[1,2]dithiolo[3,4-b]pyridin-6(7H)-one **17a** in a ~54:46 molar ratio (corresponds to ~136 mg of adduct **15a** (24%), and ~73 mg of dithiolopyridine **17a** (21%)) with trace amounts (~3.5% by weight) of starting dithiomalondianilide **1** (Table 1, entry 5). The observed signals of dithiolopyridine **17a**: ^1^H NMR (400 MHz, DMSO-*d*_6_): 2.89 (br d, ^2^*J* = 16.5 Hz, 1H, *cis* H-5), 3.64 (dd, ^2^*J* = 16.5 Hz, ^3^*J* = 8.2 Hz, 1H, *trans* H-5), 4.61–4.63 (m, 1H, H-4). ^13^C DEPTQ NMR (101 MHz, DMSO-*d*_6_): 36.3* (C-4), 37.7 (C-5), 112.5 (C-3a), 120.0* (CH Ar), 123.0* (CH Ph), 124.2* (CH Ph), 124.6* (CH Ph), 128.1* (CH Ph), 129.1* (CH Ar), 129.6* (CH Ph), 130.2* (CH Ph), 136.5 (C-1 Ph), 146.7 (C-1 Ph), 148.9 (C-1 4-NO_2_C_6_H_4_), 150.7 (C Ar), 158.0 (C-7a), 163.5 (C-3), 168.2 (C-6). *Signals with a negative phase. HRMS for **15a** (ESI) m/z: calculated for C_33_H_37_N_4_O_7_S_2_ [M + H]^+^: 665.21037; found: 665.2104 (Δ 0.05 ppm). HRMS for **17a** (ESI) m/z: calculated for C_24_H_18_N_3_O_3_S_2_ [M + H]^+^: 460.0784; found: 460.0790 (Δ 1.3 ppm).

*N-Methylmorpholinium 2,2-dimethyl-5-(1-(2-nitrophenyl)-3-(phenylamino)-2-(N-phenyl-thiocarbamoyl)-3-thioxopropyl)-4-oxo-4H-1,3-dioxin-6-olate* (**15b**) (Table 1, entry 6). To a clear solution of 2,2-dimethyl-5-(2-nitrobenzylidene)-1,3-dioxane-4,6-dione **14b** (250 mg, 0.9 mmol) and dithiomalondianilide **1** (260 mg, 0.9 mmol) in anhydrous acetone (15 mL), an excess (0.15 mL, 1.36 mmol) of N-methylmorpholine was added. The mixture was refluxed under vigorous stirring for 1.5 h. Then, acetone was partly evaporated under reduced pressure to half of its volume and the reaction mixture was left to stand overnight at ambient temperature in a stoppered flask. The precipitated bright yellow crystals were filtered off and washed with acetone and light petroleum to give 341 mg (57%) of adduct **15b**. The product contained trace amounts of starting dithiomalondianilide **1** and thiolate **16b**. ^1^H NMR (400 MHz, DMSO-*d*_6_): 1.32 (br s, 6H, Me), 2.74 (s, 3H, NMe), 3.09–3.12 (m, 4H, CH_2_NCH_2_), 3.72–3.77 (m, 4H, CH_2_OCH_2_), 5.42 (d, ^3^*J* = 12.0 Hz, 1H, CH), 6.00 (d, ^3^*J* = 12.0 Hz, 1H, CH), 7.13–7.17 (m, 1H, H-4 Ph), 7.19–7.25 (m, 1H, H-4 Ph and H-4 2-NO_2_C_6_H_4_ overlapped), 7.27–7.31 (m, 2H, H-3, H-5 Ph), 7.36–7.44 (m, 3H, H-3, H-5 Ph and H-5 2-NO_2_C_6_H_4_ overlapped), 7.49 (dd, ^3^*J* = 8.0 Hz, ^4^*J* = 0.9 Hz, 1H, H-3 2-NO_2_C_6_H_4_), 7.54 (d, ^3^*J* = 7.6 Hz, 2H, H-2, H-6 Ph), 7.75 (d, ^3^*J* = 7.7 Hz, 2H, H-2, H-6 Ph), 8.43 (d, ^3^*J* = 7.7 Hz, 1H, H-6 2-NO_2_C_6_H_4_), 9.75 (br s, 1H, HN^+^), 11.44 (s, 1H, C(S)NH), 11.74 (s, 1H, C(S)NH). ^13^C DEPTQ NMR (101 MHz, DMSO-*d*_6_): 27.2* (2 CH_3_), 38.7* (CH–Ar), 42.8* (N–CH_3_), 52.8 (CH_2_NCH_2_), 63.6 (CH_2_OCH_2_), 72.7* (CH–CSNHPh), 74.3 (C–C=O), 99.7 (O–CMe_2_–O), 122.5* (C-3 2-NO_2_C_6_H_4_), 123.0* (C-2, C-6 Ph), 123.9* (C-2, C-6 Ph), 125.9* (2C, C-4 Ph and C-4 2-NO_2_C_6_H_4_ overlapped), 126.1* (C-4 Ph), 128.2* (C-3, C-5 Ph), 128.3* (C-3, C-5 Ph), 130.3* (C-5 2-NO_2_C_6_H_4_), 131.6* (C-6 2-NO_2_C_6_H_4_), 135.7 (C-1 2-NO_2_C_6_H_4_), 139.4 (C-1 Ph), 139.8 (C-1 Ph), 149.9 (C-2 2-NO_2_C_6_H_4_), 165.5 (C–O^−^), 197.5 (C=S), 197.8 (C=S). *Signals with a negative phase. HRMS (ESI) m/z: calculated for C_33_H_37_N_4_O_7_S_2_ [M + H]^+^: 665.21037; found: 665.2106 (Δ 0.3 ppm). Elemental Analysis (C_33_H_36_N_4_O_7_S_2,_ M 664.81): calculated (%): C, 59.62; H, 5.46; N, 8.43; found (%): C, 59.76; H, 5.47; N, 8.53.

When a mixture of dithiomalondianilide **1** (260 mg, 0.9 mmol), Michael acceptor **14b** (250 mg, 0.9 mmol), and N-methylmorpholine (0.15 mL, 1.36 mmol) was refluxed in acetone (15 mL) for 2.5 h, and then, left to stand overnight, a small number of yellow crystals appeared. The mixture was concentrated under reduced pressure, and the crystalline residue was filtered off and washed with EtOH and light petroleum to give 283 mg of yellow solid. According to the NMR, the product was a mixture of Michael adduct **15b** and pyridine-2-thiolate **16b** in a ~3:1 molar ratio (corresponds to ~217 mg of adduct **15b** (36%) and ~66 mg of thiolate **16b** (13%)) (Table 1, entry 7). The identified signals of *N-methylmorpholinium 4-(2-nitrophenyl)-6-oxo-1-phenyl-3-(N-phenylthiocarbamoyl)-1,4,5,6-tetrahydropyridine-2-thiolate* **16b**: ^1^H NMR (400 MHz, DMSO-*d*_6_): 2.65 (dd, ^2^*J* = 15.7 Hz, ^3^*J* = 1.5 Hz, 1H, *cis* H-5), 2.73 (s, 3H, NMe), 3.09–3.13 (m, 4H, CH_2_NCH_2_), 3.19 (dd, ^2^*J* = 15.7 Hz, ^3^*J* = 7.7 Hz, 1H, *trans* H-5), 3.72–3.77 (m, 4H, CH_2_OCH_2_), 5.99–6.00 (m, 1H, H-4), 7.01–7.05 (m, 1H, H-4 Ph), 15.89 (br s, 1H, C(S)NHPh). The signals of most of the aromatic protons were difficult to identify due to complete or partial overlap with the signals of adduct **15b**. ^13^C DEPTQ NMR (101 MHz, DMSO-*d*_6_): 37.6* (C-4), 38.9 (C-5), 42.8* (N–CH_3_), 52.7 (CH_2_NCH_2_), 63.6 (CH_2_OCH_2_), 115.7 (C-3), 123.4* (CH Ar), 123.8* (CH Ar), 124.4* (CH Ar), 126.2* (CH Ar), 126.4* (CH Ar), 127.2* (CH Ar), 127.8* (CH Ar), 128.0* (CH Ar), 128.5* (CH Ar), 129.2* (CH Ar), 133.1* (CH Ar), 139.1 (C-1 2-NO_2_C_6_H_4_), 141.26 (C-1 Ph), 141.31 (C-1 Ph), 149.4 (C-2 2-NO_2_C_6_H_4_), 167.4 (C-2), 168.0 (C=O), 188.8 (C=S). *Signals with a negative phase.

*N-Methylmorpholinium 5-(1-(2-chlorophenyl)-3-(phenylamino)-2-(N-phenylthiocarbamo-yl)-3-thioxopropyl)-2,2-dimethyl-4-oxo-4H-1,3-dioxin-6-olate* (**15c**) (Table 1, entry 8). To a clear solution of 5-(2-chlorobenzylidene)-2,2-dimethyl-1,3-dioxane-4,6-dione **14c** (250 mg, 0.93 mmol) and dithiomalondianilide **1** (270 mg, 0.93 mmol) in anhydrous acetone (15 mL), an excess (0.15 mL, 1.36 mmol) of N-methylmorpholine was added. The mixture was refluxed under vigorous stirring, and a bright yellow crystalline adduct **15c** started to precipitate almost immediately. The mixture was heated under reflux for 20 min. The bright yellow crystals were filtered off and washed with acetone and light petroleum. The yield of pure **15c** was 268 mg (43%). ^1^H NMR (400 MHz, DMSO-*d*_6_): 1.31 (br s, 3H, Me), 1.34 (br s, 3H, Me), 2.75 (s, 3H, NMe), 3.12–3.16 (m, 4H, CH_2_NCH_2_), 3.73–3.77 (m, 4H, CH_2_OCH_2_), 5.44 (d, ^3^*J* = 12.1 Hz, 1H, CH-Ar), 5.86 (d, ^3^*J* = 12.1 Hz, 1H, CH-CSNHPh), 6.97–7.01 (m, 1H, H-4 2-ClC_6_H_4_), 7.05–7.08 (m, 1H, H-3 2-ClC_6_H_4_), 7.12–7.16 (m, 1H, H-4 Ph), 7.19–7.22 (m, 2H, H-4 Ph and H-5 2-ClC_6_H_4_ overlapped), 7.26–7.30 (m, 2H, H-3, H-5 Ph), 7.35–7.39 (m, 2H, H-3, H-5 Ph), 7.57 (d, ^3^*J* = 7.6 Hz, 2H, H-2, H-6 Ph), 7.79 (d, ^3^*J* = 7.7 Hz, 2H, H-2, H-6 Ph), 8.18 (d, ^3^*J* = 7.0 Hz, 1H, H-6 2-ClC_6_H_4_), 9.68 (br s, 1H, HN^+^), 11.52 (s, 1H, C(S)NH), 11.95 (s, 1H, C(S)NH). ^13^C DEPTQ NMR (101 MHz, DMSO-*d*_6_): 23.9* (CH_3_), 27.4* (CH_3_), 40.2* (CH–Ar), 42.7* (N–CH_3_), 52.7 (CH_2_NCH_2_), 63.5 (CH_2_OCH_2_), 73.1* (CH–CSNHPh), 73.4 (C–C=O), 99.6 (O–CMe_2_–O), 123.4* (C-2, C-6 Ph), 124.0* (C-2, C-6 Ph), 124.7* (C-3 2-ClC_6_H_4_), 125.75* (C-4 Ph), 125.78* (C-4 Ph), 126.0* (C-4 2-ClC_6_H_4_), 128.11* (C-3, C-5 Ph), 128.13* (C-3, C-5 Ph and C-5 2-ClC_6_H_4_ overlapped), 131.1* (C-6 2-ClC_6_H_4_), 133.5 (C-Cl), 139.6 (C-1 2-ClC_6_H_4_), 139.9 (C-1 Ph), 140.1 (C-1 Ph), 164.1, 167.1 (br s, 2 C–O^−^), 197.4 (C=S), 199.1 (C=S). *Signals with a negative phase. FTIR, ν_max_, cm^−1^: 3134, 2982, 2866 (N–H, C–H).

Elemental Analysis (C_33_H_36_ClN_3_O_5_S_2,_ M 654.24): calculated (%): C, 60.58; H, 5.55; N, 6.42; found (%): C, 60.73; H, 5.60; N, 6.51.

When a solution of dithiomalondianilide **1** (1.97 g, 6.9 mmol), Michael acceptor **14c** (1.85 g, 6.9 mmol), and N-methylmorpholine (1.35 mL, 10.35 mmol) in acetone (35–40 mL) was refluxed for 40 min, the yield of Michael adduct **15c** markedly increased (2.73 g, 60%). According to the NMR, the product contained only trace amounts of starting thioanilide **1** and pyridine-2-thiolate **16c** (Table 1, entry 9). Upon a longer heating period (3 h), the yields of **15c** were comparable but the yield of thiolate by-product **16c** increased (Table 1, entry 10).

The reaction of *5-(4-chlorobenzylidene)-2,2-dimethyl-1,3-dioxane-4,6-dione* **14d** and *dithiomalondianilide* **1** (Table 1, entry 11). To a clear solution of 5-(4-chlorobenzylidene)-2,2-dimethyl-1,3-dioxane-4,6-dione **14d** (250 mg, 0.94 mmol) and dithiomalondianilide **1** (270 mg, 0.94 mmol) in anhydrous acetone (15 mL), an excess (0.15 mL, 1.4 mmol) of N-methylmorpholine was added. The mixture was refluxed under vigorous stirring, and a bright yellow crystalline solid started to precipitate within 3–5 min. The mixture was heated under reflux for 40 min (monitored via TLC until the starting reagents were consumed). The crystals were filtered off and washed with acetone and light petroleum to give 214 mg of a bright yellow solid. According to the NMR, the product was a mixture of Michael adduct **15d** and pyridine-2-thiolate **16d** in a ~1:1 molar ratio (corresponds to ~116 mg of adduct **15d** (19%) and ~98 mg of thiolate **169** (19%)) (Table 1, entry 11). The identified signals of *N-methylmorpholinium 5-(1-(4-chlorophenyl)-3-(phenylamino)-2-(N-phenylthiocarbamoyl)-3-thioxopropyl)-2,2-dimethyl-4-oxo-4H-1,3-dioxin-6-olate* (**15d**): ^1^H NMR (400 MHz, DMSO-*d*_6_): 1.31 (br s, 6H, Me), 2.72 (s, 3H, NMe), 3.07–3.11 (m, 4H, CH_2_NCH_2_), 3.72–3.76 (m, 4H, CH_2_OCH_2_), 5.08 (d, ^3^*J* = 12.1 Hz, 1H, CH-Ar), 5.85 (d, ^3^*J* = 12.1 Hz, 1H, CH-CSNHPh), 6.84–7.85 (m, 14H, Ar), 9.65 (br s, 1H, HN^+^), 10.88 (s, 1H, C(S)NH), 11.85 (s, 1H, C(S)NH). ^13^C DEPTQ NMR (101 MHz, DMSO-*d*_6_): 25.8* (2 CH_3_), 42.9* (N–CH_3_), 44.6* (CH–Ar), 52.8 (CH_2_NCH_2_), 63.7 (CH_2_OCH_2_), 72.6* (CH–CSNHPh), 75.5 (C–C=O), 99.6 (O–CMe_2_–O), 123.1* (C-2, C-6 Ph), 123.5* (C-2, C-6 Ph), 130.6* (C-2, C-6 4-ClC_6_H_4_), 143.0 (C-1 4-ClC_6_H_4_), 164.7 (C–O^−^), 197.4 (C=S), 199.3 (C=S). The signals of most of the aromatic protons and carbons were difficult to identify due to complete or partial overlap with the signals of **16d**. *Signals with a negative phase. The identified signals of *N-methylmorpholinium 4-(4-chlorophenyl)-6-oxo-1-phenyl-3-(N-phenylthiocarbamoyl)-1,4,5,6-tetrahydropyridine-2-thiolate* **16d**: ^1^H NMR (400 MHz, DMSO-*d*_6_): 2.72 (s, 3H, NMe), 2.80 (dd, ^2^*J* = 15.5 Hz, ^3^*J* = 2.3 Hz, 1H, *cis* H-5), 3.00 (dd, ^2^*J* = 15.7 Hz, ^3^*J* = 5.8 Hz, 1H, *trans* H-5), 3.09–3.13 (m, 4H, CH_2_NCH_2_), 3.72–3.77 (m, 4H, CH_2_OCH_2_), 5.75–5.78 (m, 1H, H-4), 6.84–7.85 (m, 14H, Ar), 15.91 (br s, 1H, C(S)NHPh). ^13^C DEPTQ NMR (101 MHz, DMSO-*d*_6_): 39.1 (C-5), 39.6* (C-4), 42.9* (N–CH_3_), 52.8 (CH_2_NCH_2_), 63.7 (CH_2_OCH_2_), 116.7 (C-3), 129.3* (C-2, C-6 4-ClC_6_H_4_), 143.0 (C-1 4-ClC_6_H_4_), 166.4 (C-2), 168.4 (C=O), 188.9 (C=S). The signals of most of the aromatic protons and carbons were difficult to identify due to complete or partial overlap with the signals of **15d**. *Signals with a negative phase.

HRMS for **15d** (ESI) m/z: calculated for C_33_H_37_ClN_3_O_5_S_2_ [M + H]^+^: 654.1863; found: 654.1859 (Δ 0.6 ppm); calculated for C_28_H_26_ClN_2_O_4_S_2_ [M + H–NMM]^+^: 553.1023; found: 553.1014 (Δ 1.3 ppm); HRMS for **16d** (ESI) m/z: calculated for C_25_H_20_ClN_3_O_2_S_2_ [M + H + CO_2_–NMM]^+^: 495.0604; found: 495.0599 (Δ 1.0 ppm).

The reaction of *5-(2,4-dichlorobenzylidene)-2,2-dimethyl-1,3-dioxane-4,6-dione* **14e** and *dithiomalondianilide* **1** (Table 1, entry 12). To a clear solution of 5-(2,4-dichlorobenzylidene)-2,2-dimethyl-1,3-dioxane-4,6-dione **14e** (250 mg, 0.84 mmol) and dithiomalondianilide **1** (240 mg, 0.84 mmol) in anhydrous acetone (15 mL), an excess (0.14 mL, 1.25 mmol) of N-methylmorpholine was added. The mixture was refluxed under vigorous stirring and a bright yellow crystalline solid started to precipitate almost immediately. The mixture was heated under reflux for 35 min (monitored via TLC until the starting reagents were consumed). The crystals were filtered off and washed with acetone and light petroleum to give 360 mg of a bright yellow solid. According to the NMR, the product was a mixture of Michael adduct **15e** and pyridine-2-thiolate **16e** in a ~2:1 molar ratio (corresponds to ~253 mg of adduct **15e** (44%) and ~107 mg of thiolate **16e** (22%)) (Table 1, entry 12). The identified signals of *N-methylmorpholinium 5-(1-(2,4-dichlorophenyl)-3-(phenylamino)-2-(N-phenylthiocarbamoyl)-3-thioxopropyl)-2,2-dimethyl-4-oxo-4H-1,3-dioxin-6-olate* (**15e**): ^1^H NMR (400 MHz, DMSO-*d*_6_): 1.31 (br s, 3H, Me), 1.34 (br s, 3H, Me), 2.74 (s, 3H, NMe), 3.08–3.15 (m, 4H, CH_2_NCH_2_), 3.72–3.79 (m, 4H, CH_2_OCH_2_), 5.43 (d, ^3^*J* = 12.0 Hz, 1H, CH-Ar), 5.82 (d, ^3^*J* = 12.0 Hz, 1H, CH-CSNHPh), 7.02–7.39 (m, 9H, Ar), 7.62 (d, ^3^*J* = 7.8 Hz, 2H, Ar), 7.78 (d, ^3^*J* = 7.8 Hz, 2H, Ar), 9.63 (br s, 1H, HN^+^), 11.47 (s, 1H, C(S)NH), 11.98 (s, 1H, C(S)NH). ^13^C DEPTQ NMR (101 MHz, DMSO-*d*_6_): 23.9* (CH_3_), 27.4* (CH_3_), 40.0* (CH–Ar), 42.8* (N–CH_3_), 52.8 (CH_2_NCH_2_), 63.7 (CH_2_OCH_2_), 72.9* (CH–CSNHPh), 73.0 (C–C=O), 99.8 (O–CMe_2_–O), 123.2* (C-2, C-6 Ph), 124.0* (C-2, C-6 Ph), 128.19* (C-3, C-5 Ph), 128.21* (C-3, C-5 Ph), 139.2 (C-1 Ar), 139.5 (C-1 Ar), 140.0 (C-1 Ar), 197.1 (C=S), 198.8 (C=S). The signals of most of the aromatic protons and carbons were difficult to identify due to complete or partial overlap with the signals of **16e**. *Signals with a negative phase. The identified signals of *N-methylmorpholinium 4-(2,4-dichlorophenyl)-6-oxo-1-phenyl-3-(N-phenylthiocarbamoyl)-1,4,5,6-tetrahydropyridine-2-thiolate* **16e**: ^1^H NMR (400 MHz, DMSO-*d*_6_): 2.67 (dd, ^2^*J* = 15.5 Hz, ^3^*J* = 1.7 Hz, 1H, *cis* H-5), 2.74 (s, 3H, NMe), 3.06 (dd, ^2^*J* = 15.5 Hz, ^3^*J* = 6.7 Hz, 1H, *trans* H-5), 3.08–3.15 (m, 4H, CH_2_NCH_2_), 3.72–3.79 (m, 4H, CH_2_OCH_2_), 5.87–5.88 (m, 1H, H-4), 7.02–7.39 (m, 7H, Ar), 7.45 (dd, ^3^*J* = 8.4 Hz, ^4^*J* = 1.8 Hz, 1H, H-5 2,4-Cl_2_C_6_H_3_), 7.53 (d, ^4^*J* = 1.8 Hz, 1H, H-3 2,4-Cl_2_C_6_H_3_), 7.69 (d, ^3^*J* = 7.8 Hz, 2H, Ar), 8.21 (d, ^3^*J* = 8.7 Hz, 2H, Ar), 9.63 (br s, 1H, HN^+^), 15.90 (br s, 1H, C(S)NHPh). ^13^C DEPTQ NMR (101 MHz, DMSO-*d*_6_): 37.5 (C-5), 38.8* (C-4), 42.8* (N–CH_3_), 52.8 (CH_2_NCH_2_), 63.7 (CH_2_OCH_2_), 115.1 (C-3), 141.2 (C-1 Ar), 141.4 (C-1 Ar), 167.2 (C-2), 167.9 (C=O), 188.7 (C=S). The signals of most of the aromatic protons and carbons were difficult to identify due to complete or partial overlap with the signals of **15e**. *Signals with a negative phase. For adduct **15e**: HRMS (ESI) m/z: calculated for C_33_H_36_Cl_2_N_3_O_5_S_2_ [M + H]^+^: 688.1473; found: 688.1470 (Δ 0.43 ppm).

The reaction of *2,2-dimethyl-5-(2-thienylmethylene)-1,3-dioxane-4,6-dione* **14f** and *dithiomalondianilide* **1** (Table 1, entries 13,14). To a clear solution of 2,2-dimethyl-5-(2-thienylmethylene)-1,3-dioxane-4,6-dione **14f** (250 mg, 1.05 mmol) and dithiomalondianilide **1** (300 mg, 1.05 mmol) in anhydrous acetone (15 mL), an excess (0.17 mL, 1.58 mmol) of N-methylmorpholine was added. The mixture was refluxed under vigorous stirring and the formation of a small amount of crystalline product was detected within ~15 min. After 80 min, heating was stopped and the acetone was evaporated under reduced pressure. The residue (369 mg of yellow powder) contained, along with substantial amounts of starting reagents (about 50% of initial amounts), adduct **15f** and thiolate **16f** in a ~14:1 molar ratio. Full conversion was achieved in 3 h, acetone was removed *in vacuo*, and the residue contained 247 mg of yellow powder. NMR analysis revealed a complex mixture consisting of adduct **15f** (~35%), thiolate **16f** (~20%), and dithiolopyridine **17f** (~8%). The Michael adduct (*N-methylmorpholinium 5-(3-(phenylamino)-2-(N-phenylthiocarbamoyl)-1-(2-thienyl)-3-thioxopropyl)-2,2-dimethyl-4-oxo-4H-1,3-dioxin-6-olate*) **15f** was identified by characteristic signals in the ^1^H and ^13^C NMR spectra: ^1^H NMR (400 MHz, DMSO-*d*_6_): 1.35 (br s, 6H, Me), 2.54 (s, 3H, NMe), 2.82–2.90 (m, 4H, CH_2_NCH_2_), 3.65–3.75 (m, 4H, CH_2_OCH_2_), 5.31 (d, ^3^*J* = 11.5 Hz, 1H, CH-Ar), 5.73 (d, ^3^*J* = 11.5 Hz, 1H, CH-CSNHPh), 10.78 (br s, 1H, C(S)NH), 11.78 (br s, 1H, C(S)NH). ^13^C DEPTQ NMR (101 MHz, DMSO-*d*_6_): 25.3* (2 CH_3_), 40.7* (CH–Ar), 43.8* (N–CH_3_), 53.5 (CH_2_NCH_2_), 64.4 (CH_2_OCH_2_), 74.6* (CH–CSNHPh), 99.6 (O–CMe_2_–O), 197.0 (C=S), 199.5 (C=S). Other signals of protons and carbons were difficult to identify due to complete or partial overlap. *Signals with a negative phase. Side products **16f** and **17f** were identified via two characteristic *ABX*-patterns of –CH_2_–CH– fragments at δ 2.65–3.08 ppm and 5.00–5.70 ppm. In addition, thiolate **16f** was recognized via a peak at δ 15.81 ppm (C(S)NH) in ^1^H NMR and via peaks at δ 115.5 (C-3) and δ 188.7 (C=S) ppm in the ^13^C NMR spectrum. HRMS for **15f** (ESI) m/z: calculated for C_31_H_36_N_3_O_5_S_3_ [M + H]^+^: 626.1817; found: 626.1813 (Δ 0.6 ppm); calculated for C_26_H_25_N_2_O_4_S_3_ [M + H–NMM]^+^: 525.0977; found: 525.0974 (Δ 0.6 ppm).

The reaction of *2,2-dimethyl-5-(3-nitrobenzylidene)-1,3-dioxane-4,6-dione* **14g** and *dithiomalondianilide* **1** (Table 1, entry 15). To a clear solution of 2,2-dimethyl-5-(3-nitro-benzylidene)-1,3-dioxane-4,6-dione **14b** (250 mg, 0.9 mmol) and dithiomalondianilide **1** (260 mg, 0.9 mmol) in anhydrous acetone (15 mL), an excess (0.15 mL, 1.36 mmol) of N-methylmorpholine was added. The mixture was refluxed under vigorous stirring for 80 min (TLC control). No crystals appeared when the reaction mass was allowed to cool to room temperature. Acetone was evaporated under reduced pressure to give a viscous orange mass. Treatment with the cold mixture EtOH:acetone (1:1) resulted in the formation of a dark yellow crystalline solid (223 mg). According to the NMR, the product was a mixture of Michael adduct **15g**, pyridine-2-thiolate **16g**, and [1,2]dithiolo[3,4-b]pyridine **17g** in a ~42:32:26 molar ratio (corresponds to ~110 mg of adduct **15g** (18%), ~65 mg of thiolate **16g** (14%), and ~48 mg of dithiolopyridine **17g** (12%)) along with trace amounts of starting thioanilide **1**. The identified signals of *N-methylmorpholinium 2,2-dimethyl-5-(1-(3-nitro-phenyl)-3-(phenylamino)-2-(N-phenylthiocarbamoyl)-3-thioxopropyl)-4-oxo-4H-1,3-dioxin-6-olate* (**15g**): ^1^H NMR (400 MHz, DMSO-*d*_6_): 1.31 (br s, 6H, Me), 2.69 (s, 3H, NMe), 2.98–3.06 (m, 4H, CH_2_NCH_2_), 3.70–3.77 (m, 4H, CH_2_OCH_2_), 5.22 (d, ^3^*J* = 12.1 Hz, 1H, CH-Ar), 5.93 (d, ^3^*J* = 12.1 Hz, 1H, CH-CSNHPh), 6.87–8.48 (m, 14H, Ar), 10.92 (s, 1H, C(S)NH), 11.95 (s, 1H, C(S)NH). ^13^C DEPTQ NMR (101 MHz, DMSO-*d*_6_): 25.9* (2 CH_3_), 43.4* (N–CH_3_), 45.3* (CH–Ar), 53.2 (CH_2_NCH_2_), 64.1 (CH_2_OCH_2_), 71.8* (CH–CSNHPh), 74.9 (C–C=O), 99.7 (O–CMe_2_–O), 197.0 (C=S), 198.8 (C=S). The signals of most of the aromatic protons and carbons were difficult to identify due to complete or partial overlap with the signals of side products. *Signals with a negative phase. The identified signals of *N-methylmorpholinium 4-(3-nitrophenyl)-6-oxo-1-phenyl-3-(N-phenylthiocarbamoyl)-1,4,5,6-tetrahydropyridine-2-thiolate* **16g**: ^1^H NMR (400 MHz, DMSO-*d*_6_): 2.69 (s, 3H, NMe), 2.85 (dd, ^2^*J* = 15.7 Hz, ^3^*J* = 2.3 Hz, 1H, *cis* H-5), 2.98–3.06 (m, 4H, CH_2_NCH_2_), 3.10 (dd, ^2^*J* = 15.7 Hz, ^3^*J* = 6.0 Hz, 1H, *trans* H-5), 3.70–3.77 (m, 4H, CH_2_OCH_2_), 5.87–5.88 (m, 1H, H-4), 15.92 (br s, 1H, C(S)NHPh). ^13^C DEPTQ NMR (101 MHz, DMSO-*d*_6_): 36.0* (C-4), 38.0 (C-5), 43.4* (N–CH_3_), 53.2 (CH_2_NCH_2_), 64.1 (CH_2_OCH_2_), 116.1 (C-3), 166.9 (C-2), 168.2 (C=O), 188.8 (C=S). *Signals with a negative phase. The identified signals of *4-(3-nitrophenyl)-7-phenyl-3-(phenylimino)-4,5-dihydro-3H-[1,2]-dithiolo[3,4-b]pyridin-6(7H)-one* **17g**: ^1^H NMR (400 MHz, DMSO-*d*_6_): 2.95 (br d, ^2^*J* = 16.6 Hz, 1H, *cis* H-5), 3.63 (dd, ^2^*J* = 16.6 Hz, ^3^*J* = 8.1 Hz, 1H, *trans* H-5), 4.64–4.66 (m, 1H, H-4). ^13^C DEPTQ NMR (101 MHz, DMSO-*d*_6_): 40.2 (C-5), 40.3* (C-4), 112.8 (C-3a), 158.0 (C-7a), 163.6 (C-3), 168.4 (C-6). *Signals with a negative phase.

The reaction of *5-(4-methoxybenzylidene)-2,2-dimethyl-1,3-dioxane-4,6-dione* **14h** and *dithiomalondianilide* **1** (Table 1, entries 16,17). To a clear solution of compound **14h** (250 mg, 0.95 mmol) and dithiomalondianilide **1** (270 mg, 0.95 mmol) in anhydrous acetone (15 mL), an excess (0.16 mL, 1.43 mmol) of N-methylmorpholine was added. The mixture was refluxed under vigorous stirring and the formation of a small amount of crystalline product was detected within ~20 min. When the reaction was quenched after 1 h, acetone was evaporated under reduced pressure and the residue was treated with cold EtOH to give a yellow solid. According to the NMR, the product consisted mostly of unreacted **1** and **14h**, but also contained the adduct **15h** (27%), thiolate **16h** (7%), and traces of dithiolopyridine **17h**. According to the TLC, the reaction was completed in 4 h. The acetone was evaporated and the residue was treated with cold EtOH to give 220 mg of a yellow solid. However, the NMR spectrum revealed the signals of the starting thioamide **1**. The main products were adduct **15h** and dithiolopyridine **17h**, along with small amounts of thiolate **16h** (molar ratio ~53:37:10, corresponds to ~120 mg (19%) of **15h**, 57 mg (13%) of **17h**, and ~22 mg (4%) of **16h**). The identified signals of *N-methylmorpholinium 5-(1-(4-methoxyphenyl)-3-(phenylamino)-2-(N-phenylthiocarbamoyl)-3-thioxopropyl)-2,2-dimethyl-4-oxo-4H-1,3-dioxin-6-olate* (**15h**): ^1^H NMR (400 MHz, DMSO-*d*_6_): 1.29 (br s, 6H, 2 Me), 2.62 (s, 3H, NMe), 2.92–2.98 (m, 4H, CH_2_NCH_2_), 3.64 (s, 3H, MeO), 3.65–3.75 (m, 4H, CH_2_OCH_2_), 4.99 (d, ^3^*J* = 12.1 Hz, 1H, CH-Ar), 5.82 (d, ^3^*J* = 12.1 Hz, 1H, CH-CSNHPh), 6.64 (d, ^3^*J* = 8.7 Hz, 2H, H-3, H-5 4-MeOC_6_H_4_), 7.07–7.86 (m, 12H, Ar), 10.88 (s, 1H, C(S)NH), 11.77 (s, 1H, C(S)NH). ^13^C DEPTQ NMR (101 MHz, DMSO-*d*_6_): 25.5* (CH_3_), 26.2* (CH_3_), 43.4* (N–CH_3_), 44.3* (CH–Ar), 53.2 (CH_2_NCH_2_), 54.7* (OCH_3_), 64.1 (CH_2_OCH_2_), 73.5* (CH–CSNHPh), 76.2 (C–C=O), 99.4 (O–CMe_2_–O), 112.1* (C-3, C-5 4-MeOC_6_H_4_), 197.9 (C=S), 199.8 (C=S). The signals of most of the aromatic protons and carbons were difficult to identify due to complete or partial overlap with the signals of side products. *Signals with a negative phase. The identified signals of *N-methylmorpholinium 4-(4-methoxyphe-nyl)-6-oxo-1-phenyl-3-(N-phenylthiocarbamoyl)-1,4,5,6-tetrahydropyridine-2-thiolate* **16g**: ^1^H NMR (400 MHz, DMSO-*d*_6_): 2.62 (s, 3H, NMe), 2.92–2.98 (m, 4H, CH_2_NCH_2_), 3.85 (s, 3H, MeO), 3.65–3.75 (m, 4H, CH_2_OCH_2_), 5.70–5.71 (m, 1H, H-4), 15.92 (s, 1H, CSNHPh). ^13^C DEPTQ NMR (101 MHz, DMSO-*d*_6_): 43.4* (N–CH_3_), 53.2 (CH_2_NCH_2_), 55.8* (OCH_3_), 64.1 (CH_2_OCH_2_), 113.2* (C-3, C-5 4-MeOC_6_H_4_), 117.4 (C-3), 165.9 (C-2), 168.7 (C=O), 189.0 (C=S). *Signals with a negative phase. The identified signals of *4-(4-methoxyphenyl)-7-phenyl-3-(phenylimino)-4,5-dihydro-3H-[1,2]dithiolo[3,4-b]pyridin-6(7H)-one* **17g**: ^1^H NMR (400 MHz, DMSO-*d*_6_): 2.81 (br d, ^2^*J* = 16.2 Hz, 1H, *cis* H-5), 3.51 (dd, ^2^*J* = 16.2 Hz, ^3^*J* = 7.8 Hz, 1H, *trans* H-5), 3.74 (s, 3H, MeO), 4.39–4.41 (m, 1H, H-4). ^13^C DEPTQ NMR (101 MHz, DMSO-*d*_6_): 35.7* (C-4), 38.5 (C-5), 55.1* (OCH_3_), 114.2 (C-3a), 114.3* (C-3, C-5 4-MeOC_6_H_4_), 133.0 (C^1^ 4-MeOC_6_H_4_), 157.0 (C-7a), 158.3 (C^4^ 4-MeOC_6_H_4_), 163.6 (C-3), 168.8 (C=O). *Signals with a negative phase. HRMS for **15h** (ESI) m/z: calculated for C_34_H_40_N_3_O_6_S_2_ [M + H]^+^: 650.2359; found: 650.2353 (Δ 0.9 ppm); calculated for C_29_H_29_N_2_O_5_S_2_ [M + H–NMM]^+^: 549.1518; found: 549.1511 (Δ 1.3 ppm); HRMS for dithiolopyridine **17h** (ESI) m/z: calculated for C_25_H_21_N_2_O_2_S_2_ [M + H]^+^: 445.1044; found: 445.1043 (Δ 0.2 ppm).

The reaction of *5-(4-hydroxybenzylidene)-2,2-dimethyl-1,3-dioxane-4,6-dione* **14i** and *dithiomalondianilide* **1** (Table 1, entry 18). To a clear solution of compound **14i** (250 mg, 1.007 mmol) and dithiomalondianilide **1** (290 mg, 1.007 mmol) in anhydrous acetone (15 mL), an excess (0.17 mL, 1.51 mmol) of N-methylmorpholine was added. The mixture was refluxed under vigorous stirring for 1.5 h (no crystalline precipitate appeared). Then, the reaction was quenched, the acetone was evaporated under reduced pressure, and the residue was treated with cold EtOH to give a yellow solid (167 mg). According to the NMR, the product consisted mostly of unreacted **1** and **14i**, but also contained the adduct **15i** (~20%) and traces of thiolate **16i** (7%). The identified signals of *N-methylmorpholinium 5-(1-(4-hydroxyphenyl)-3-(phenylamino)-2-(N-phenylthiocarbamoyl)-3-thioxopropyl)-2,2-di-methyl-4-oxo-4H-1,3-dioxin-6-olate* (**15i**): ^1^H NMR (400 MHz, DMSO-*d*_6_): 1.30 (br s, 6H, 2 Me), 2.54 (s, 3H, NMe), 2.80–2.88 (m, 4H, CH_2_NCH_2_), 3.65–3.71 (m, 4H, CH_2_OCH_2_), 4.93 (d, ^3^*J* = 12.1 Hz, 1H, CH-Ar), 5.78 (d, ^3^*J* = 12.1 Hz, 1H, CH-CSNHPh), 6.47 (d, ^3^*J* = 8.4 Hz, 2H, H-3, H-5 4-HOC_6_H_4_), 7.36–7.37 (m, 2H, H-2, H-6 4-HOC_6_H_4_), 8.80 (br s, 1H, OH), 10.87 (s, 1H, C(S)NH), 11.71 (s, 1H, C(S)NH). ^13^C DEPTQ NMR (101 MHz, DMSO-*d*_6_): 25.4* (CH_3_), 26.3* (CH_3_), 43.8* (N–CH_3_), 44.4* (CH–Ar), 53.5 (CH_2_NCH_2_), 64.4 (CH_2_OCH_2_), 73.8* (CH–CSNHPh), 76.3 (C–C=O), 99.4 (O–CMe_2_–O), 113.5* (C-3, C-5 4-HOC_6_H_4_), 123.2* (C-2, C-6 Ph), 123.7* (C-2, C-6 Ph), 129.8* (C-2, C-6 4-HOC_6_H_4_), 134.4 (C-1 4-HOC_6_H_4_), 154.4 (C–OH), 163.7 (C–O^−^), 198.0 (C=S), 200.0 (C=S). The signals of most of the aromatic protons and carbons were difficult to identify due to complete or partial overlap with the signals of side products. *Signals with a negative phase. The identified signals of *N-methylmorpholinium 4-(4-hydroxyphenyl)-6-oxo-1-phenyl-3-(N-phenylthiocarbamoyl)-1,4,5,6-tetrahydropyridine-2-thiolate* (**16i**): ^1^H NMR (400 MHz, DMSO-*d*_6_): 2.54 (s, 3H, NMe), 2.66–2.75 (m, 1H, H-5), 2.80–2.88 (m, 4H, CH_2_NCH_2_), 3.65–3.71 (m, 4H, CH_2_OCH_2_), 5.66–5.68 (m, 1H, H-4), 15.92 (s, 1H, CSNHPh). The identified signals of starting dithiomalondianilide **1**: ^1^H NMR (400 MHz, DMSO-*d*_6_): 4.28 (s, 2H, CH_2_), 7.84–7.87 (m, 4H, 2 H–2, H–6 Ph), 11.85 (br s, 2H, 2 NH). ^13^C DEPTQ NMR (101 MHz, DMSO-*d*_6_): 62.8 (CH_2_), 123.0* (2 C–2, 2 C–6 Ph), 126.2* (2 C–4 Ph), 128.6* (2 C–3, 2 C–5 Ph), 139.4 (2 C–1 Ph), 195.4 (C=S). The identified signals of **14i**: ^1^H NMR (400 MHz, DMSO-*d*_6_): 1.71 (s, 6H, 2 Me), 6.89 (d, ^3^*J* = 8.8 Hz, 2H, H-3, H-5 4-HOC_6_H_4_), 8.17 (d, ^3^*J* = 8.8 Hz, 2H, H-2, H-6 4-HOC_6_H_4_), 8.25 (s, 1H, –CH=), 8.80 (br s, 1H, OH). ^13^C DEPTQ NMR (101 MHz, DMSO-*d*_6_): 26.9* (2 CH_3_), 103.9 (O–CMe_2_–O), 109.8 (C=CHAr), 115.9* (C-3, C-5 4-HOC_6_H_4_), 137.9* (C-2, C-6 4-HOC_6_H_4_), 157.0 (C–OH), 163.7 (C=O). HRMS for **15i** (ESI) m/z: calculated for C_33_H_38_N_3_O_6_S_2_ [M + H]^+^: 636.2202; found: 636.2199 (Δ 0.5 ppm); calculated for C_28_H_27_N_2_O_5_S_2_ [M + H–NMM]^+^: 535.1361; found: 535.1356 (Δ 0.9 ppm); HRMS for unreacted dithiomalondianilide **1** (ESI) m/z: calculated for C_15_H_15_N_2_S_2_ [M + H]^+^: 287.0677; found: 287.0672 (Δ 1.7 ppm).

*4-(4-(Dimethylamino)phenyl)-7-phenyl-3-(phenylimino)-4,5-dihydro-3H-[1,2]dithiolo[3,4-b]pyridin-6(7H)-one***17j** (Table 1, entry 19). To a clear solution of 5-[4-(dimethylamino)-benzylidene]-2,2-dimethyl-1,3-dioxane-4,6-dione **14j** (250 mg, 0.91 mmol) and dithio-malondianilide **1** (260 mg, 0.91 mmol) in anhydrous acetone (15 mL), an excess (0.15 mL, 1.36 mmol) of N-methylmorpholine was added. The mixture was refluxed under vigorous stirring for 80 min (monitored via TLC until the starting reagents were consumed). No crystalline precipitate appeared; the acetone was evaporated under reduced pressure and the tar residue was treated with warm (40 °C) BuOH to give a yellow solid. It was filtered off, and washed with BuOH, EtOH, and light petroleum to give 120 mg (29%) of pure dithiolopyridine **17j**. ^1^H NMR (400 MHz, DMSO-*d*_6_): 2.78 (br d, ^2^*J* = 16.0 Hz, 1H, *cis* H-5), 2.87 (s, 6H, NMe_2_), 3.47 (dd, ^2^*J* = 16.0 Hz, ^3^*J* = 7.7 Hz, 1H, *trans* H-5), 4.33–4.35 (m, 1H, H-4), 6.74 (d, ^3^*J* = 8.7 Hz, 2H, H-3, H-5 4-Me_2_NC_6_H_4_), 6.91 (d, ^3^*J* = 7.5 Hz, 2H, H-2, H-6 N(7)Ph), 7.08–7.12 (m, 1H, H-4 N(7)Ph), 7.19 (d, ^3^*J* = 8.7 Hz, 2H, H-2, H-6 4-Me_2_NC_6_H_4_), 7.33–7.45 (m, 4H, Ph), 7.54–7.56 (m, 3H, Ph). ^13^C DEPTQ NMR (101 MHz, DMSO-*d*_6_): 35.6* (C-4), 38.6 (C-5), 40.2* (NMe_2_), 112.8* (C-3, C-5 4-Me_2_NC_6_H_4_), 114.7 (C-3a), 120.0* (C-2, C-6 PhN(7)), 124.5* (C-4 PhN(7)), 127.1* (C-2, C-6 4-Me_2_NC_6_H_4_), 128.4 (C-1 4-Me_2_NC_6_H_4_), 129.0* (C-2, C-6=N-Ph), 129.8* (4C, C-3, C-5 of both phenyls overlapped), 130.0* (C-4 Ph), 136.7 (C-1=N-Ph), 149.6 (C-4 4-Me_2_NC_6_H_4_), 150.9 (C-1 PhN(7)), 156.7 (C-7a), 163.7 (C-3), 168.9 (C-6). *Signals with a negative phase. Elemental Analysis (C_26_H_23_N_3_OS_2,_ M 457.61): calculated (%): C, 68.24; H, 5.07; N, 9.18; found (%): C, 68.20; H, 5.19; N, 9.10.

*4-(2-Chlorophenyl)-7-phenyl-3-(phenylimino)-4,5-dihydro-3H-[1,2]dithiolo[3,4-b]pyridin-6(7H)-one* (**17c**). To a solution of Michael adduct 15c (225 mg, 0.344 mmol) in DMF (2 mL), an aqueous 10% solution of KOH (0.19 mL, 0.35 mmol) and corresponding chloroacetamide **20a**–**c** (0.35 mmol) were added. The reaction mixture was stirred for 1.5 h, and then, diluted with water (15 mL). A yellowish solid was filtered off and recrystallized from EtOH–acetone (4:1) to give dithiolopyridine **17c** as a yellow powder in a 45–56% yield. ^1^H NMR (400 MHz, DMSO-*d*_6_): 2.74 (dd, ^2^*J* = 16.3 Hz, ^3^*J* = 1.4 Hz, 1H, *cis* H-5), 3.64 (dd, ^2^*J* = 16.3 Hz, ^3^*J* = 8.3 Hz, 1H, *trans* H-5), 4.76–4.78 (m, 1H, H-4), 6.84 (dd, ^3^*J* = 8.3 Hz, ^4^*J* = 1.0 Hz, 2H, H-2, H-6 N(7)Ph), 7.06–7.10 (m, 1H, H-4 N(7)Ph), 7.31–7.43 (m, 3H, H-Ar), 7.37 (dd, ^3^*J* = 7.6 Hz, ^4^*J* = 1.6 Hz, 1H, H-Ar), 7.43–7.47 (m, 1H, H-Ar), 7.50–7.52 (m, 2H, H-Ar), 7.54–7.58 (m, 4H, H-Ar). 

^13^C DEPTQ NMR (101 MHz, DMSO-*d*_6_): 34.2* (C-4), 36.9 (C-5), 112.0 (C-3a), 120.0* (C-2, C-6 PhN(7)), 124.5* (C-4 PhN(7)), 127.3* (CH-Ar), 128.2* (CH-Ar), 129.0* (C-2, C-6=N-Ph), 129.2* (CH-Ar), 129.7* (4C, C-3, and C-5 of both phenyls overlapped), 130.1* (C-4 Ph), 130.3* (CH-Ar), 132.6 (C-1 Ar), 136.5 (C-1=N-Ph), 137.4 (C–Cl), 150.6 (C-1 PhN(7)), 158.6 (C-7a), 163.2 (C-3), 167.9 (C-6). *Signals with a negative phase. FTIR, ν_max_, cm^−1^: 2982, 2868 (C-H); 1653 (C=N). Elemental Analysis (C_24_H_17_ClN_2_OS_2,_ M 448.99): calculated (%): C, 64.20; H, 3.82; N, 6.24; found (%): C, 64.16; H, 3.95; N, 6.20.

X-ray studies for single crystals of **15b**.

Single crystals of N-methylmorpholinium 2,2-dimethyl-5-(1-(2-nitrophenyl)-3-(phenylamino)-2-(N-phenylthiocarbamoyl)-3-thioxopropyl)-4-oxo-4H-1,3-dioxin-6-olate **15b** (C_33_H_36_N_4_O_7_S_2_, M 664.78) were isolated from the reaction mixture (acetone solution). The crystal was kept at 100.01(11) K during data collection. Using Olex2 [94], the structure was solved with the olex2.solve structure solution program using Charge Flipping and refined with the SHELXL [95] package using Gauss-Newton minimization. The crystals were monoclinic, at 100.01(11) K: *a* = 14.39109(7) Å; *b* = 22.46449(13) Å; *c* = 20.81473(12) Å; α = 90°; β = 98.9856(5)°; γ = 90°; *V* = 6646.58(7) Å^3^; *T* = 100.01(11); space group P2_1_/n (no. 14); Z = 8; *μ*(Cu K_α_) = 1.896 mm^−1^; *d*_calc_ = 1.329 mg/mm^3^; F(000) = 2800.0; and 51297 reflections measured, 13563 unique (R_int_ = 0.0349), which were used in all calculations. The final wR_2_ was 0.0882 (all data) and the final R_1_ was 0.0336 (*I* > 2σ(*I*)). A full set of crystallographic data has been deposited at the Cambridge Crystallographic Data Center (CCDC **2218133**).

## 4. Conclusions

Generally, the reaction of arylmethylidene Meldrum’s acids **14** with N,N′-diphenyldithiomalondiamide **1** leads to complex mixtures containing the expected Michael adducts **15** as the products of their further heterocyclization (pyridines **16**) and oxidative transformation (dithiolopyridines **17**). Pure Michael adducts **15** as sole products were isolated only in a few cases. Seemingly, the main factors determining the outcome of the reaction are the presence of electron-withdrawing substituents in the aromatic fragment of arylmethylidene Meldrum’s acids **14** (due to greater reactivity) and the low solubility of some Michael adducts **15** in a chosen solvent, as this prevents them from undergoing further transformations. The presence of donor substituents leads to longer reaction times and the incomplete conversion of starting reagents, and favors the formation of N-methylmorpholinium 4-aryl-6-oxo-3-(N-phenylthiocarbamoyl)-1,4,5,6-tetrahydro-pyridin-2-thiolates **16** and 4,5-dihydro-3H-[1,2]dithiolo[3,4-b]pyridin-6(7H)-ones **17** as side products. The attempts to run the alkylation of Michael adducts **15** with certain α-chloroacetamides proved to be unsuccessful, and only the oxidation products of [1,2]dithiolo[3,4-b]pyridin-6(7H)-ones **17** were isolated. A logical continuation of this study would involve, firstly, improvement of the reaction regiocontrol and, secondly, direct and selective synthesis of the adducts’ **15** transformation products—1,4,5,6-tetrahydropyridin-2-thiolates **16** and [1,2]dithiolo[3,4-b]pyridines **17**.

## Data Availability

The file Electronic Supplementary Materials contains ^1^H and ^13^C DEPTQ NMR; 2D NMR ^1^H-^13^C HSQC; and ^1^H-^13^C HMBC, FTIR, and HRMS spectral charts for all the newly synthesized compounds (Figures S1–S72, S74–S81, Tables S1–S4), as well as X-ray crystallography data (Figure S73 and Tables S5–S11).

## Supplementary Materials

The following supporting information can be downloaded at: https://www.mdpi.com/article/10.3390/ijms232415997/s1.

## References

[1] Becker J., Stidsen C.E. (1988). Recent developments in the synthesis and chemistry of 2(1H)-pyridinethiones and related compounds. Sulfur Rep..

[2] Litvinov V.P., Rodinovskaya L.A., Sharanin Y.A., Shestopalov A.M., Senning A. (1992). Advances in the chemistry of 3-cyanopyridin-2(1H)-ones, -thiones, and -selenones. J. Sulfur Chem..

[3] Litvinov V.P. (1993). Advances in the chemistry of hydrogenated 3-cyanopyridine-2(1H)-thiones and -selenones. Phosphorus Sulfur Silicon Relat. Elem..

[4] Litvinov V.P. (1998). Partially hydrogenated pyridinechalcogenones. Russ. Chem. Bull..

[5] Litvinov V.P., Krivokolysko S.G., Dyachenko V.D. (1999). Synthesis and properties of 3-cyanopyridine-2(1H)-chalcogenones. Chem. Heterocycl. Compd..

[6] Litvinov V.P. (2006). The chemistry of 3-cyanopyridine-2(1H)-chalcogenones. Russ. Chem. Rev..

[7] Kuthan J., Šcebek P., Böuhm S. (1994). Developments in the chemistry of thiopyrans, selenopyrans, and teluropyrans. Adv. Heterocycl. Chem..

[8] Elnagdi M.H., Moustafa M.S., Al-Mousawi S.M., Mekheimer R.A., Sadek K.U. (2015). Recent developments in utility of green multi-component reactions for the efficient synthesis of polysubstituted pyrans, thiopyrans, pyridines, and pyrazoles. Mol. Divers..

[9] Sosnovskikh V.Y. (2019). Synthesis and properties of 2,3-heteroannulated thiochromones-hetero analogs of thioxanthone. Chem. Heterocycl. Compd..

[10] Litvinchuk M.B., Bentya A.V., Slyvka N.Y., Vovk M.V. (2020). 2-Ylidene-1,3-thiazolidines and their nonhydrogenated analogs: Methods of synthesis and chemical properties. Chem. Heterocycl. Compd..

[11] Taubert K., Kraus S., Schulze B. (2002). Isothiazol-3(2H)-Ones, Part I: Synthesis, reactions and biological activity. J. Sulfur Chem..

[12] Kletskov A.V., Bumagin N.A., Zubkov F.I., Grudinin D.G., Potkin V.I. (2020). Isothiazoles in the design and synthesis of biologically active substances and ligands for metal complexes. Synthesis.

[13] Metwally M.A., Abdel-Latif E., Bondock S. (2007). Thiocarbamoyl derivatives as synthons in heterocyclic synthesis. J. Sulfur Chem..

[14] Shafran Y., Glukhareva T., Dehaen W., Bakulev V. (2018). Recent developments in the chemistry of 1,2,3-thiadiazoles. Adv. Heterocycl. Chem..

[15] Bakulev V., Shafran Y., Dehaen W. (2019). Progress in intermolecular and intramolecular reactions of thioamides with diazo compounds and azides. Tetrahedron Lett..

[16] Bakhite E.A.-G. (2003). Recent trends in the chemistry of thienopyridines. Phosphorus Sulfur Silicon Relat. Elem..

[17] Litvinov V.P., Dotsenko V.V., Krivokolysko S.G. (2005). Thienopyridines: Synthesis, properties, and biological activity. Russ. Hcem. Bull. Int. Ed..

[18] Litvinov V.P., Dotsenko V.V., Krivokolysko S.G. (2007). The chemistry of thienopyridines. Adv. Heterocycl. Chem..

[19] Sajadikhah S.S., Marandi G. (2019). Recent approaches to the synthesis of thieno[2,3-b]pyridines (microreview). Chem. Heterocycl. Compd..

[20] Dotsenko V.V., Buryi D.S., Lukina D.Y., Krivokolysko S.G. (2020). Recent advances in the chemistry of thieno[2,3-*b*]pyridines 1. Methods of synthesis of thieno[2,3-*b*]pyridines. Russ. Chem. Bull. Int. Ed..

[21] Larionova N.A., Shestopalov A.M., Rodinovskaya L.A., Zubarev A.A. (2022). Synthesis of biologically active heterocycles via a domino sequence involving an S_N_2/Thorpe–Ziegler Reaction Step. Synthesis.

[22] Dotsenko V.V., Frolov K.A., Krivokolysko S.G. (2015). Synthesis of partially hydrogenated 1,3,5-thiadiazines by Mannich reaction. Chem. Heterocycl. Compd..

[23] Dotsenko V.V., Frolov K.A., Chigorina E.A., Khrustaleva A.N., Bibik E.Y., Krivokolysko S.G. (2019). New possibilities of the Mannich reaction in the synthesis of N-, S,N-, and Se,N-heterocycles. Russ. Chem. Bull. Int. Ed..

[24] Abdel-Galil F.M., Sherif S.M., Elnagdi M.H. (1986). Utility of cyanoacetamide and cyanothioacetamide in heterocyclic synthesis. Heterocycles.

[25] Litvinov V.P. (1999). Cyanoacetamides and their thio- and selenocarbonyl analogues as promising reagents for fine organic synthesis. Russ. Chem. Rev..

[26] Dyachenko V.D., Dyachenko I.V., Nenajdenko V.G. (2018). Cyanothioacetamide: A polyfunctional reagent with broad synthetic utility. Russ. Chem. Rev..

[27] Britsun V.N., Esipenko A.N., Lozinskii M.O. (2008). Heterocyclization of thioamides containing an active methylene group (review). Chem. Heterocycl. Compd..

[28] Lozynskii M.O., Britsun V.M., Esipenko A.M., Borysevych A.M. (2008). Thio- and dithioamides of malonic acid: Synthesis, structure and heterocyclizations. Ukr. Khim. Zhurn..

[29] Barnikow G., Kath V., Richter D. (1965). Isothiocyanate. II. N,N′-Aryl-substituierte Dithiomalonsäurediamide. J. Prakt. Chem..

[30] Sinotsko A.E., Bespalov A.V., Pashchevskaya N.V., Dotsenko V.V., Aksenov N.A., Aksenova I.V. (2021). *N*,*N*′-Diphenyldithiomalonodiamide: Structural Features, Acidic Properties, and In Silico Estimation of Biological Activity. Russ. J. Gen. Chem..

[31] Barnikow G., Kunzek H. (1966). Nickel-Chelate von N,N′-Diaryl-dithiomalonsäure-diamiden. Z. Chem..

[32] Peyronel G., Pellacani G.C., Benetti G., Pollacci G. (1973). Nickel(II) complexes with dithiomalonamide and NN′-diphenyldithiomalonamide. J. Chem. Soc. Dalton Trans..

[33] Pellacani G.C. (1974). Palladium(II) complexes with dithiomalonamide and N,N′-diphenyldithiomalonamide. Can. J. Chem..

[34] Pellacani G.C., Peyronel G., Pollacci G., Coronati R. (1976). Zinc(II) complexes of dithiomalonamide, N,N′-dimethyl- and N,N′-diphenyl-dithiomalonamide: ZnLX_2_ (X = Cl, Br, I) and ZnL_2_(ClO_4_)_2_. J. Inorg. Nucl. Chem..

[35] Pellacani G.C., Peyronel G., Malavasi W., Menabue L. (1977). Antimony and bismuth trihalide complexes of dithiomalonamide, N,N′-dimethyl- and N,N′-diphenyl-dithiomalonamide. J. Inorg. Nucl. Chem..

[36] Pal T., Ganguly A., Maity D.S., Livingstone S.E. (1986). N,N′-diphenyldithiomalonamide as a gravimetric reagent for nickel and cobalt. Talanta.

[37] Pal T., Ganguly A., Pal A. (1988). Spectrophotometric determination of cobalt and nickel with N,N′-diphenyldithiomalonamide and elucidation of structures of the compounds. J. Ind. Chem. Soc..

[38] Battaglia L.P., Bonamartini Corradi A., Marzotto A., Menabue L., Pellacani G.C. (1988). Nickel(II) and palladium(II) complexes of dithiomalonamides: Ligands which favor the formation of π-conjugation systems in the coordination. J. Crystallogr. Spectrosc. Res..

[39] Battaglia L.P., Bonamartini Corradi A., Marzotto A., Menabue L., Pellacani G.C. (1988). Co-ordinative abilities of ligands which favour S,S chelation: Copper(I) halide complexes of N,N′-diphenyldithiomalonamide. The crystal and molecular structure of bis(N,N′-diphenyldithiomalonamide)copper(I) iodide–methanol (2/1). J. Chem. Soc. Dalton Trans..

[40] Singh T., Singh R., Verma V.K. (1990). Evaluation of 2,4-dithiomalonamides as extreme pressure additives in the four-ball test. Proc. Inst. Mech. Eng. Part D J. Automob. Eng..

[41] Singh T. (2008). Tribochemistry and EP activity assessment of Mo-S complexes in lithium-base greases. Adv. Tribol..

[42] Kumar A., Singh M.M. (1993). Substituted dithiomalonamides as inhibitor for the corrosion of AISI 304SS in phosphoric acid—Hydrochloric acid mixture. Anti-Corros. Methods Mater..

[43] Schmidt U. (1959). Synthesen mit den Thioamiden der Malonsäure, I. 3,5-Diamino-dithiyliumsalze. Ein neuer pseudoaromatischer Fünfring mit 1,2-Stellung der S-Atome. Chem. Ber..

[44] Barnikow G. (1967). Isothiocyanate, XIV. 3.5-Bis-arylamino-1.2-dithioliumsalze aus N.N′-Diaryl-dithiomalonsäure-diamiden. Chem. Ber..

[45] Nizovtseva T.V., Komarova T.N., Nakhmanovich A.S., Larina L.I., Lopyrev V.A., Kalistratova E.F. (2002). Reaction of Dithiomalonamide and Dianilide with α-Acetylene Ketones. Russ. J. Org. Chem..

[46] Nizovtseva T.V., Komarova T.N., Nakhmanovich A.S., Larina L.I., Lopyrev V.A. (2003). Synthesis of 1,3-dithiinium salts. Arkivoc.

[47] Elokhina V.N., Yaroshenko T.I., Nakhmanovich A.S., Larina L.I., Amosova S.V. (2006). Reaction of dithiomalonic acid dianilide with substituted acetylenic ketones. Russ. J. Gen. Chem..

[48] Volkova K.A., Nakhmanovich A.S., Elokhina V.N., Yaroshenko T.I., Larina L.I., Shulunova A.M., Amosova S.V. (2007). Reaction of dithiomalonic acid dianilide with methyl propiolate. Russ. J. Org. Chem..

[49] Obydennov K.L., Golovko N.A., Kosterina M.F., Pospelova T.A., Slepukhin P.A., Morzherin Y.Y. (2014). Synthesis of 4-oxothiazolidine-2,5-diylidenes containing thioamide group based on dithiomalonamides. Russ. Chem. Bull. Int. Ed..

[50] Beckert R., Gruner M. (1997). Regioselektive Cyclisierung von Thiocarbonsäureamiden mit Bisimidoylchloriden—Synthese von Thiazolidinen mit einer Ketenacetal-Substruktur. Z. Naturforsch. B Chem. Sci..

[51] Barnikow G. (1966). Isothiocyanate, X. Pyrazole und Thiophene aus N.N′-Diaryl-dithiomalonsäure-diamiden. Liebigs Ann. Chem..

[52] Degorce S., Jung F.H., Harris C.S., Koza P., Lecoq J., Stevenin A. (2011). Diversity-orientated synthesis of 3,5-bis(arylamino)pyrazoles. Tetrahedron Lett..

[53] Bakulev V.A., Lebedev A.T., Dankova E.F., Mokrushin V.S., Petrosyan V.S. (1989). Two directions of cyclization of α-diazo-β-dithioamides. New rearrangements of 1,2,3-triazole-4-carbothiamides. Tetrahedron.

[54] Dotsenko V.V., Krivokolysko S.G., Frolov K.A., Chigorina E.A., Polovinko V.V., Dmitrienko A.O., Bushmarinov I.S. (2015). Synthesis of [1,2]dithiolo[3,4-*b*]pyridines via the reaction of dithiomalondianilide with arylmethylidenemalononitriles. Chem. Heterocycl. Compd..

[55] Goncharenko M.P., Sharanin Y.A., Turov A.V. (1993). Meldrum’s acid in reactions with arylmethylenecyanothioacetamides. Russ. J. Org. Chem..

[56] Nesterov V.N., Krivokolysko S.G., Dyachenko V.D., Dotsenko V.V., Litvinov V.P. (1997). Synthesis, properties, and structures of ammonium 4-aryl-5-cyano-2-oxo-1,2,3,4-tetrahydropyridine-6-thiolates. Russ. Chem. Bull..

[57] Dyachenko V.D., Krivokolysko S.G., Litvinov V.P. (1997). A new method for the synthesis of N-methylmorpholinium 4-aryl-5-cyano-2-oxo-1,2,3,4-tetrahydropyridine-6-thiolates and their properties. Russ. Chem. Bull..

[58] Dyachenko V.D., Krivokolysko S.G., Litvinov V.P. (1997). Synthesis and some properties of 4-alkyl-5-cyano-6-mercapto-3,4-dihydropyridin-2(1H)-ones. Russ. Chem. Bull..

[59] Krivokolysko S.G., Dyachenko V.D., Litvinov V.P. (1999). Synthesis and alkylation of N-methylmorpholinium 5-cyano-4-(3- and 4-hydroxyphenyl)-2-oxo-1,2,3,4-tetrahydropyridine-6-thiolates. Russ. Chem. Bull..

[60] Krivokolysko S.G., Chernega A.N., Litvinov V.P. (2002). Synthesis, structure, and alkylation of N-methylmorpholinium 5-[2-cyanoethyl-1-(4-hydroxy-3-methoxyphenyl)-2-thiocarbamoyl]-2,2-dimethyl-6-oxo-1,3-dioxa-4-cyclohexen-4-olate. Chem. Heterocycl. Compd..

[61] Dotsenko V.V., Krivokolysko S.G., Chernega A.N., Litvinov V.P. (2003). Fused sulfurcontaining pyridine systems 1. Synthesis and structures of tetrahydropyridothienopyridinone and tetrahydropyridothiopyranopyridinone derivatives. Russ. Chem. Bull. Int. Ed..

[62] Dyachenko V.D. (2006). A simple and efficient route to substituted 2-alkylsulfanyl-6-oxo-1,4,5,6-tetrahydropyridine-3-carbonitriles. Russ. J. Org. Chem..

[63] Dotsenko V.V., Lebedeva I.A., Krivokolysko S.G., Povstyanoi M.V., Povstyanoi V.M., Kostyrko E.O. (2012). Reaction of ethyl 4-aryl-6-bromomethyl-2-oxo-1,2,3,4-tetrahydropyrimidine-5-carboxylates with N-methylmorpholinium 3-cyano-1,4-dihydro- and 3-cyano-1,4,5,6-tetrahydropyridine-2-thiolates. Chem. Heterocycl. Compd..

[64] Dotsenko V.V., Krivokolysko S.G., Litvinov V.P. (2012). The Mannich reaction in the synthesis of N,S-containing heterocycles. 11. Synthesis of 3,3′-(1,4-phenylene)-bis(8-aryl-6-oxo-3,4,7,8-tetrahydro-2H,6H-pyrido[2,1-b][1,3,5]thiadiazine-9-carbonitriles). Russ. Chem. Bull. Int. Ed..

[65] Osolodkin D.I., Kozlovskaya L.I., Dueva E.V., Dotsenko V.V., Rogova Y.V., Frolov K.A., Krivokolysko S.G., Romanova E.G., Morozov A.S., Karganova G.G. (2013). Inhibitors of Tick-Borne Flavivirus Reproduction from Structure-Based Virtual Screening. ACS Med. Chem. Lett..

[66] Dotsenko V.V., Frolov K.A., Pekhtereva T.M., Papaianina O.S., Suykov S.Y., Krivokolysko S.G. (2014). Design and synthesis of pyrido[2,1-b][1,3,5]thiadiazine library via uncatalyzed Mannich-type reaction. ACS Comb. Sci..

[67] Bibik I.V., Bibik E.Y., Dotsenko V.V., Frolov K.A., Krivokolysko S.G., Aksenov N.A., Aksenova I.V., Shcherbakov S.V., Ovcharov S.N. (2021). Synthesis and analgesic activity of new heterocyclic cyanothioacetamide derivatives. Russ. J. Gen. Chem..

[68] Krivokolysko D.S., Dotsenko V.V., Bibik E.Y., Samokish A.A., Venidiktova Y.S., Frolov K.A., Krivokolysko S.G., Vasilin V.K., Pankov A.A., Aksenov N.A. (2021). New 4-(2-furyl)-1,4-dihydronicotinonitriles and 1,4,5,6-tetrahydronicotinonitriles: Synthesis, structure, and analgesic activity. Russ. J. Gen. Chem..

[69] Krivokolysko S.G., Dyachenko V.D., Nesterov V.N., Litvinov V.P. (2001). Novel stereoselective synthesis and molecular and crystalline structure of 3-allyl-4-(4-bromophenyl)-3-cyano-6-oxopiperidine-2-thione. Chem. Heterocycl. Compd..

[70] Dotsenko V.V., Krivokolysko S.G., Chernega A.N., Litvinov V.P. (2003). Synthesis and structure of pyrido[2,1-b][1,3,5]thiadiazine derivatives. Doklady Chem..

[71] Bibik E.Y., Yaroshevskaya O.G., Devdera A.V., Demenko A.V., Zakharov V.V., Frolov K.A., Dotsenko V.V., Krivokolysko S.G. (2017). Search for anti-inflammatory agents in the tetrahydropyrido[2,1-b][1,3,5]thiadiazine series. Pharm. Chem. J..

[72] Bybik E.Y., Yaroshevskaya O.G., Demenko A.V., Frolov K.A., Dotsenko V.V., Kryvokolysko S.G. (2018). The effect of derivatives of tetrahydropyrido[2,1-b][1,3,5]thiadiazine on hematologic indices of rats with subacute parotitis. Res. Results Pharmacol..

[73] Bibik E.Y., Saphonova A.A., Yeryomin A.V., Frolov K.A., Dotsenko V.V., Krivokolysko S.G. (2017). Study of analeptic activity of tetrahydropyrido[2,1-b][1,3,5]thiadiazine derivatives. Res. Result Pharmacol. Clin. Pharmacol..

[74] Bibik E.Y., Nekrasa I.A., Demenko A.V., Frolov K.A., Dotsenko V.V., Krivokolysko S.G. (2019). Studying the adaptogenic activity of a series of tetrahydropyrido[2,1-b][1,3,5]thiadiazine derivatives. Bull. Siber. Med..

[75] Wang Y., Duraiswami C., Madauss K.P., Tran T.B., Williams S.P., Deng S.J., Graybill T.L., Hammond M., Jones D.G., Grygielko E.T. (2009). 2-Amino-9-aryl-3-cyano-4-methyl-7-oxo-6,7,8,9-tetrahydropyrido[2′,3′: 4,5]thieno[2,3-b]pyridine derivatives as selective progesterone receptor agonists. Bioorg. Med. Chem. Lett..

[76] Frolov K.A., Dotsenko V.V., Krivokolysko S.G., Litvinov V.P. (2005). Three-component condensation in the synthesis of substituted tetrahydropyridinethiolates. Russ. Chem. Bull. Int. Ed..

[77] Frolov K.A., Dotsenko V.V., Krivokolysko S.G. (2012). Synthesis and reactions of 1,2-*bis* [3-cyano-4-(2-fluorophenyl)-6-oxo-1,4,5,6-tetrahydropyridin-2-yl]diselane. Chem. Heterocycl. Compd..

[78] Baggaley K.H., Jennings L.J.A., Tyrrell A.W.R. (1982). Synthesis of 2-substituted isothiazolopyridin-3-ones. J. Heterocycl. Chem..

[79] Borgna P., Pregnolato M., Invernizzi A.G., Mellerio G. (1993). On the reaction between 3H-1,2-dithiolo[3,4-b]pyridine-3-thione and primary alkyl and arylalkylamines. J. Heterocycl. Chem..

[80] Pregnolato M., Terreni M., Ubiali D., Pagani G., Borgna P., Pastoni F., Zampollo F. (2000). 3H-[1,2]Dithiolo[3,4-b]pyridine-3-thione and its derivatives. Synthesis and antimicrobial activity. Il Farmaco.

[81] Dotsenko V.V., Krivokolysko S.G. (2013). Oxidation of thioamides with the DMSO–HCl system: A convenient and efficient method for the synthesis of 1,2,4-thiadiazoles, isothiazolo[5,4-b]pyridines, and heterocyclic disulfides. Chem. Heterocycl. Compd..

[82] Furdas S.D., Shekfeh S., Bissinger E.M., Wagner J.M., Schlimme S., Valkov V., Hendzel M., Jung M., Sippl W. (2011). Synthesis and biological testing of novel pyridoisothiazolones as histone acetyltransferase inhibitors. Bioorg. Med. Chem..

[83] Pagani G., Pregnolato M., Ubiali D., Terreni M., Piersimoni C., Scaglione F., Fraschini F., Rodríguez Gascón A., Pedraz Muñoz J.L. (2000). Synthesis and in vitro anti-mycobacterium activity of N-alkyl-1,2-dihydro-2-thioxo-3-pyridinecarbothioamides. Preliminary toxicity and pharmacokinetic evaluation. J. Med. Chem..

[84] Deeb A., Essawy A., El-Gendy A.M., Shaban A. (1990). Heterocyclic synthesis with 3-cyano-2(1H)-pyridinethione: Synthesis of 3-oxo-2,3-dihydroisothiazolo[5,4-b]pyridine and related compounds. Monatsh. Chem..

[85] Hussain S.M., Sherif S.M., Youssef M.M. (1994). New Synthesis of Polyfunctionally Substituted 2-Mercaptopyridines and Fused Pyridines. Gazz. Chim. Ital..

[86] Gompper R., Elser W. (1967). Stabile 1,4-Dipole aus Ketenacetalen und Schwefelkohlenstoff und ihre Verwendung zur Synthese von Heterocyclen. Angew. Chem..

[87] Jian F., Zheng J., Li Y., Wang J. (2009). Novel ((3*Z*,5*Z*)-3,5-bis(phenylimino)-1,2-dithiolan-4-yl) and 3H-[1,2]dithiolo[3,4-b]quinolin-4 (9H)-one heterocycles: An effective and facile green route. Green Chem..

[88] Haasnoot C.A.G., de Leeuw F.A.A.M., Altona C. (1980). The relationship between proton-proton NMR coupling constants and substituent electronegativities—I: An empirical generalization of the Karplus equation. Tetrahedron.

[89] Donders L.A., de Leeuw F.A.A.M., Altona C. (1989). Relationship between proton-proton NMR coupling constants and substituent electronegativities. IV—An extended Karplus equation accounting for interactions between substituents and its application to coupling constant data calculated by the Extended Hückel method. Magn. Reson. Chem..

[90] Mohite A.R., Bhat R.G. (2013). A practical and convenient protocol for the synthesis of (*E*)-α,β-unsaturated acids. Org. Lett..

[91] Kaupp G., Naimi-Jamal M.R., Schmeyers J. (2003). Solvent-free Knoevenagel condensations and Michael additions in the solid state and in the melt with quantitative yield. Tetrahedron.

[92] Krapivin G.D., Kul’nevich V.G., Val’ter N.I. (1989). 2,2-Dimethyl-5-(5-R-2-furfurylidene)-1,3-dioxane-4,6-diones. 4. Synthesis, stereostructures, and properties of thiophene analogs. Chem. Heterocycl. Compd..

[93] Sinotsko A.E., Dotsenko V.V., Aksenov N.A. (2021). The Reactions of N,N′-Diphenyldithiomalonamide with Michael Acceptors. Chem. Proc..

[94] Dolomanov O.V., Bourhis L.J., Gildea R.J., Howard J.A.K., Puschmann H. (2009). OLEX2: A complete structure solution, refinementand analysis program. J. Appl. Cryst..

[95] Sheldrick G.M. (2015). Crystal structure refinement with SHELXL. Acta Cryst..

