# Supplementary material for: The Reactions of N,N′-Diphenyldithiomalondiamide with Arylmethylidene Meldrum’s Acids
Source: Int J Mol Sci. 2022 Dec 15;23(24):15997. doi: 10.3390/ijms232415997 (PMC9785638; doi:10.3390/ijms232415997)

Electronic Supplementary Material file

The reactions of N,N'-diphenyldithiomalondiamide with arylmethylidene Meldrum's acids

Victor V. Dotsenko[1,2,*], Alexander V. Aksenov[2,*], Anna E. Sinotsko[1], Ekaterina A. Varzieva[1], Alena A. Russkikh[1], Arina G. Levchenko[1], Nicolai A. Aksenov[2] and Inna V. Aksenova[2]

[1]Department of Organic Chemistry and Technologies, Kuban State University, 149 Stavropolskaya St., 350040 Krasnodar, Russia;

[2]Department of Chemistry, North Caucasus Federal University, 1a Pushkin St., 355017 Stavropol, Russia;

*Correspondence: victor_dotsenko_@mail.ru (V.V.D.), aaksenov@ncfu.ru (A.V.A.).

Contents

Figure S1. ^{1}H NMR spectrum of the Michael adduct 15a', DMSO-d_6 (400 MHz) (Table 1, entry 1) 6
Figure S3. FTIR spectrum of the Michael adduct 15a' (Table 1, entry 1) 7
Figure S4. FTIR spectrum of the Michael adduct 15a (Table 1, entry 4) 7
Figure S5. ^{1}H NMR spectrum of the Michael adduct 15a, DMSO-d_6 (400 MHz) (Table 1, entry 4) 8
Figure S6. ^{13}C NMR spectrum of the Michael adduct 15a, DMSO-d_6 (101 MHz) (Table 1, entry 4) 8
Figure S7. ^{1}H–^{13}C HSQC NMR spectrum of the Michael adduct 15a, DMSO-d_6 (400/101 MHz) (Table 1, entry 4) 9
Figure S8. ^{1}H–^{13}C HSQC NMR spectrum of the Michael adduct 15a, DMSO-d_6 (400/101 MHz) (Table 1, entry 4) (*fragment*) 10
Figure S9. ^{1}H–^{13}C HSQC NMR spectrum of the Michael adduct 15a, DMSO-d_6 (400/101 MHz) (Table 1, entry 4) (*fragment*) 11
Figure S10. ^{1}H–^{13}C HMBC NMR spectrum of the Michael adduct 15a, DMSO-d_6 (400/101 MHz) (Table 1, entry 4) 12
Figure S11. ^{1}H–^{13}C HMBC NMR spectrum of the Michael adduct 15a, DMSO-d_6 (400/101 MHz) (Table 1, entry 4) (*fragments*) 13
Figure S12. ^{1}H–^{13}C HMBC NMR spectrum of the Michael adduct 15a, DMSO-d_6 (400/101 MHz) (Table 1, entry 4) (*fragments*) 14
Table S1. The observed correlations in the ^{1}H-^{13}C HSQC and ^{1}H-^{13}C HMBC 2D NMR spectra of the Michael adduct 15a (Table 1, entry 4) 15
Figure S13. ^{1}H NMR spectrum of a mixture of Michael adduct 15a and dithiolopyridine 17a, DMSO-d_6 (400 MHz) (Table 1, entry 5) 16
Figure S14. ^{13}C DEPTQ NMR spectrum of a mixture of Michael adduct 15a and dithiolopyridine 17a, DMSO-d_6 (101 MHz) (Table 1, entry 5) 16

Figure S15. Comparison of ^{13}C DEPTQ NMR spectrum of pure 15a (Table 1, entry 4) with the spectrum of a mixture of Michael adduct 15a and dithiolopyridine 17a, DMSO-d_6 (101 MHz) (Table 1, entry 5) 17
Figure S16. ^{1}H–^{13}C HSQC NMR spectrum of a mixture of Michael adduct 15a and dithiolopyridine 17a, DMSO-d_6 (400/101 MHz) (Table 1, entry 5).......... 18
Figure S17. ^{1}H–^{13}C HSQC NMR spectrum of a mixture of Michael adduct 15a and dithiolopyridine 17a, DMSO-d_6 (400/101 MHz) (Table 1, entry 5) (*fragment*) 19
Figure S18. ^{1}H–^{13}C HMBC NMR spectrum of a mixture of Michael adduct 15a and dithiolopyridine 17a, DMSO-d_6 (400/101 MHz) (Table 1, entry 5).......... 20
Figure S19. ^{1}H–^{13}C HMBC NMR spectrum of a mixture of Michael adduct 15a and dithiolopyridine 17a, DMSO-d_6 (400/101 MHz) (Table 1, entry 5) (*fragment*) 21
Figure S20. ^{1}H–^{13}C HMBC NMR spectrum of a mixture of Michael adduct 15a and dithiolopyridine 17a, DMSO-d_6 (400/101 MHz) (Table 1, entry 5) (*fragment*) 22
Figure S21. ^{1}H–^{13}C HMBC NMR spectrum of a mixture of Michael adduct 15a and dithiolopyridine 17a, DMSO-d_6 (400/101 MHz) (Table 1, entry 5) (*fragment*) 23
Figure S22. ^{1}H NMR spectrum of the Michael adduct 15b, DMSO-d_6 (400 MHz) (Table 1, entry 6) 24
Figure S23. ^{13}C DEPTQ NMR spectrum of the Michael adduct 15b, DMSO-d_6 (101 MHz) (Table 1, entry 6) 24
Figure S24. ^{1}H–^{13}C HSQC NMR spectrum of the Michael adduct 15b, DMSO-d_6 (400/101 MHz) (Table 1, entry 6) 25
Figure S25. ^{1}H–^{13}C HSQC NMR spectrum of the Michael adduct 15b, DMSO-d_6 (400/101 MHz) (Table 1, entry 6) (*fragments*).......... 26
Figure S26. ^{1}H–^{13}C HMBC NMR spectrum of the Michael adduct 15b, DMSO-d_6 (400/101 MHz) (Table 1, entry 6) 27
Figure S27. ^{1}H–^{13}C HMBC NMR spectrum of the Michael adduct 15b, DMSO-d_6 (400/101 MHz) (Table 1, entry 6) (*fragments*).......... 28
Table S2. The observed correlations in the ^{1}H-^{13}C HSQC and ^{1}H-^{13}C HMBC 2D NMR spectra of the Michael adduct 15b (Table 1, entry 6) 29
Figure S28. ^{1}H NMR spectrum of a mixture of Michael adduct 15b and thiolate 16b, DMSO-d_6 (400 MHz) (Table 1, entry 7) 30
Figure S29. ^{13}C DEPTQ NMR spectrum of a mixture of Michael adduct 15b and thiolate 16b, DMSO-d_6 (101 MHz) (Table 1, entry 7).......... 30
Figure S30. Comparison of ^{13}C DEPTQ NMR spectrum of pure 15b (Table 1, entry 4) with the spectrum of a mixture of Michael adduct 15b and pyridine-2-thiolate 16b, DMSO-d_6 (101 MHz) (Table 1, entry 7) 31
Figure S31. Comparison of ^{13}C DEPTQ NMR spectrum of pure 15b (Table 1, entry 4) with the spectrum of a mixture of Michael adduct 15b and pyridine-2-thiolate 16b, DMSO-d_6 (101 MHz) (Table 1, entry 7) (*continued*) 32
Figure S32. ^{1}H–^{13}C HSQC NMR spectrum of a mixture of Michael adduct 15b and pyridine-2-thiolate 16b, DMSO-d_6 (400/101 MHz) (Table 1, entry 7) 33
Figure S33. ^{1}H–^{13}C HSQC NMR spectrum of a mixture of Michael adduct 15b and pyridine-2-thiolate 16b, DMSO-d_6 (400/101 MHz) (Table 1, entry 7) (*fragments*) 34

Figure S34. ^1H–^{13}C HMBC NMR spectrum of a mixture of Michael adduct 15b and pyridine-2-thiolate 16b, DMSO-d_6 (400/101 MHz) (Table 1, entry 7) 35
Figure S35. ^1H–^{13}C HMBC NMR spectrum of a mixture of Michael adduct 15b and pyridine-2-thiolate 16b, DMSO-d_6 (400/101 MHz) (Table 1, entry 7) (*fragments*) 36
Figure S36. ^1H–^{13}C HMBC NMR spectrum of a mixture of Michael adduct 15b and pyridine-2-thiolate 16b, DMSO-d_6 (400/101 MHz) (Table 1, entry 7) (*fragments*) 37
Figure S37. ^1H NMR spectrum of the Michael adduct 15c, DMSO-d_6 (400 MHz) (Table 1, entry 8) 38
Figure S38. ^{13}C DEPTQ NMR spectrum of the Michael adduct 15c, DMSO-d_6 (101 MHz) (Table 1, entry 8) 38
Figure S39. ^1H–^{13}C HSQC NMR spectrum of the Michael adduct 15c, DMSO-d_6 (400/101 MHz) (Table 1, entry 8) 39
Figure S40. ^1H–^{13}C HSQC NMR spectrum of the Michael adduct 15c, DMSO-d_6 (400/101 MHz) (Table 1, entry 8) (*fragments*) 40
Figure S41. ^1H–^{13}C HMBC NMR spectrum of the Michael adduct 15c, DMSO-d_6 (400/101 MHz) (Table 1, entry 8) 40
Figure S42. ^1H–^{13}C HMBC NMR spectrum of the Michael adduct 15c, DMSO-d_6 (400/101 MHz) (Table 1, entry 8) (*fragments*) 42
Table S3. The observed correlations in the ^1H-^{13}C HSQC and ^1H-^{13}C HMBC 2D NMR spectra of the Michael adduct 15c (Table 1, entry 8) 43
Figure S43. FTIR spectrum of the Michael adduct 15c (Table 1, entry 8) 44
Figure S44. ^1H NMR spectrum of the Michael adduct 15c, DMSO-d_6 (400 MHz) (Table 1, entry 9) 45
Figure S45. ^{13}C DEPTQ NMR spectrum of the Michael adduct 15c, DMSO-d_6 (101 MHz) (Table 1, entry 9) 45
Figure S46. ^1H NMR spectrum of a mixture of Michael adduct 15d and cyclization product 16d, DMSO-d_6 (400 MHz) (Table 1, entry 11) 46
Figure S47. ^{13}C DEPTQ NMR spectrum of a mixture of Michael adduct 15d and cyclization product 16d, DMSO-d_6 (101 MHz) (Table 1, entry 11) 46
Figure S48. ^1H–^{13}C HSQC NMR spectrum of a mixture of Michael adduct 15d and cyclization product 16d, DMSO-d_6 (400/101 MHz) (Table 1, entry 11) 47
Figure S49. ^1H–^{13}C HSQC NMR spectrum of a mixture of Michael adduct 15d and cyclization product 16d, DMSO-d_6 (400/101 MHz) (Table 1, entry 11) (*fragments*) 48
Figure S50. ^1H–^{13}C HMBC NMR spectrum of a mixture of Michael adduct 15d and cyclization product 16d, DMSO-d_6 (400/101 MHz) (Table 1, entry 11) 49
Figure S51. ^1H–^{13}C HMBC NMR spectrum of a mixture of Michael adduct 15d and cyclization product 16d, DMSO-d_6 (400/101 MHz) (Table 1, entry 11) (*fragments*) 50
Figure S52. ^1H NMR spectrum of a mixture of Michael adduct 15e and cyclization product 16e, DMSO-d_6 (400 MHz) (Table 1, entry 12) 51
Figure S53. ^{13}C DEPTQ NMR spectrum of a mixture of Michael adduct 15e and cyclization product 16e, DMSO-d_6 (101 MHz) (Table 1, entry 12) 51
Figure S54. ^1H NMR spectrum of a mixture of 15g,16g,17g, DMSO-d_6 (400 MHz) (Table 1, entry 15) 52

Figure S55. ^{13}C DEPTQ NMR spectrum of a mixture of 15g,16g,17g, DMSO-d_6 (101 MHz) (Table 1, entry 15) ... 52
Figure S56. ^{1}H NMR spectrum of the products of reaction of 4-methoxybenzylidene Meldrum's acid 14h with dithiomalondianilide 1, DMSO-d_6 (400 MHz) (Table 1, entry 17) 53
Figure S57. ^{13}C DEPTQ NMR spectrum of the products of reaction of 4-methoxybenzylidene Meldrum's acid 14h with dithiomalondianilide 1, DMSO-d_6 (400 MHz) (Table 1, entry 17) ... 53
Figure S58. ^{1}H NMR spectrum of the products of reaction of 4-hydroxybenzylidene Meldrum's acid 14i with dithiomalondianilide 1, DMSO-d_6 (400 MHz) (Table 1, entry 18) 54
Figure S59. ^{13}C DEPTQ NMR spectrum of the products of reaction of 4-hydroxybenzylidene Meldrum's acid 14i with dithiomalondianilide 1, DMSO-d_6 (101 MHz) (Table 1, entry 18) ... 54
Figure S60. ^{1}H–^{13}C HSQC NMR spectrum of the products of reaction of 4-hydroxybenzylidene Meldrum's acid 14i with dithiomalondianilide 1, DMSO-d_6 (400/101 MHz) (Table 1, entry 18) ... 55
Figure S61. ^{1}H–^{13}C HSQC NMR spectrum of the products of reaction of 4-hydroxybenzylidene Meldrum's acid 14i with dithiomalondianilide 1, DMSO-d_6 (400/101 MHz) (Table 1, entry 18) (*fragments*) ... 56
Figure S62. ^{1}H–^{13}C HMBC NMR spectrum of the products of reaction of 4-hydroxybenzylidene Meldrum's acid 14i with dithiomalondianilide 1, DMSO-d_6 (400/101 MHz) (Table 1, entry 18) ... 57
Figure S63. ^{1}H–^{13}C HMBC NMR spectrum of the products of reaction of 4-hydroxybenzylidene Meldrum's acid 14i with dithiomalondianilide 1, DMSO-d_6 (400/101 MHz) (Table 1, entry 18) (*fragments*) ... 58
Figure S64. ^{1}H NMR spectrum of 4-(4-(dimethylamino)phenyl)-7-phenyl-3-(phenylimino)-4,5-dihydro-3H-[1,2]dithiolo[3,4-b]pyridin-6(7H)-one 17j, DMSO-d_6 (400 MHz) (Table 1, entry 19) ... 59
Figure S65. ^{13}C DEPTQ NMR spectrum of 4-(4-(dimethylamino)phenyl)-7-phenyl-3-(phenylimino)-4,5-dihydro-3H-[1,2]dithiolo[3,4-b]pyridin-6(7H)-one 17j, DMSO-d_6 (101 MHz) (Table 1, entry 19) ... 59
Figure S66. ^{1}H–^{13}C HSQC NMR spectrum of 4-(4-(dimethylamino)phenyl)-7-phenyl-3-(phenylimino)-4,5-dihydro-3H-[1,2]dithiolo[3,4-b]pyridin-6(7H)-one 17j, DMSO-d_6 (400/101 MHz) (Table 1, entry 19) ... 60
Figure S67. ^{1}H–^{13}C HSQC NMR spectrum of 4-(4-(dimethylamino)phenyl)-7-phenyl-3-(phenylimino)-4,5-dihydro-3H-[1,2]dithiolo[3,4-b]pyridin-6(7H)-one 17j, DMSO-d_6 (400/101 MHz) (Table 1, entry 19) (*fragments*) ... 61
Figure S68. ^{1}H–^{13}C HMBC NMR spectrum of 4-(4-(dimethylamino)phenyl)-7-phenyl-3-(phenylimino)-4,5-dihydro-3H-[1,2]dithiolo[3,4-b]pyridin-6(7H)-one 17j, DMSO-d_6 (400/101 MHz) (Table 1, entry 19) ... 62
Figure S69. ^{1}H–^{13}C HMBC NMR spectrum of 4-(4-(dimethylamino)phenyl)-7-phenyl-3-(phenylimino)-4,5-dihydro-3H-[1,2]dithiolo[3,4-b]pyridin-6(7H)-one 17j, DMSO-d_6 (400/101 MHz) (Table 1, entry 19) (*fragments*) ... 63
Table S4. The observed correlations in the ^{1}H-^{13}C HSQC and ^{1}H-^{13}C HMBC 2D NMR spectra of [1,2]dithiolo[3,4-b]pyridine 17j (Table 1, entry 19) ... 64

Figure S70. ^{1}H NMR spectrum of 4-(2-chlorophenyl)-7-phenyl-3-(phenylimino)-4,5-dihydro-3H-[1,2]dithiolo[3,4-b]pyridin-6(7H)-one 17c, DMSO-d_6 (400 MHz) 65
Figure S71. ^{13}C DEPTQ NMR spectrum of 4-(2-chlorophenyl)-7-phenyl-3-(phenylimino)-4,5-dihydro-3H-[1,2]dithiolo[3,4-b]pyridin-6(7H)-one 17c, DMSO-d_6 (101 MHz) 65
Figure S72. FTIR spectrum of 4-(2-chlorophenyl)-7-phenyl-3-(phenylimino)-4,5-dihydro-3H-[1,2]dithiolo[3,4-b]pyridin-6(7H)-one 17c (ATR mode) 66
Figure S73. ORTEP drawings of the crystal structure showing 50% probability thermal ellipsoids (CCDC 2218133) and microphotography of the single crystal of compound 15b used for X-Ray diffraction analysis at the bottom 67
Table S5. Crystal data and structure refinement for N-methylmorpholinium 2,2-dimethyl-5-(1-(2-nitrophenyl)-3-(phenylamino)-2-(N-phenylthiocarbamoyl)-3-thioxopropyl)-4-oxo-4H-1,3-dioxin-6-olate 15b 68
Table S6. Fractional Atomic Coordinates (×10^4) and Equivalent Isotropic Displacement Parameters (Å^2×10^3) for adduct 15b. U_{eq} is defined as 1/3 of of the trace of the orthogonalised U_{IJ} tensor. 69
Table S7. Anisotropic Displacement Parameters (Å^2×10^3) for 15b. The Anisotropic displacement factor exponent takes the form: $-2\pi^2[h^2a^{*2}U_{11}+...+2hka\times b\times U_{12}]$ 71
Table S8. Bond Lengths for adduct 15b. 73
Table S9. Bond Angles for adduct 15b. 74
Table S10. Hydrogen Bonds for adduct 15b. 75
Table S11. Hydrogen Atom Coordinates (Å×10^4) and Isotropic Displacement Parameters (Å^2×10^3) for adduct 15b. 76
Figure S74. HRMS data for compound 15a' (Table 1, entry 1) 78
Figure S75. HRMS data for a mixture of Michael adduct 15a and dithiolopyridine 17a (Table 1, entry 5) 79
Figure S76. HRMS data for compound 15b (Table 1, entry 6) 80
Figure S77. HRMS data for Michael adduct 15d and cyclization product 16d (Table 1, entry 11) 81
Figure S78. HRMS data for a mixture of Michael adduct 15e and cyclization product 16e. 82
Figure S79. HRMS data for a mixture of Michael adduct 15f and by-products 16f,17f (Table 1, entry 14) 83
Figure S80. HRMS data for a mixture of Michael adduct 15h and by-products 16h,17h (Table 1, entry 17) 84
Figure S81. HRMS data for a mixture of Michael adduct 15i and by-products 16i and strating dithiomalondianilide (Table 1, entry 18) 85

Figure S1. ^{1}H NMR spectrum of the Michael adduct 15a', DMSO-d_6 (400 MHz) (Table 1, entry 1)

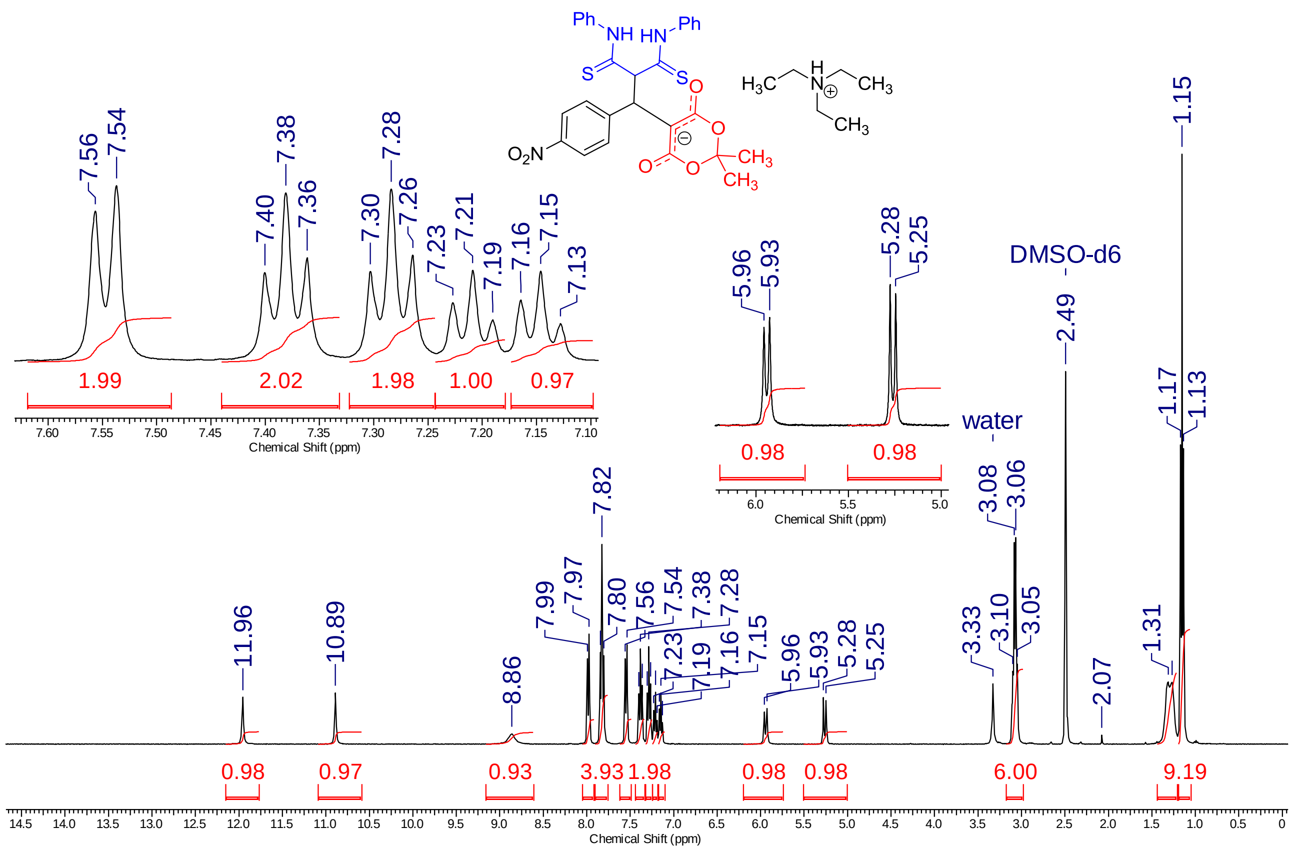

Figure S2. ^{13}C DEPTQ NMR spectrum of the Michael adduct 15a', DMSO-d_6 (101 MHz) (Table 1, entry 1)

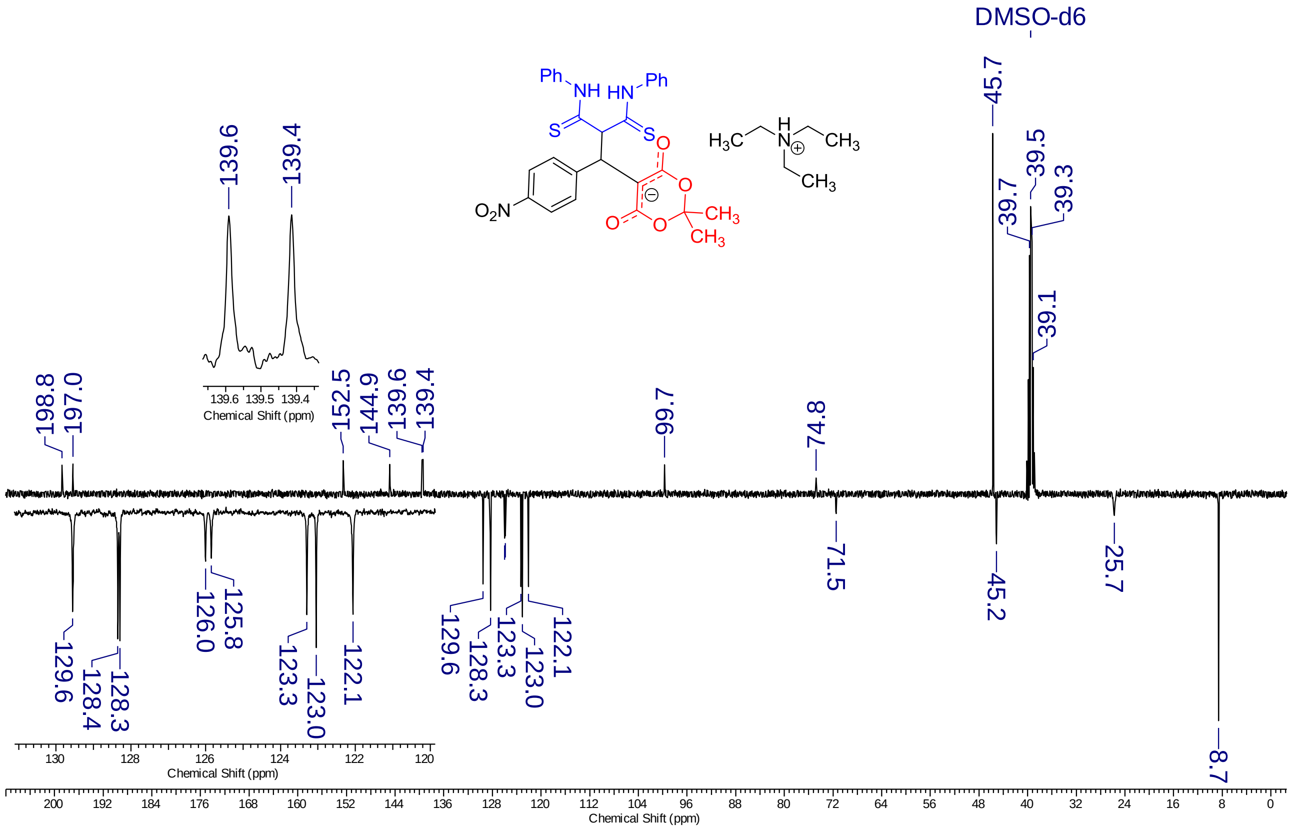

Figure S3. FTIR spectrum of the Michael adduct 15a' (Table 1, entry 1)

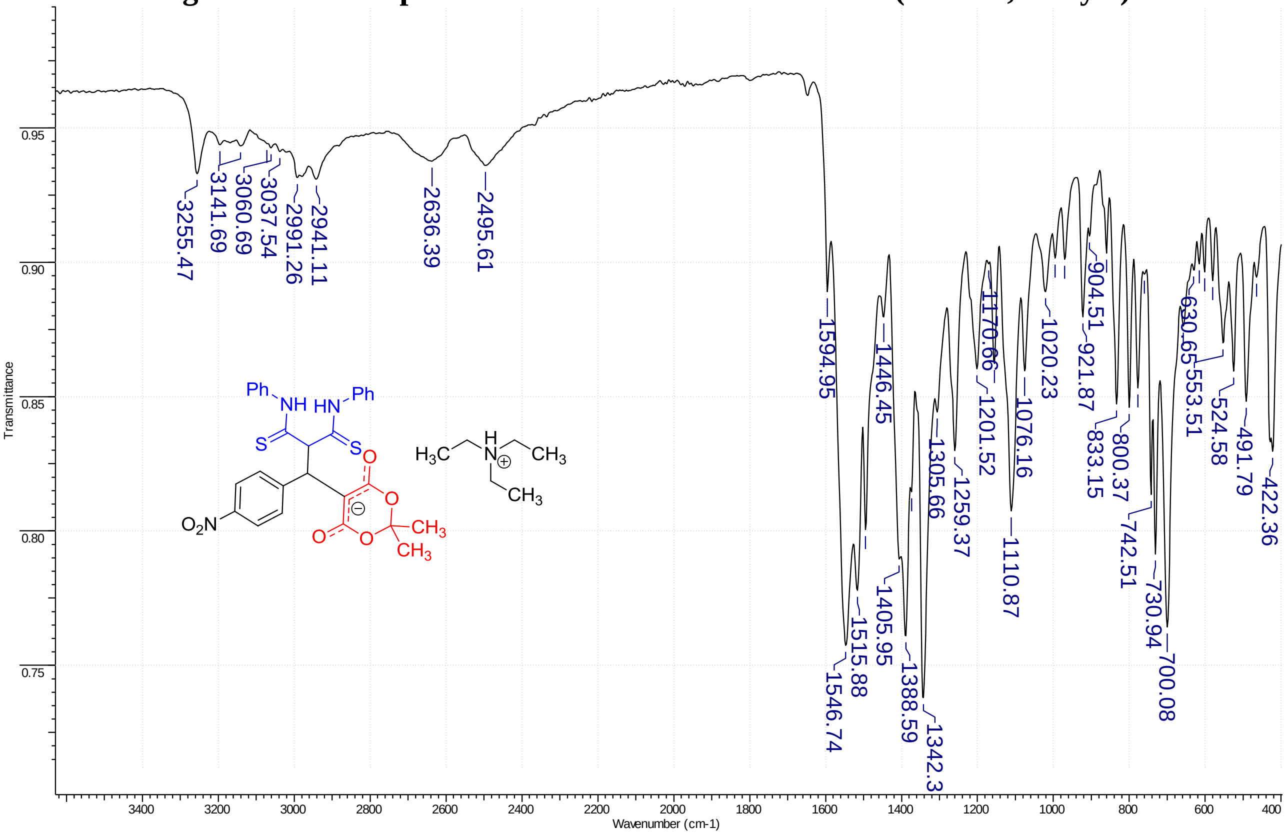

Figure S4. FTIR spectrum of the Michael adduct 15a (Table 1, entry 4)

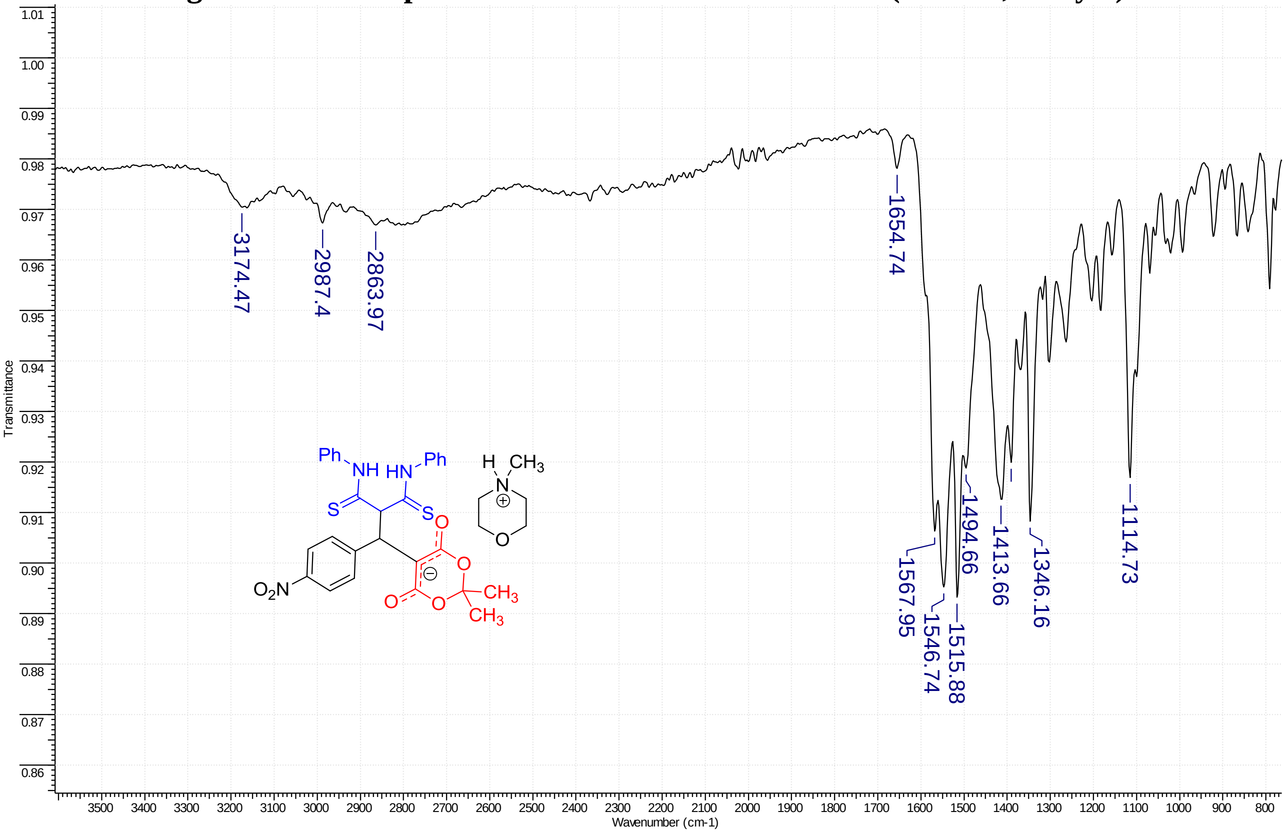

Figure S5. ^{1}**H NMR spectrum of the Michael adduct 15a, DMSO-d$_6$ (400 MHz) (Table 1, entry 4)**

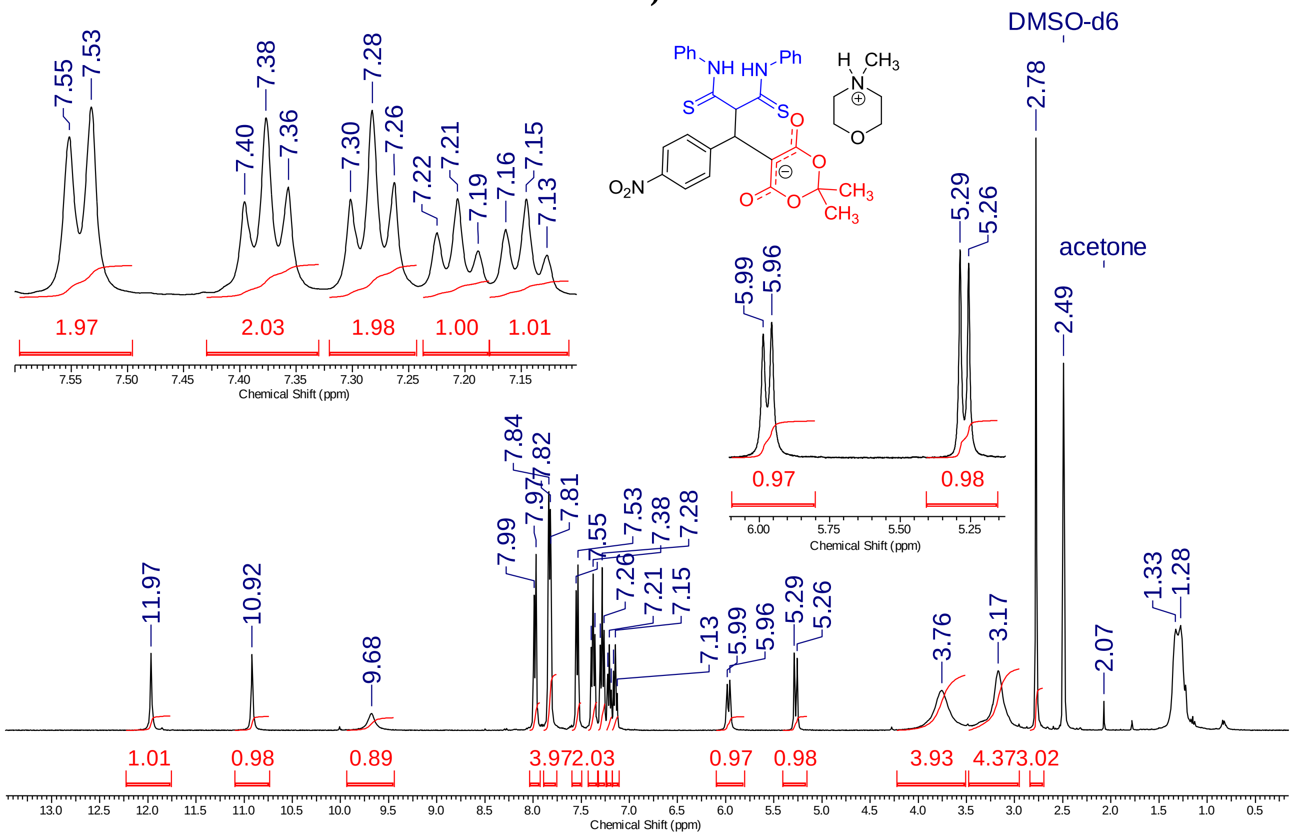

Figure S6. ^{13}**C NMR spectrum of the Michael adduct 15a, DMSO-d$_6$ (101 MHz) (Table 1, entry 4)**

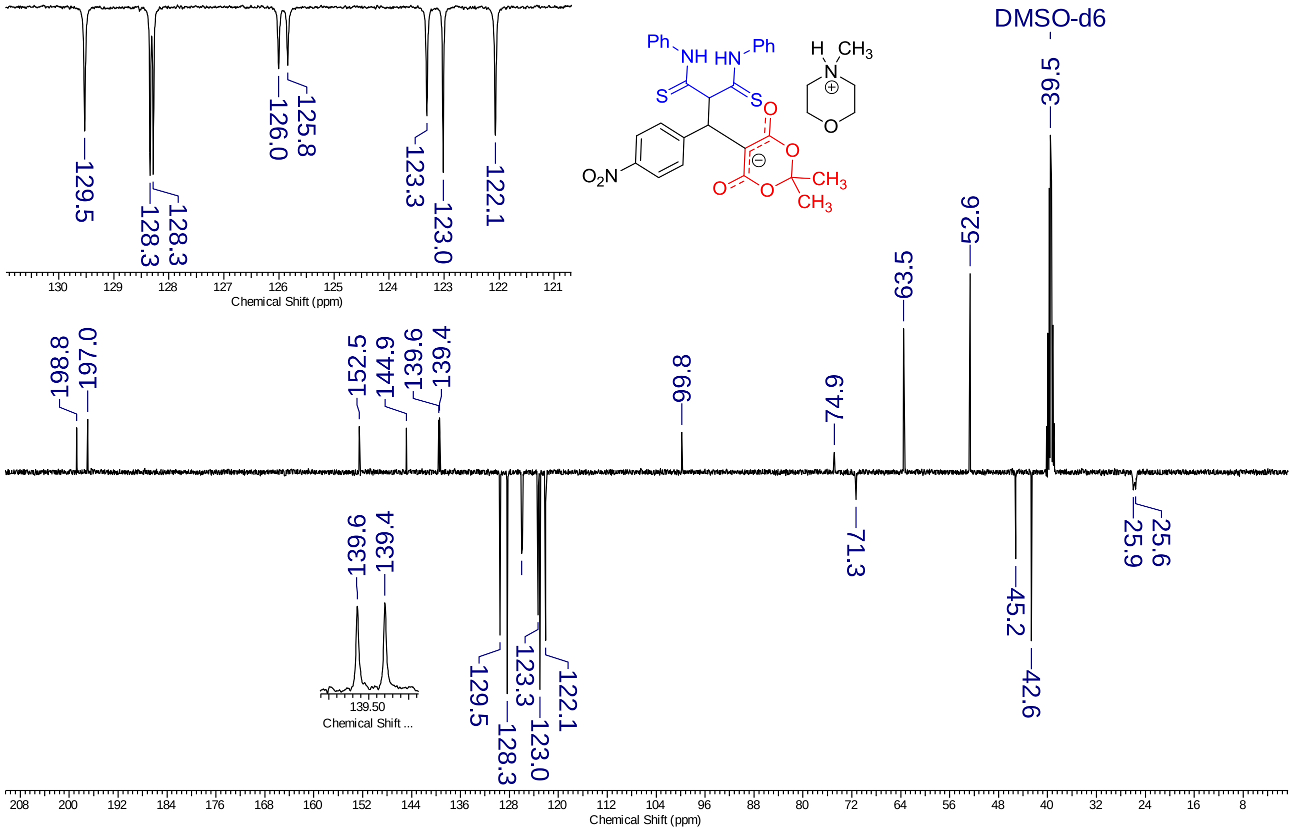

Figure S7. ^{1}H–^{13}C **HSQC NMR spectrum of the Michael adduct 15a, DMSO-d_6 (400/101 MHz) (Table 1, entry 4)**

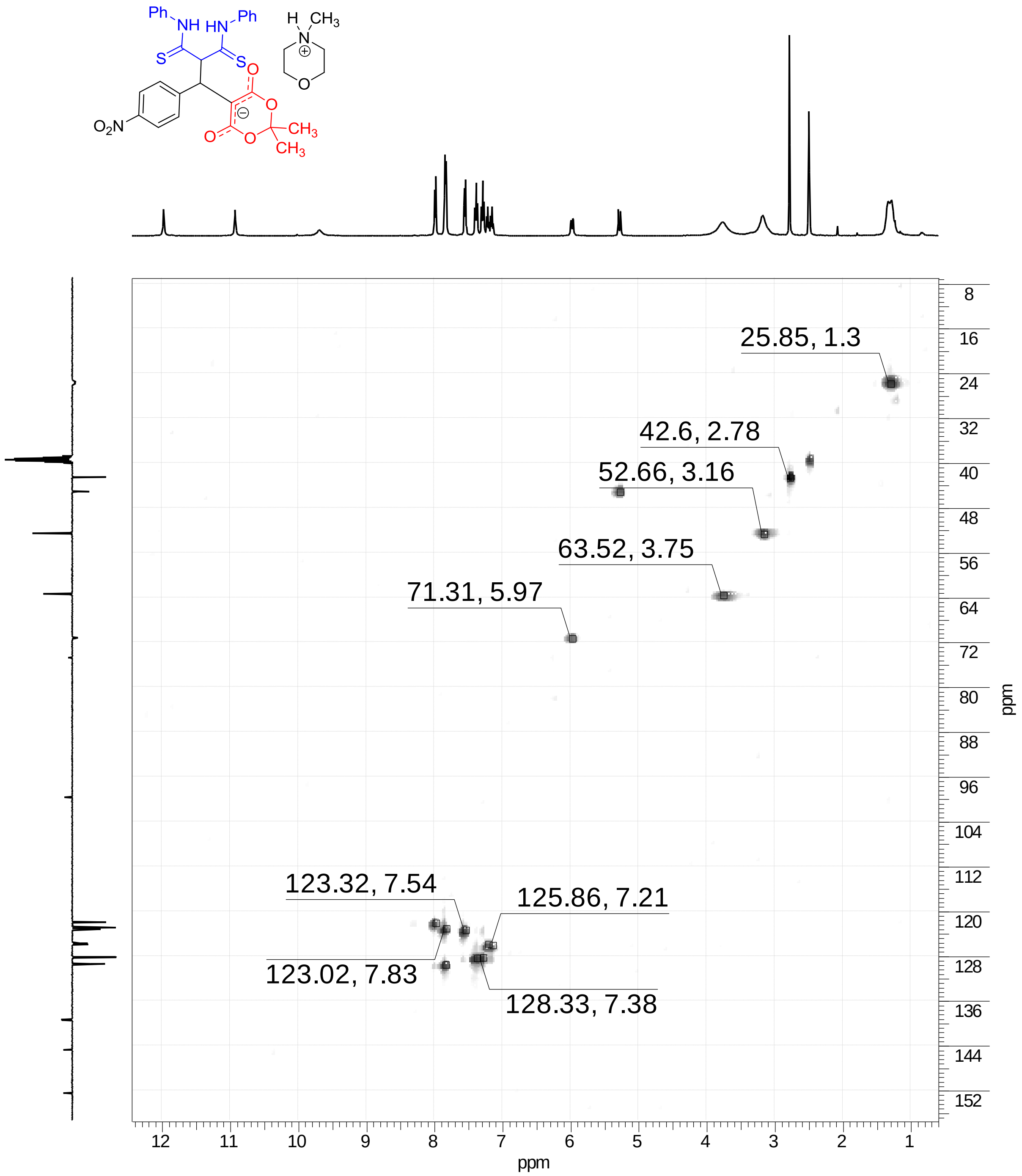

Figure S8. ^{1}H–^{13}C **HSQC NMR spectrum of the Michael adduct 15a, DMSO-d_6 (400/101 MHz) (Table 1, entry 4) (*fragment*)**

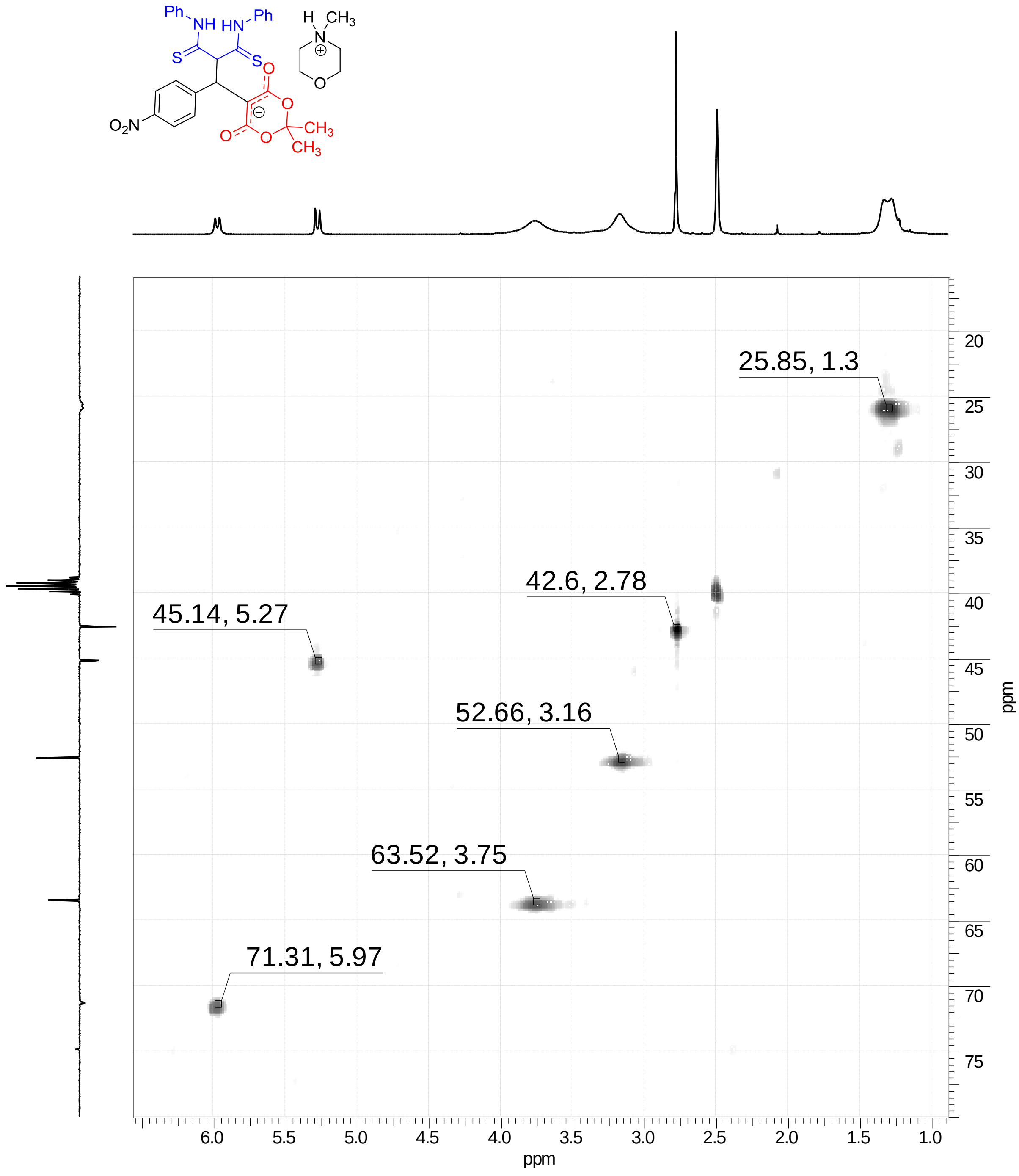

Figure S9. $^{1}H–^{13}C$ **HSQC NMR spectrum of the Michael adduct 15a, DMSO-d$_6$ (400/101 MHz) (Table 1, entry 4) (*fragment*)**

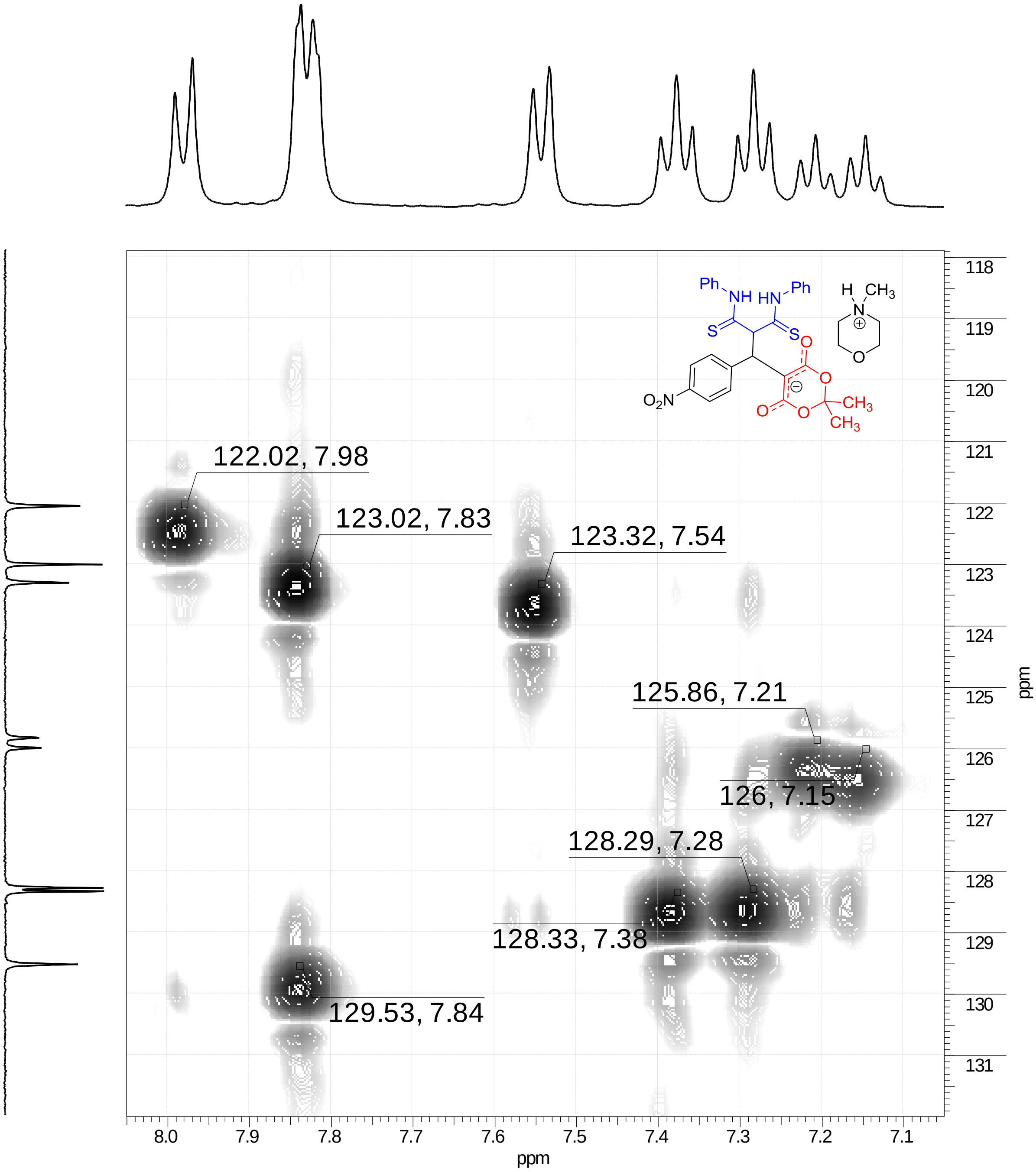

Figure S10. ^{1}H–^{13}C **HMBC NMR spectrum of the Michael adduct 15a, DMSO-d_6 (400/101 MHz) (Table 1, entry 4)**

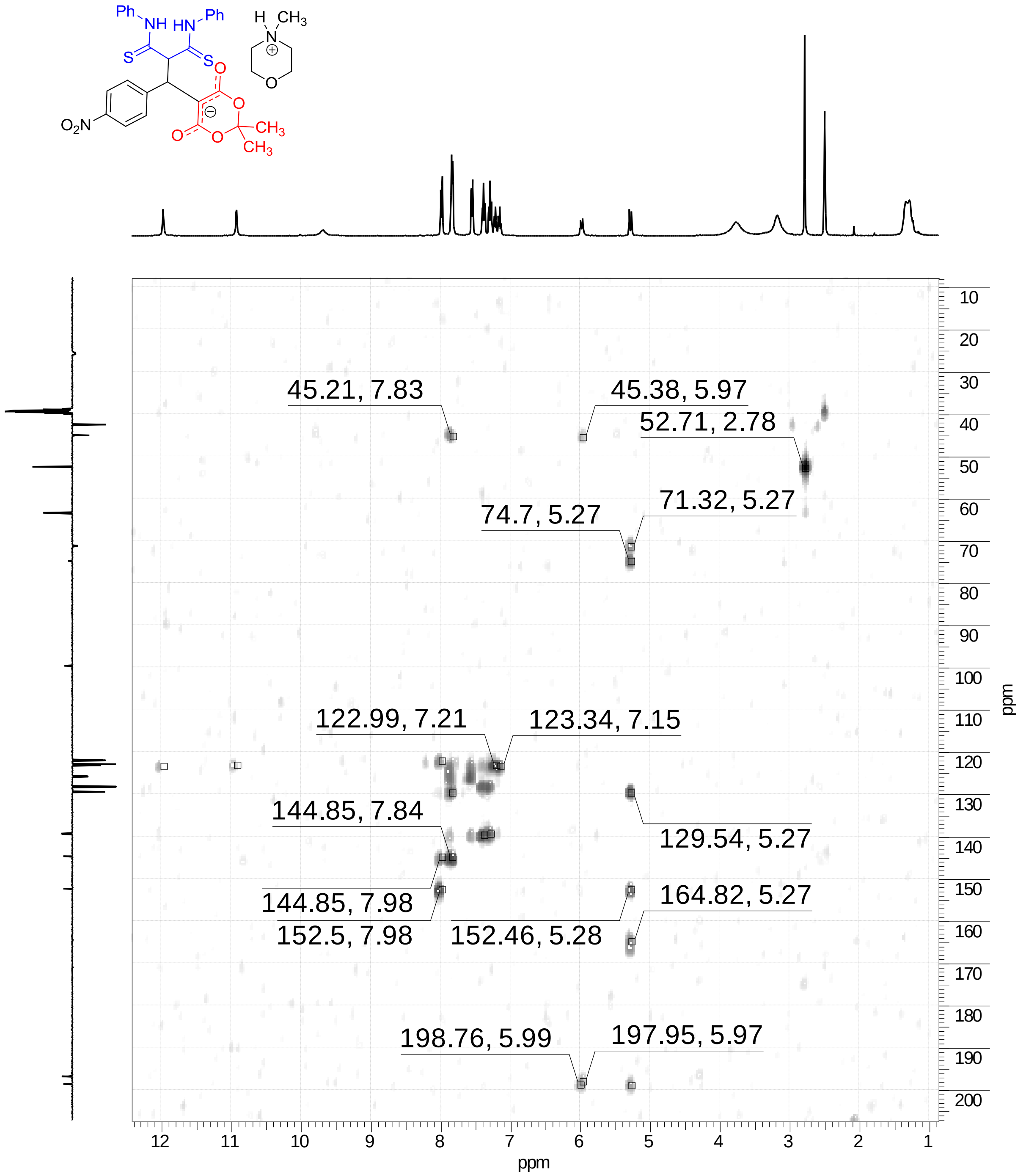

Figure S11. ^{1}H–^{13}C HMBC NMR spectrum of the Michael adduct 15a, DMSO-d_6 (400/101 MHz) (Table 1, entry 4) (*fragments*)

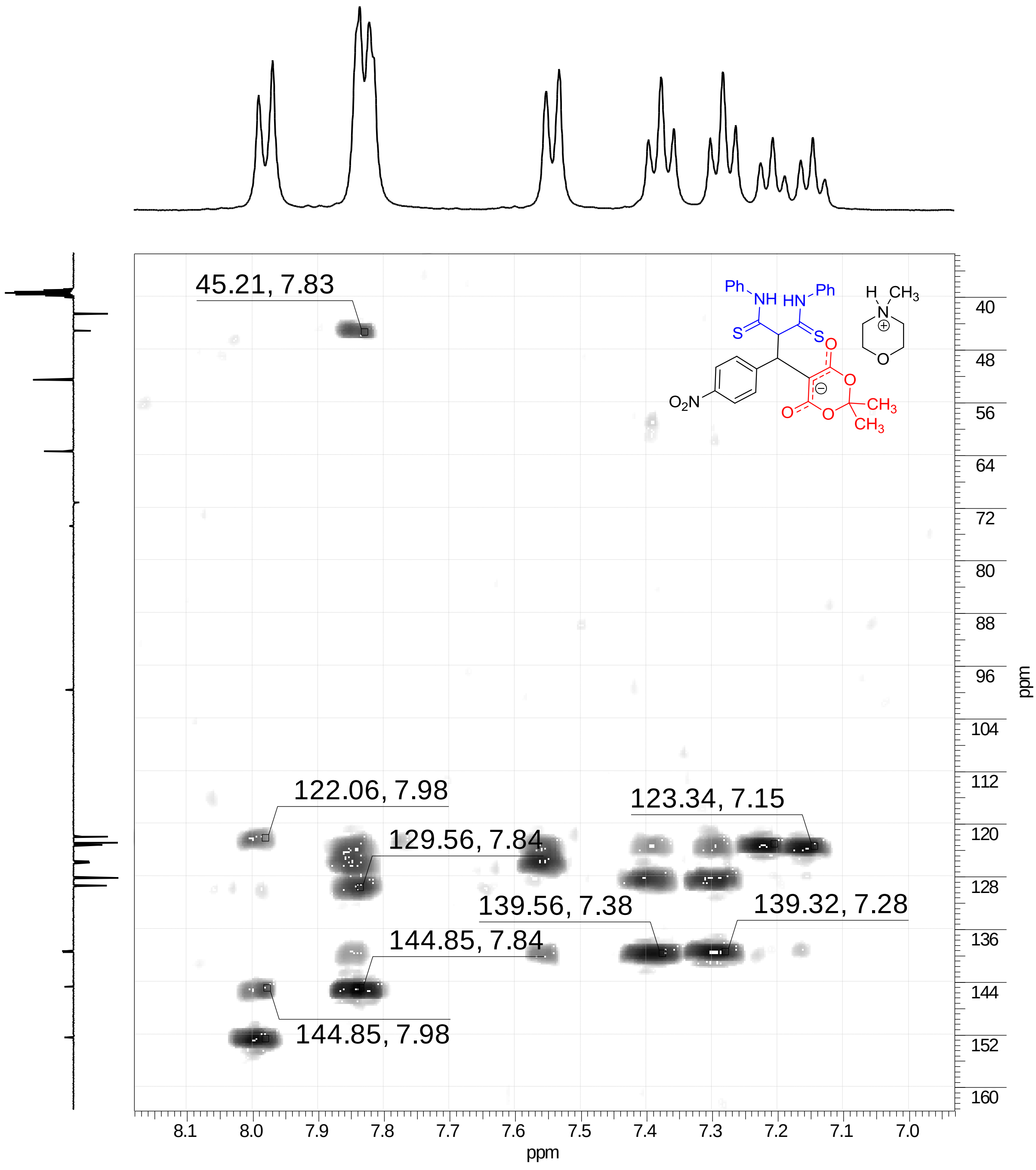

Figure S12. ^{1}H–^{13}C **HMBC NMR spectrum of the Michael adduct 15a, DMSO-d_6 (400/101 MHz) (Table 1, entry 4) (*fragments*)**

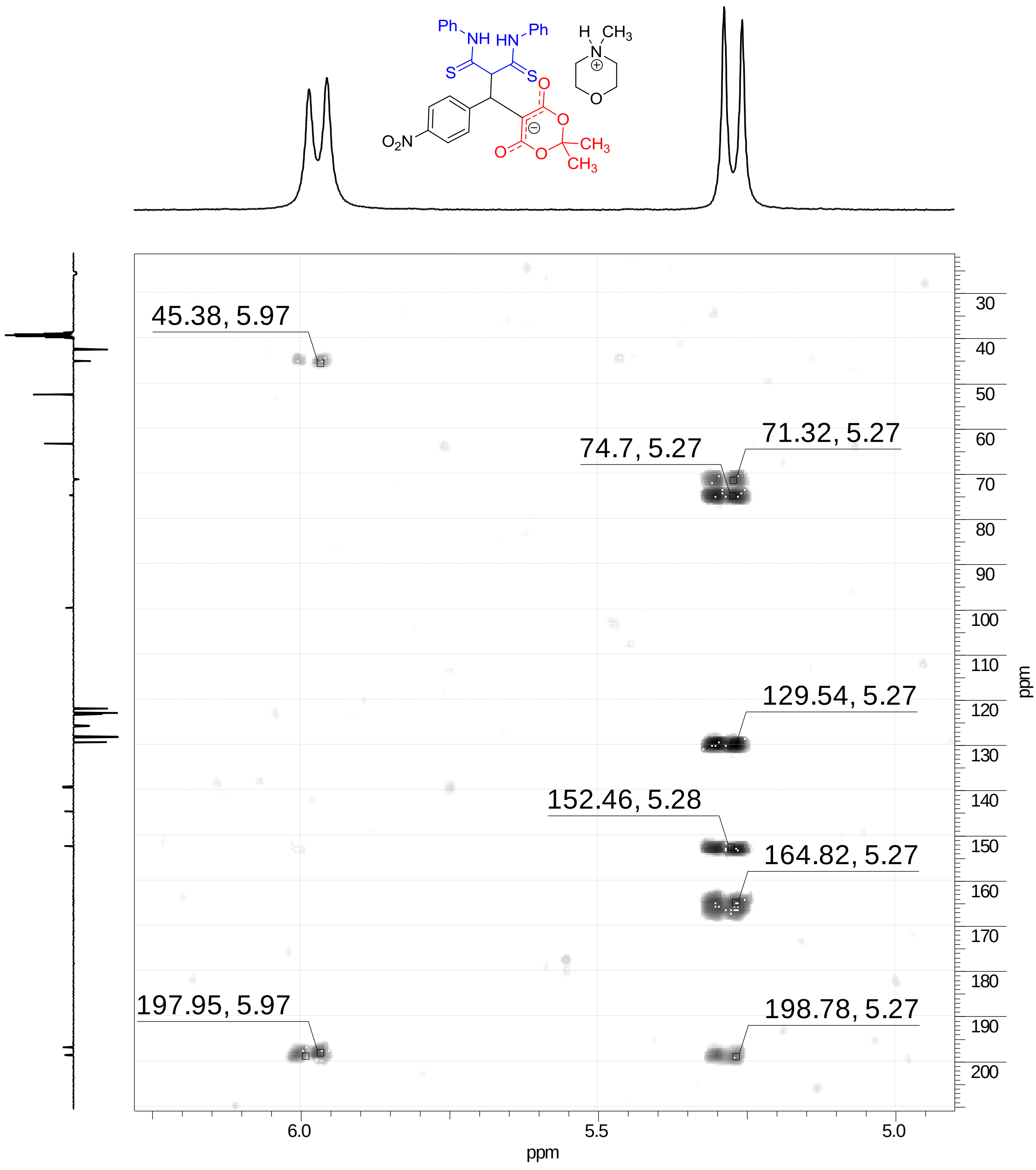

Table S1. The observed correlations in the ^{1}H-^{13}C HSQC and ^{1}H-^{13}C HMBC 2D NMR spectra of the Michael adduct 15a (Table 1, entry 4)

^{13}C chemical shifts are given in **red**, ^{1}H shifts – in **blue**

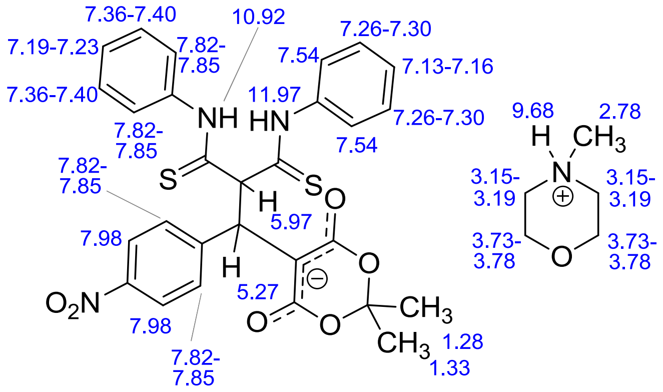

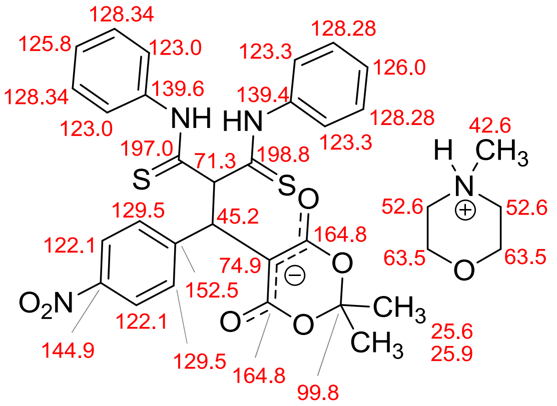

^{1}H NMR shifts, δ, ppm	Correlations in HSQC spectrum, δ, ppm	Correlations in HMBC spectrum, δ, ppm
1.28 (br s, 3H, Me)<br>1.33 (br s, 3H, Me)	25.6* (CH_3)<br>25.9* (CH_3)	–
2.78 (s, 3H, NMe)	42.6* (N–CH_3)	52.6 (CH_2NCH_2)
3.15-3.19 (m, 4H, CH_2NCH_2)	52.6 (CH_2NCH_2)	–
3.73-3.78 (m, 4H, CH_2OCH_2)	63.5 (CH_2OCH_2)	–
5.27 (d, ^{3}J = 12.1 Hz, 1H, CH)	45.2* (<u>C</u>H–Ar)	71.3* (<u>C</u>H–CSNHPh), 74.9 (<u>C</u>–C=O), 129.5* (C-2 C-6 4-$NO_2C_6H_4$), 152.5 (C–1 4-$NO_2C_6H_4$), 164.8 (C–O^-), 197.0 (C=S), 198.8 (C=S)
5.97 (d, ^{3}J = 12.1 Hz, 1H, CH)	71.3* (<u>C</u>H–CSNHPh)	45.2* (<u>C</u>H–Ar), 197.0 (C=S), 198.8 (C=S).
7.13-7.16 (m, 1H, H-4 Ph)	126.0* (C-4 Ph)	123.3* (C-2, C-6 Ph)
7.19-7.23 (m 1H, H-4 Ph)	125.8* (C-4 Ph)	123.0* (C-2, C-6 Ph)
7.26-7.30 (m, 2H, H-3, H-5 Ph)	128.28* (C-3, C-5 Ph)	123.3* (C-2, C-6 Ph), 128.28* (C-3, C-5 Ph), 139.4 (C-1 Ph)
7.36-7.40 (m, 2H, H-3, H-5 Ph)	128.34* (C-3, C-5 Ph)	123.0* (C-2, C-6 Ph), 128.34* (C-3, C-5 Ph), 139.6 (C-1 Ph)
7.54 (d, ^{3}J = 7.8 Hz, 2H, H-2, H-6 Ph)	123.3* (C-2, C-6 Ph)	123.3* (C-2, C-6 Ph), 126.0* (C-4 Ph), 139.4 (C-1 Ph)
7.82-7.85 (m, 4H, H-2, H-6 Ph and H-2, H-6 4-$NO_2C_6H_4$ overlapped)	123.0* (C-2, C-6 Ph), 129.5* (C-2 C-6 4-$NO_2C_6H_4$)	45.2* (<u>C</u>H–Ar), 123.0* (C-2, C-6 Ph), 125.8* (C-4 Ph), 139.6 (C-1 Ph), 144.9 (C-4 4-$NO_2C_6H_4$)
7.98 (d, ^{3}J = 8.6 Hz, 2H, H-3, H-5 4-$NO_2C_6H_4$)	122.1* (C-3 C-5 4-$NO_2C_6H_4$)	122.1* (C-3 C-5 4-$NO_2C_6H_4$), 144.9 (C-4 4-$NO_2C_6H_4$), 152.5 (C–1 4-$NO_2C_6H_4$)
9.68 (br s, 1H, HN^+)	–	–
10.92 (s, 1H, C(S)NH)	–	123.0* (C-2, C-6 Ph)
11.97 (s, 1H, C(S)NH)	–	123.3* (C-2, C-6 Ph)

Figure S13. ^{1}H NMR spectrum of a mixture of Michael adduct 15a and dithiolopyridine 17a, DMSO-d_6 (400 MHz) (Table 1, entry 5)

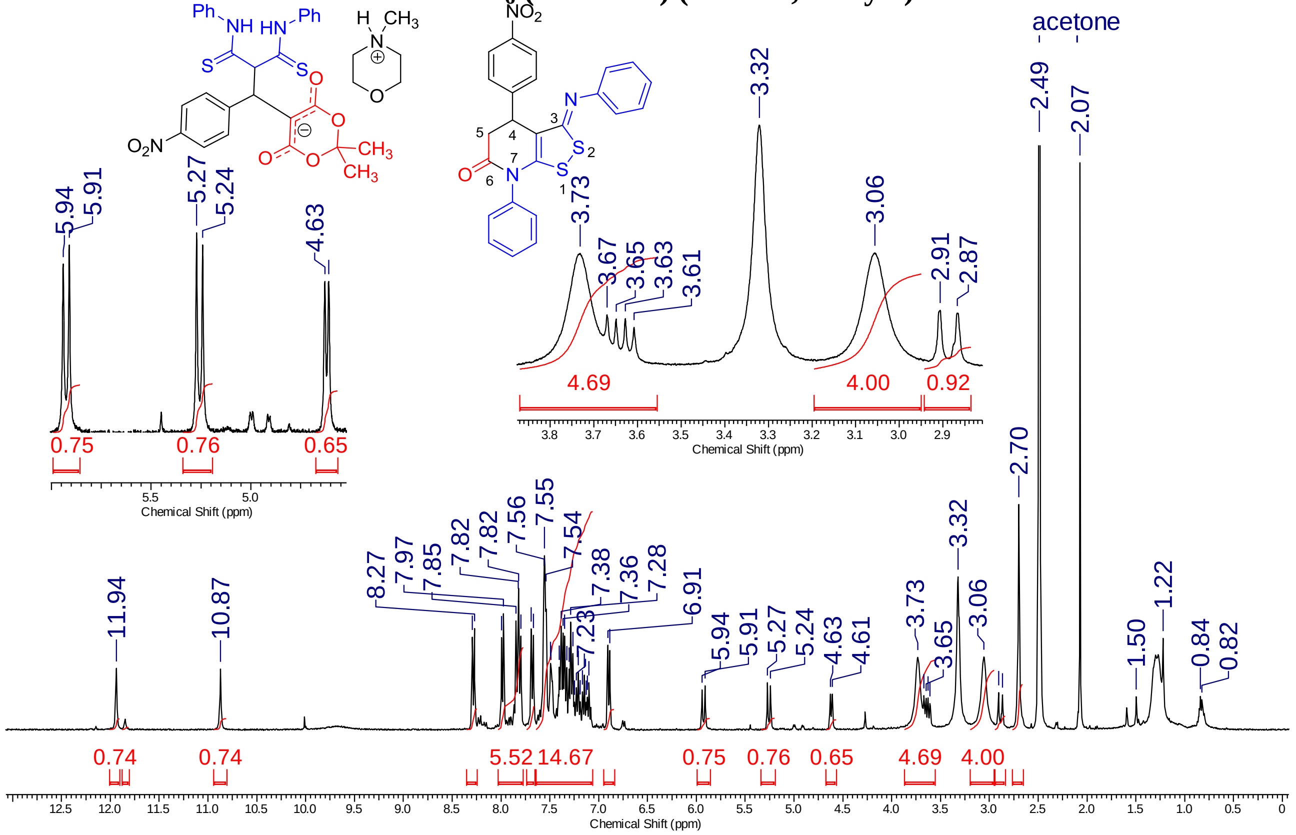

Figure S14. ^{13}C DEPTQ NMR spectrum of a mixture of Michael adduct 15a and dithiolopyridine 17a, DMSO-d_6 (101 MHz) (Table 1, entry 5)

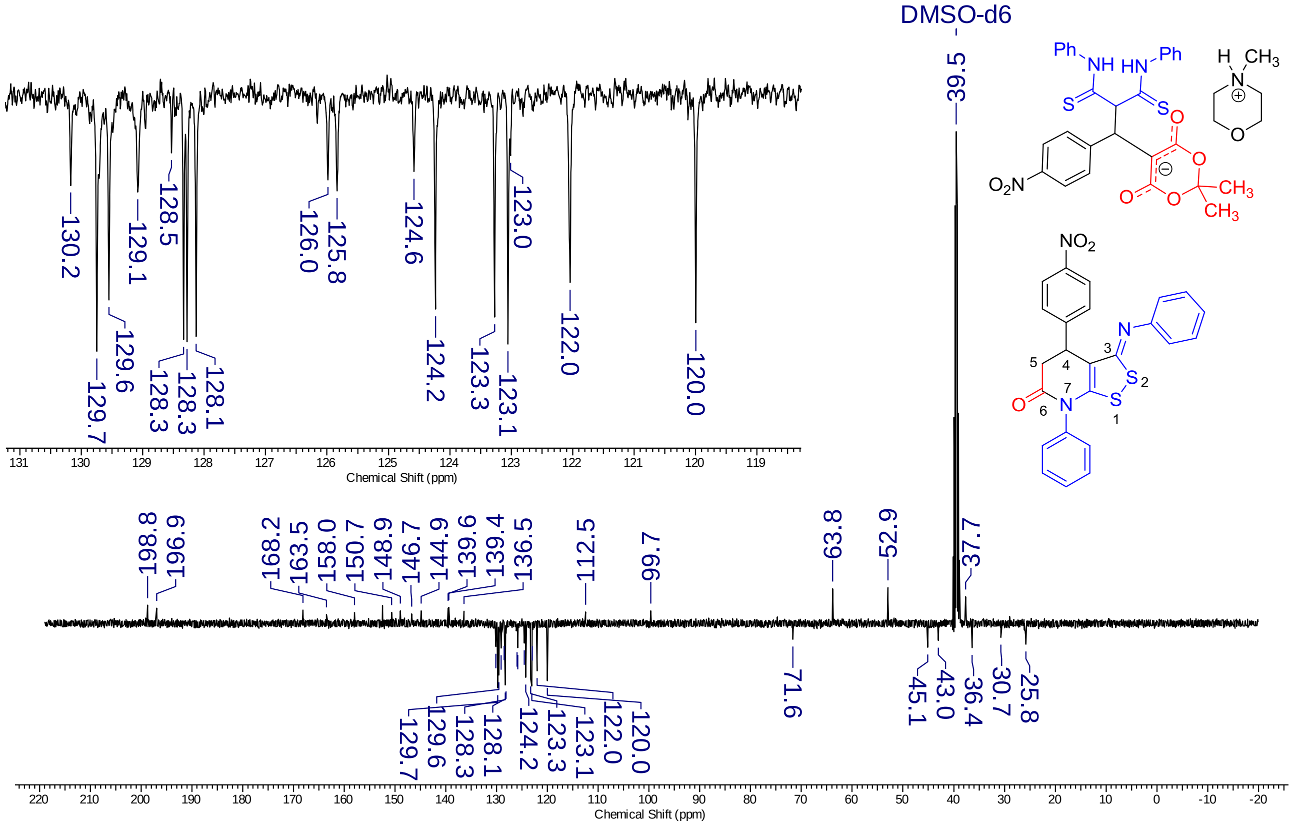

Figure S15. Comparison of ^{13}C DEPTQ NMR spectrum of pure 15a (Table 1, entry 4) with the spectrum of a mixture of Michael adduct 15a and dithiolopyridine 17a, DMSO-d_6 (101 MHz) (Table 1, entry 5)

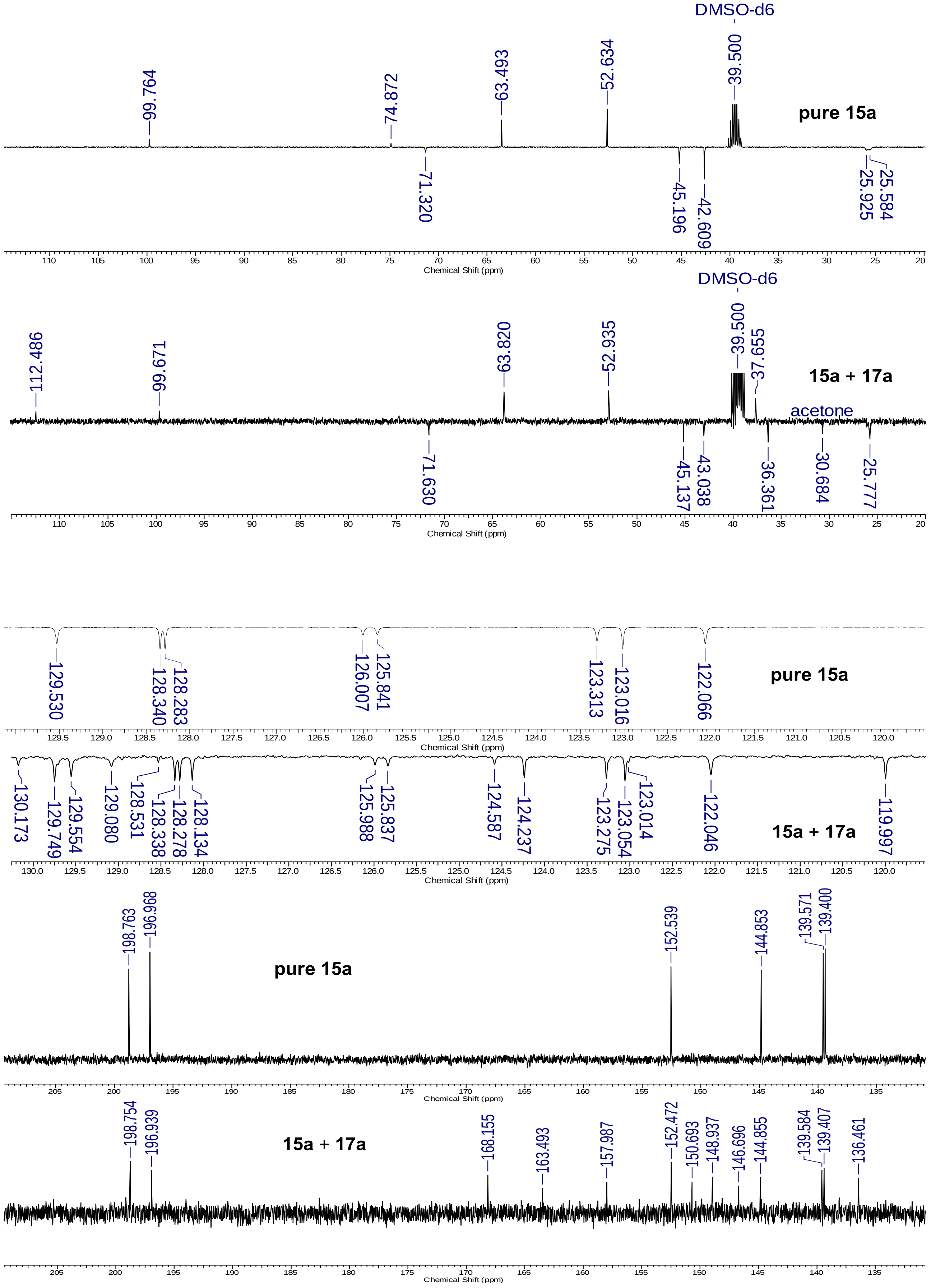

Figure S16. ^{1}H–^{13}C **HSQC NMR spectrum of a mixture of Michael adduct 15a and dithiolopyridine 17a, DMSO-d_6 (400/101 MHz) (Table 1, entry 5)**

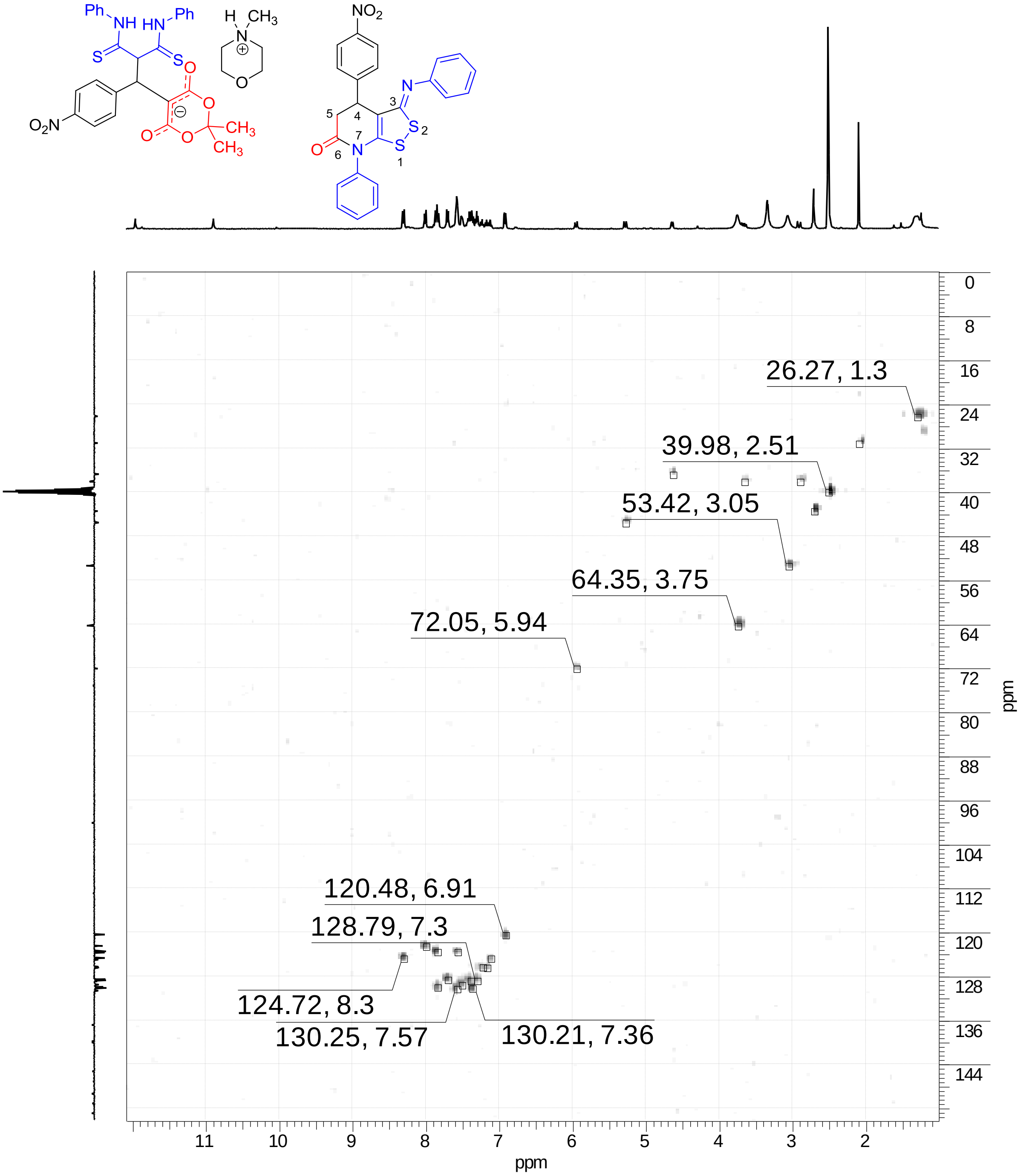

Figure S17. ^{1}H–^{13}C **HSQC NMR spectrum of a mixture of Michael adduct 15a and dithiolopyridine 17a, DMSO-d$_6$ (400/101 MHz) (Table 1, entry 5) (*fragment*)**

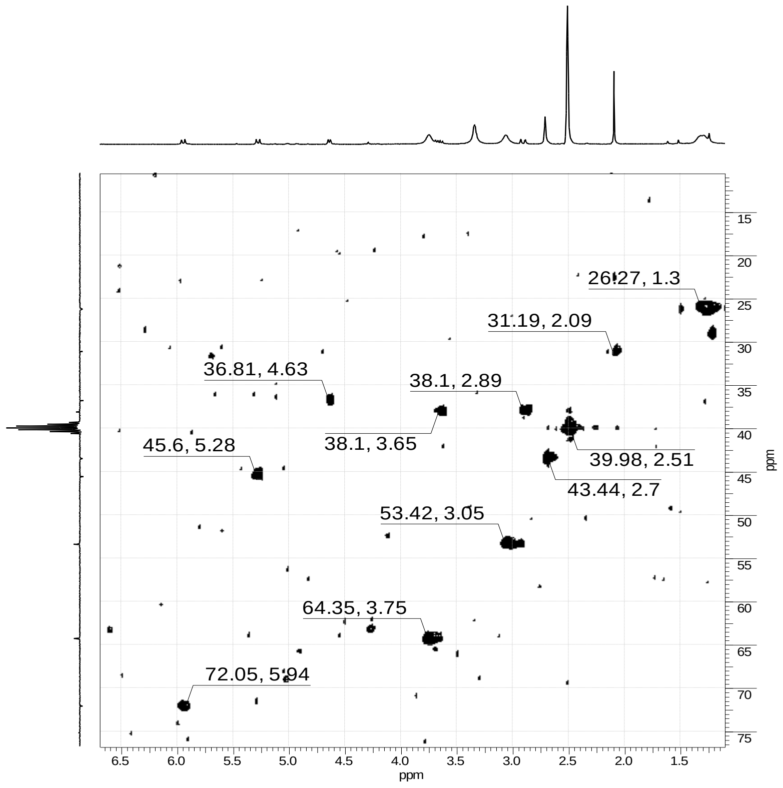

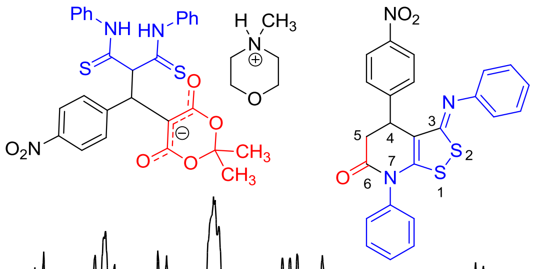

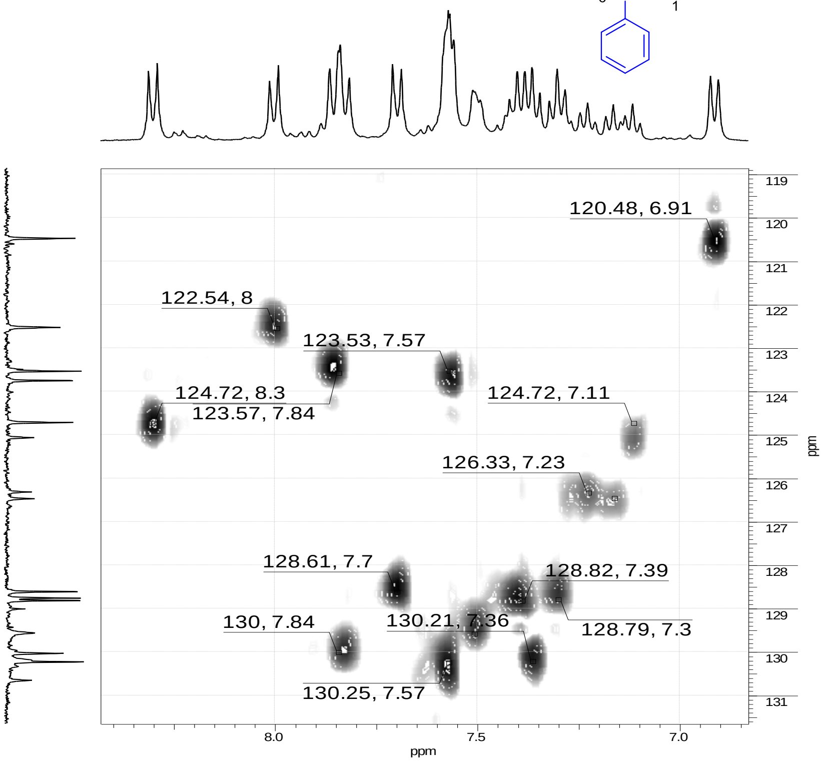

Figure S18. ^{1}H–^{13}C HMBC NMR spectrum of a mixture of Michael adduct 15a and dithiolopyridine 17a, DMSO-d_6 (400/101 MHz) (Table 1, entry 5)

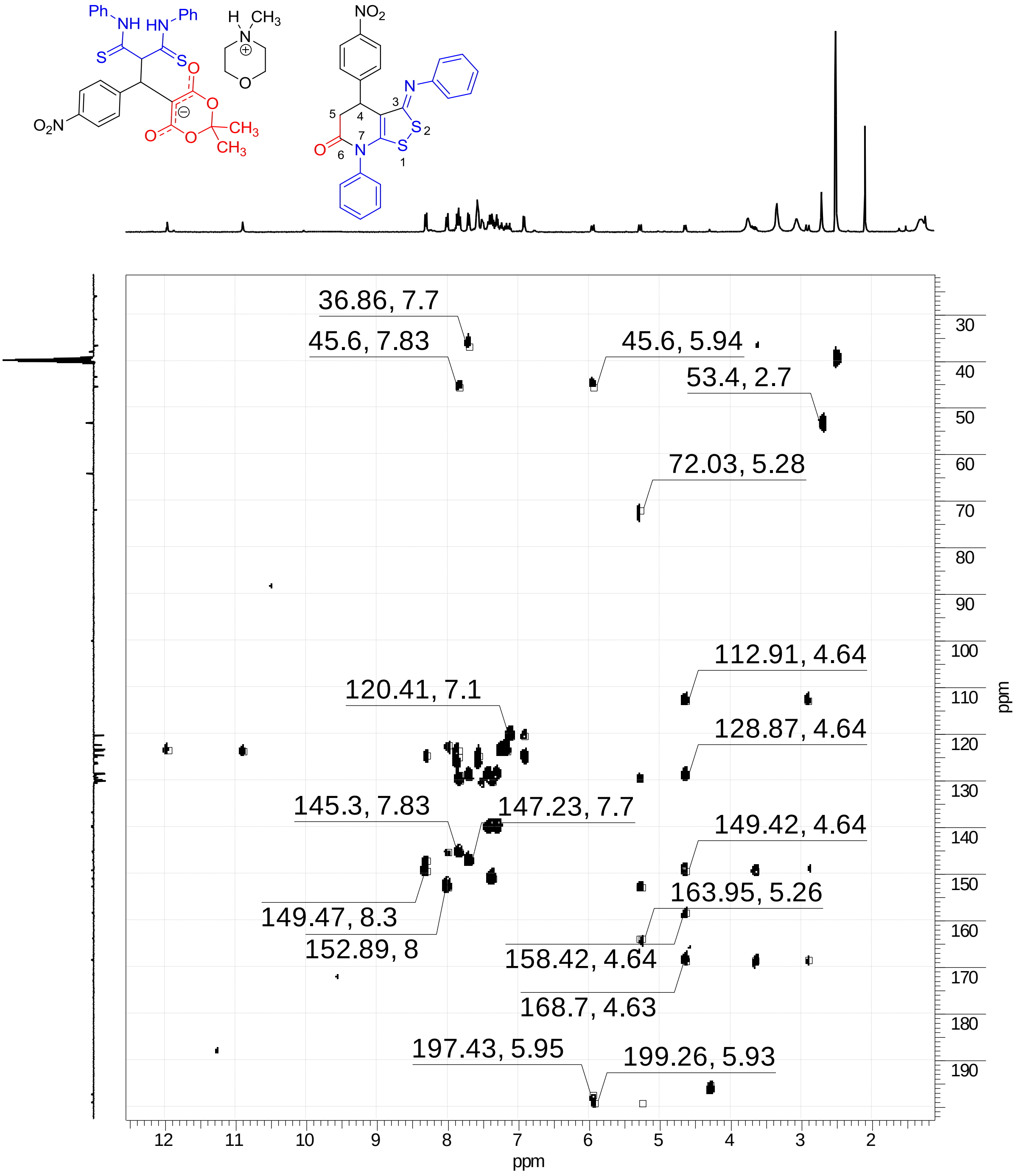

Figure S19. ^{1}H–^{13}C **HMBC NMR spectrum of a mixture of Michael adduct 15a and dithiolopyridine 17a, DMSO-d$_6$ (400/101 MHz) (Table 1, entry 5) (*fragment*)**

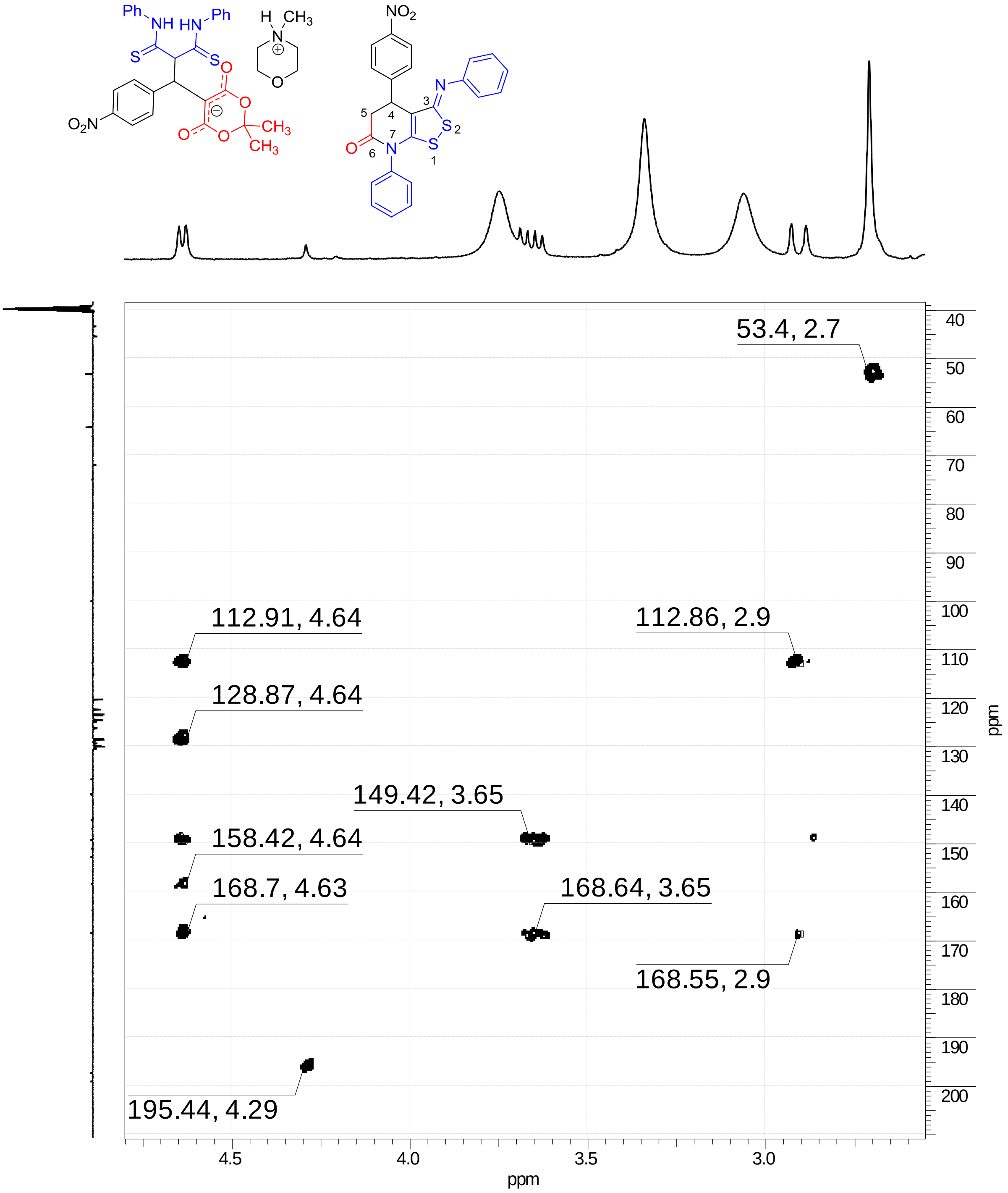

Figure S20. ^{1}H–^{13}C **HMBC NMR spectrum of a mixture of Michael adduct 15a and dithiolopyridine 17a, DMSO-d$_6$ (400/101 MHz) (Table 1, entry 5) (*fragment*)**

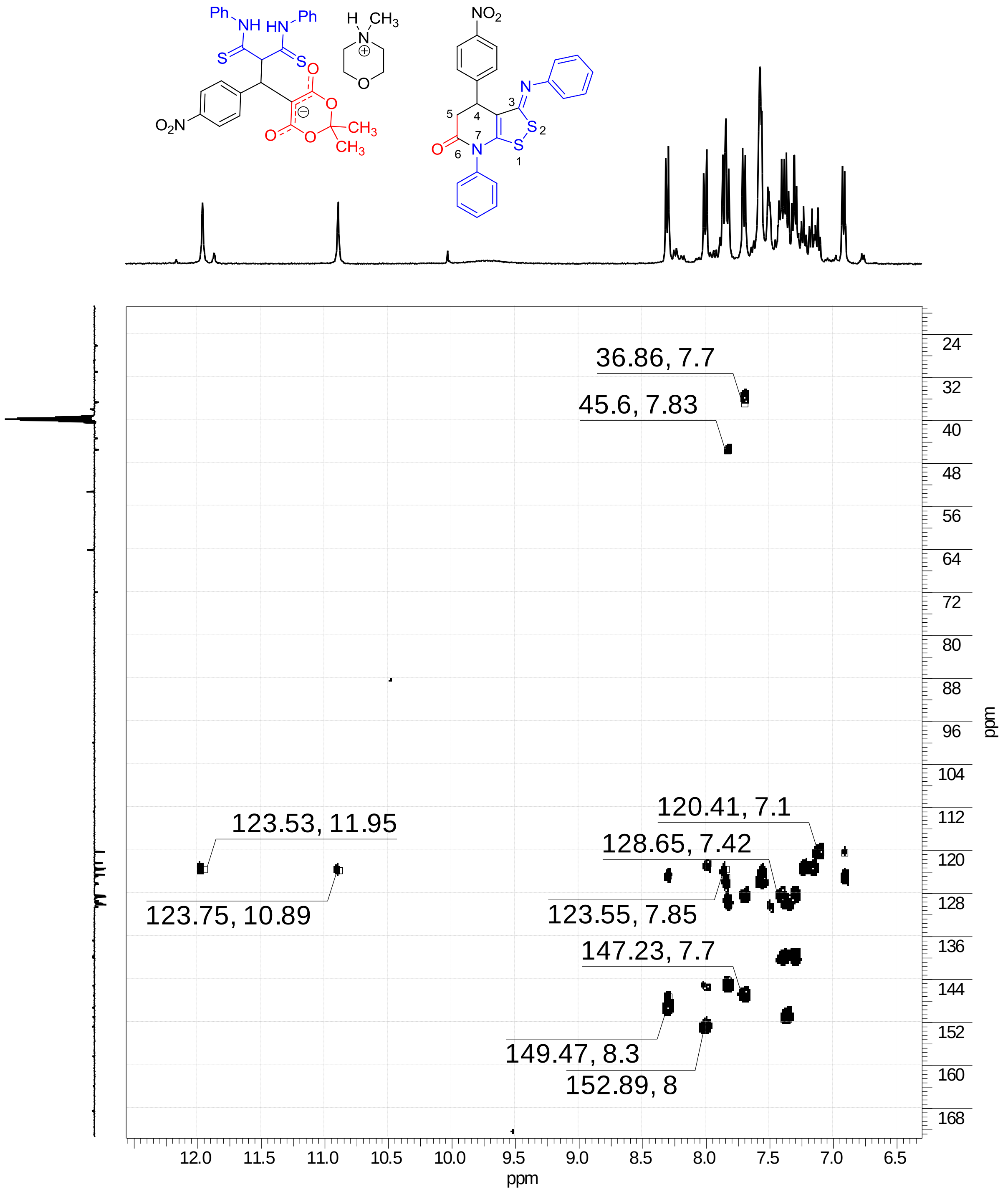

Figure S21. ^{1}H–^{13}C HMBC NMR spectrum of a mixture of Michael adduct 15a and dithiolopyridine 17a, DMSO-d_6 (400/101 MHz) (Table 1, entry 5) (*fragment*)

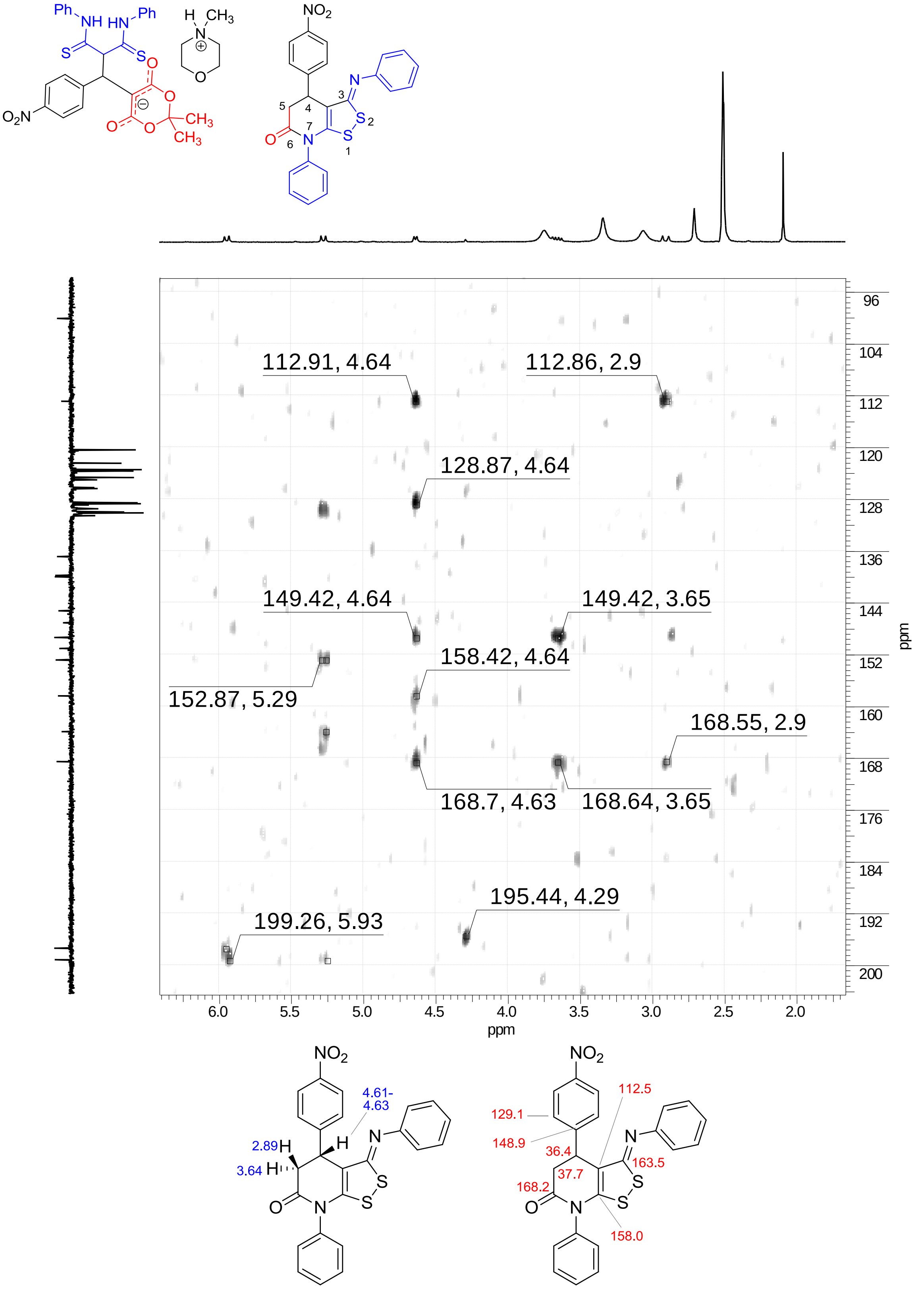

The assigned chemical shifts
based on the observed correlations

Figure S22. ^{1}**H NMR spectrum of the Michael adduct 15b, DMSO-d_6 (400 MHz) (Table 1, entry 6)**

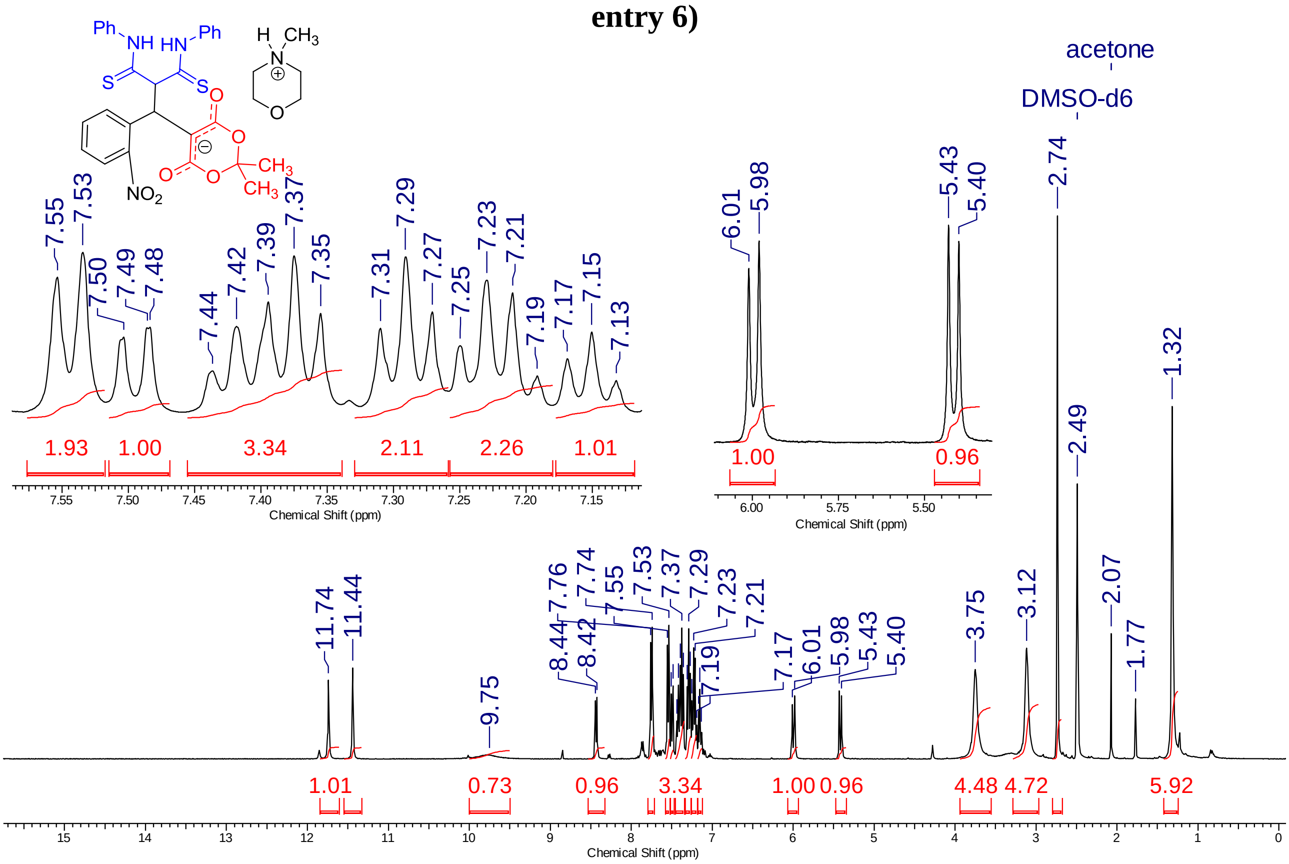

Figure S23. ^{13}**C DEPTQ NMR spectrum of the Michael adduct 15b, DMSO-d_6 (101 MHz) (Table 1, entry 6)**

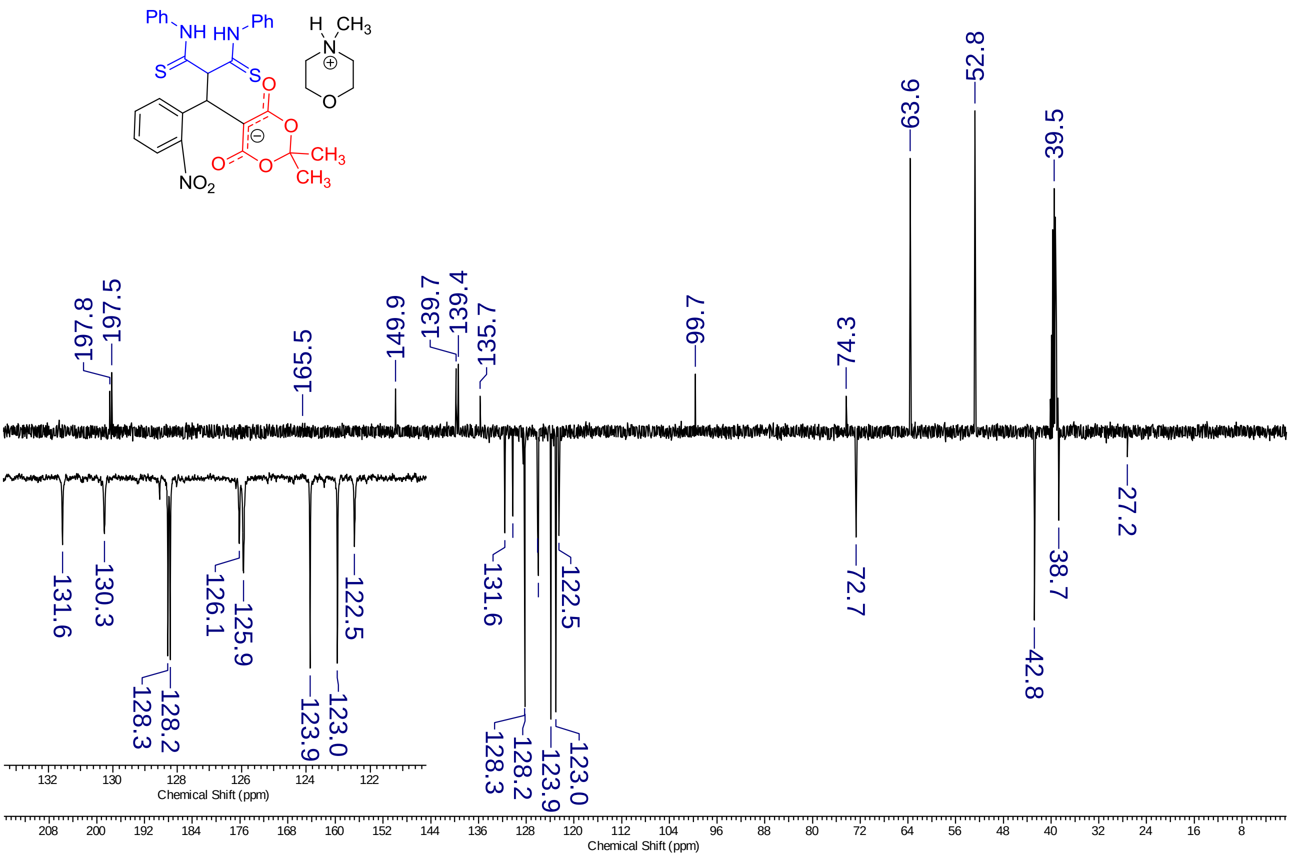

Figure S24. ^{1}H–^{13}C **HSQC NMR spectrum of the Michael adduct 15b, DMSO-d_6 (400/101 MHz) (Table 1, entry 6)**

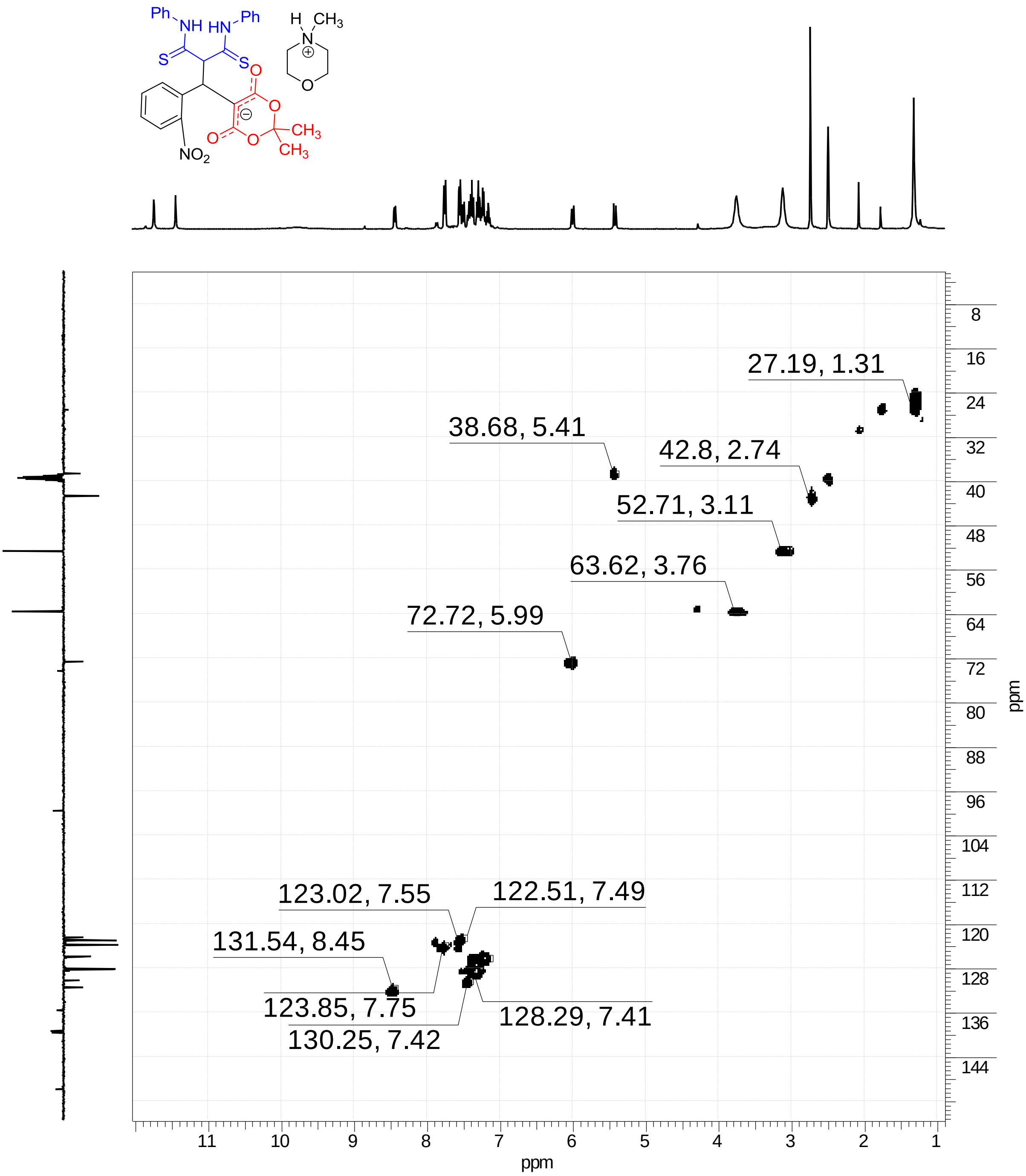

Figure S25. ^{1}H–^{13}C **HSQC NMR spectrum of the Michael adduct 15b, DMSO-d$_6$ (400/101 MHz) (Table 1, entry 6) (*fragments*)**

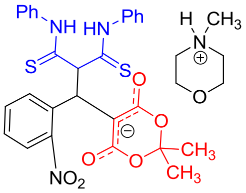

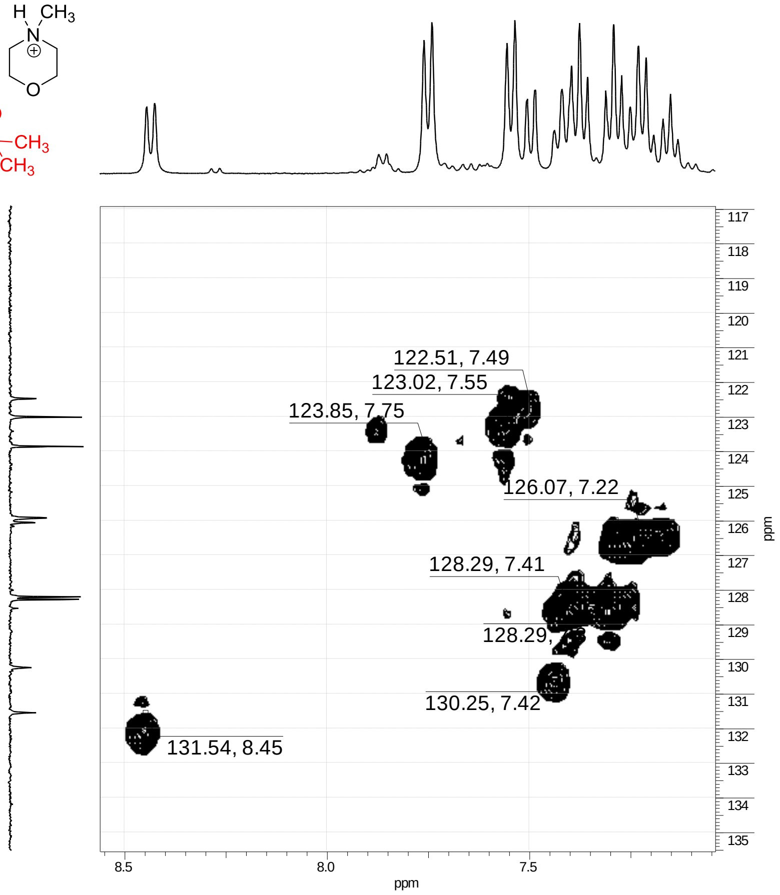

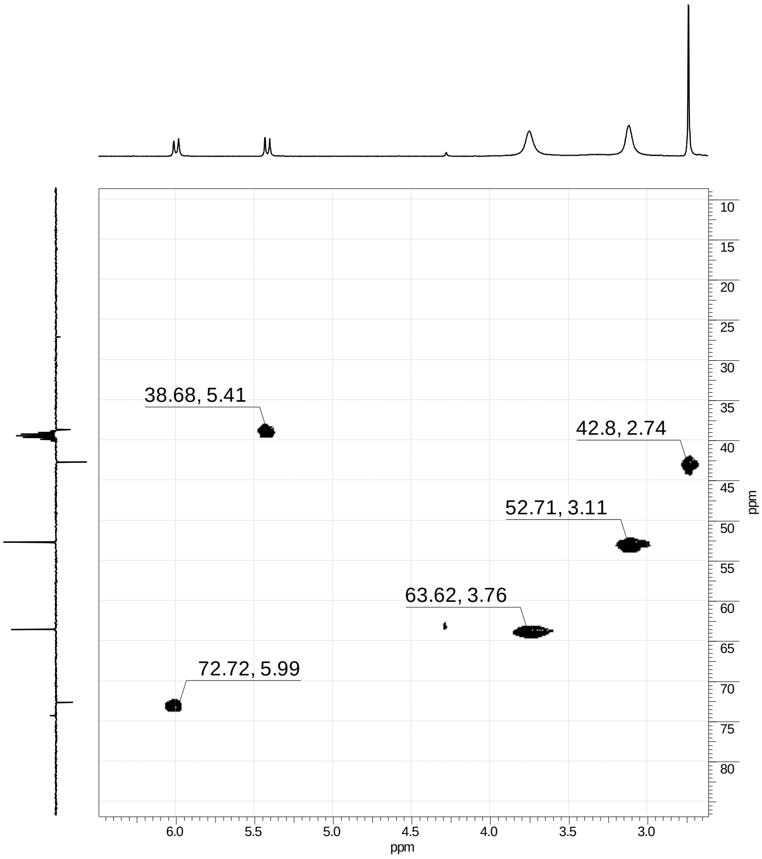

Figure S26. ^{1}H–^{13}C **HMBC NMR spectrum of the Michael adduct 15b, DMSO-d$_6$ (400/101 MHz) (Table 1, entry 6)**

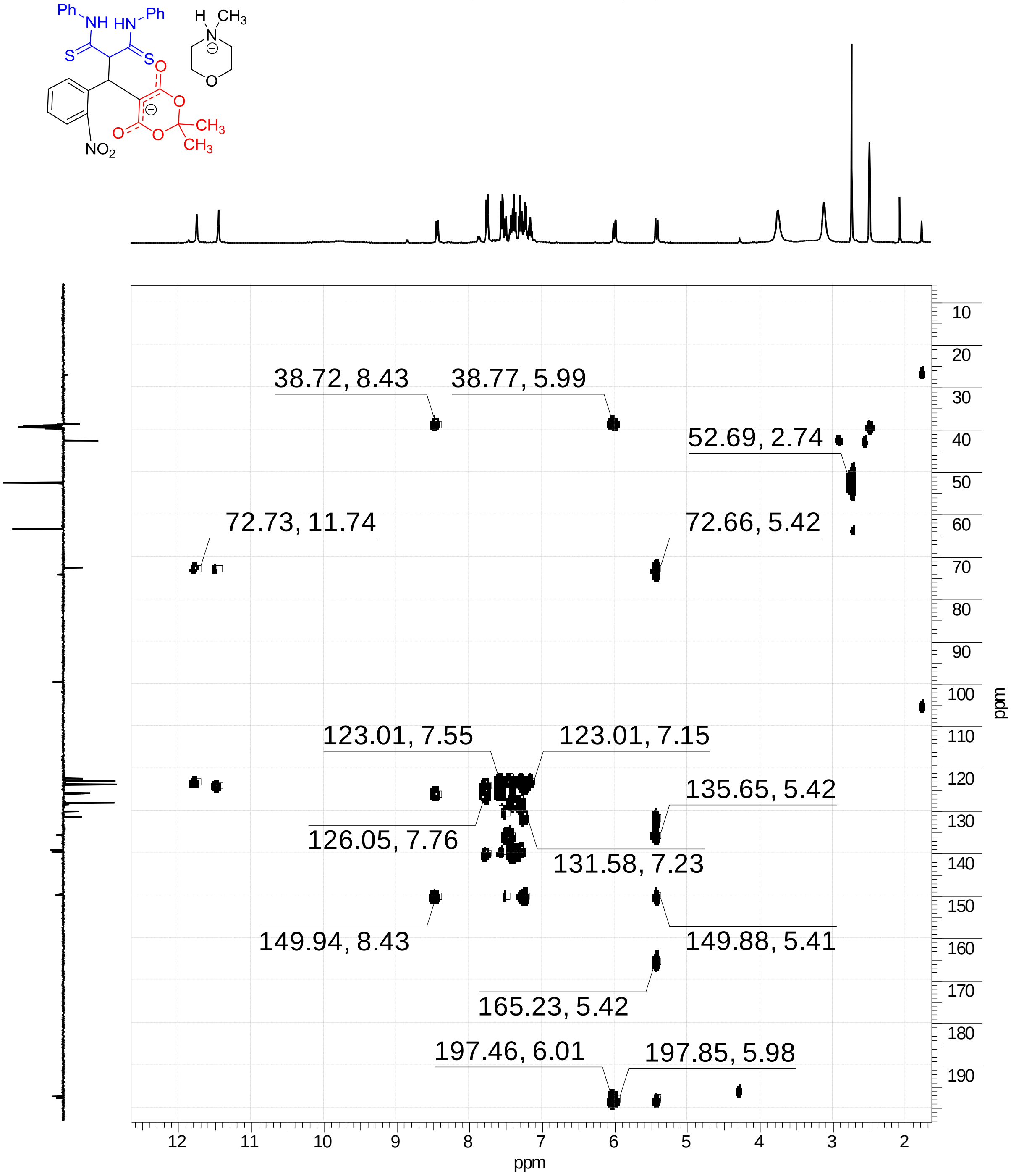

Figure S27. ^{1}H–^{13}C **HMBC NMR spectrum of the Michael adduct 15b, DMSO-d$_6$ (400/101 MHz) (Table 1, entry 6) (*fragments*)**

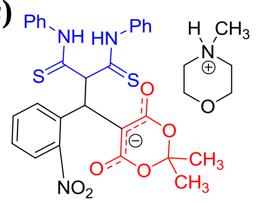

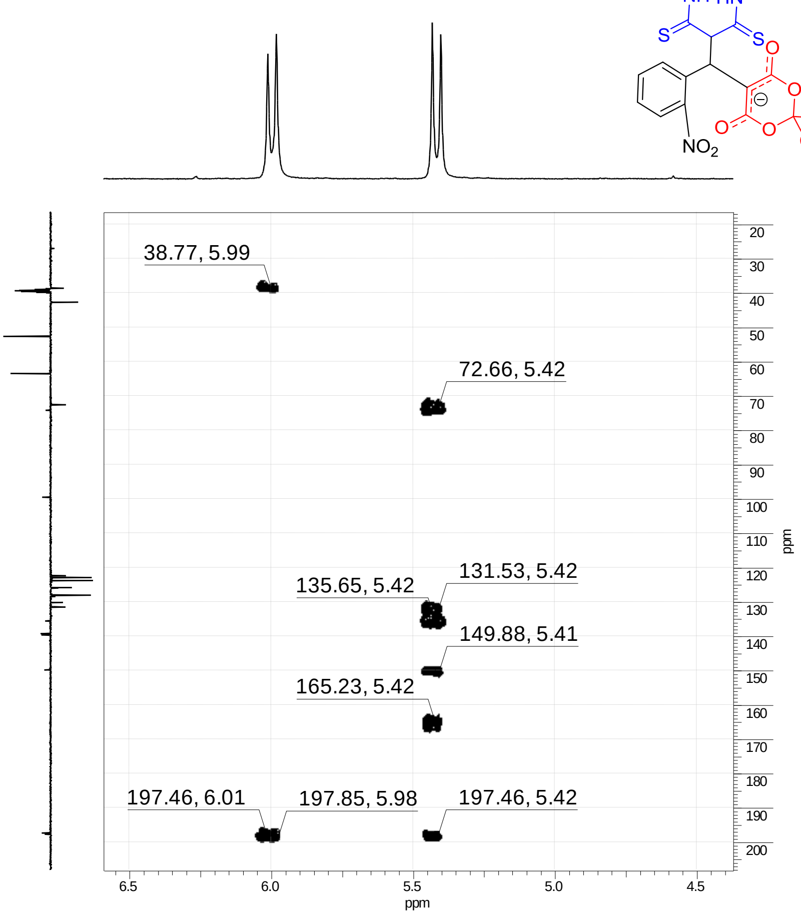

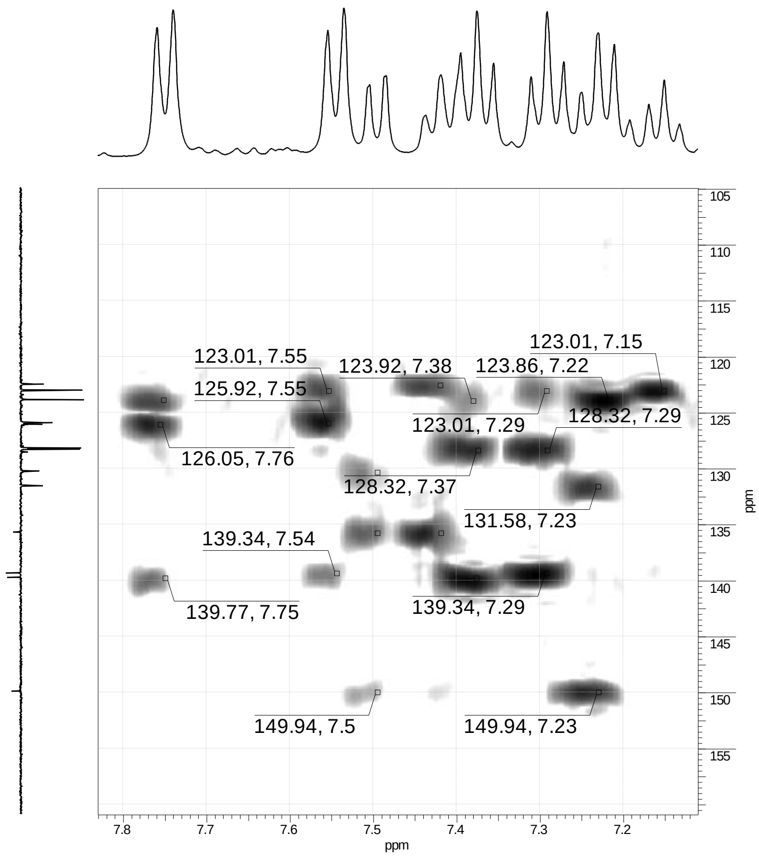

Table S2. The observed correlations in the ^1H-^{13}C HSQC and ^1H-^{13}C HMBC 2D NMR spectra of the Michael adduct 15b (Table 1, entry 6)

^{13}C chemical shifts are given in **red**, ^1H shifts – in **blue**

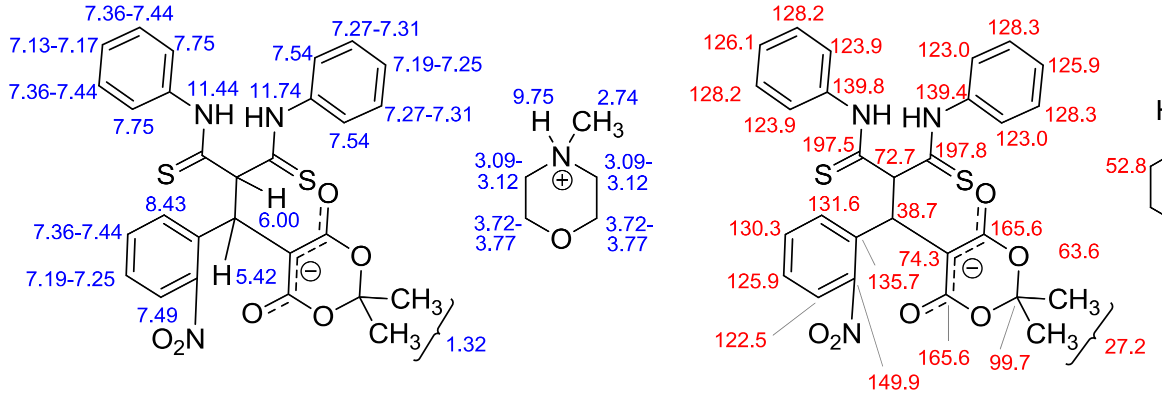

^1H NMR shifts, δ, ppm	Correlations in HSQC spectrum, δ, ppm	Correlations in HMBC spectrum, δ, ppm
1.32 (br s, 6H, Me)	27.2* (2 CH_3)	99.7 (O–CMe$_2$–O)
2.74 (s, 3H, NMe)	42.8* (N–CH_3)	52.8 (CH_2NCH_2)
3.09-3.12 (m, 4H, CH_2NCH_2)	52.8 (CH_2NCH_2)	–
3.72-3.77 (m, 4H, CH_2OCH_2)	63.6 (CH_2OCH_2)	–
5.42 (d, ^3J = 12.0 Hz, 1H, CH-Ar)	38.7* (CH–Ar)	72.7* (CH–CSNHPh), 74.3 (C–C=O), 131.6* (C-6 2-$NO_2C_6H_4$), 135.7 (C-1 2-$NO_2C_6H_4$), 149.9 (C-2 2-$NO_2C_6H_4$), 197.5 (C=S), 197.8 (C=S)
6.00 (d, ^3J = 12.0 Hz, 1H, CH-CSNHPh)	72.7* (CH–CSNHPh)	38.7* (CH–Ar), 197.5 (C=S), 197.8 (C=S)
7.13-7.17 (m, 1H, H-4 Ph)	125.9* (2C, C-4 Ph and C-4 2-$NO_2C_6H_4$ overlapped)	123.0* (C-2, C-6 Ph)
7.19-7.25 (m, 1H, H-4 Ph and H-4 2-$NO_2C_6H_4$ overlapped)	125.9* (2C, C-4 Ph and C-4 2-$NO_2C_6H_4$ overlapped), 126.1* (C-4 Ph)	123.9* (C-2, C-6 Ph), 131.6* (C-6 2-$NO_2C_6H_4$), 149.9 (C-2 2-$NO_2C_6H_4$)
7.27-7.31 (m, 2H, H-3, H-5 Ph)	128.3* (C-3, C-5 Ph)	123.0* (C-2, C-6 Ph), 128.3* (C-3, C-5 Ph), 139.4 (C-1 Ph)
7.36-7.44 (m, 3H, H-3, H-5 Ph and H-5 2-$NO_2C_6H_4$ overlapped)	128.2* (C-3, C-5 Ph), 130.3* (C-5 2-$NO_2C_6H_4$)	122.5* (C-3 2-$NO_2C_6H_4$), 123.9* (C-2, C-6 Ph), 128.2* (C-3, C-5 Ph), 135.7 (C-1 2-$NO_2C_6H_4$), 139.8 (C-1 Ph)
7.49 (dd, ^3J = 8.0 Hz, ^4J = 0.9 Hz, 1H, H-3 2-$NO_2C_6H_4$)	122.5* (C-3 2-$NO_2C_6H_4$)	130.3* (C-5 2-$NO_2C_6H_4$), 135.7 (C-1 2-$NO_2C_6H_4$), 149.9 (C-2 2-$NO_2C_6H_4$)
7.54 (d, ^3J = 7.6 Hz, 2H, H-2, H-6 Ph)	123.0* (C-2, C-6 Ph)	123.0* (C-2, C-6 Ph), 125.9* (2C, C-4 Ph and C-4 2-$NO_2C_6H_4$ overlapped), 139.4 (C-1 Ph)
7.75 (d, ^3J = 7.7 Hz, 2H, H-2, H-6 Ph)	123.9* (C-2, C-6 Ph)	123.9* (C-2, C-6 Ph), 126.1* (C-4 Ph), 139.8 (C-1 Ph)
8.43 (d, ^3J = 7.7 Hz, 1H, H-6 2-$NO_2C_6H_4$)	131.6* (C-6 2-$NO_2C_6H_4$)	38.7* (CH–Ar), 125.9* (2C, C-4 Ph and C-4 2-$NO_2C_6H_4$ overlapped), 149.9 (C-2 2-$NO_2C_6H_4$)
9.75 (br s, 1H, HN^+)	–	–
11.44 (s, 1H, C(S)NH)	–	72.7* (CH–CSNHPh), 123.9* (C-2, C-6 Ph)
11.74 (s, 1H, C(S)NH)	–	72.7* (CH–CSNHPh), 123.0* (C-2, C-6 Ph)

*Signals with a negative phase.

Figure S28. ^{1}H **NMR spectrum of a mixture of Michael adduct 15b and thiolate 16b, DMSO-d_6 (400 MHz) (Table 1, entry 7)**

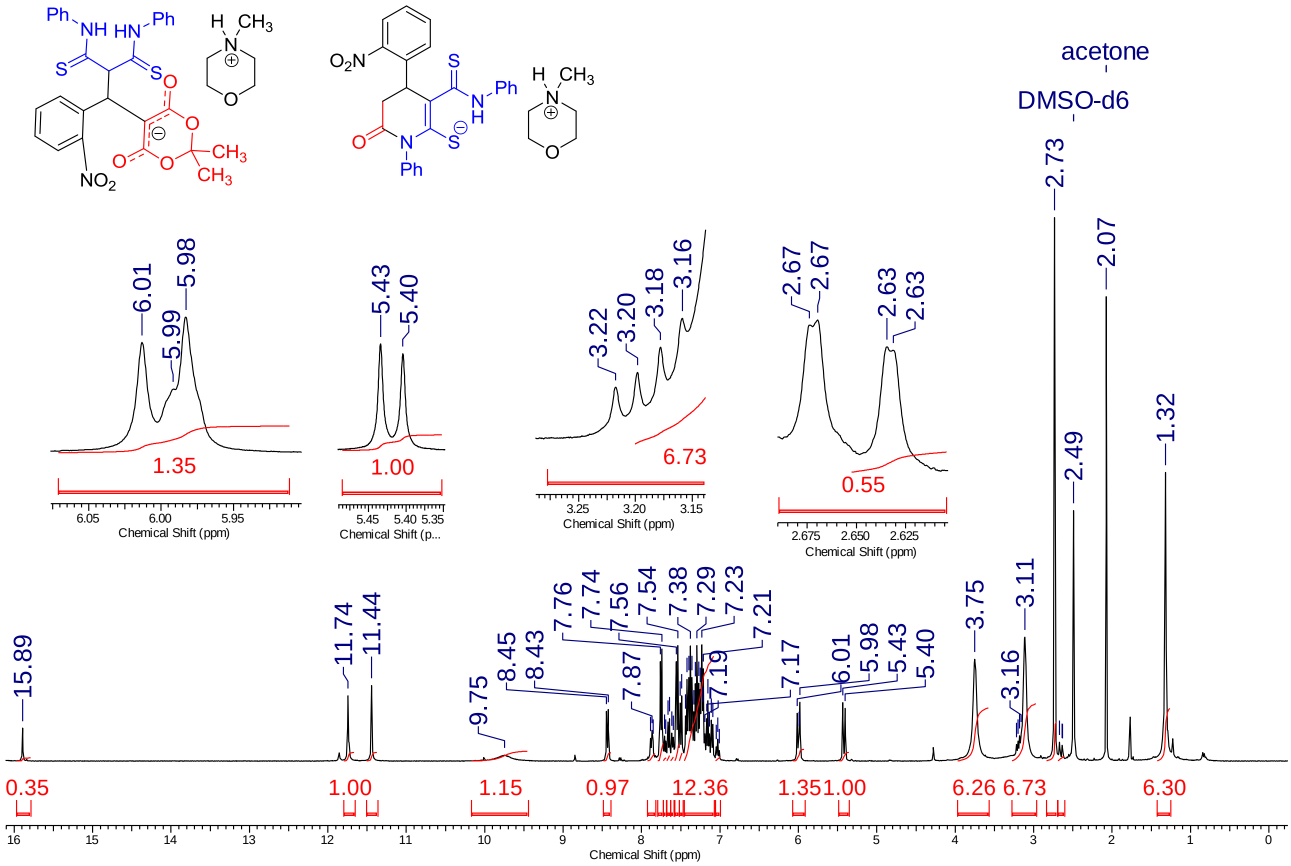

Figure S29. ^{13}C **DEPTQ NMR spectrum of a mixture of Michael adduct 15b and thiolate 16b, DMSO-d_6 (101 MHz) (Table 1, entry 7)**

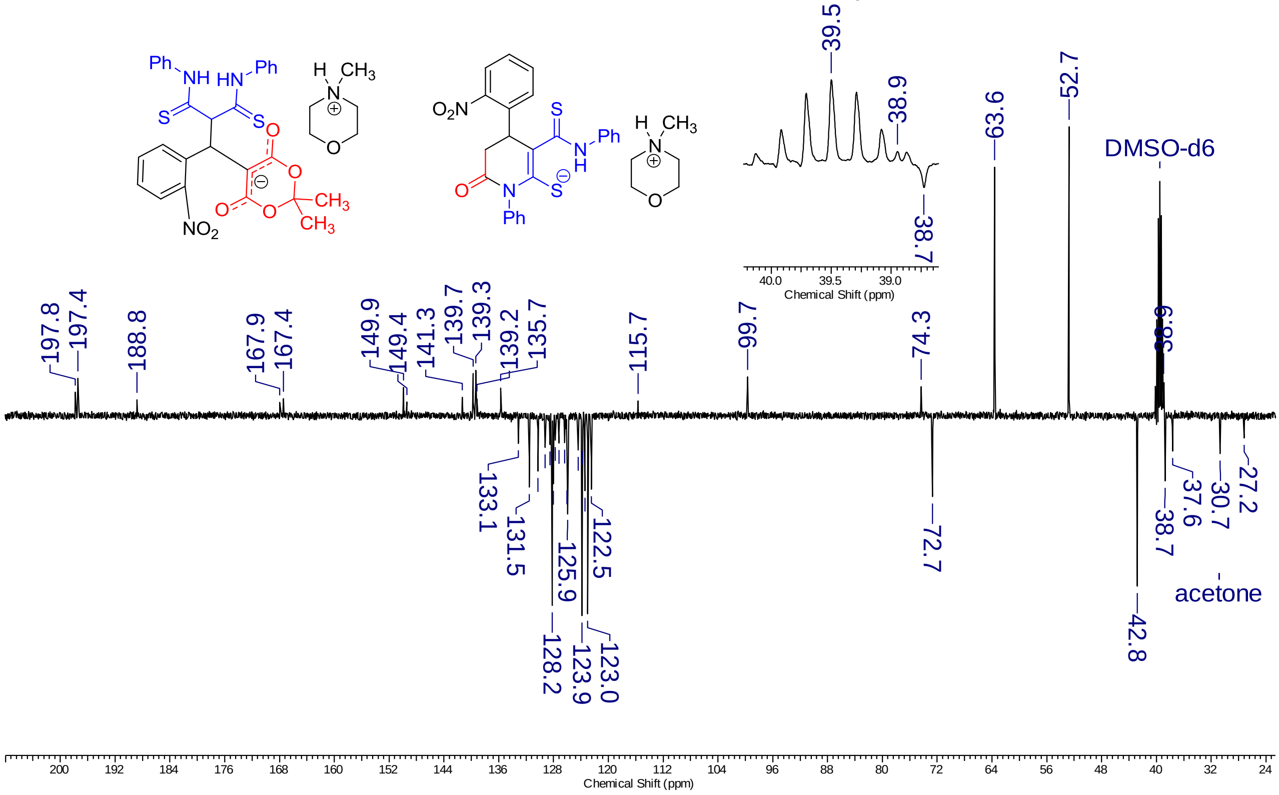

Figure S30. Comparison of ^{13}C DEPTQ NMR spectrum of pure 15b (Table 1, entry 4) with the spectrum of a mixture of Michael adduct 15b and pyridine-2-thiolate 16b, DMSO-d_6 (101 MHz) (Table 1, entry 7)

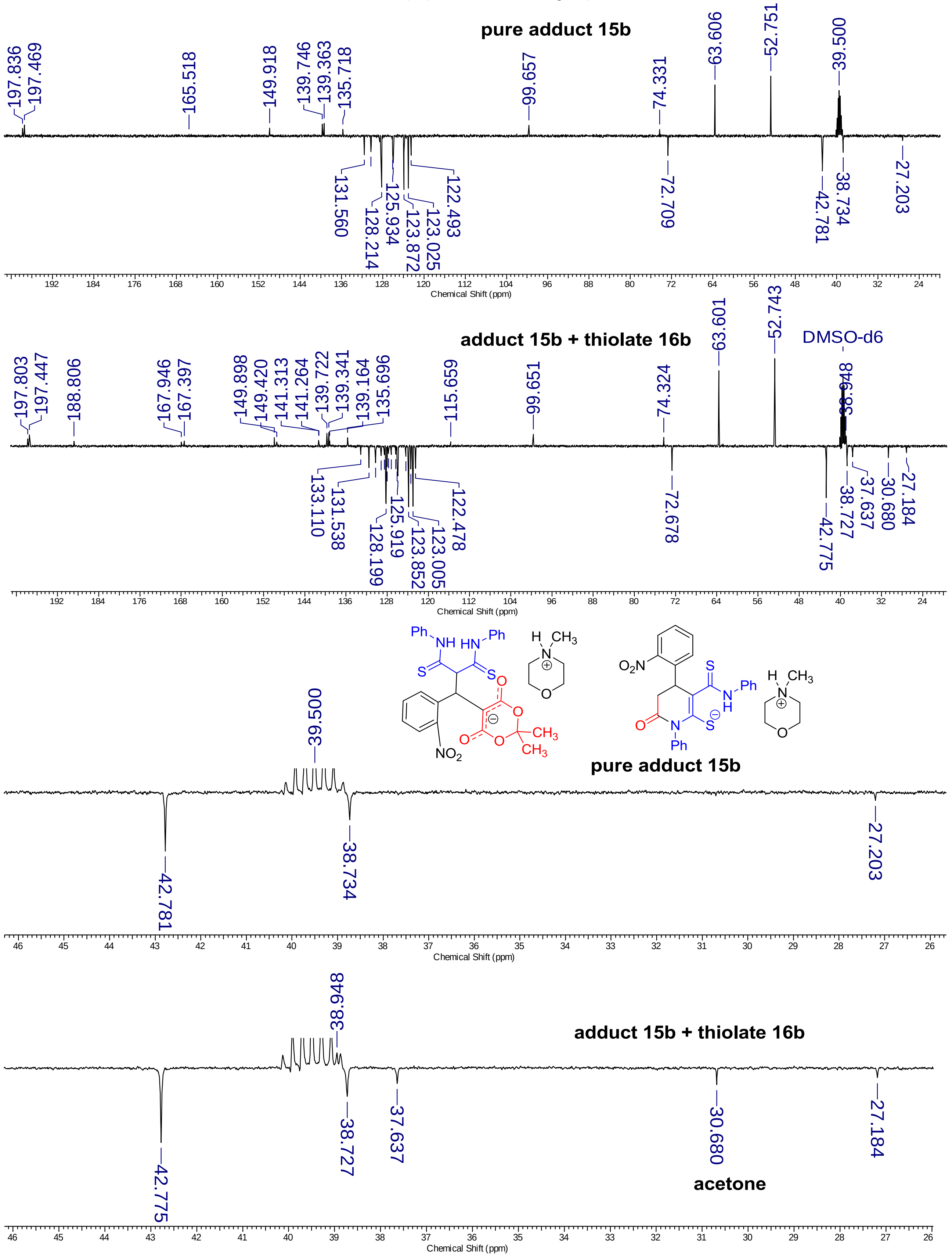

Figure S31. Comparison of ^{13}C DEPTQ NMR spectrum of pure 15b (Table 1, entry 4) with the spectrum of a mixture of Michael adduct 15b and pyridine-2-thiolate 16b, DMSO-d_6 (101 MHz) (Table 1, entry 7) (*continued*)

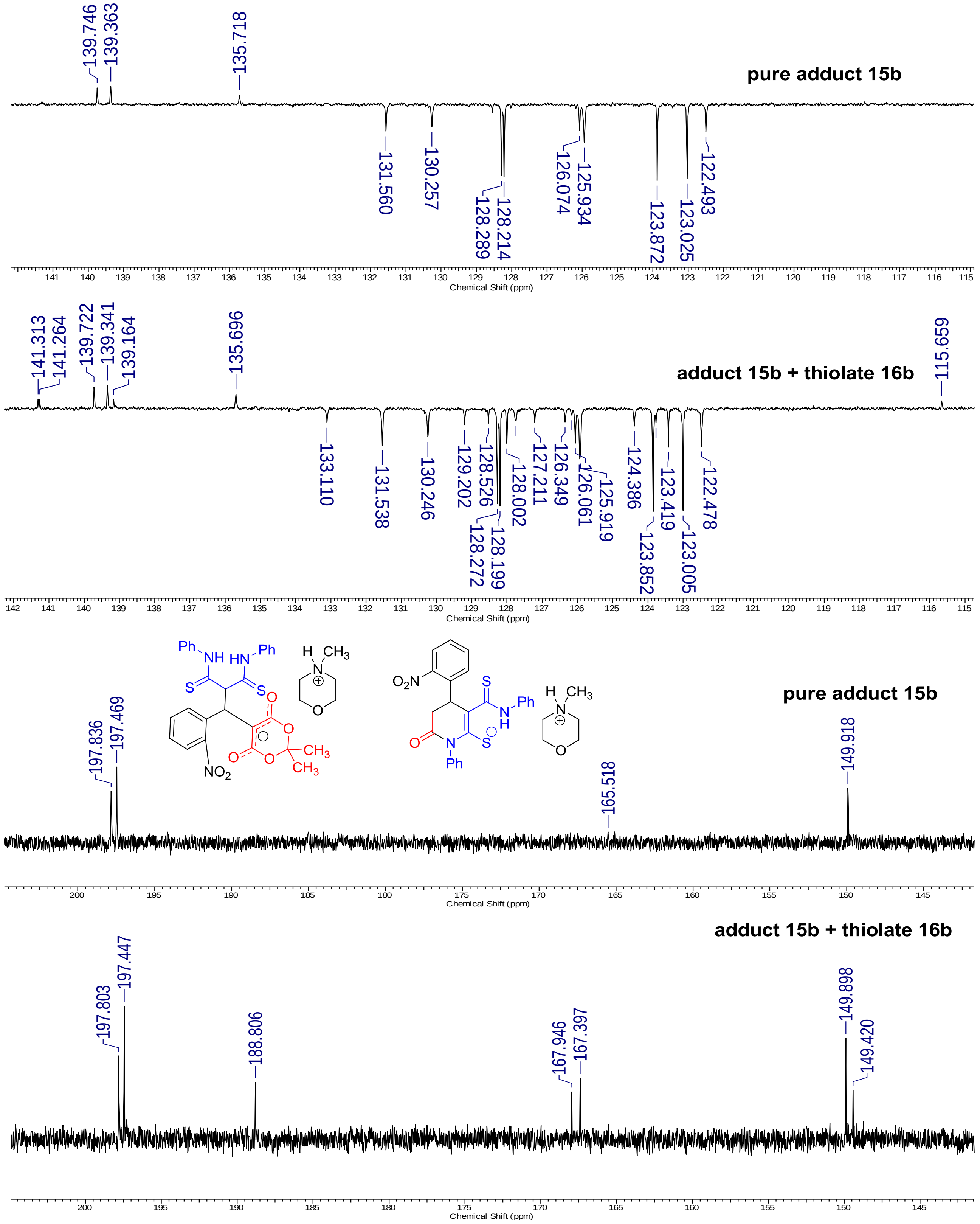

Figure S32. ^{1}H–^{13}C HSQC NMR spectrum of a mixture of Michael adduct 15b and pyridine-2-thiolate 16b, DMSO-d_6 (400/101 MHz) (Table 1, entry 7)

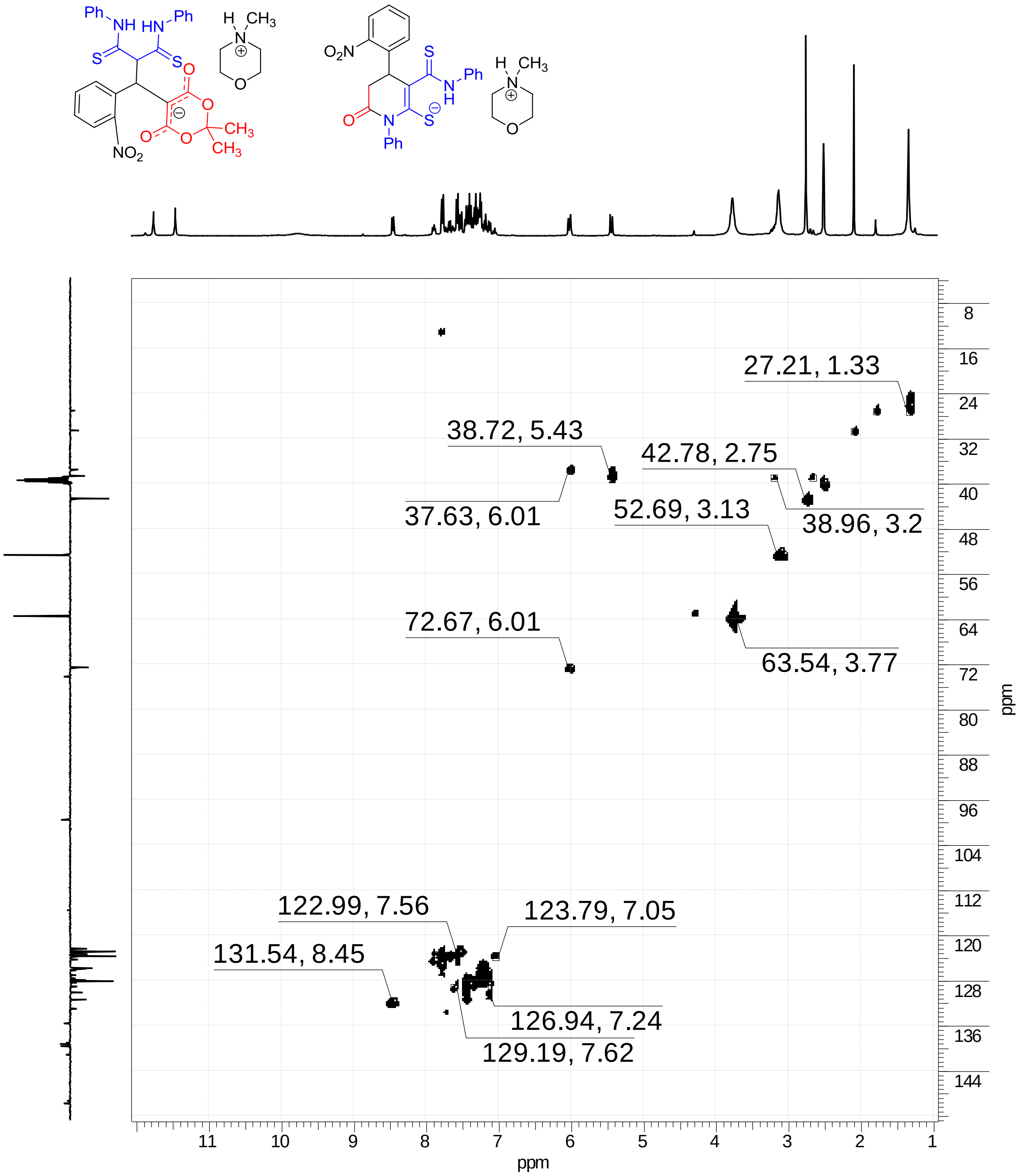

Figure S33. ^{1}H–^{13}C **HSQC NMR spectrum of a mixture of Michael adduct 15b and pyridine-2-thiolate 16b, DMSO-d$_6$ (400/101 MHz) (Table 1, entry 7) (*fragments*)**

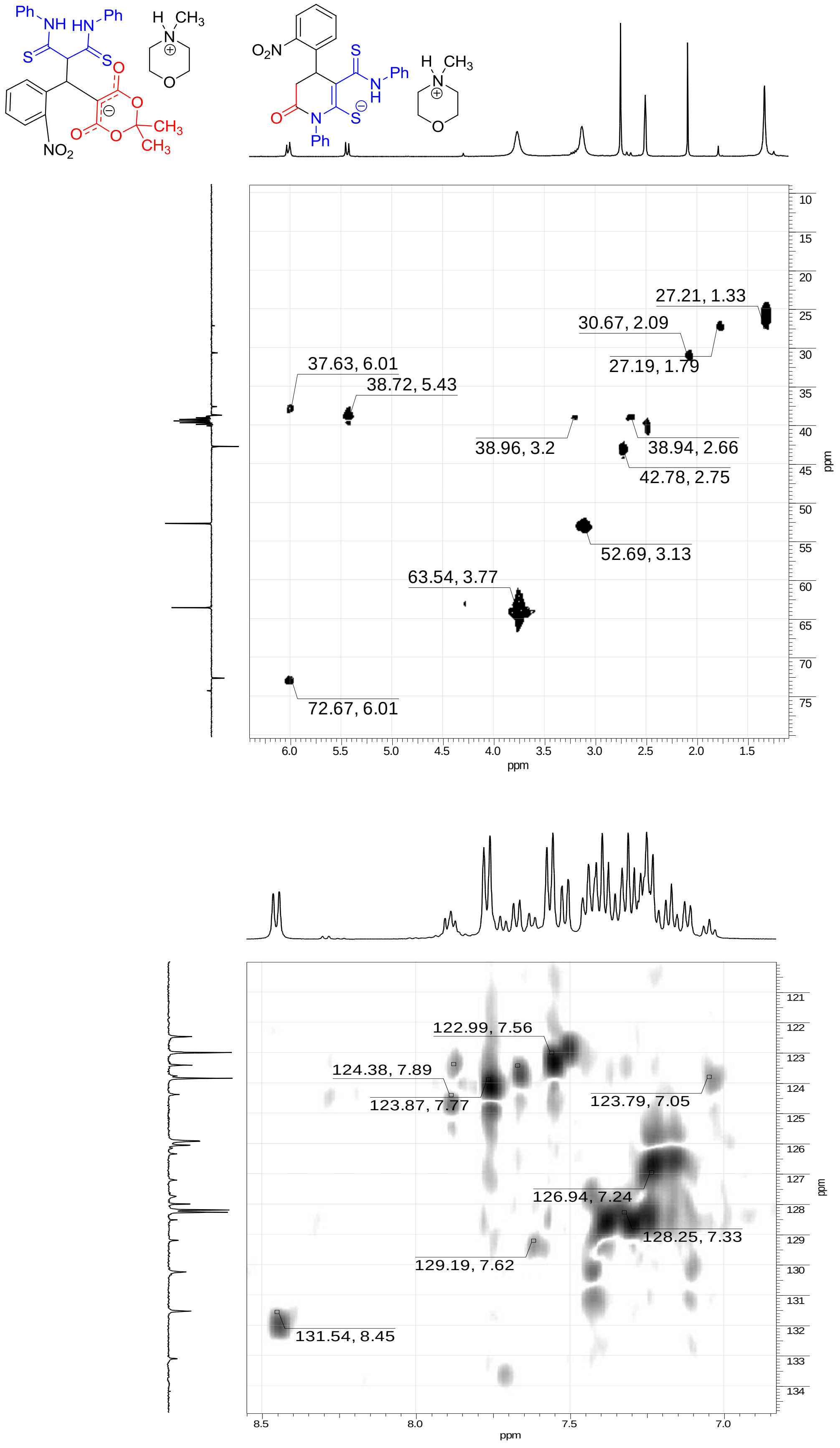

Figure S34. ^{1}H–^{13}C HMBC NMR spectrum of a mixture of Michael adduct 15b and pyridine-2-thiolate 16b, DMSO-d_6 (400/101 MHz) (Table 1, entry 7)

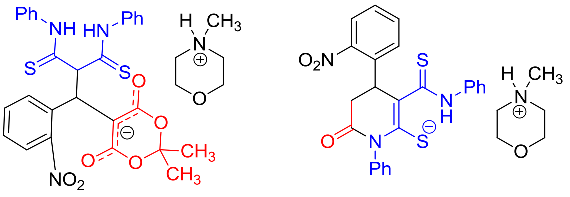

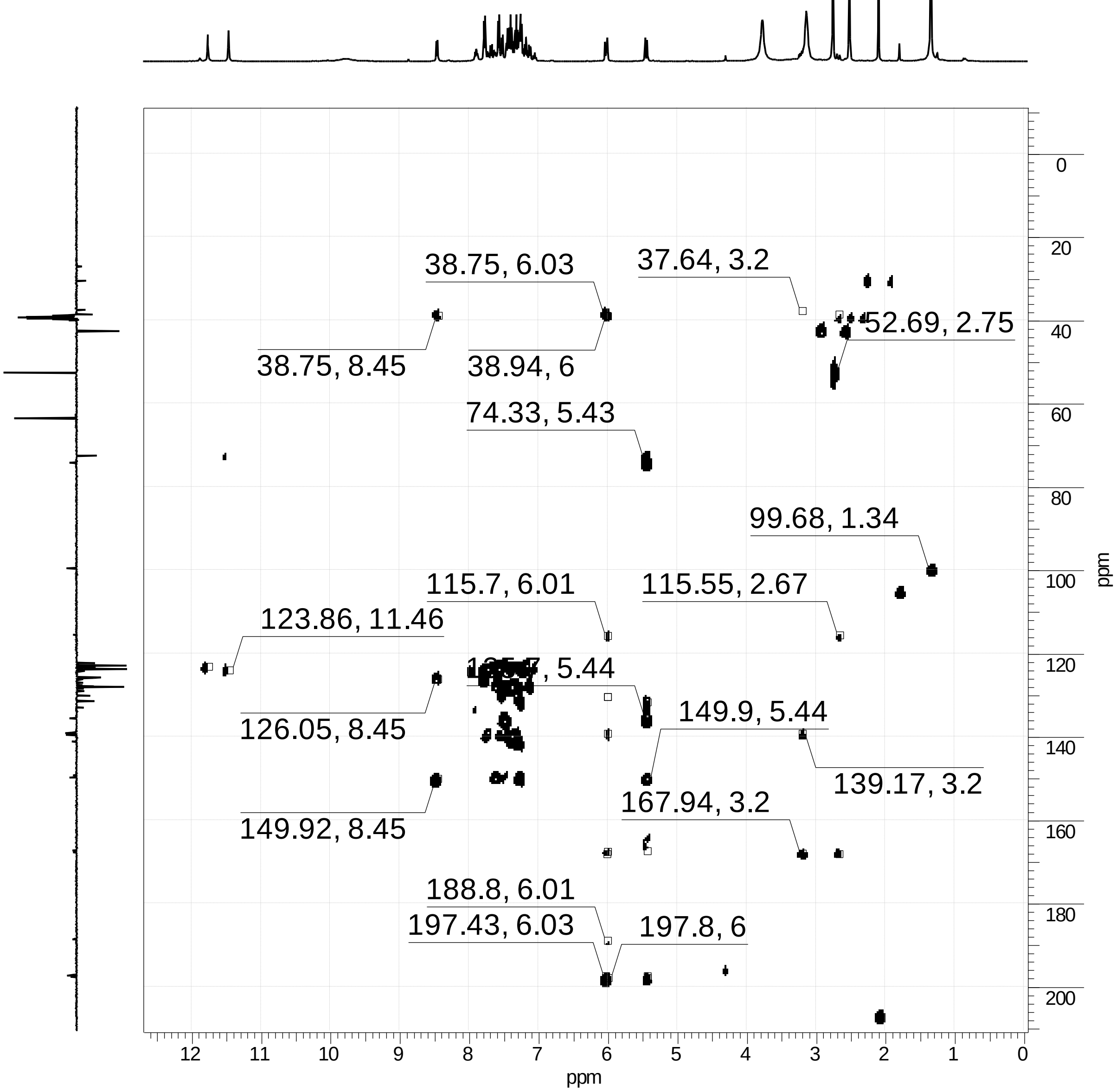

Figure S35. ^{1}H–^{13}C **HMBC NMR spectrum of a mixture of Michael adduct 15b and pyridine-2-thiolate 16b, DMSO-d$_6$ (400/101 MHz) (Table 1, entry 7) (*fragments*)**

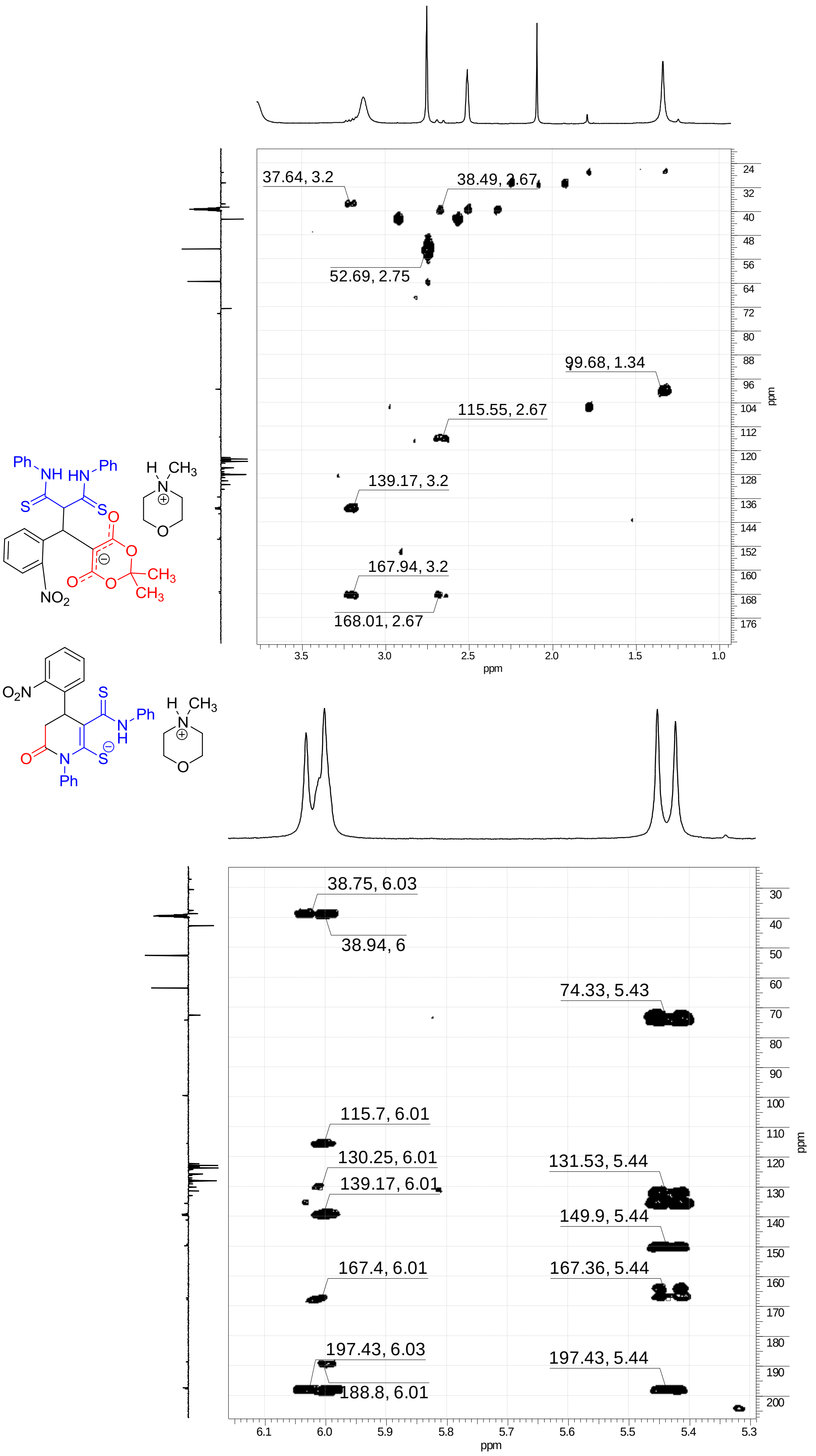

Figure S36. ^{1}H–^{13}C **HMBC NMR spectrum of a mixture of Michael adduct 15b and pyridine-2-thiolate 16b, DMSO-d_6 (400/101 MHz) (Table 1, entry 7) (*fragments*)**

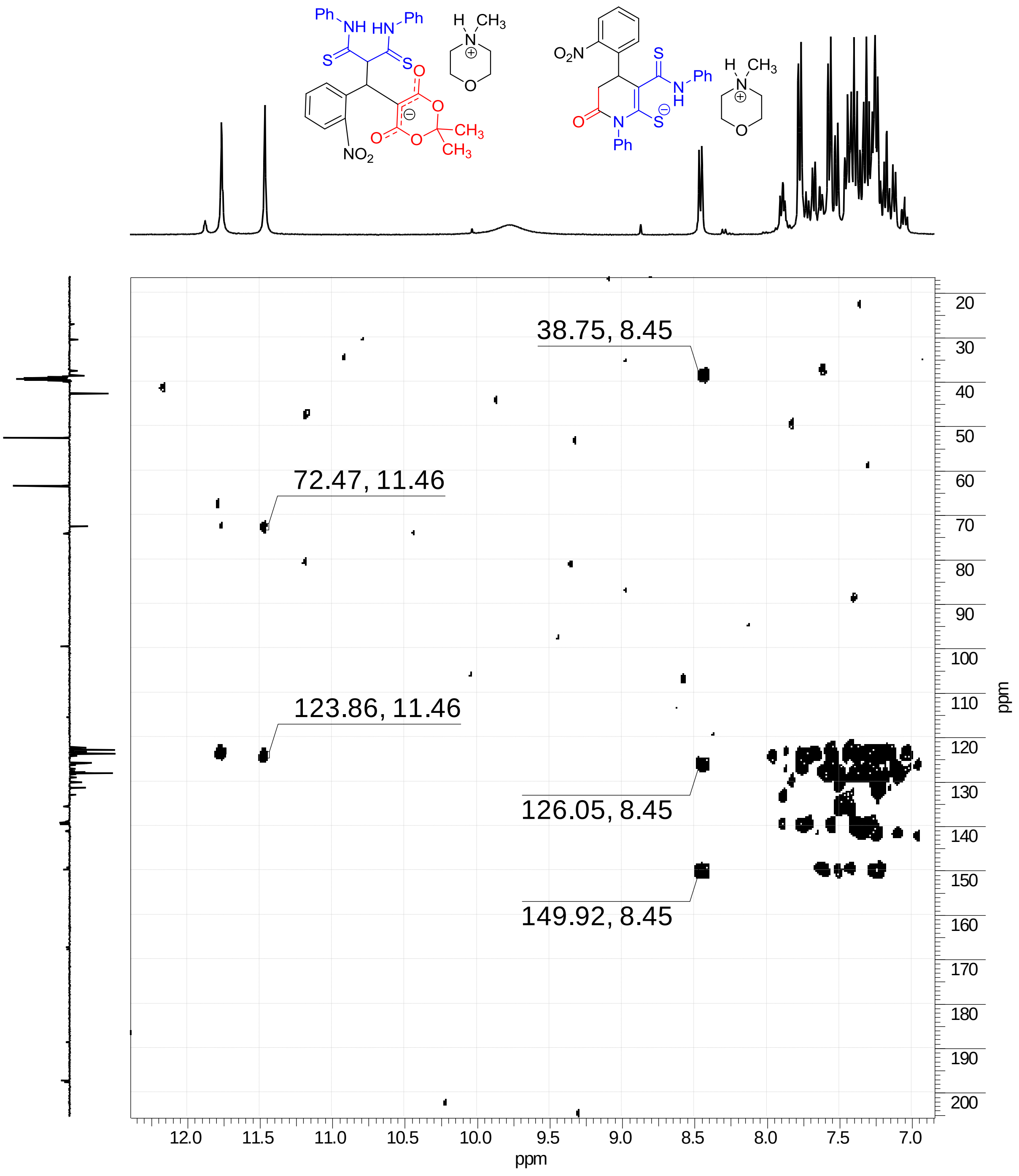

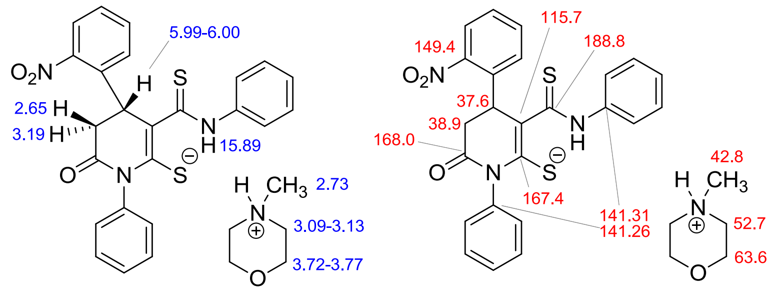

The assigned chemical shifts
based on the observed correlations

Figure S37. ^{1}**H NMR spectrum of the Michael adduct 15c, DMSO-d$_6$ (400 MHz) (Table 1, entry 8)**

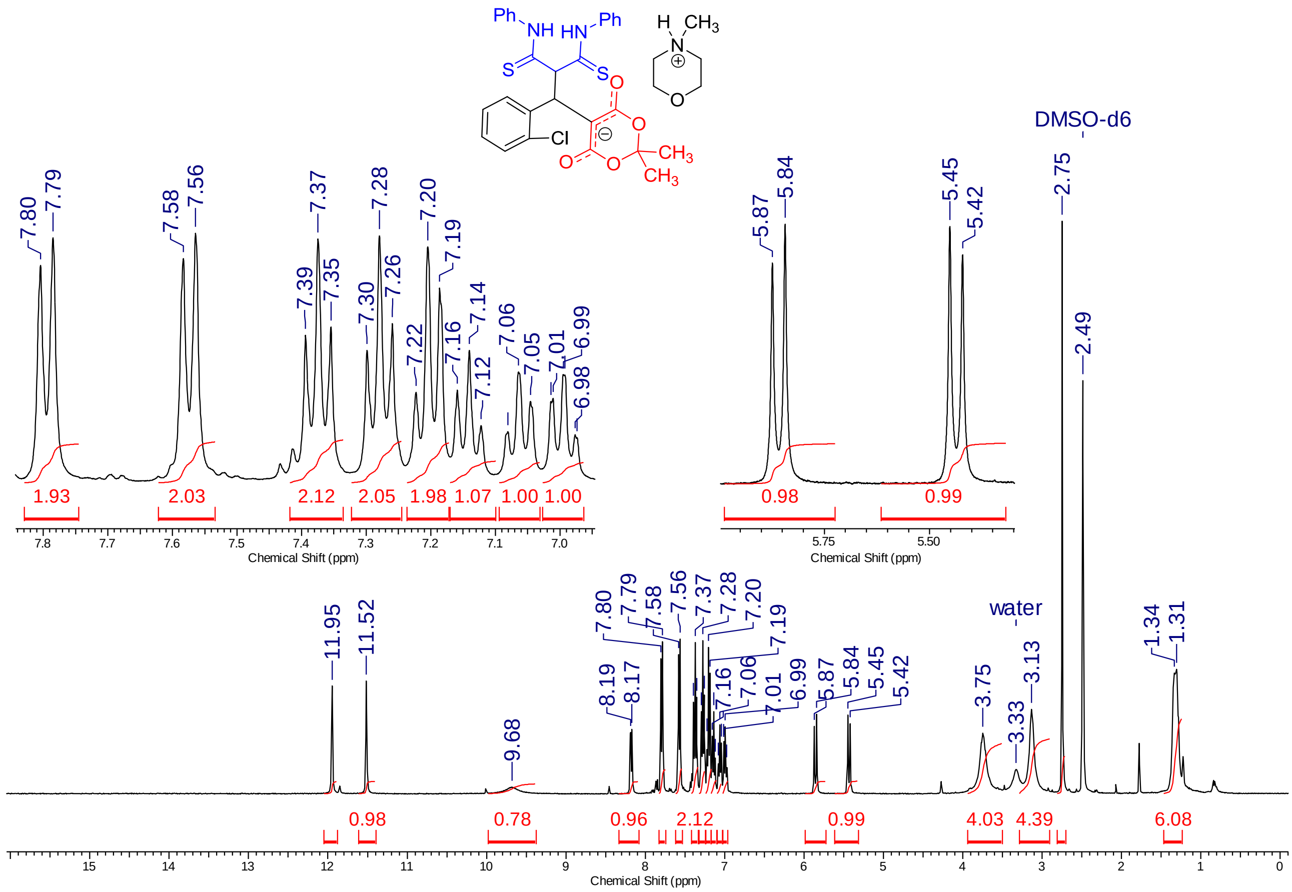

Figure S38. ^{13}**C DEPTQ NMR spectrum of the Michael adduct 15c, DMSO-d$_6$ (101 MHz) (Table 1, entry 8)**

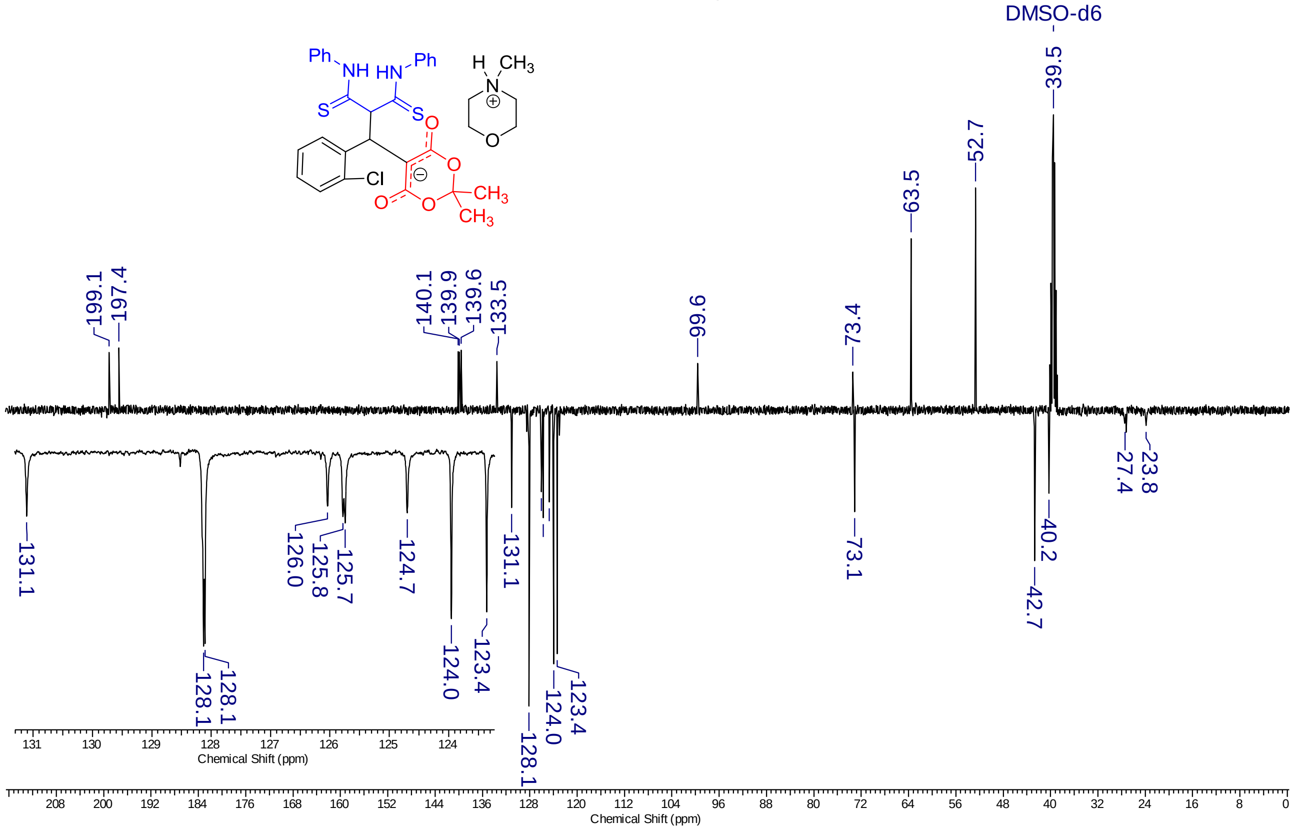

Figure S39. $^{1}H–^{13}C$ **HSQC NMR spectrum of the Michael adduct 15c, DMSO-d_6 (400/101 MHz) (Table 1, entry 8)**

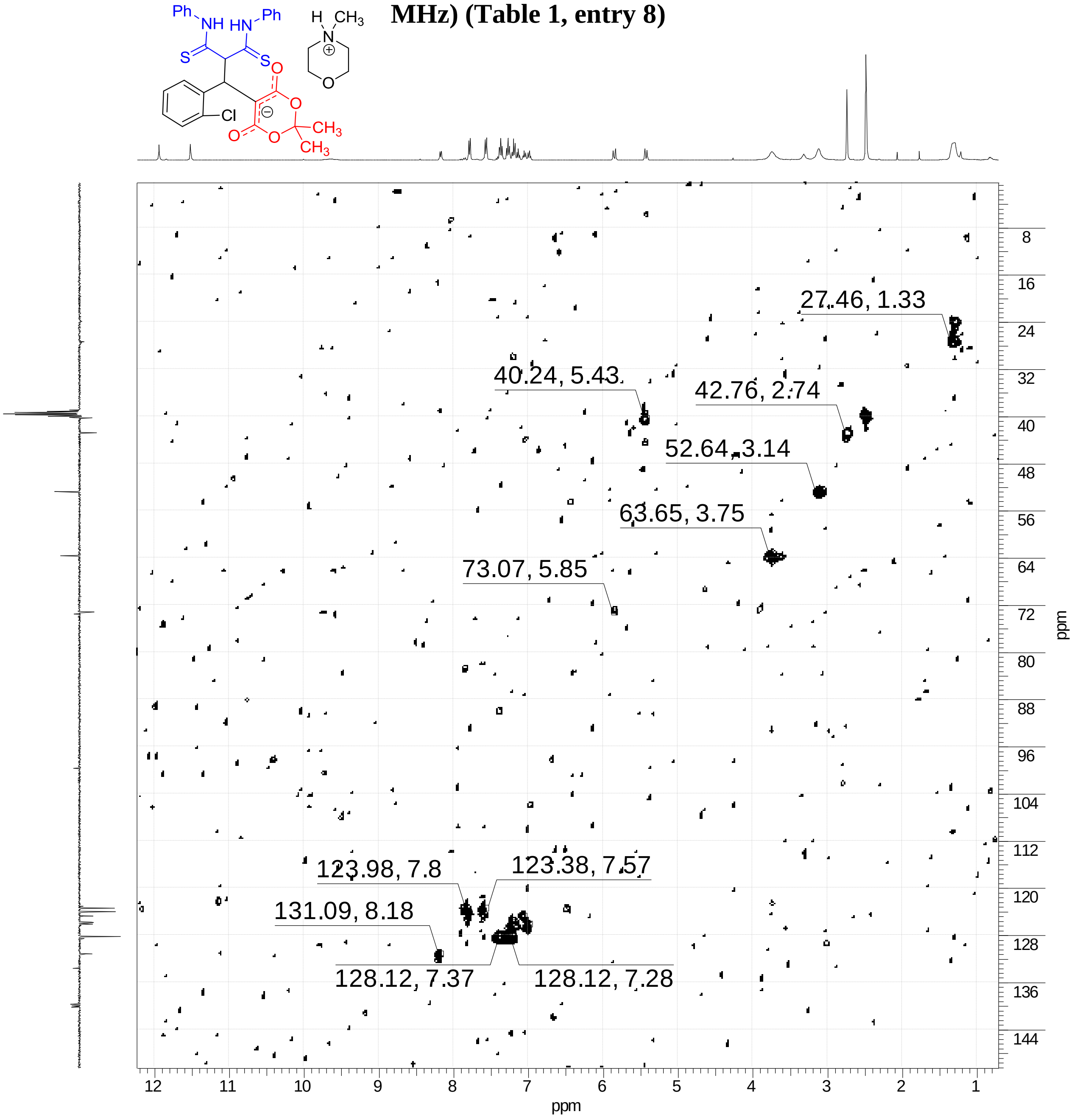

Figure S40. ^{1}H–^{13}C **HSQC NMR spectrum of the Michael adduct 15c, DMSO-d$_6$ (400/101 MHz) (Table 1, entry 8) (*fragments*)**

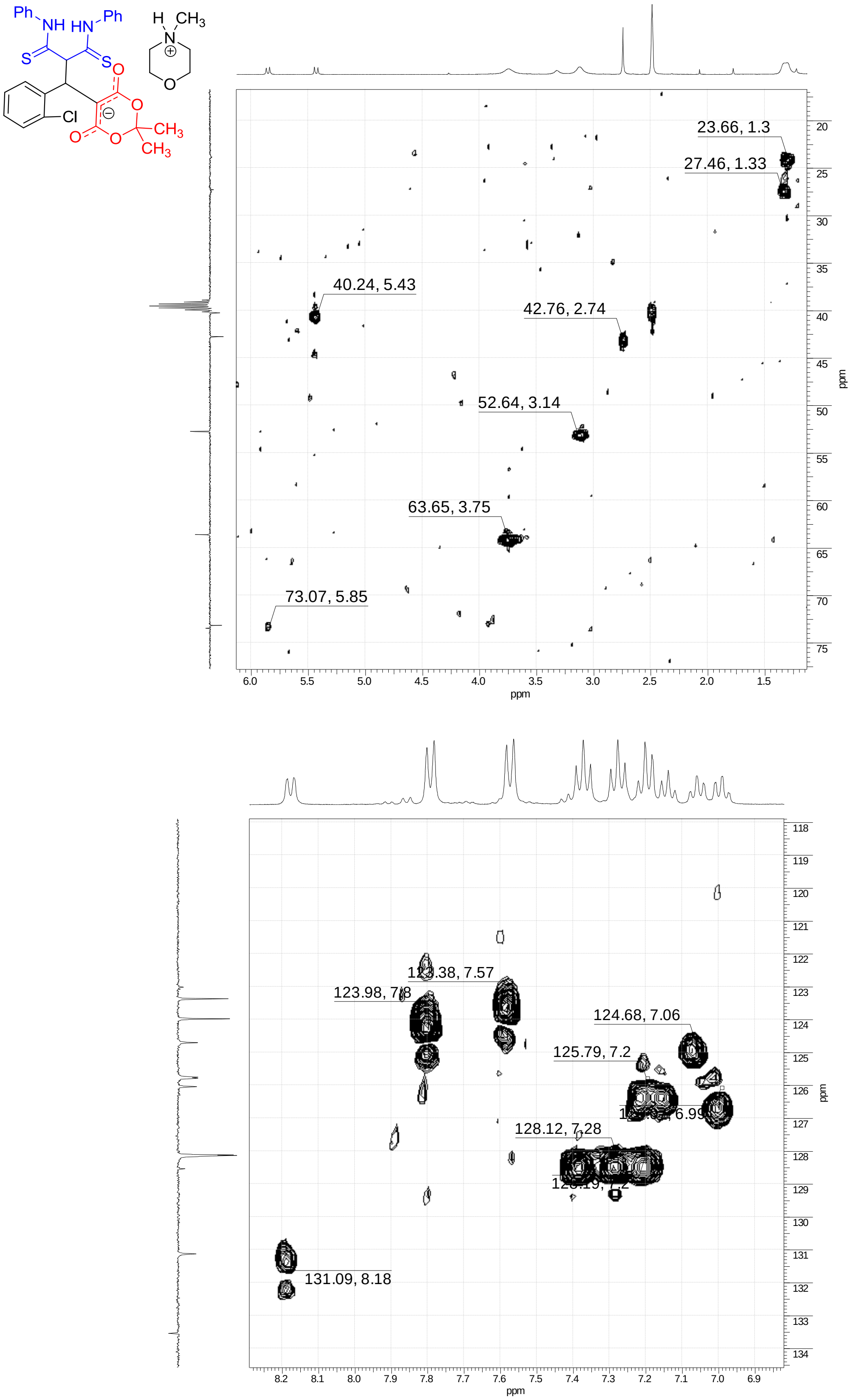

Figure S41. ^{1}H–^{13}C **HMBC NMR spectrum of the Michael adduct 15c, DMSO-d$_6$ (400/101 MHz) (Table 1, entry 8)**

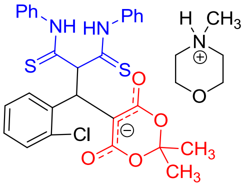

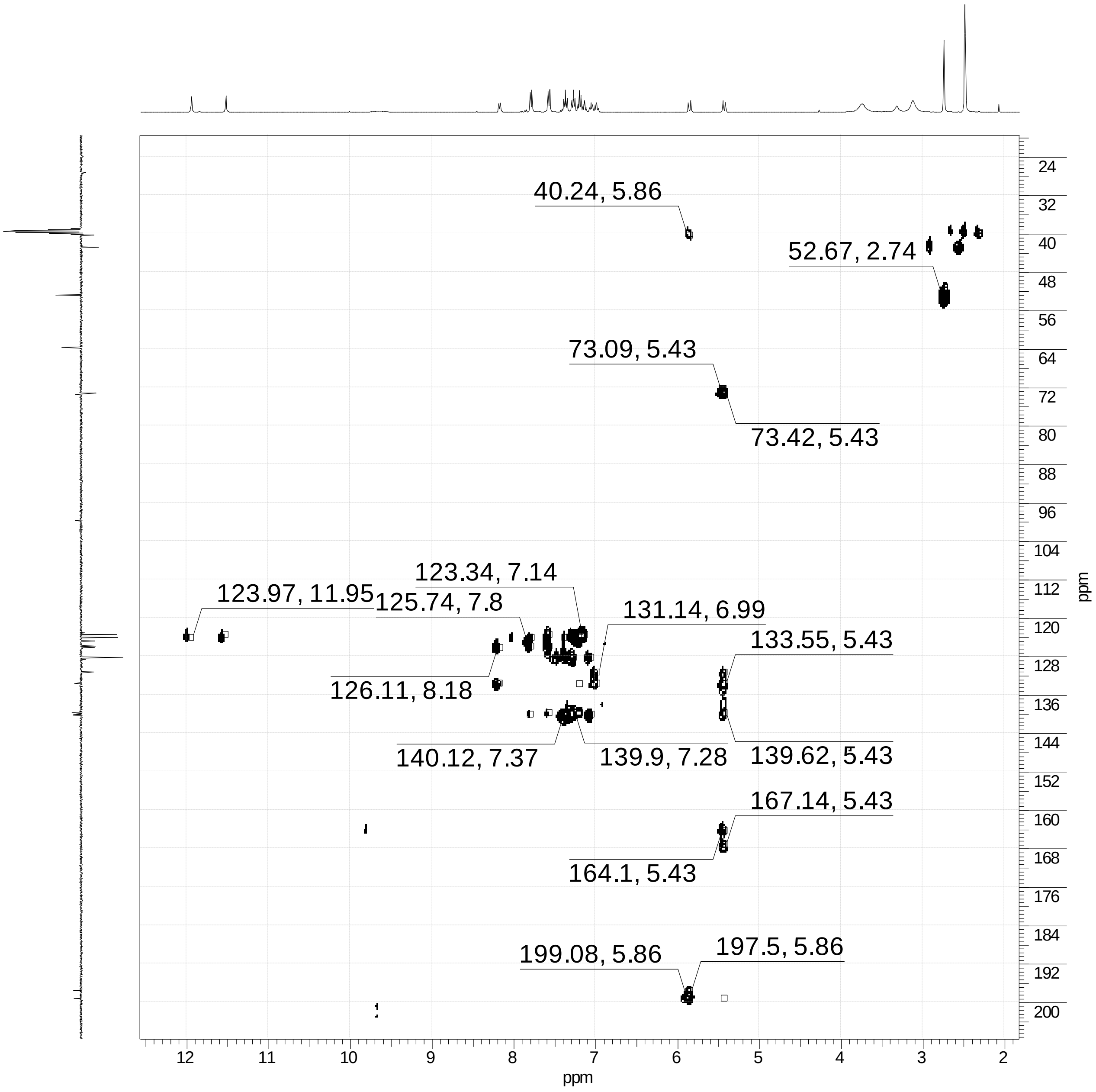
40.24, 5.86
52.67, 2.74
73.09, 5.43
73.42, 5.43
123.34, 7.14
123.97, 11.95
125.74, 7.8
131.14, 6.99
133.55, 5.43
126.11, 8.18
140.12, 7.37
139.9, 7.28
139.62, 5.43
167.14, 5.43
164.1, 5.43
199.08, 5.86
197.5, 5.86
ppm
ppm

Figure S42. ^{1}H–^{13}C **HMBC NMR spectrum of the Michael adduct 15c, DMSO-d_6 (400/101 MHz) (Table 1, entry 8) (*fragments*)**

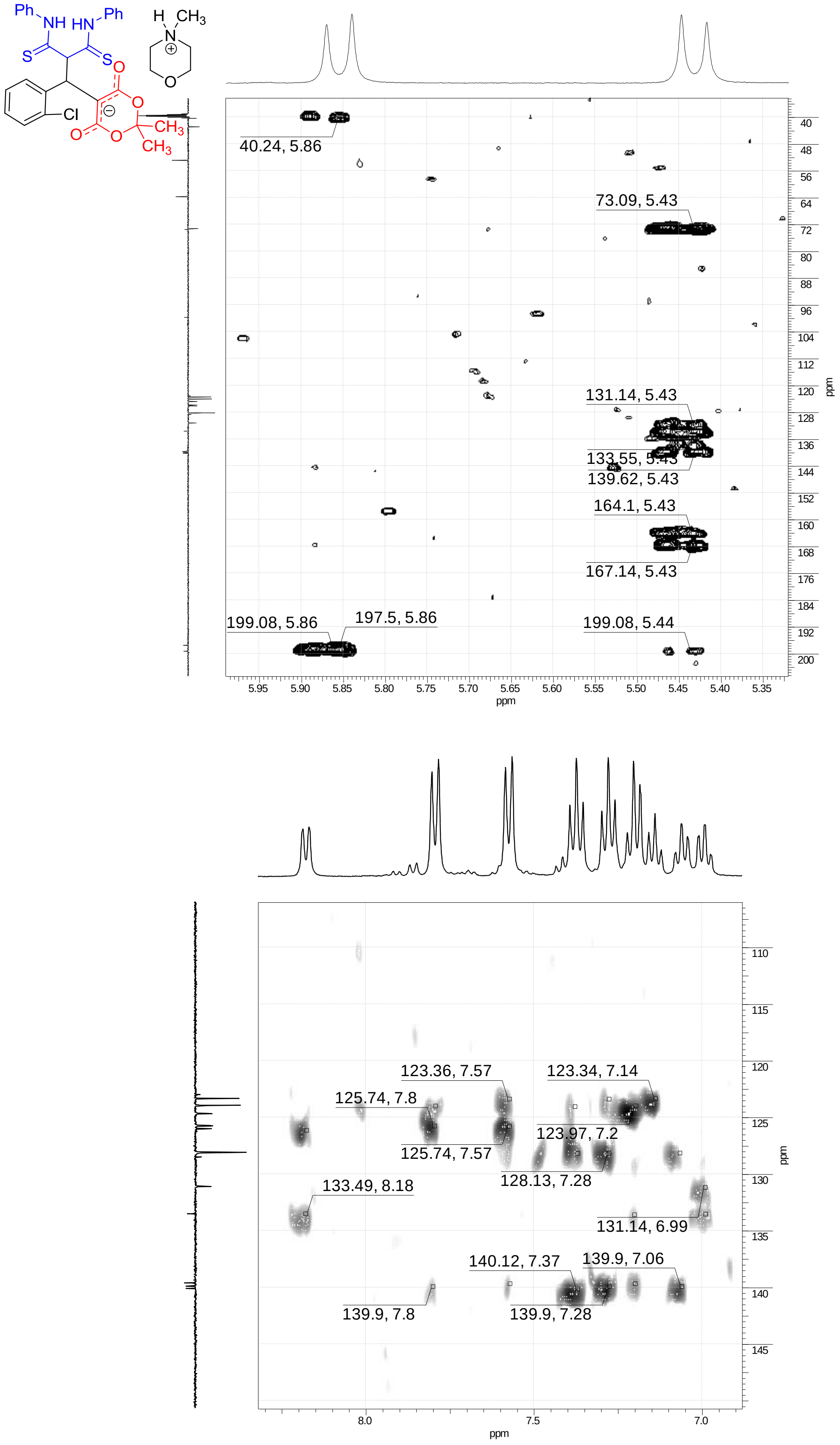

Table S3. The observed correlations in the ^{1}H-^{13}C HSQC and ^{1}H-^{13}C HMBC 2D NMR spectra of the Michael adduct 15c (Table 1, entry 8)

^{13}C chemical shifts are given in **red**, ^{1}H shifts – in **blue**

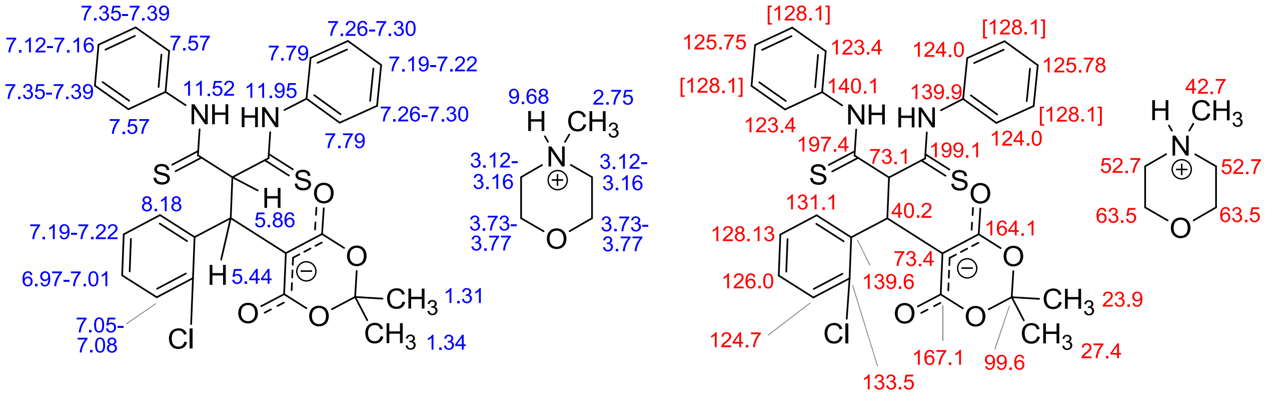

^{1}H NMR shifts, δ, ppm	Correlations in HSQC spectrum, δ, ppm	Correlations in HMBC spectrum, δ, ppm
1.31 (br s, 3H, Me)	23.9* (CH_3)	–
1.34 (br s, 3H, Me)	27.4* (CH_3)	–
2.75 (s, 3H, NMe)	42.7* (N–CH_3)	52.7 (CH_2NCH_2)
3.12-3.16 (m, 4H, CH_2NCH_2)	52.7 (CH_2NCH_2)	–
3.73-3.77 (m, 4H, CH_2OCH_2)	63.5 (CH_2OCH_2)	–
5.44 (d, ^{3}J = 12.1 Hz, 1H, C<u>H</u>-Ar)	40.2* (<u>C</u>H–Ar)	73.1* (<u>C</u>H–CSNHPh), 73.4 (<u>C</u>–C=O), 131.1* (C-6 4-ClC_6H_4), 133.5 (C-Cl), 139.6 (C-1 4-ClC_6H_4), 164.1, 167.1 (br s, 2 C–O^-), 199.1 (C=S) (weak)
5.86 (d, ^{3}J = 12.1 Hz, 1H, C<u>H</u>-CSNHPh)	73.1* (<u>C</u>H–CSNHPh)	40.2* (<u>C</u>H–Ar), 197.4 (C=S), 199.1 (C=S)
6.97-7.01 (m, 1H, H-4 4-ClC_6H_4)	126.0* (C-4 4-ClC_6H_4)	131.1* (C-6 4-ClC_6H_4), 133.5 (C-Cl)
7.05-7.08 (m, 1H, H-3 4-ClC_6H_4)	124.7* (C-3 4-ClC_6H_4)	128.13* (C-3, C-5 Ph and C-5 4-ClC_6H_4 overlapped), 139.6 (C-1 4-ClC_6H_4)
7.12-7.16 (m, 1H, H-4 Ph)	125.75* (C-4 Ph)	123.4* (C-2, C-6 Ph)
7.19-7.22 (m, 2H, H-4 Ph and H-5 4-ClC_6H_4 overlapped)	125.78* (C-4 Ph), 128.13* (C-3, C-5 Ph and C-5 4-ClC_6H_4 overlapped)	124.0* (C-2, C-6 Ph), 133.5 (C-Cl), 139.6 (C-1 4-ClC_6H_4),
7.26-7.30 (m, 2H, H-3, H-5 Ph)	128.11* (C-3, C-5 Ph), 128.13* (C-3, C-5 Ph and C-5 4-ClC_6H_4 overlapped)**	123.4* (C-2, C-6 Ph) (weak), [128.1]**, 140.1 (C-1 Ph)
7.35-7.39 (m, 2H, H-3, H-5 Ph)		124.0* (C-2, C-6 Ph) (weak), [128.1]**, 139.9 (C-1 Ph)
7.57 (d, ^{3}J = 7.6 Hz, 2H, H-2, H-6 Ph)	123.4* (C-2, C-6 Ph)	123.4* (C-2, C-6 Ph), 125.75* (C-4 Ph), 140.1 (C-1 Ph)
7.79 (d, ^{3}J = 7.7 Hz, 2H, H-2, H-6 Ph)	124.0* (C-2, C-6 Ph),	124.0* (C-2, C-6 Ph), 125.78* (C-4 Ph), 139.9 (C-1 Ph)
8.18 (d, ^{3}J = 7.0 Hz, 1H, H-6 4-ClC_6H_4)	131.1* (C-6 4-ClC_6H_4)	126.0* (C-4 4-ClC_6H_4), 133.5 (C-Cl)
9.68 (br s, 1H, HN^+)	–	–
11.52 (s, 1H, C(S)NH)	–	123.4* (C-2, C-6 Ph)
11.95 (s, 1H, C(S)NH)	–	124.0* (C-2, C-6 Ph)

*Signals with a negative phase.

**Due to the close positions of signals, it is difficult to make an accurate cross peaks assignment.

Figure S43. FTIR spectrum of the Michael adduct 15c (Table 1, entry 8)

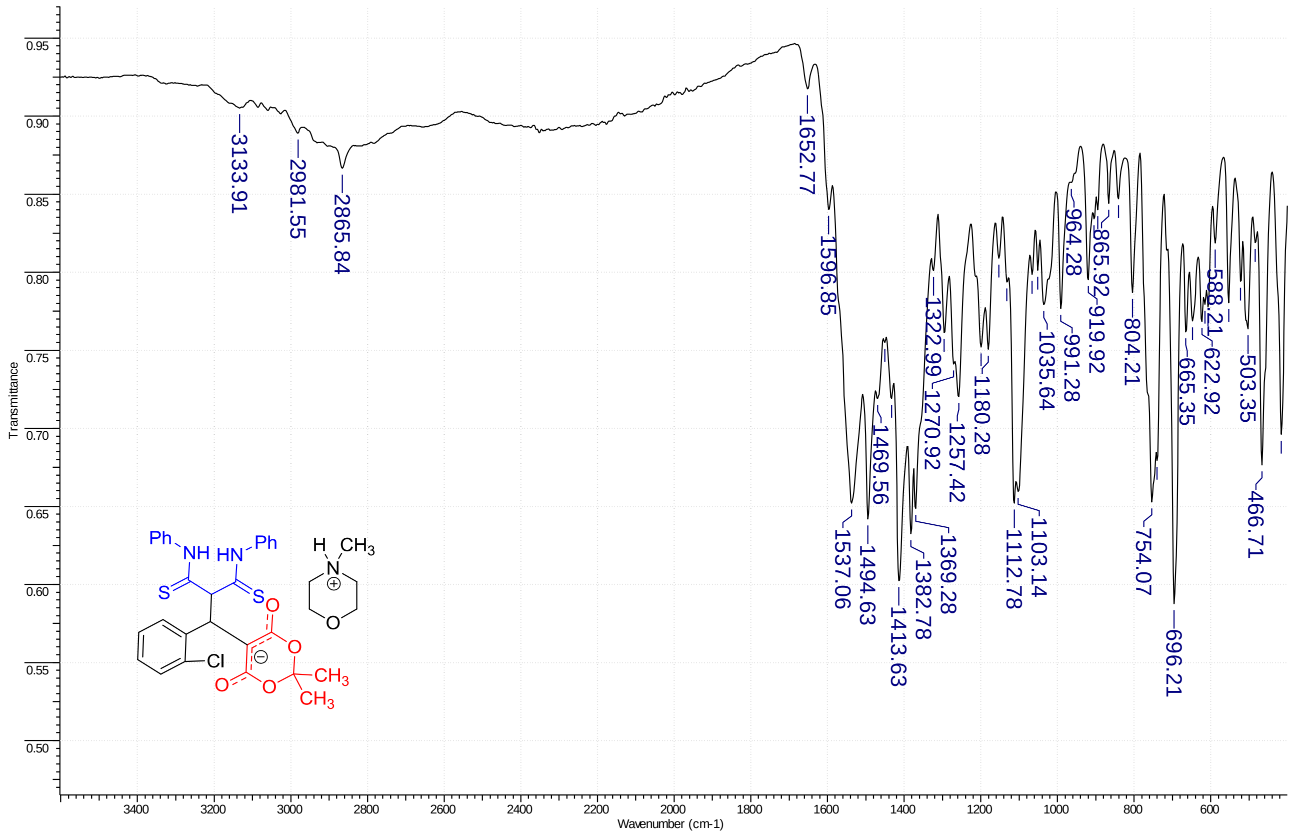

Figure S44. ^{1}**H NMR spectrum of the Michael adduct 15c, DMSO-d$_6$ (400 MHz) (Table 1, entry 9)**

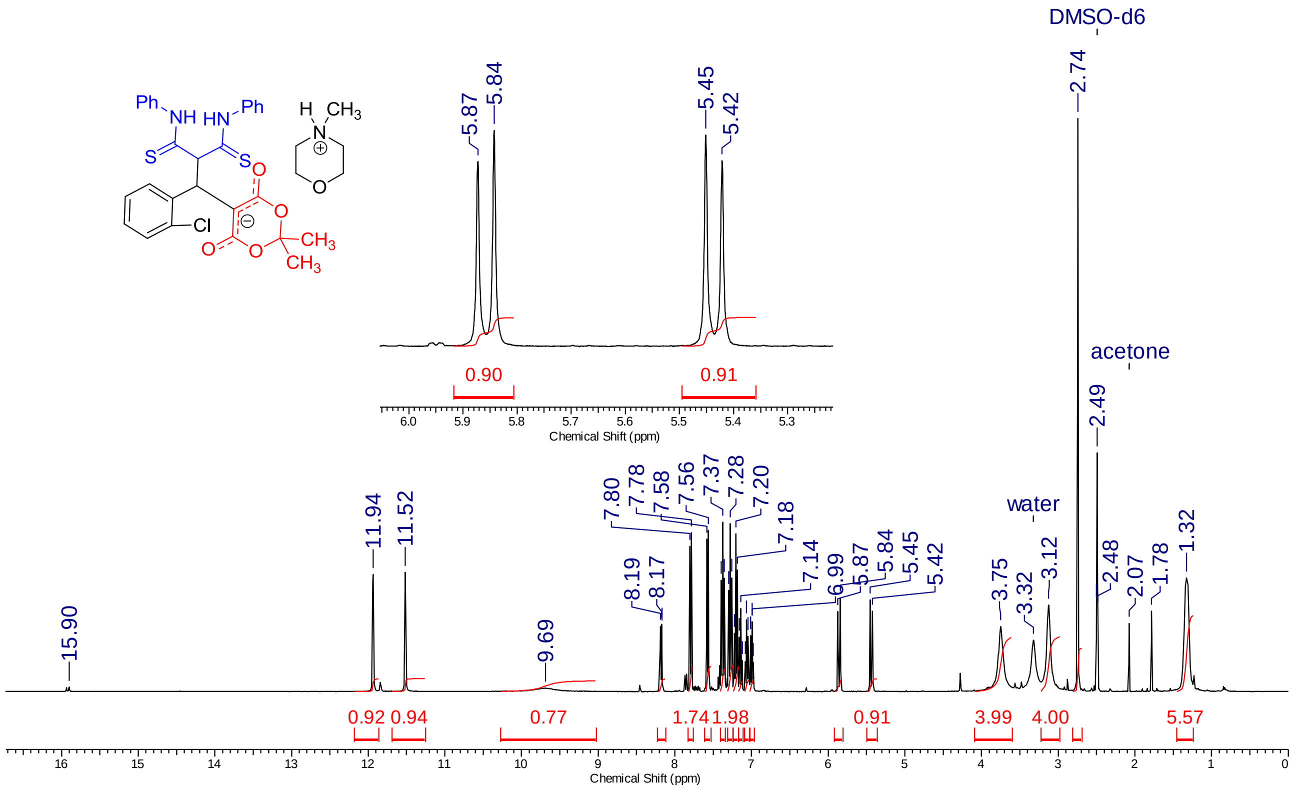

Figure S45. ^{13}**C DEPTQ NMR spectrum of the Michael adduct 15c, DMSO-d$_6$ (101 MHz) (Table 1, entry 9)**

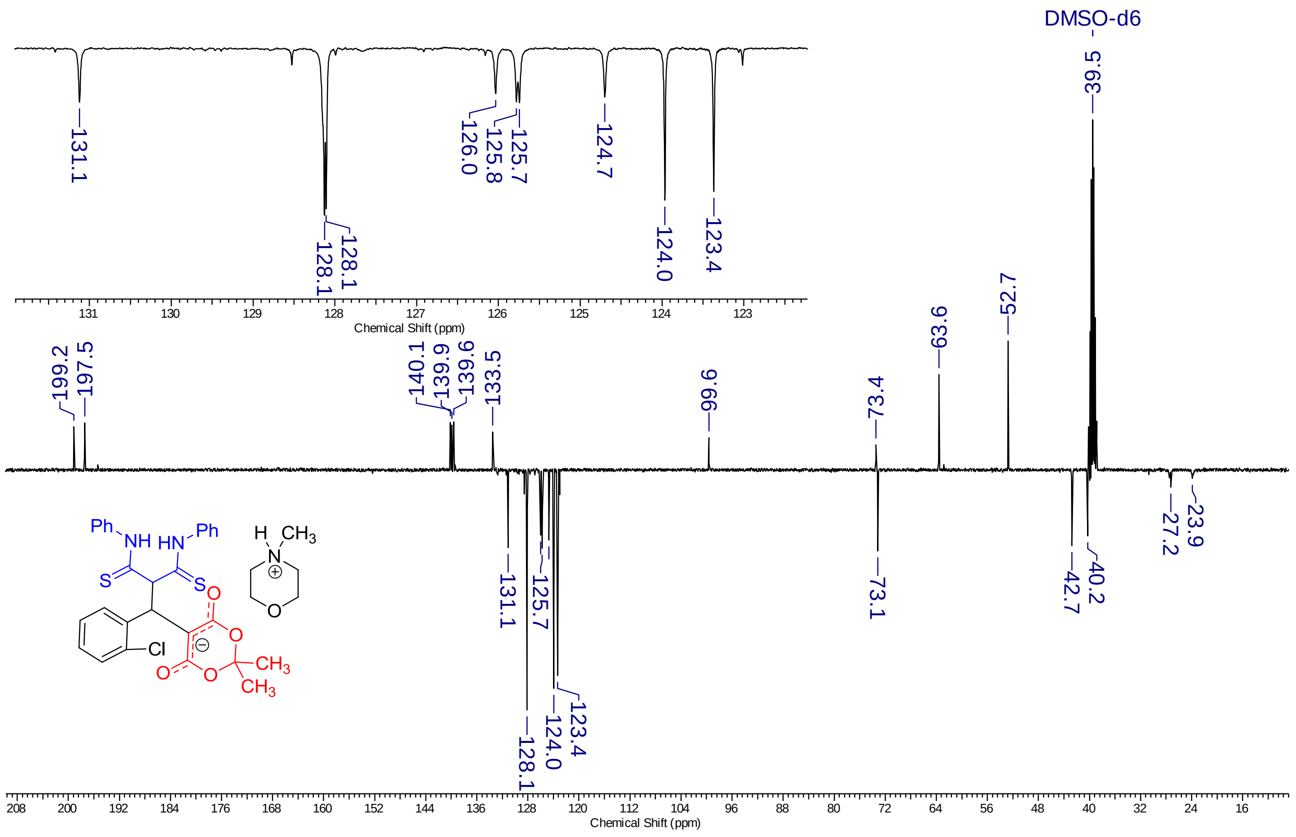

Figure S46. ^{1}**H NMR spectrum of a mixture of Michael adduct 15d and cyclization product 16d, DMSO-d$_6$ (400 MHz) (Table 1, entry 11)**

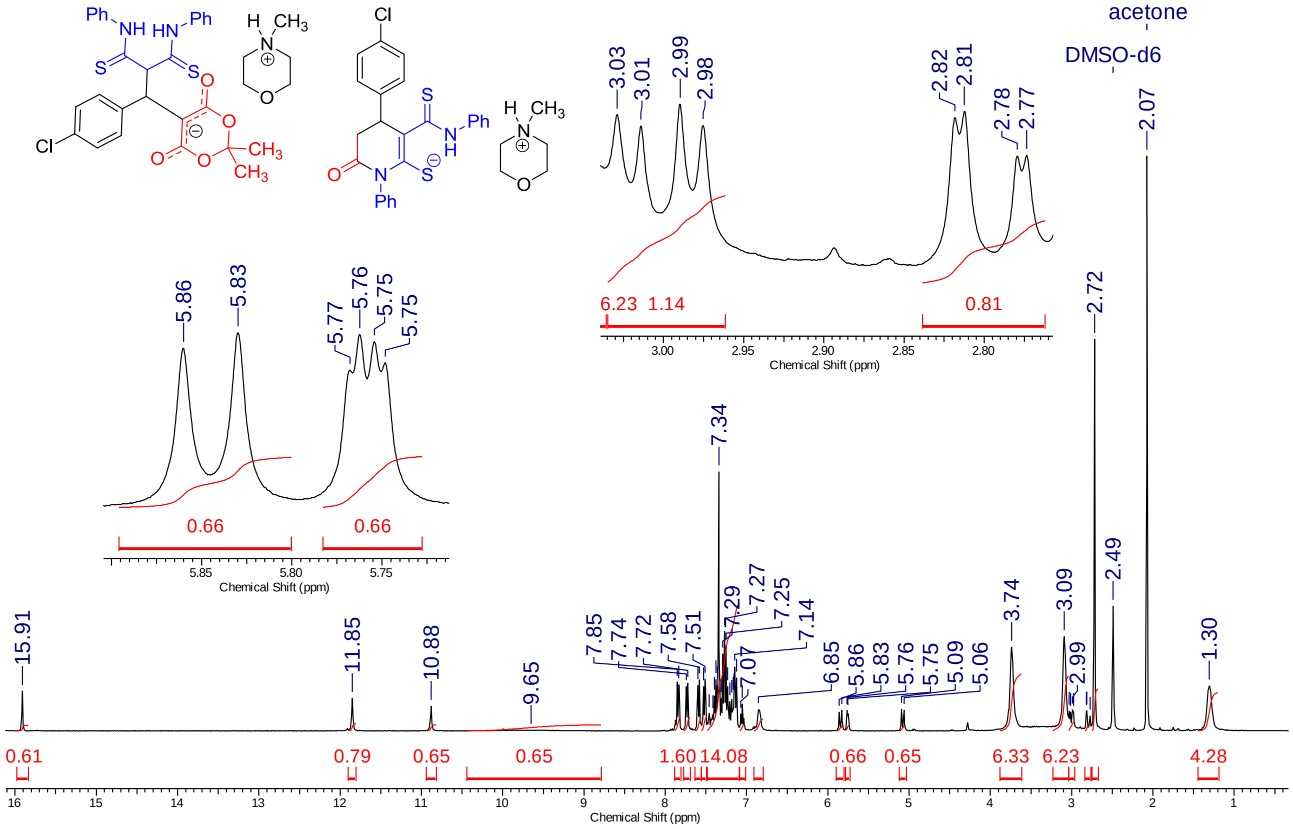

Figure S47. ^{13}**C DEPTQ NMR spectrum of a mixture of Michael adduct 15d and cyclization product 16d, DMSO-d$_6$ (101 MHz) (Table 1, entry 11)**

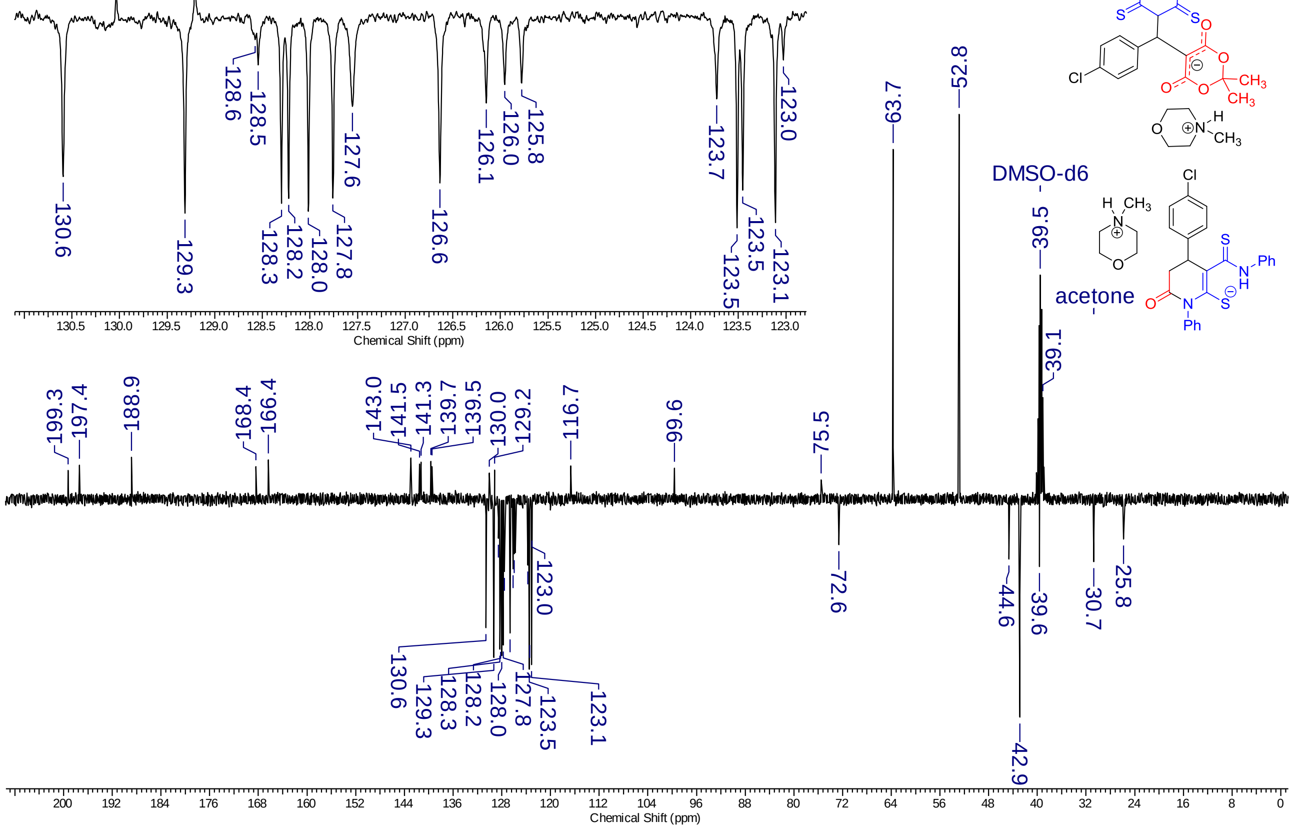

Figure S48. ^{1}H–^{13}C HSQC NMR spectrum of a mixture of Michael adduct 15d and cyclization product 16d, DMSO-d_6 (400/101 MHz) (Table 1, entry 11)

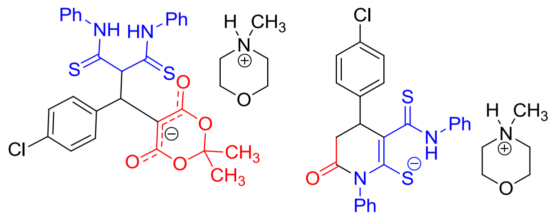

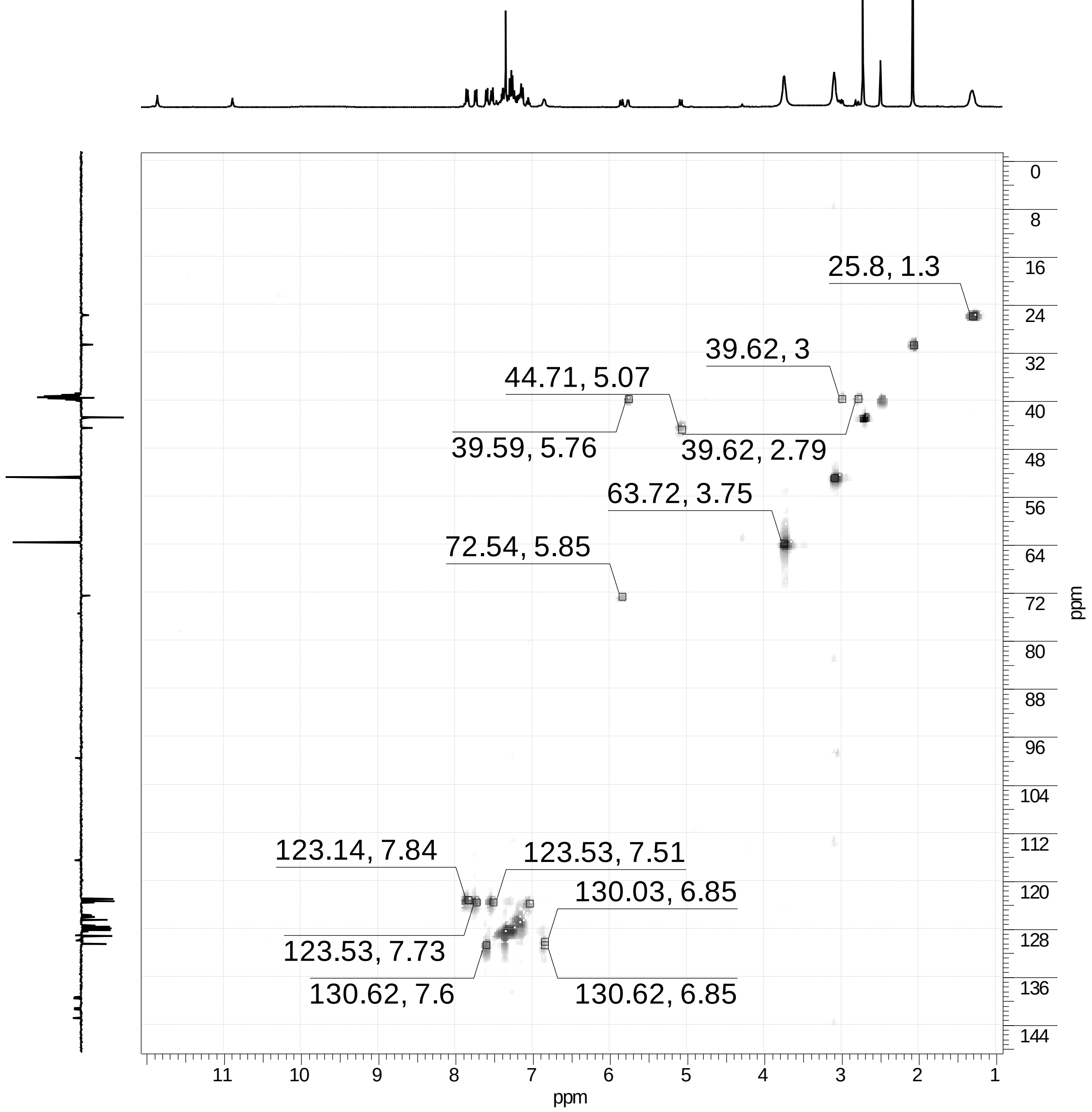

Figure S49. **^{1}H–^{13}C HSQC NMR spectrum of a mixture of Michael adduct 15d and cyclization product 16d, DMSO-d_6 (400/101 MHz) (Table 1, entry 11) (*fragments*)**

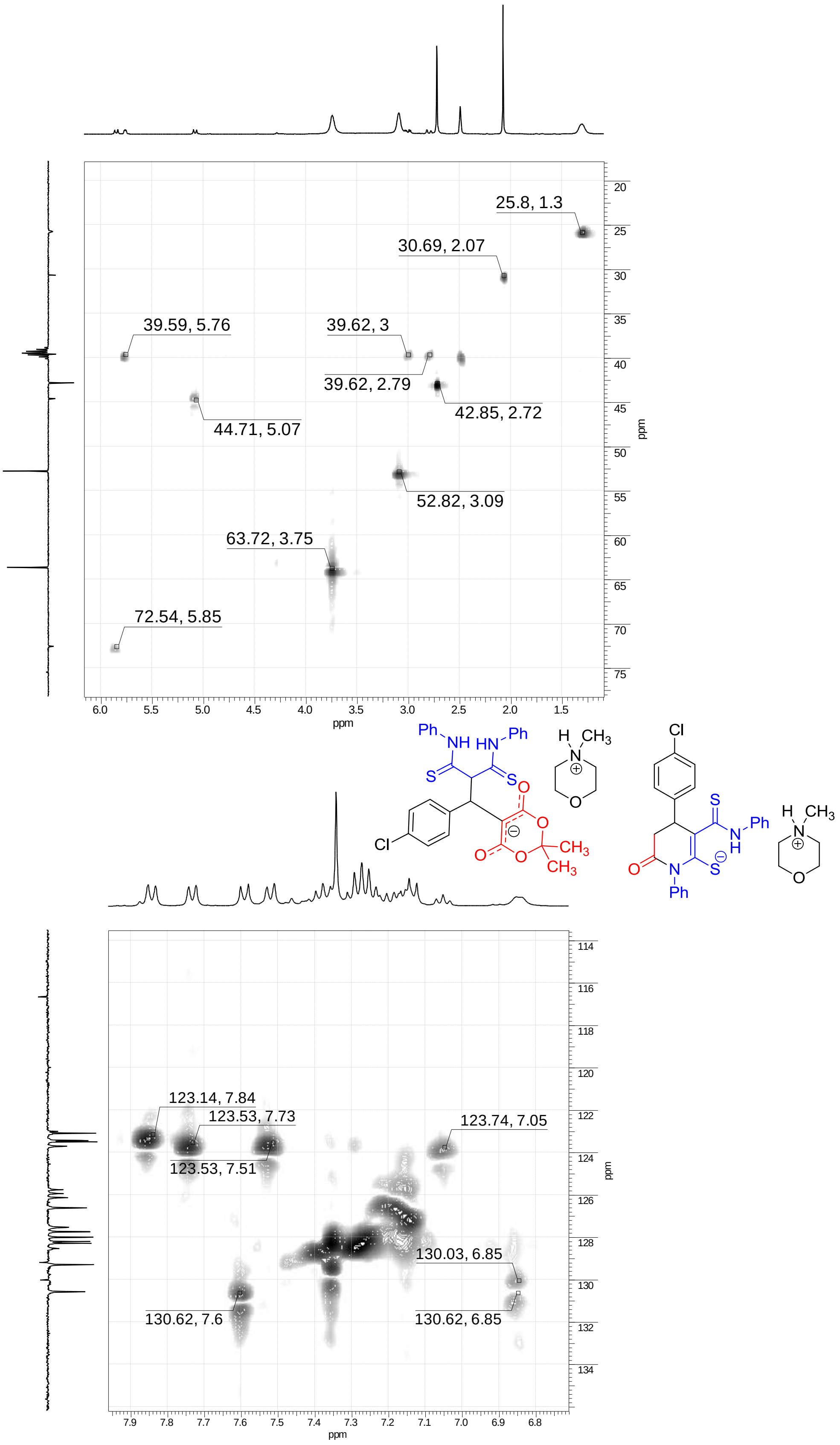

Figure S50. ^{1}H–^{13}C **HMBC NMR spectrum of a mixture of Michael adduct 15d and cyclization product 16d, DMSO-d$_6$ (400/101 MHz) (Table 1, entry 11)**

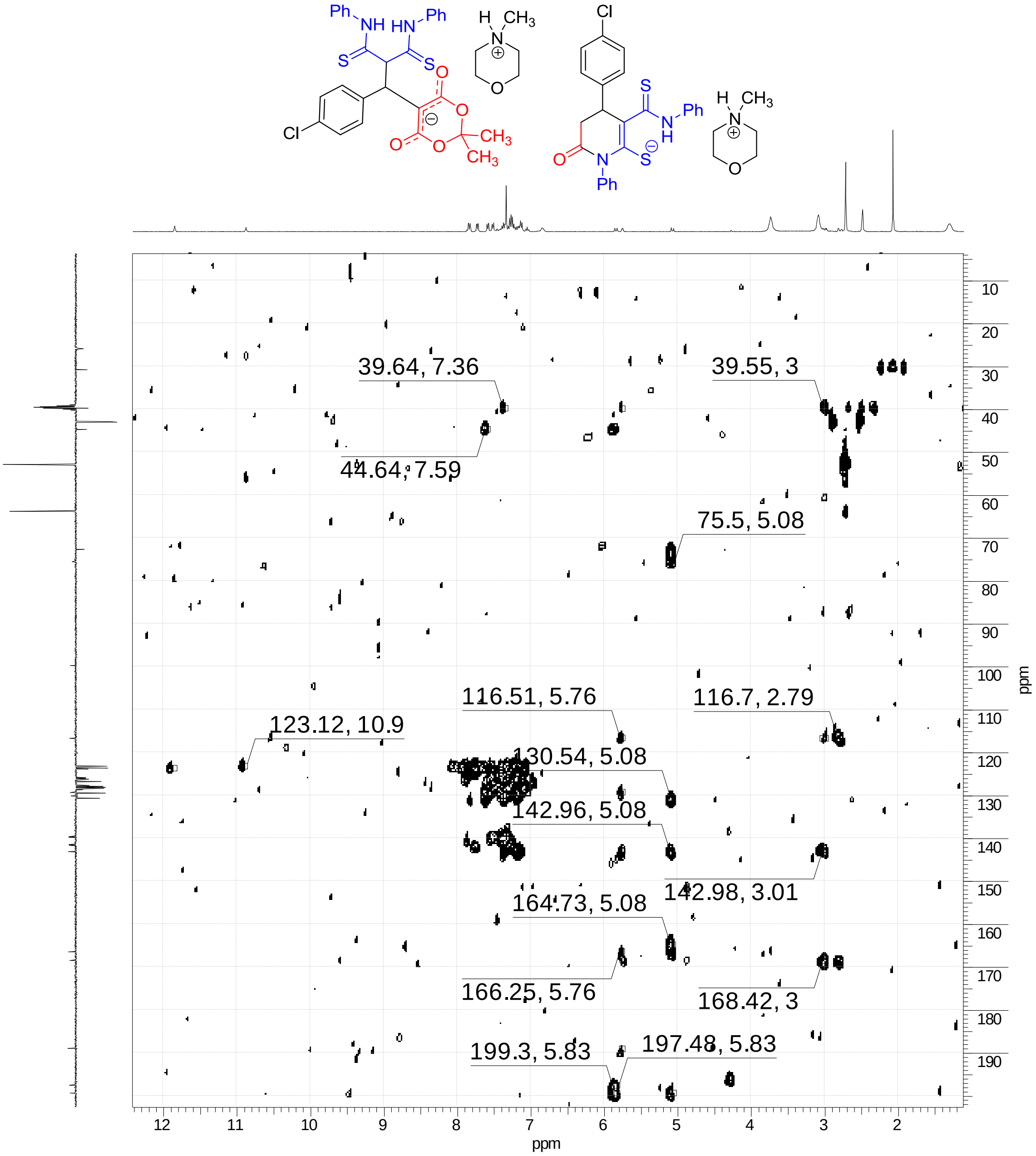

Figure S51. ^{1}H–^{13}C **HMBC NMR spectrum of a mixture of Michael adduct 15d and cyclization product 16d, DMSO-d$_6$ (400/101 MHz) (Table 1, entry 11) (*fragments*)**

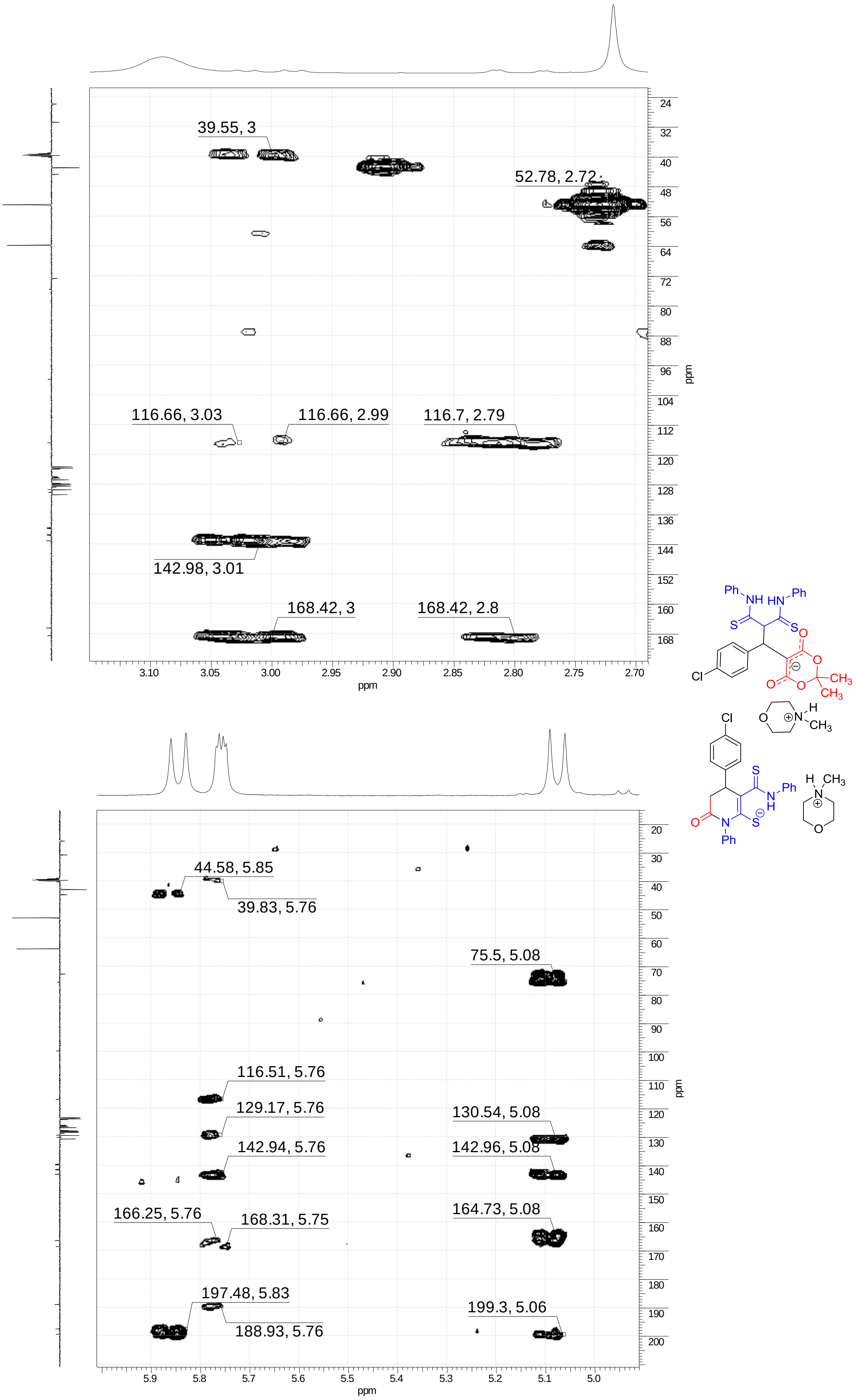

Figure S52. ^{1}H NMR spectrum of a mixture of Michael adduct 15e and cyclization product 16e, DMSO-d_6 (400 MHz) (Table 1, entry 12)

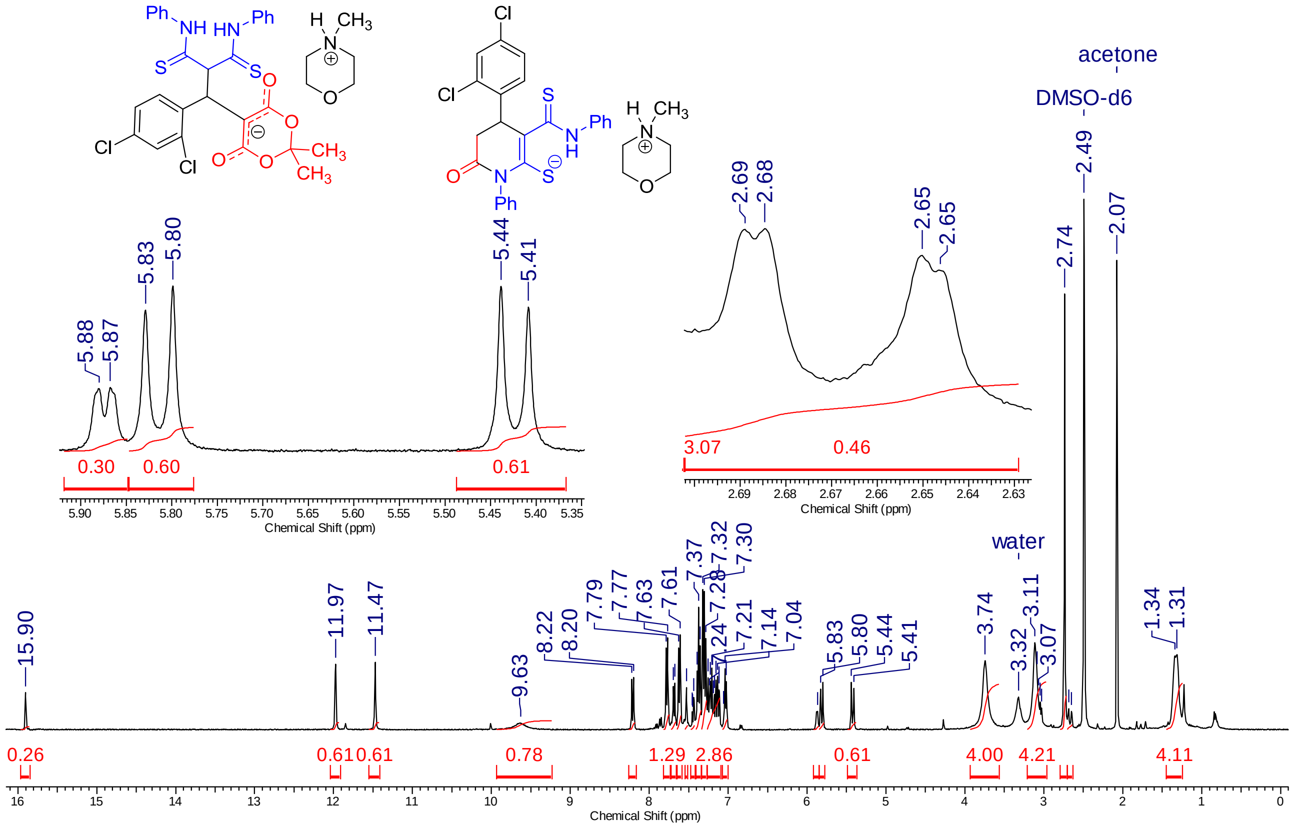

Figure S53. ^{13}C DEPTQ NMR spectrum of a mixture of Michael adduct 15e and cyclization product 16e, DMSO-d_6 (101 MHz) (Table 1, entry 12)

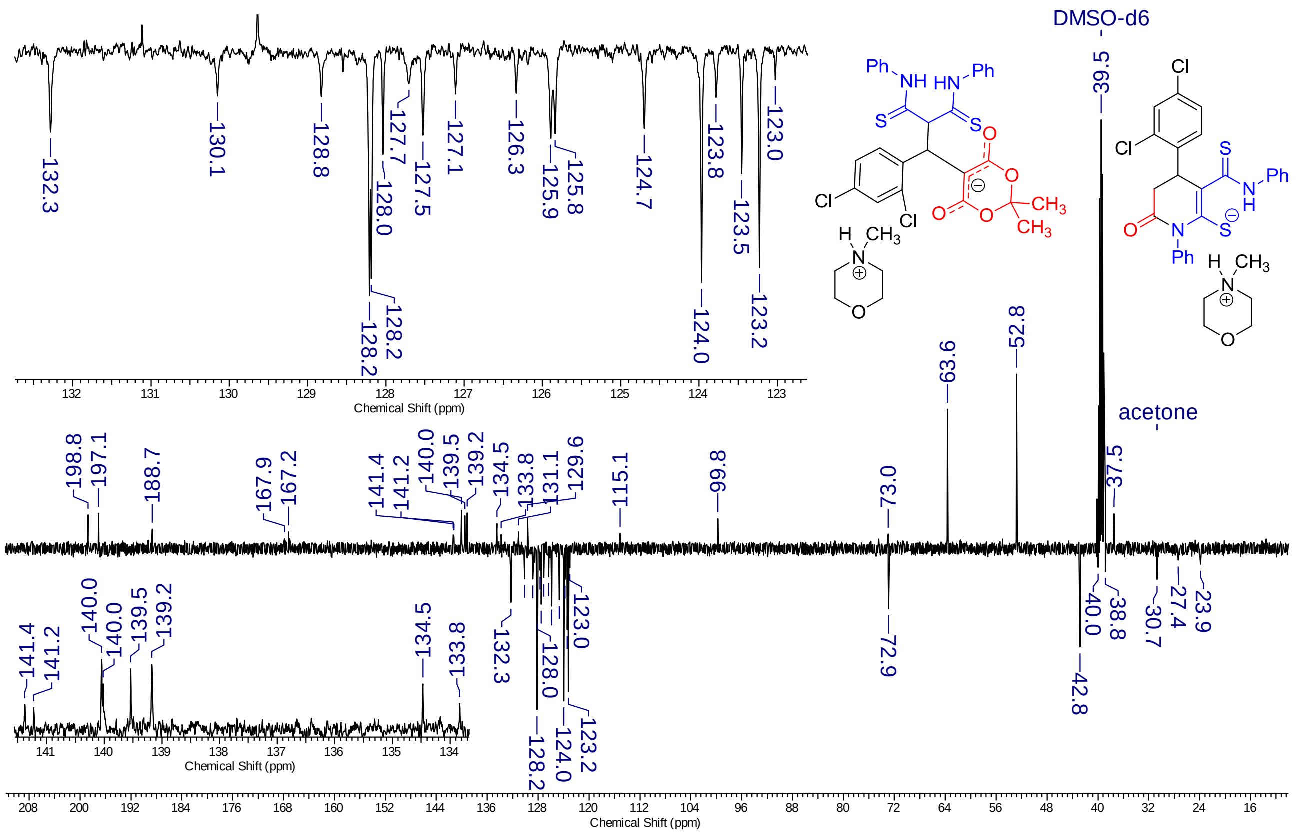

Figure S54. ^{1}H NMR spectrum of a mixture of 15g,16g,17g, DMSO-d_6 (400 MHz) (Table 1, entry 15)

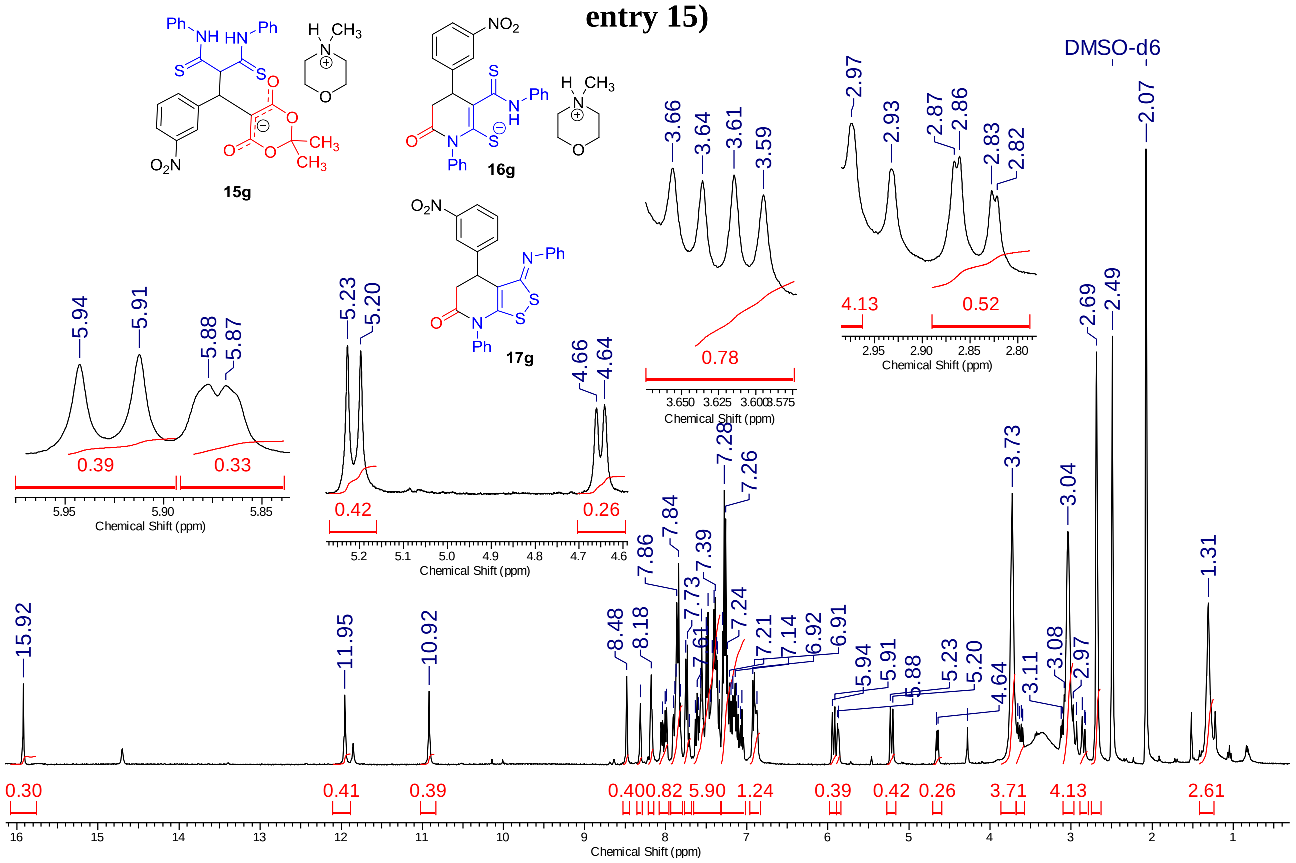

Figure S55. ^{13}C DEPTQ NMR spectrum of a mixture of 15g,16g,17g, DMSO-d_6 (101 MHz) (Table 1, entry 15)

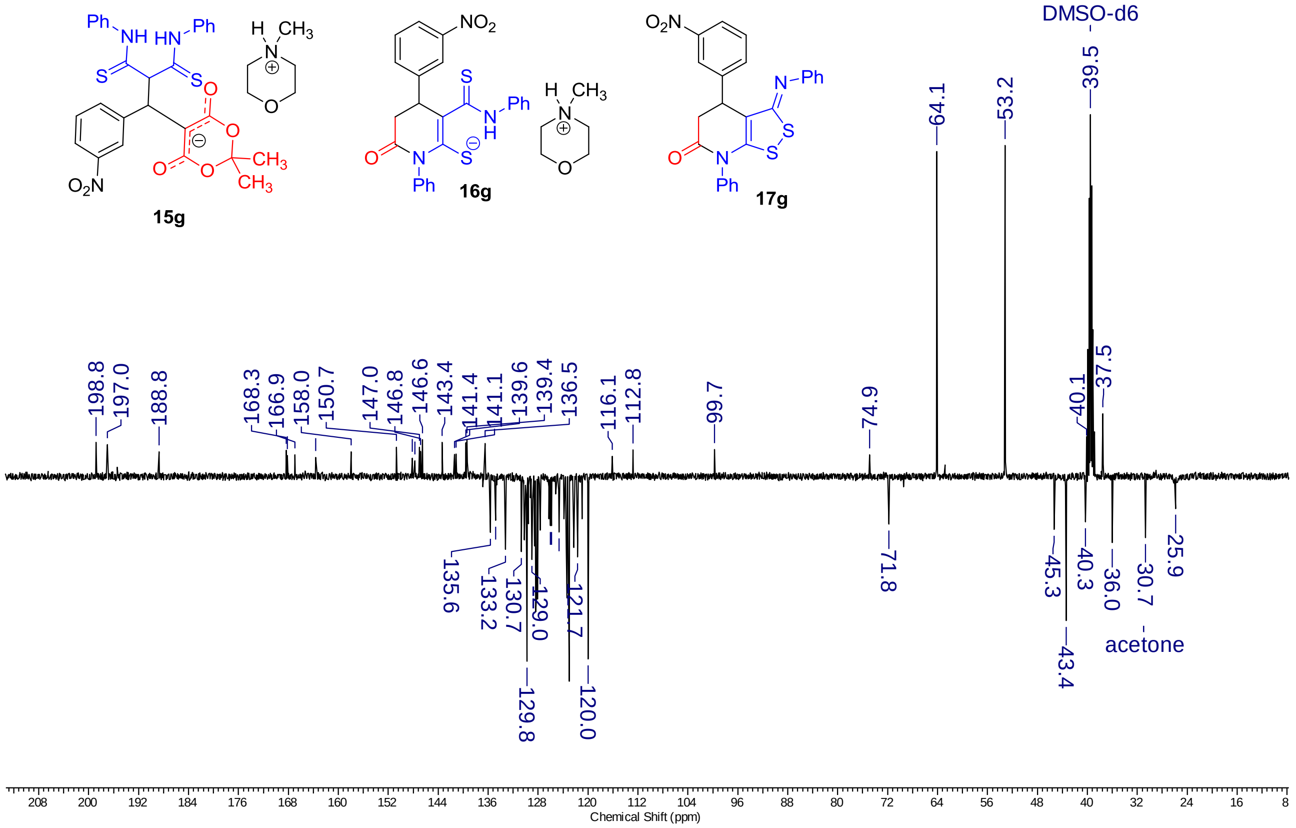

Figure S56. ^{1}**H NMR spectrum of the products of reaction of 4-methoxybenzylidene Meldrum's acid 14h with dithiomalondianilide 1, DMSO-d$_6$ (400 MHz) (Table 1, entry 17)**

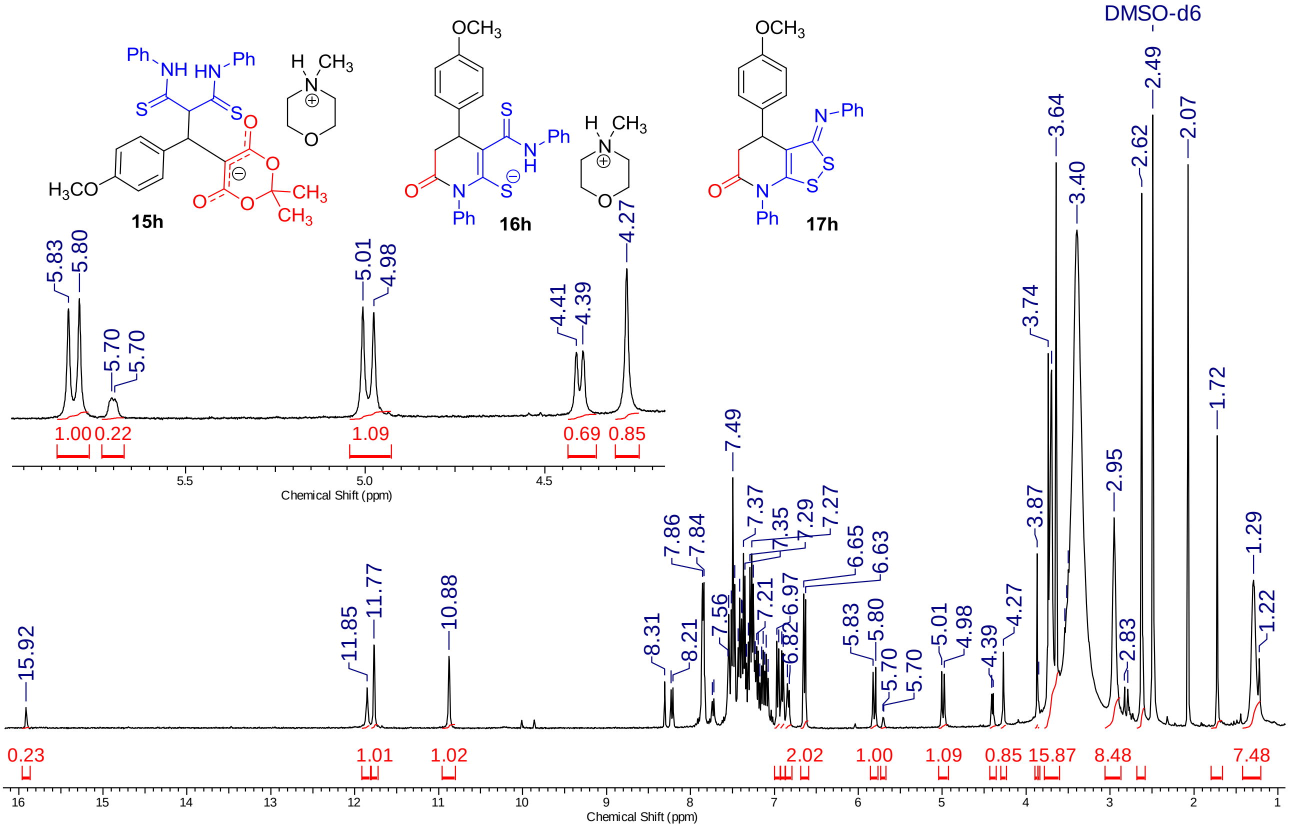

Figure S57. ^{13}**C DEPTQ NMR spectrum of the products of reaction of 4-methoxybenzylidene Meldrum's acid 14h with dithiomalondianilide 1, DMSO-d$_6$ (400 MHz) (Table 1, entry 17)**

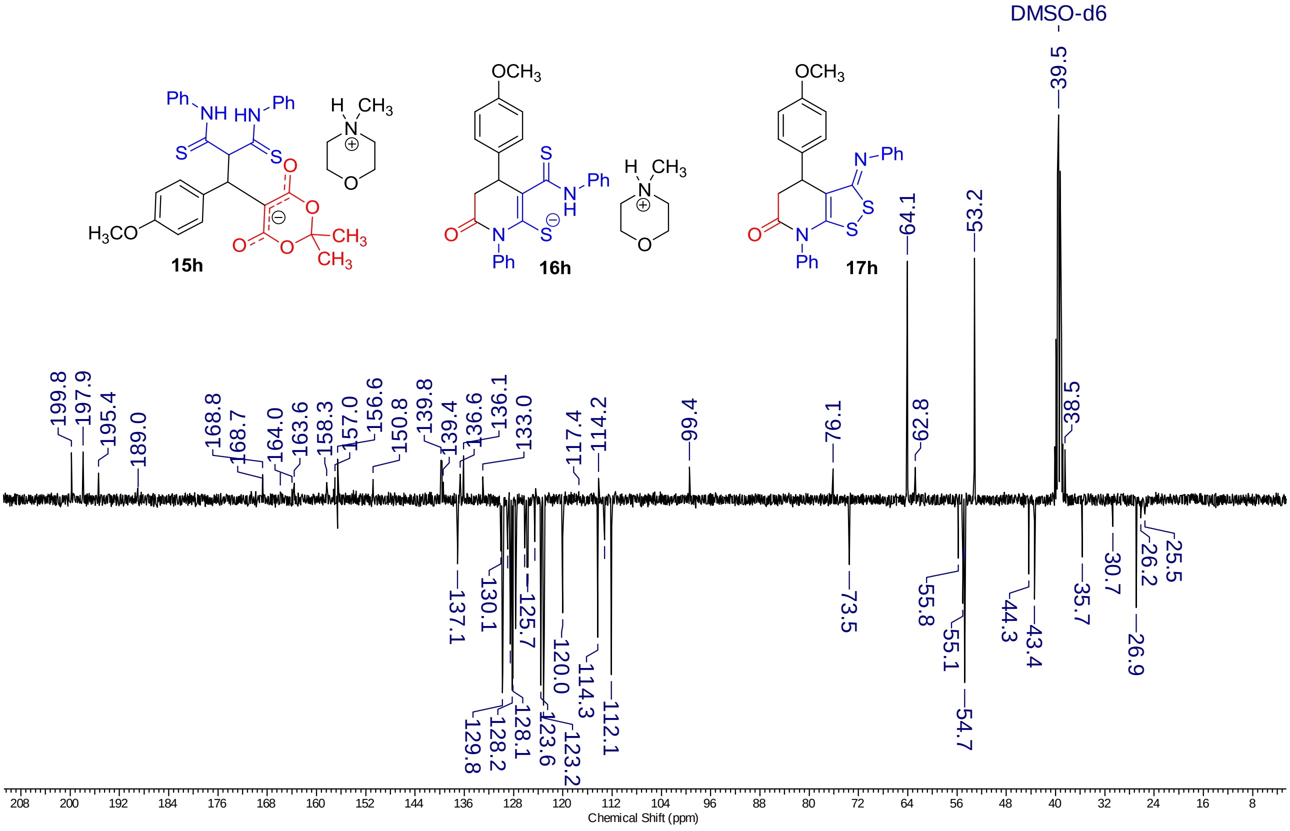

Figure S58. ^{1}H NMR spectrum of the products of reaction of 4-hydroxybenzylidene Meldrum's acid 14i with dithiomalondianilide 1, DMSO-d$_6$ (400 MHz) (Table 1, entry 18)

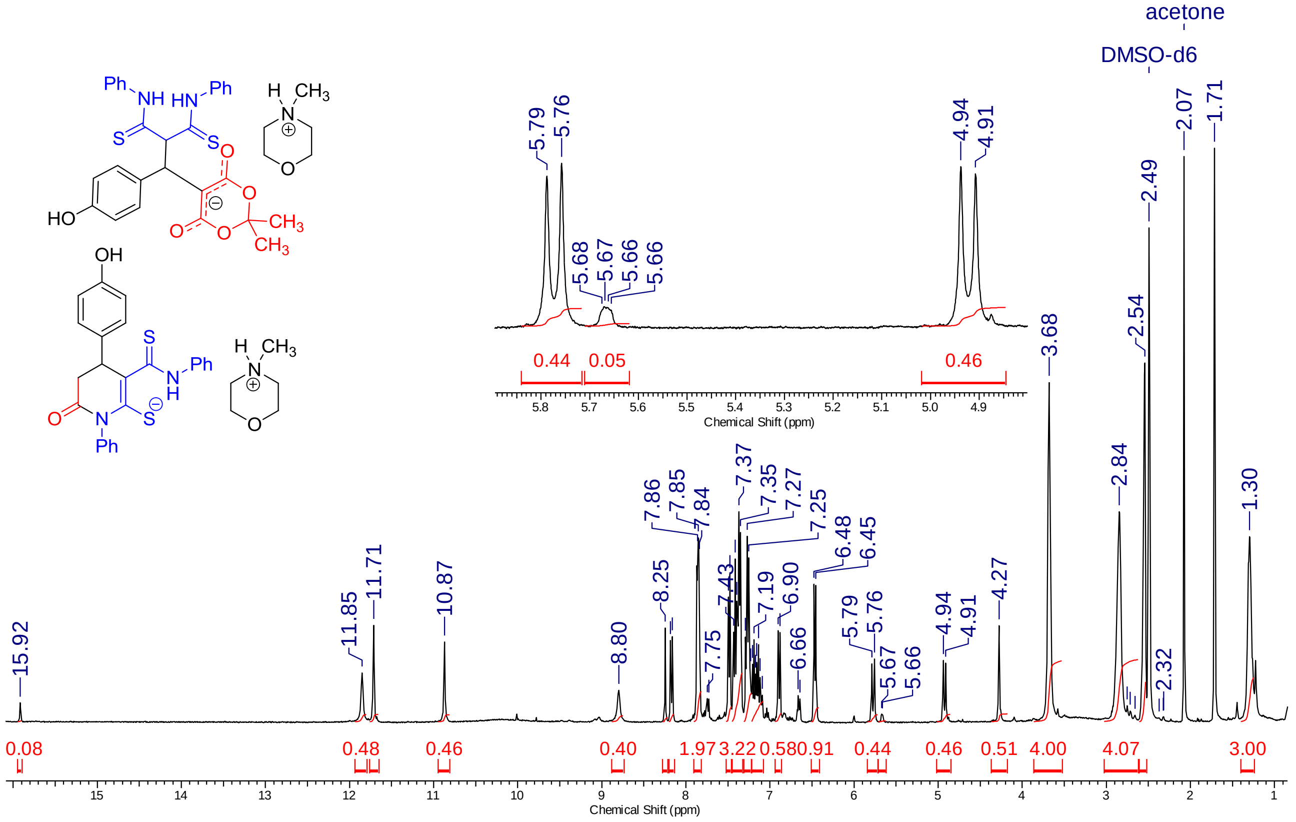

Figure S59. ^{13}C DEPTQ NMR spectrum of the products of reaction of 4-hydroxybenzylidene Meldrum's acid 14i with dithiomalondianilide 1, DMSO-d$_6$ (101 MHz) (Table 1, entry 18)

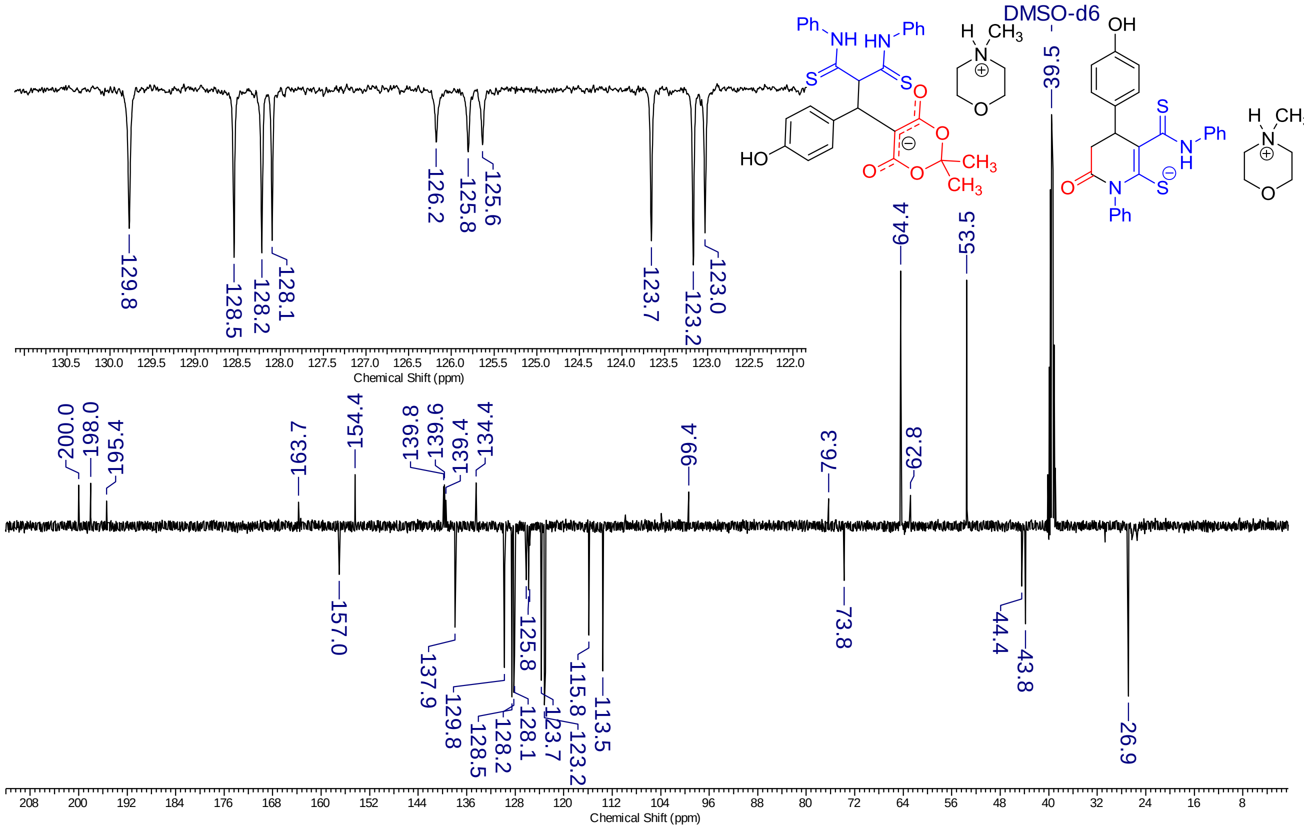

Figure S60. ^{1}H–^{13}C **HSQC NMR spectrum of the products of reaction of 4-hydroxybenzylidene Meldrum's acid 14i with dithiomalondianilide 1, DMSO-d_6 (400/101 MHz) (Table 1, entry 18)**

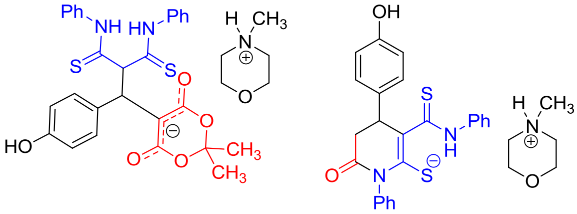

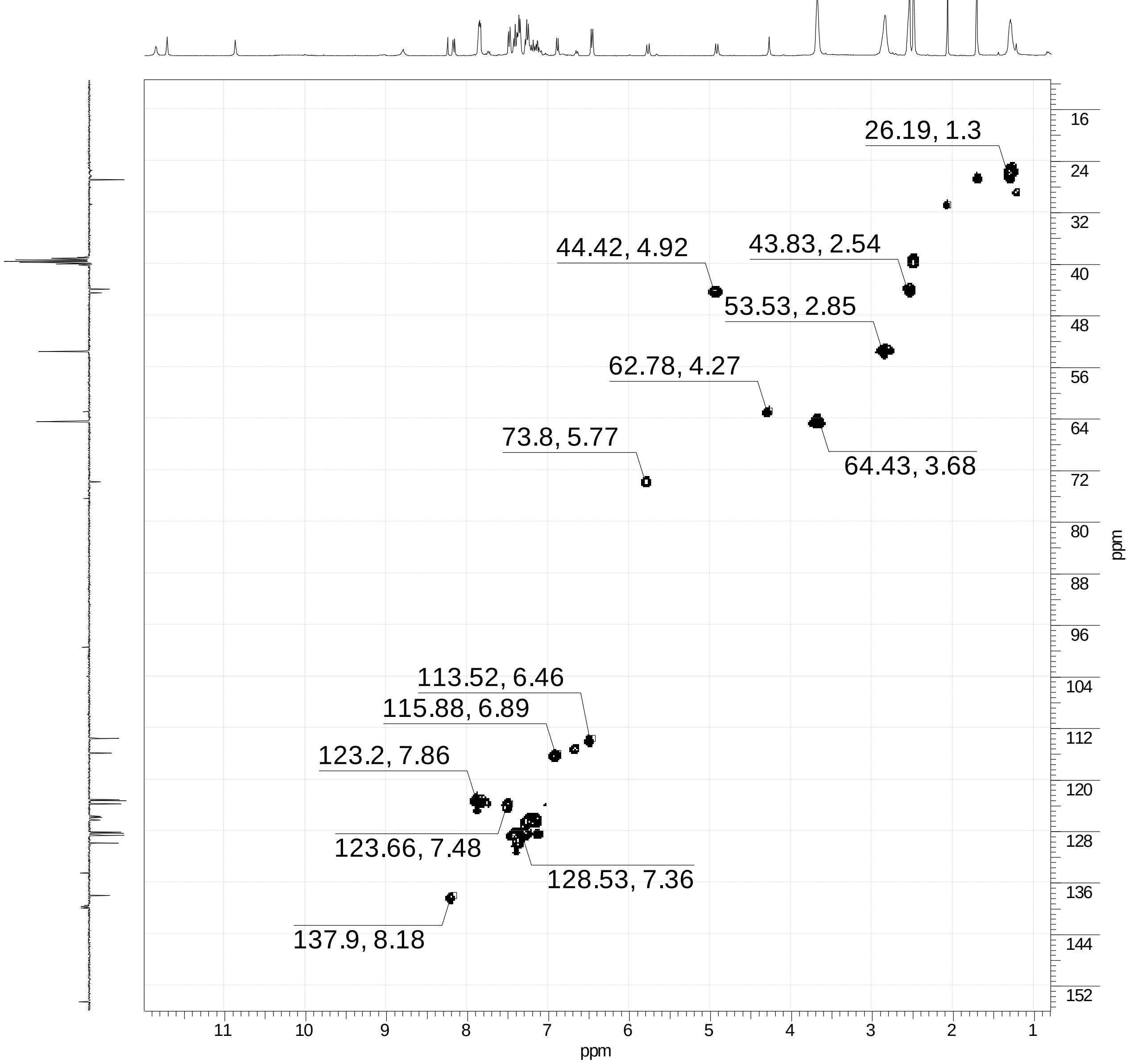

Figure S61. ^{1}H–^{13}C **HSQC NMR spectrum of the products of reaction of 4-hydroxybenzylidene Meldrum's acid 14i with dithiomalondianilide 1, DMSO-d$_6$ (400/101 MHz) (Table 1, entry 18) (*fragments*)**

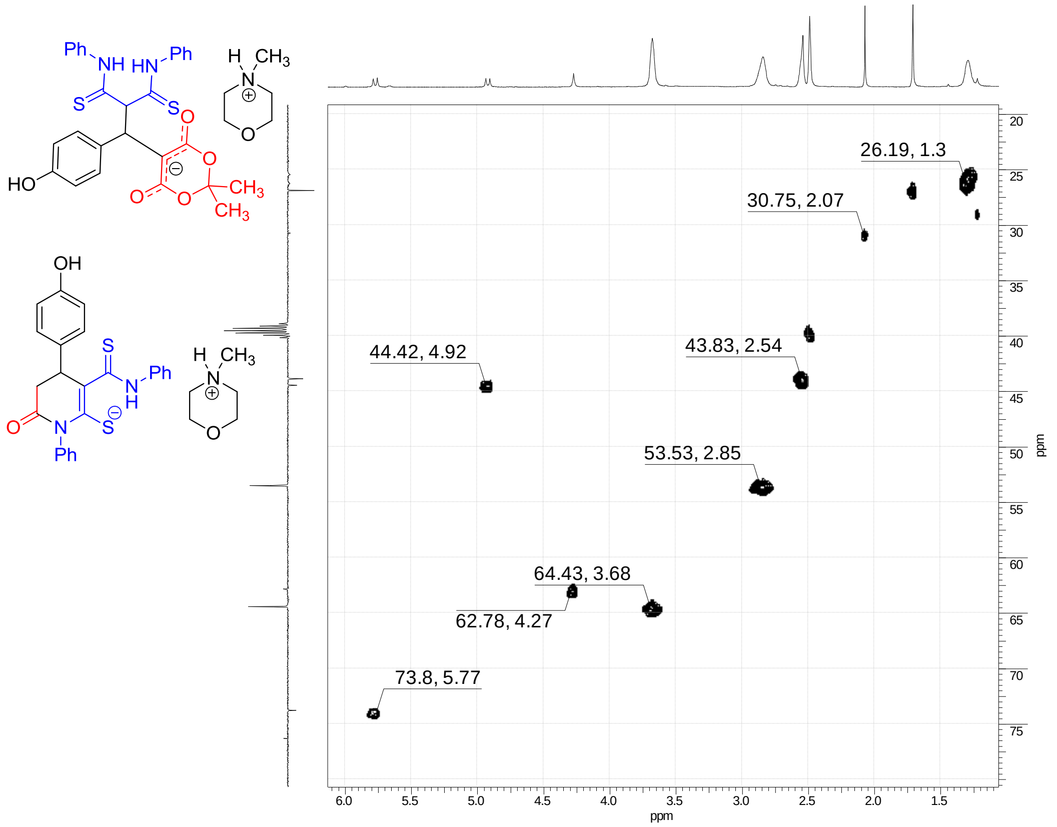

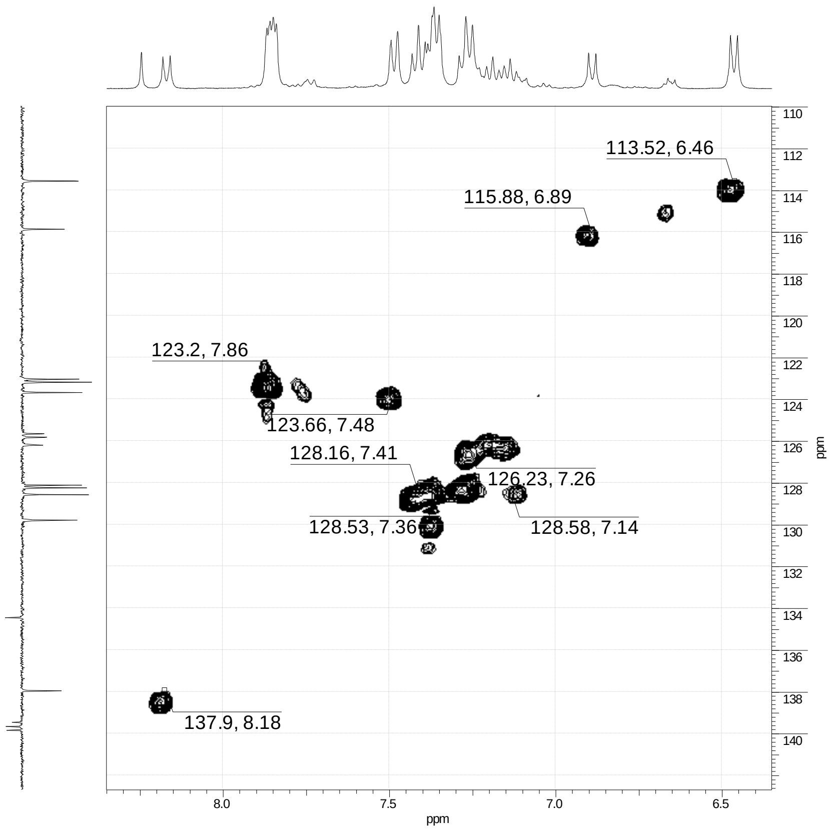

Figure S62. ^{1}H–^{13}C HMBC NMR spectrum of the products of reaction of 4-hydroxybenzylidene Meldrum's acid 14i with dithiomalondianilide 1, DMSO-d_6 (400/101 MHz) (Table 1, entry 18)

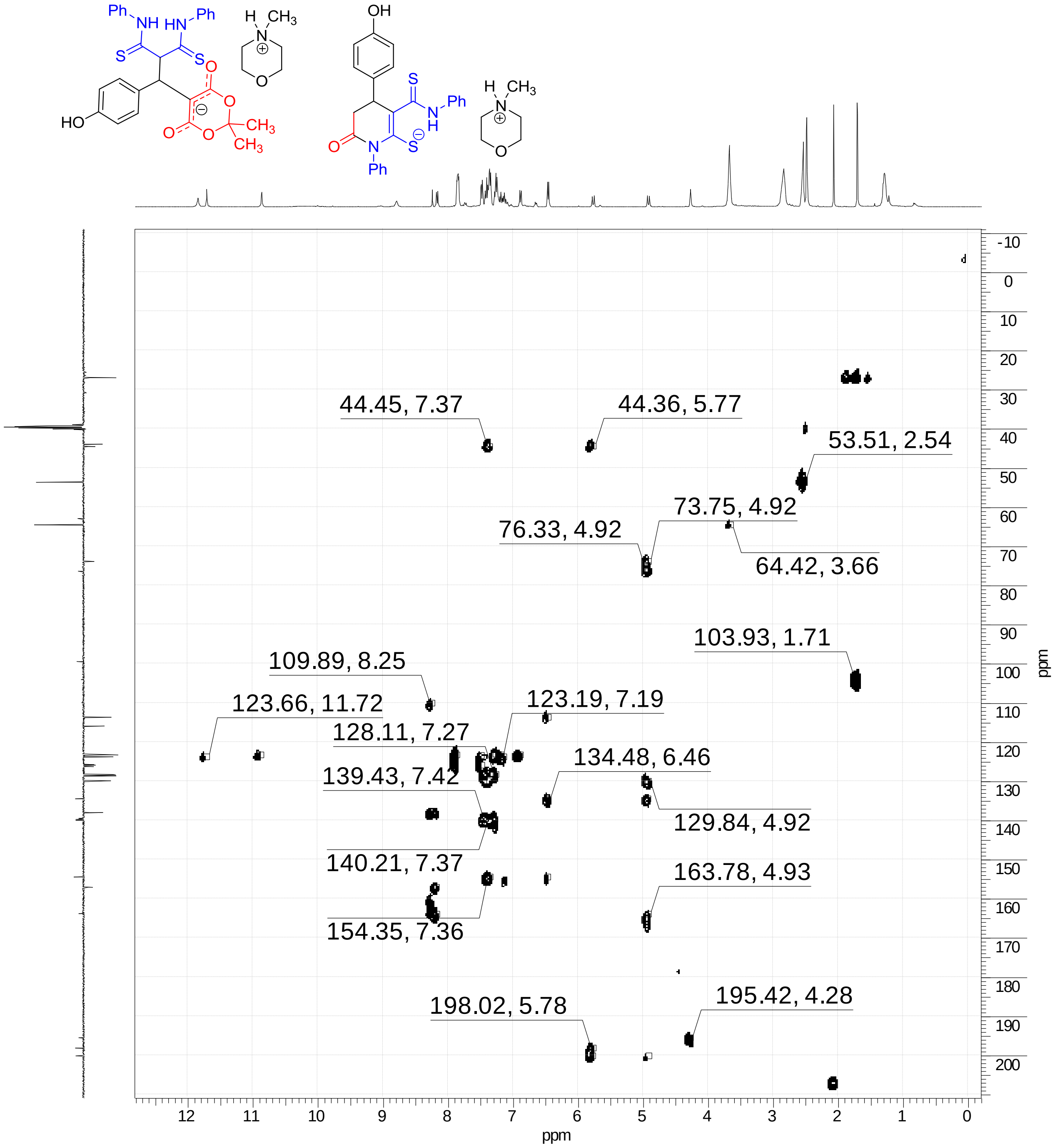

Figure S63. ^{1}H–^{13}C HMBC NMR spectrum of the products of reaction of 4-hydroxybenzylidene Meldrum's acid 14i with dithiomalondianilide 1, DMSO-d_6 (400/101 MHz) (Table 1, entry 18) (*fragments*)

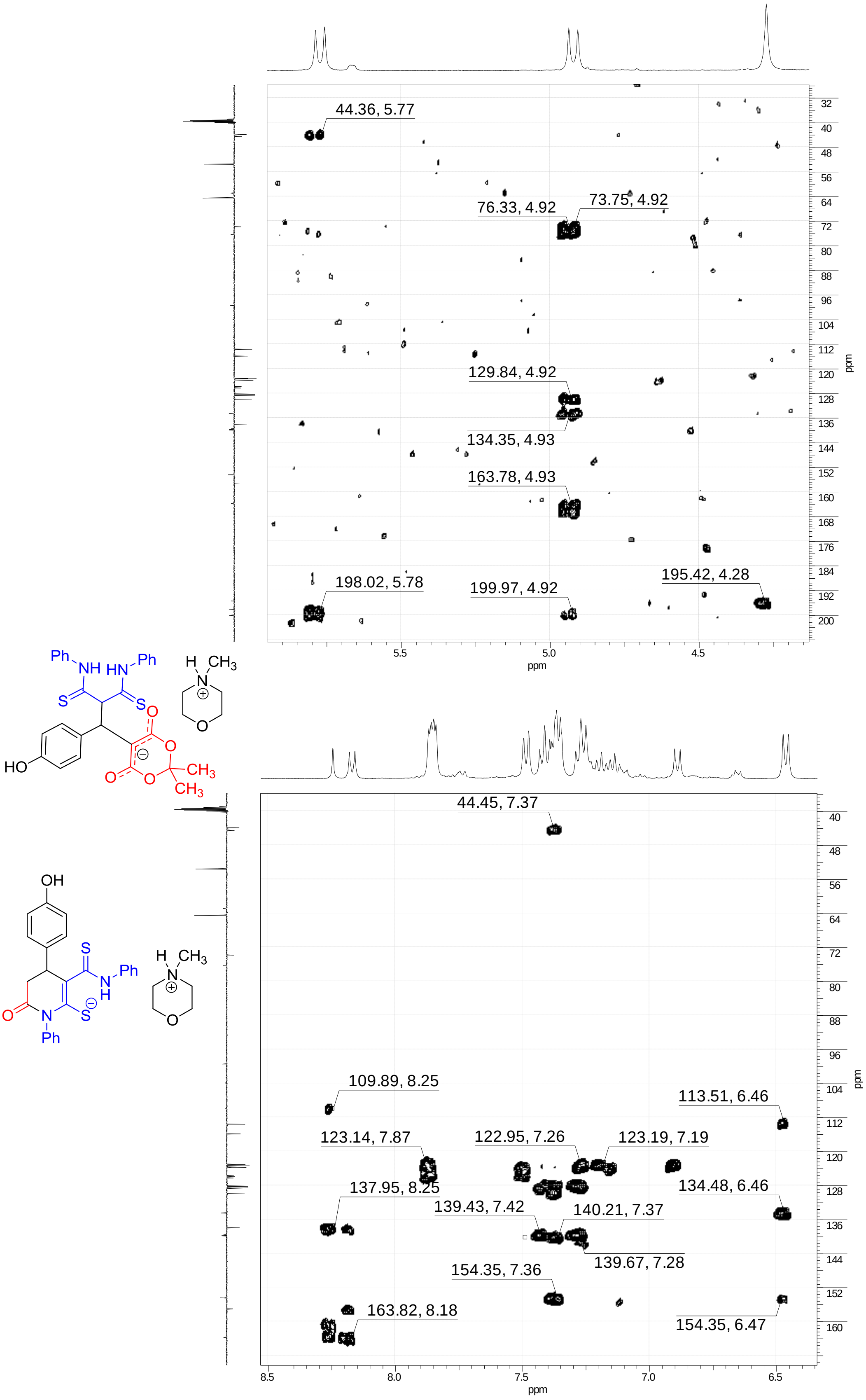

Figure S64. ^{1}**H NMR spectrum of 4-(4-(dimethylamino)phenyl)-7-phenyl-3-(phenylimino)-4,5-dihydro-3H-[1,2]dithiolo[3,4-b]pyridin-6(7H)-one 17j, DMSO-d$_6$ (400 MHz) (Table 1, entry 19)**

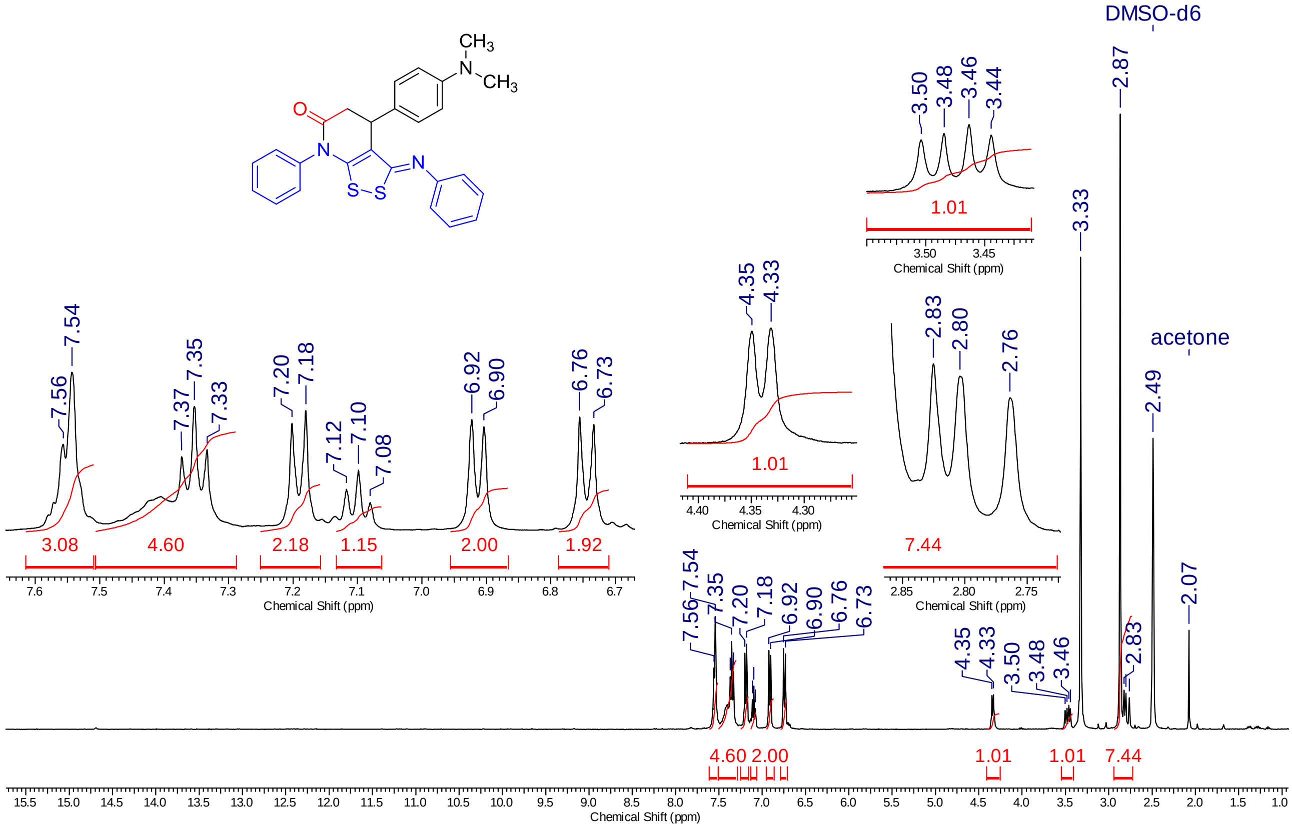

Figure S65. ^{13}**C DEPTQ NMR spectrum of 4-(4-(dimethylamino)phenyl)-7-phenyl-3-(phenylimino)-4,5-dihydro-3H-[1,2]dithiolo[3,4-b]pyridin-6(7H)-one 17j, DMSO-d$_6$ (101 MHz) (Table 1, entry 19)**

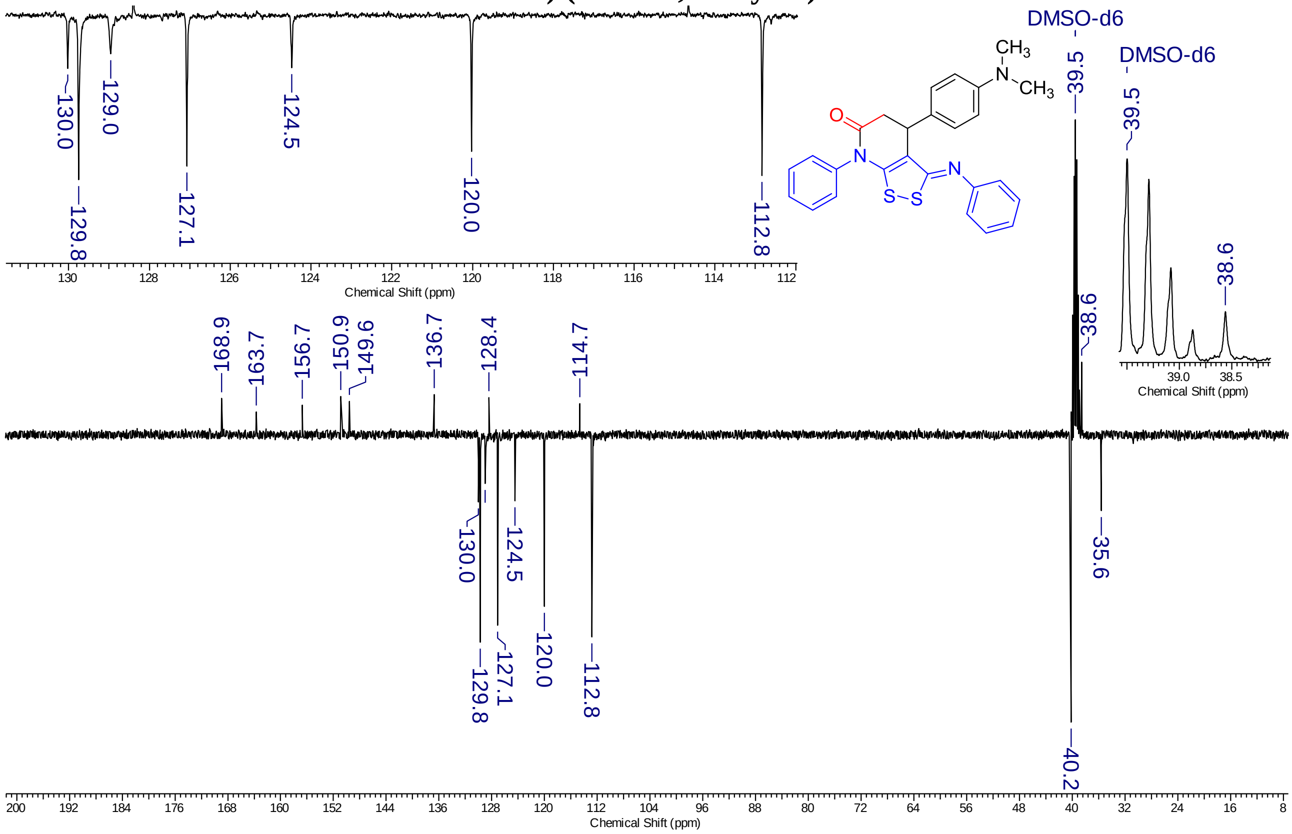

Figure S66. ^{1}H–^{13}C **HSQC NMR spectrum of 4-(4-(dimethylamino)phenyl)-7-phenyl-3-(phenylimino)-4,5-dihydro-3H-[1,2]dithiolo[3,4-b]pyridin-6(7H)-one 17j, DMSO-d_6 (400/101 MHz) (Table 1, entry 19)**

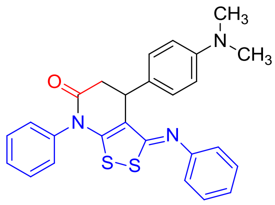

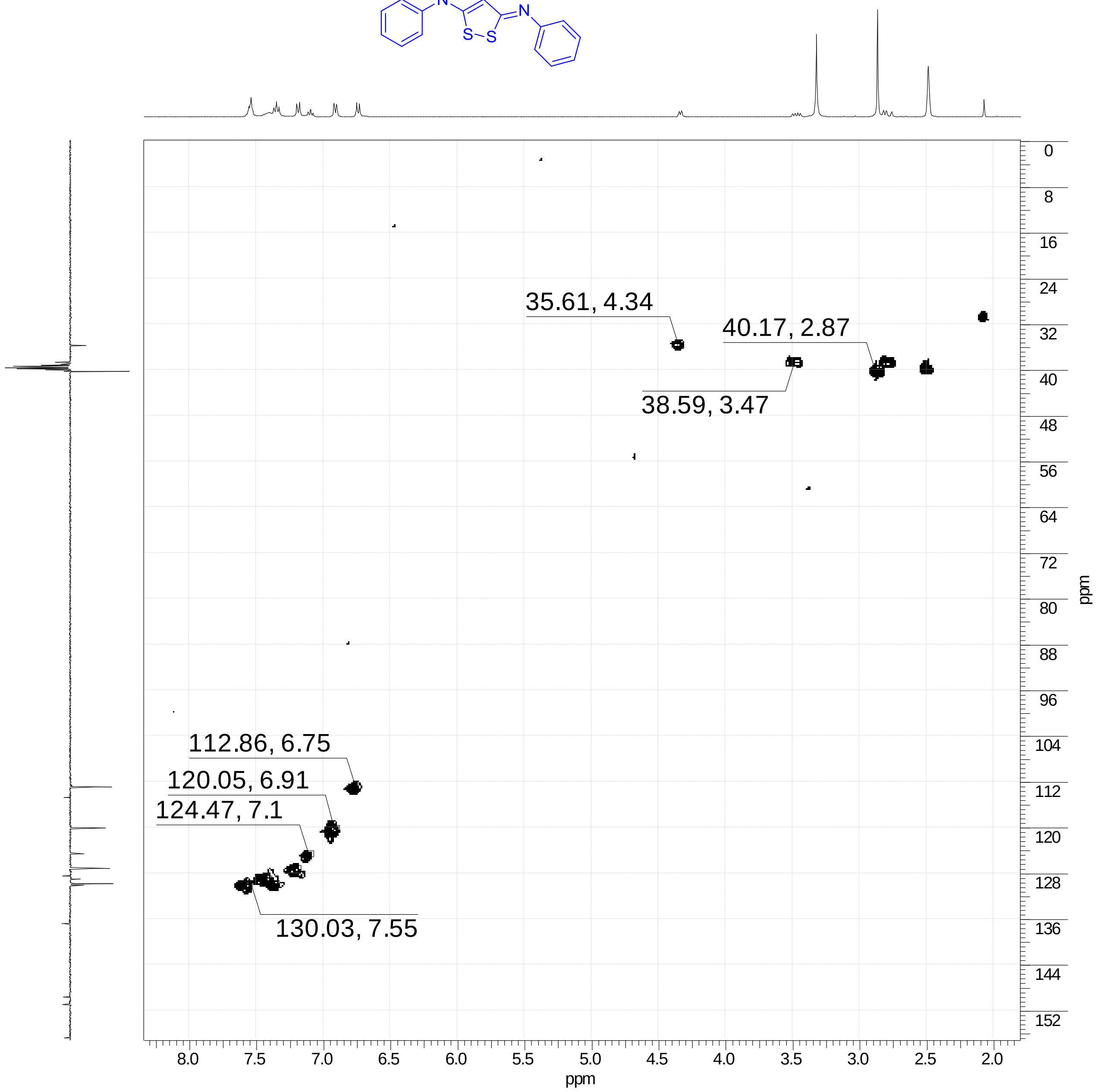

Figure S67. ^{1}H–^{13}C HSQC NMR spectrum of 4-(4-(dimethylamino)phenyl)-7-phenyl-3-(phenylimino)-4,5-dihydro-3H-[1,2]dithiolo[3,4-b]pyridin-6(7H)-one 17j, DMSO-d_6 (400/101 MHz) (Table 1, entry 19) (*fragments*)

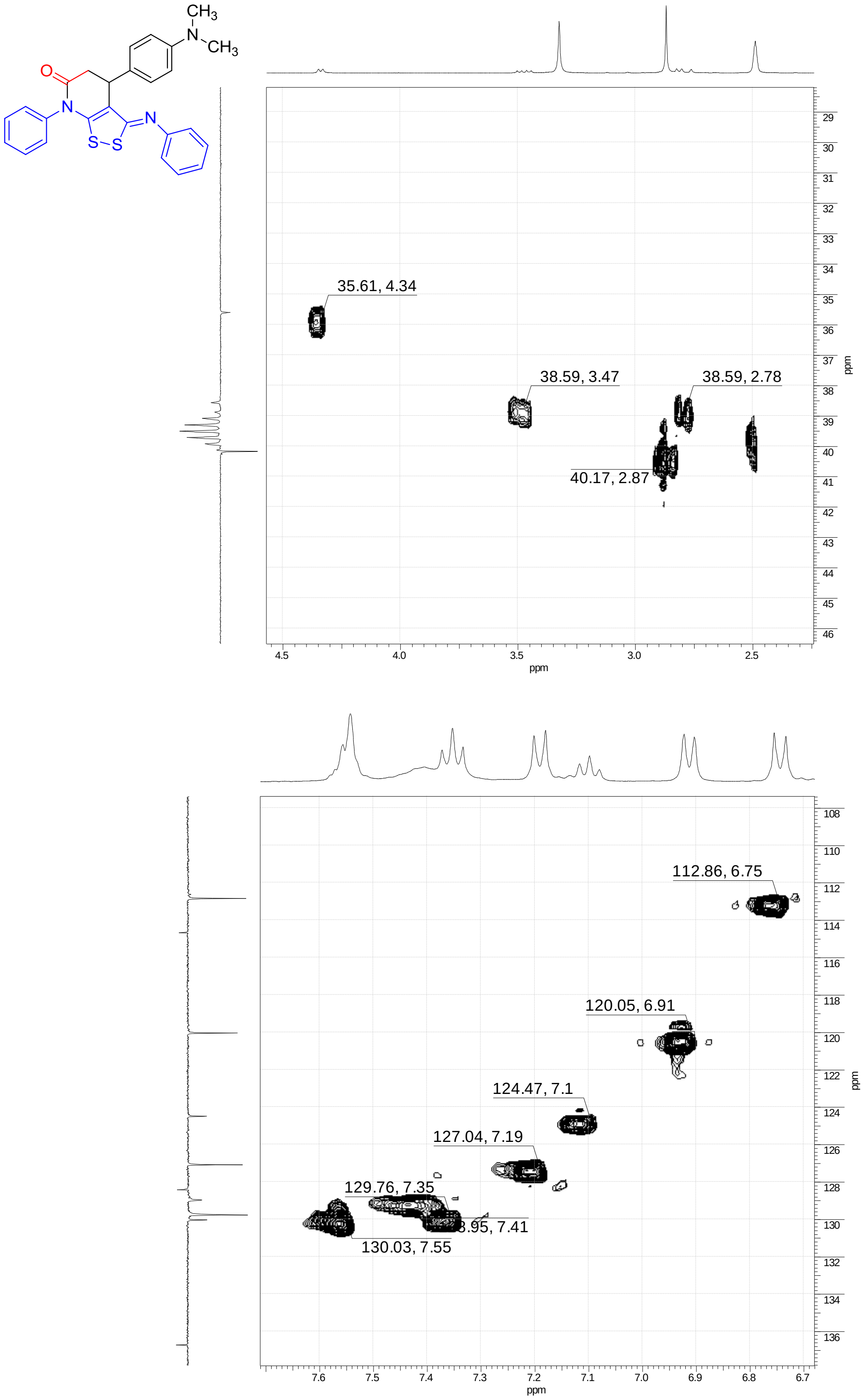

Figure S68. ^{1}H–^{13}C HMBC NMR spectrum of 4-(4-(dimethylamino)phenyl)-7-phenyl-3-(phenylimino)-4,5-dihydro-3H-[1,2]dithiolo[3,4-b]pyridin-6(7H)-one 17j, DMSO-d_6 (400/101 MHz) (Table 1, entry 19)

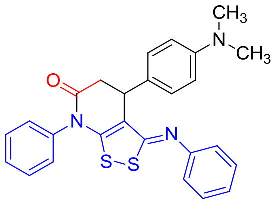

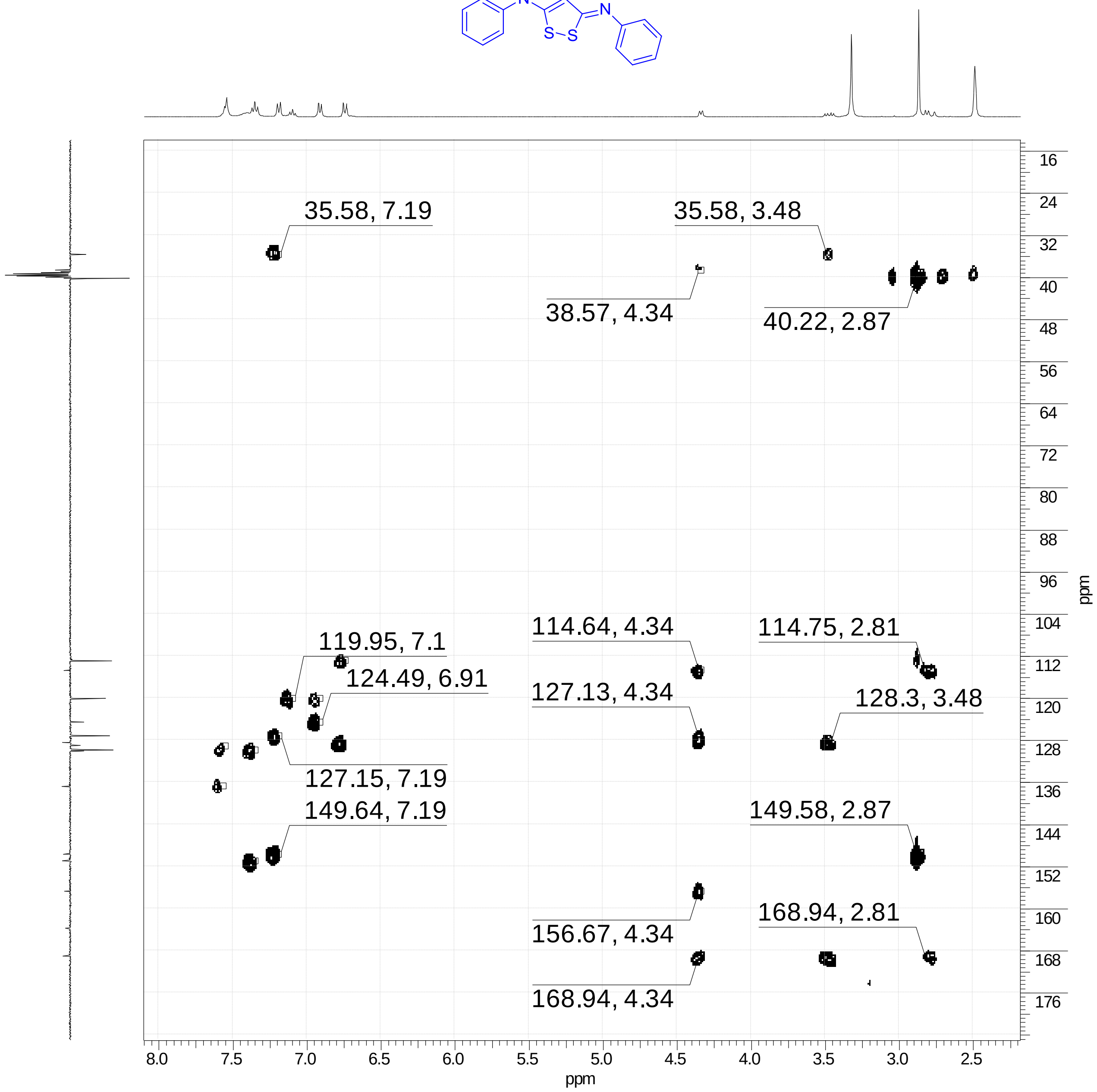

Figure S69. ^{1}H–^{13}C **HMBC NMR spectrum of 4-(4-(dimethylamino)phenyl)-7-phenyl-3-(phenylimino)-4,5-dihydro-3H-[1,2]dithiolo[3,4-b]pyridin-6(7H)-one 17j, DMSO-d_6 (400/101 MHz) (Table 1, entry 19) (*fragments*)**

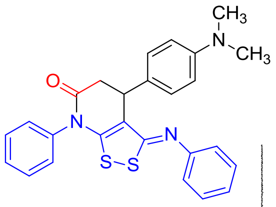

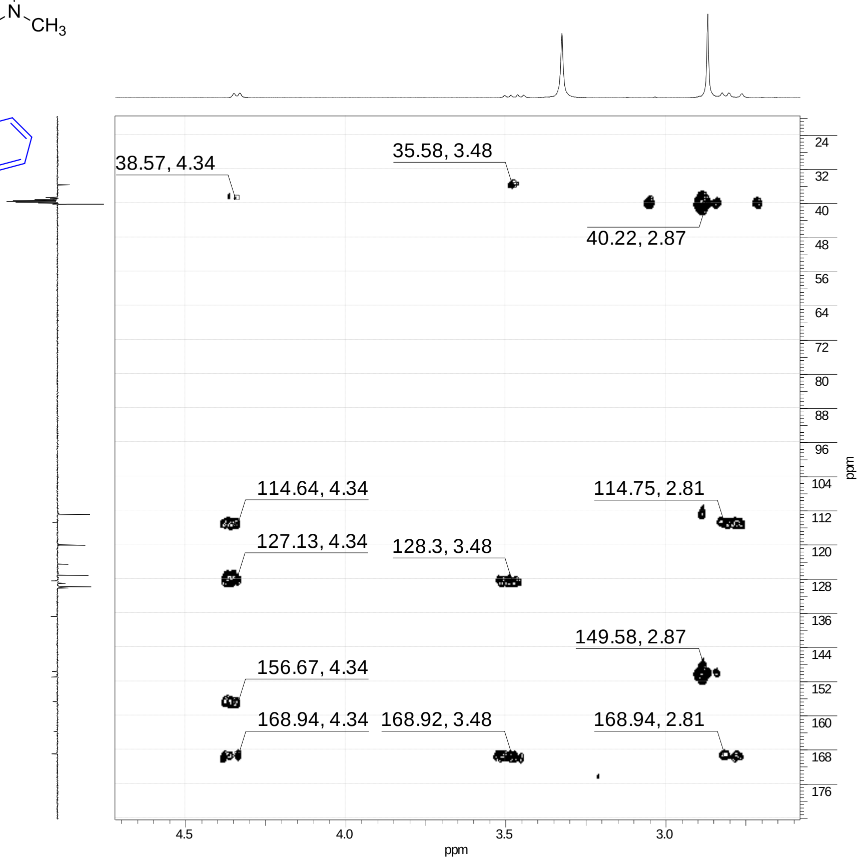

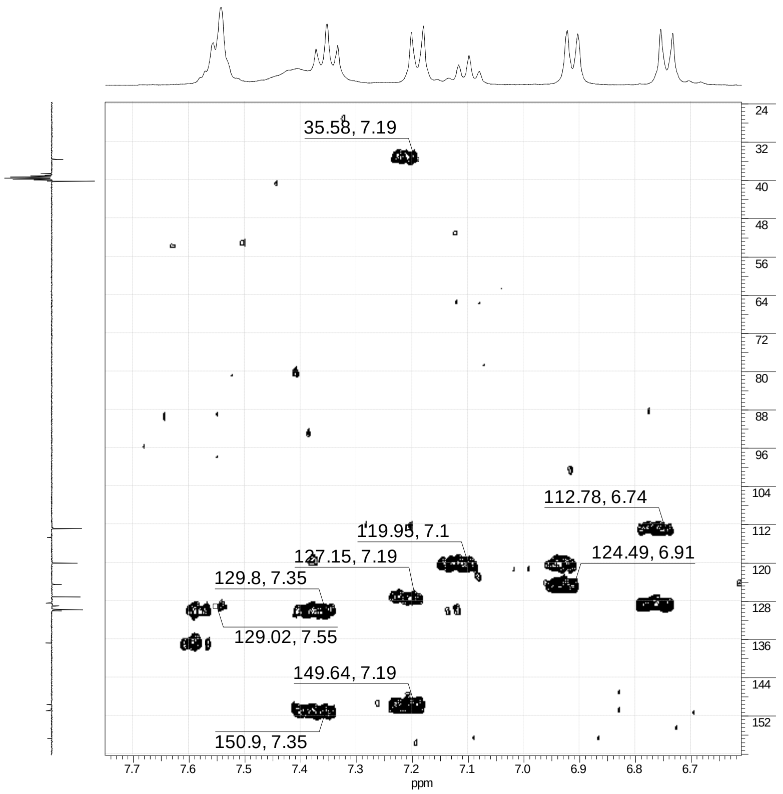

Table S4. The observed correlations in the ^{1}H-^{13}C HSQC and ^{1}H-^{13}C HMBC 2D NMR spectra of [1,2]dithiolo[3,4-b]pyridine 17j (Table 1, entry 19)

^{13}C chemical shifts are given in **red**, ^{1}H shifts – in **blue**

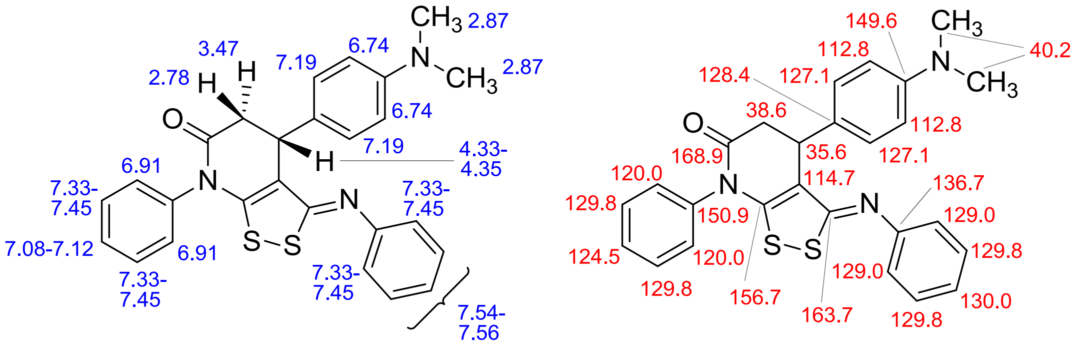

^{1}H NMR shifts, δ, ppm	Correlations in HSQC spectrum, δ, ppm	Correlations in HMBC spectrum, δ, ppm
2.78 (br d, ^{2}J = 16.0 Hz, 1H, *cis* H-5)	38.6 (C-5)	114.7 (C-3a), 168.9 (C=O)
2.87 (s, 6H, NMe_2)	40.2* (NMe_2)	40.2* (NMe_2), 112.8* (C-3, C-5 4-$Me_2NC_6H_4$) (weak), 149.6 ($\underline{C}$-NMe_2)
3.47 (dd, ^{2}J = 16.0 Hz, ^{3}J = 7.7 Hz, 1H, *trans* H-5)	38.6 (C-5)	35.6* (C-4), 128.4 (C-1 4-$Me_2NC_6H_4$), 168.9 (C=O)
4.33-4.35 (m, 1H, H-4)	35.6* (C-4)	38.6 (C-5), 114.7 (C-3a), 127.1* (C-2, C-6 4-$Me_2NC_6H_4$), 156.7 (C-7a), 163.7 ($\underline{C}$=N–Ph), 168.9 (C=O)
6.74 (d, ^{3}J = 8.7 Hz, 2H, H-3, H-5 4-$Me_2NC_6H_4$)	112.8* (C-3, C-5 4-$Me_2NC_6H_4$)	112.8* (C-3, C-5 4-$Me_2NC_6H_4$), 128.4 (C-1 4-$Me_2NC_6H_4$)
6.91 (d, ^{3}J = 7.5 Hz, 2H, H-2, H-6 N(7)Ph)	120.0* (C-2, C-6 PhN(7))	120.0* (C-2, C-6 PhN(7)), 124.5* (C-4 PhN(7))
7.08-7.12 (m, 1H, H-4 N(7)Ph)	124.5* (C-4 PhN(7))	120.0* (C-2, C-6 PhN(7))
7.19 (d, ^{3}J = 8.7 Hz, 2H, H-2, H-6 4-$Me_2NC_6H_4$)	127.1* (C-2, C-6 4-$Me_2NC_6H_4$)	127.1* (C-2, C-6 4-$Me_2NC_6H_4$), 149.6 ($\underline{C}$-NMe_2)
7.33-7.45 (m, 4H, Ph)	129.0* (C-2, C-6 =N-Ph), 129.8* (C-3, C-5 PhN(7))	120.0* (C-2, C-6 PhN(7)) (weak), 129.8, 150.9 (C-1 PhN(7))
7.54-7.56 (m, 3H, Ph)	129.8* (C-3, C-5 =N-Ph), 130.0* (C-4 =N-Ph)	129.0* (C-2, C-6 =N-Ph), 136.7 (C-1 =N-Ph)

*Signals with a negative phase.

Figure S70. ^{1}H NMR spectrum of 4-(2-chlorophenyl)-7-phenyl-3-(phenylimino)-4,5-dihydro-3H-[1,2]dithiolo[3,4-b]pyridin-6(7H)-one 17c, DMSO-d_6 (400 MHz)

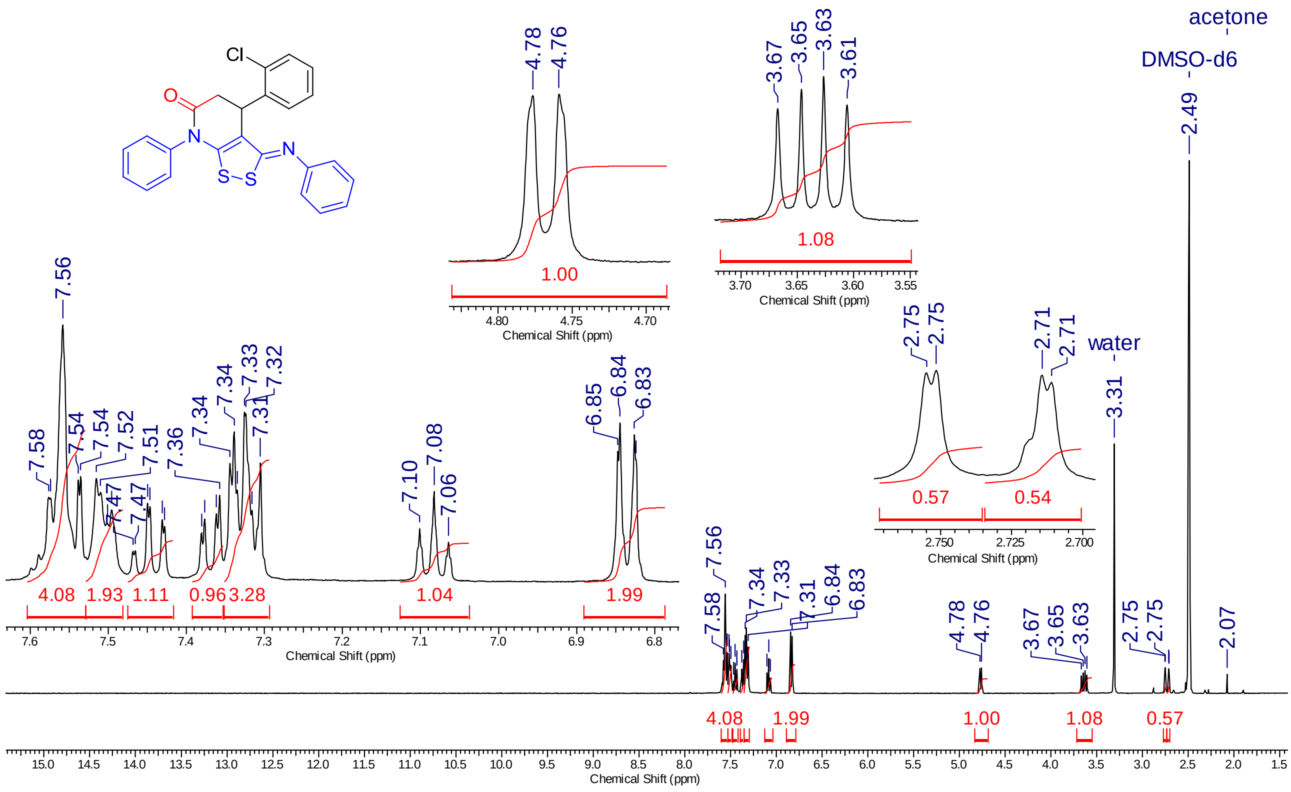

Figure S71. ^{13}C DEPTQ NMR spectrum of 4-(2-chlorophenyl)-7-phenyl-3-(phenylimino)-4,5-dihydro-3H-[1,2]dithiolo[3,4-b]pyridin-6(7H)-one 17c, DMSO-d_6 (101 MHz)

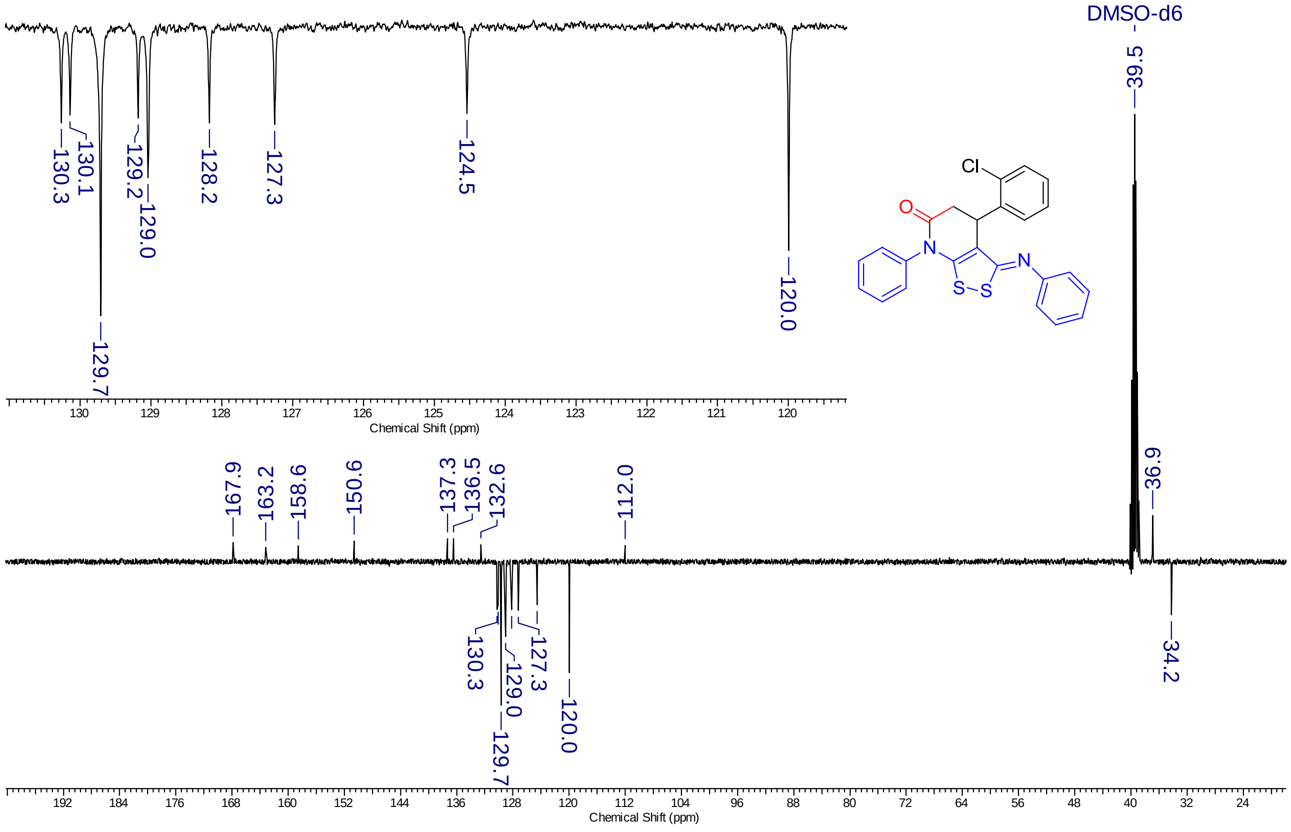

Figure S72. FTIR spectrum of 4-(2-chlorophenyl)-7-phenyl-3-(phenylimino)-4,5-dihydro-3H-[1,2]dithiolo[3,4-b]pyridin-6(7H)-one 17c (ATR mode)

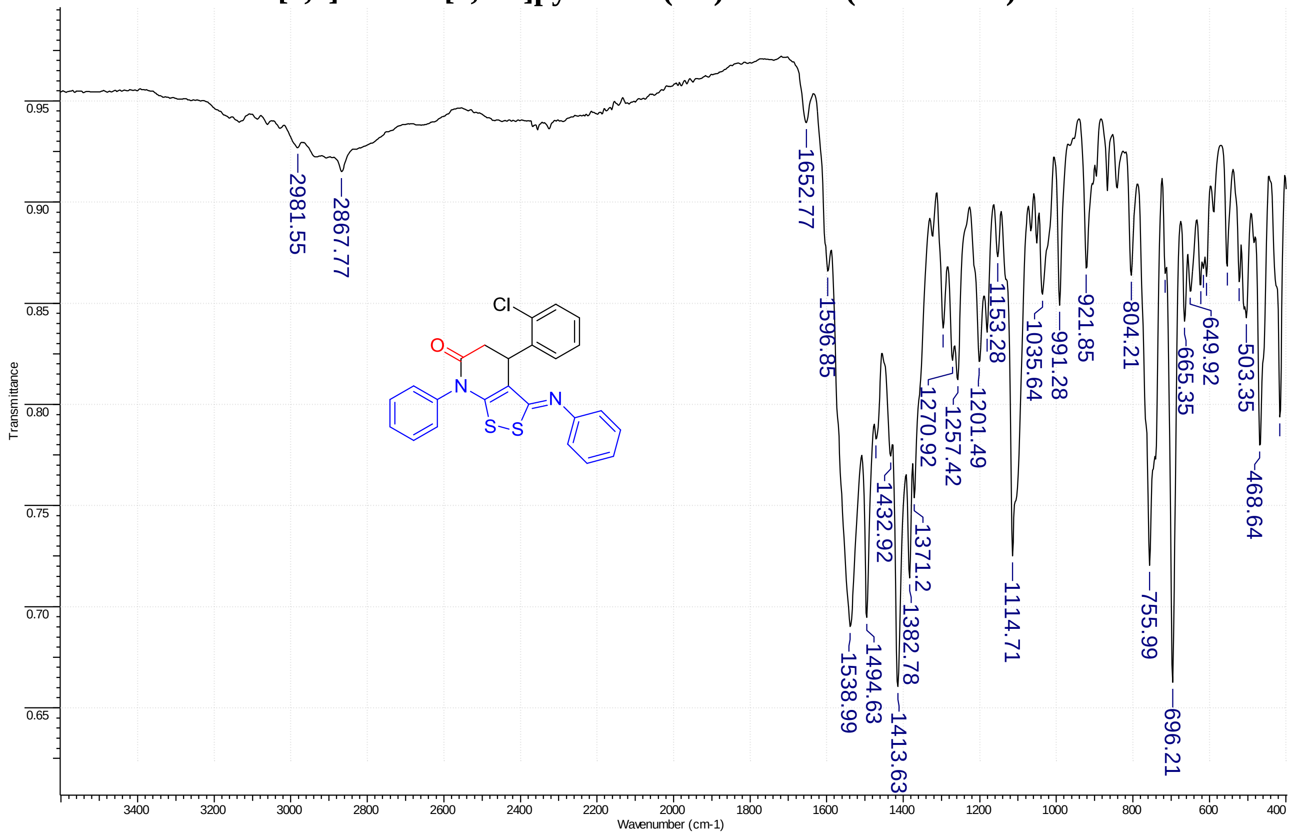

Figure S73. ORTEP drawings of the crystal structure showing 50% probability thermal ellipsoids (CCDC 2218133) and microphotography of the single crystal of compound 15b used for X-Ray diffraction analysis at the bottom.

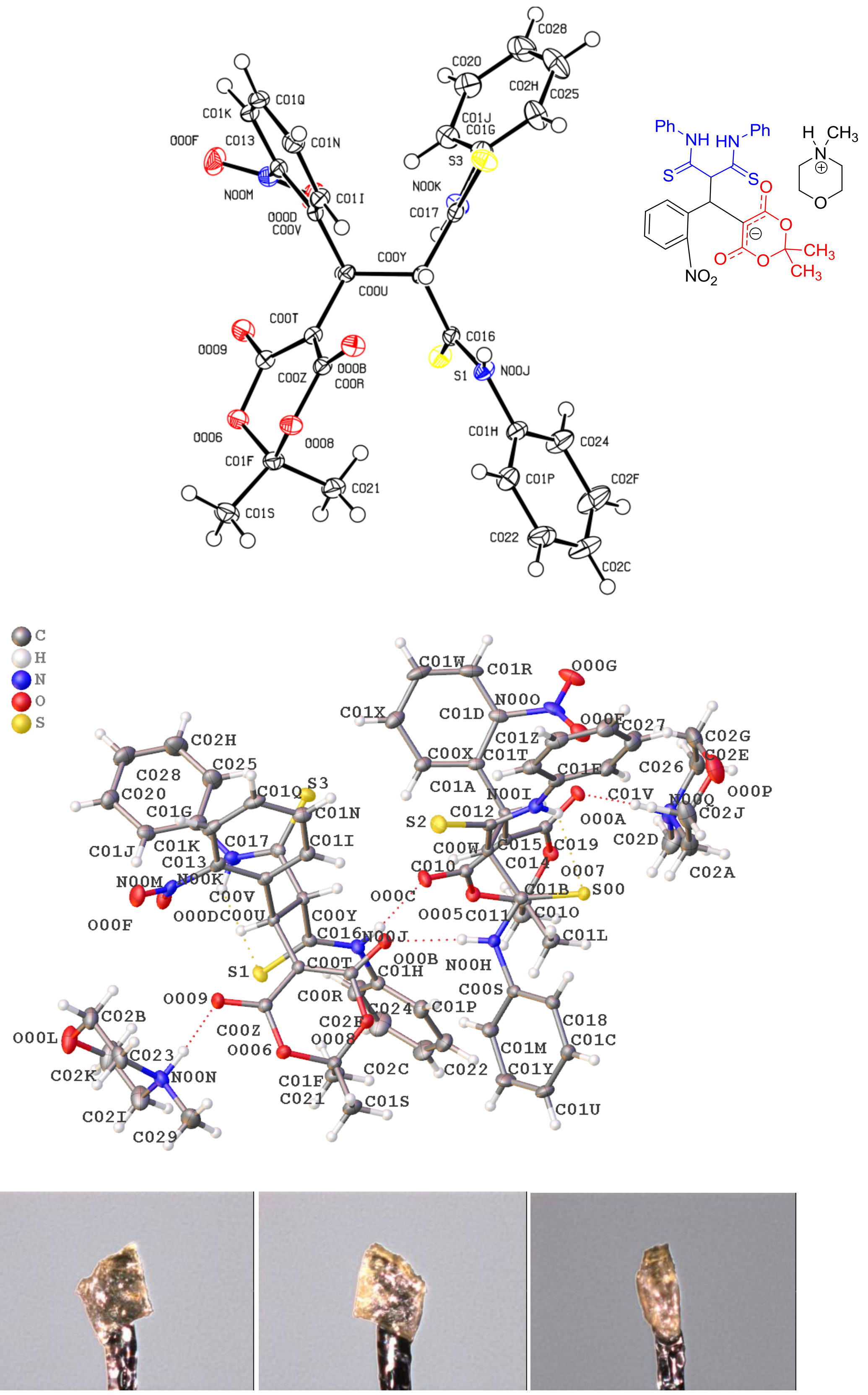

Table S5. Crystal data and structure refinement for N-methylmorpholinium 2,2-dimethyl-5-(1-(2-nitrophenyl)-3-(phenylamino)-2-(N-phenylthiocarbamoyl)-3-thioxopropyl)-4-oxo-4H-1,3-dioxin-6-olate 15b

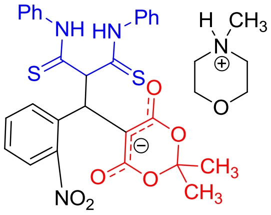

Crystal data and structure refinement for **15b**	
Identification code	ANNA_SAE52_2
Empirical formula	$C_{33}H_{36}N_4O_7S_2$
Formula weight	664.78
Temperature/K	100.01(11)
Crystal system	monoclinic
Space group	$P2_1/n$
a/Å	14.39109(7)
b/Å	22.46449(13)
c/Å	20.81473(12)
α/°	90
β/°	98.9856(5)
γ/°	90
Volume/Å^3	6646.58(7)
Z	8
ρ_{calc}mg/mm^3	1.329
m/mm^{-1}	1.896
F(000)	2800.0
Crystal size/mm^3	0.359 × 0.294 × 0.164
2Θ range for data collection	7.36 to 152.14°
Index ranges	$-17 \le h \le 12$, $-28 \le k \le 27$, $-25 \le l \le 26$
Reflections collected	51297
Independent reflections	13563[R(int) = 0.0349]
Data/restraints/parameters	13563/0/819
Goodness-of-fit on F^2	1.023
Final R indexes [I>=2σ (I)]	$R_1 = 0.0336$, $wR_2 = 0.0833$
Final R indexes [all data]	$R_1 = 0.0416$, $wR_2 = 0.0882$
Largest diff. peak/hole / e Å^{-3}	0.46/-0.38

Table S6. Fractional Atomic Coordinates (×10^4) and Equivalent Isotropic Displacement Parameters (Å^2×10^3) for adduct 15b. U_{eq} is defined as 1/3 of of the trace of the orthogonalised U_{IJ} tensor.

Fractional Atomic Coordinates (×10^4) and Equivalent Isotropic Displacement Parameters (Å^2×10^3) for 15b. U_{eq} is defined as 1/3 of of the trace of the orthogonalised U_{IJ} tensor.

Atom	*x*	*y*	*z*	U(eq)
S00	5385.2(3)	2509.8(2)	2857.2(2)	18.77(8)
S1	1884.8(2)	1948.8(2)	6363.6(2)	19.36(8)
S2	5964.0(3)	3982.6(2)	4584.0(2)	22.76(8)
S3	1943.9(3)	3922.2(2)	5246.1(2)	23.48(8)
O005	2168.9(7)	2194.0(4)	3528.8(5)	17.2(2)
O006	5107.8(7)	1647.5(5)	7022.0(5)	19.0(2)
O007	2073.9(7)	2380.0(5)	2413.8(5)	17.7(2)
O008	5228.0(7)	1740.5(5)	5919.3(5)	18.2(2)
O009	4391.7(7)	2334.9(5)	7527.0(5)	21.1(2)
O00A	2791.4(7)	3151.8(5)	2052.1(5)	18.2(2)
O00B	4583.5(7)	2483.4(5)	5306.7(5)	20.1(2)
O00C	3040.4(7)	2743.4(5)	4275.8(5)	20.6(2)
O00D	2883.6(7)	3415.9(5)	7551.4(5)	25.7(2)
O00E	4286.9(8)	4191.4(5)	2170.2(6)	27.0(2)
O00F	4096.2(8)	3807.2(6)	8146.0(5)	28.8(3)
O00G	2932.5(8)	4582.3(6)	1805.1(6)	32.6(3)
N00H	5299.8(8)	2251.0(5)	4102.3(6)	15.2(2)
N00I	5845.7(8)	3825.6(5)	3293.3(6)	16.9(2)
N00J	2294.4(8)	2012.0(5)	5155.5(6)	16.7(2)
N00K	1474.3(9)	3334.8(6)	6263.5(6)	20.0(2)
O00L	3599.9(9)	2232.1(7)	9320.3(6)	38.3(3)
N00M	3643.2(8)	3678.2(6)	7614.1(6)	19.7(3)
N00N	4935.0(9)	1728.3(6)	8590.8(6)	22.4(3)
N00O	3525.7(9)	4399.6(6)	2251.4(7)	23.1(3)
O00P	4156.2(10)	3246.0(8)	569.3(6)	43.7(3)
N00Q	2671.3(9)	2554.4(7)	945.7(6)	24.4(3)
C00R	4642.6(9)	2236.5(6)	5842.3(7)	15.7(3)
C00S	5738.4(6)	1680.0(3)	4108.6(5)	15.5(3)
C018	6549.2(6)	1584.0(3)	3837.4(4)	17.9(3)
C01C	6981.6(5)	1029.1(4)	3891.2(5)	21.5(3)
C01U	6603.2(7)	570.1(3)	4216.2(5)	25.3(3)
C01Y	5792.5(7)	666.1(3)	4487.4(5)	28.2(3)
C01M	5360.1(6)	1221.0(4)	4433.6(5)	21.7(3)
C00T	4231.8(9)	2409.2(6)	6386.0(7)	14.3(3)
C00U	3516.9(9)	2905.8(6)	6391.7(6)	13.6(3)
C00V	3960.8(9)	3519.5(6)	6469.6(7)	15.1(3)
C00W	4759.9(9)	3235.5(6)	3786.2(6)	13.8(3)
C00X	3542.3(9)	4031.8(6)	3398.6(7)	16.0(3)
C00Y	2733.5(9)	2883.2(6)	5783.4(6)	13.9(3)
C00Z	4552.7(10)	2144.7(6)	6989.8(7)	16.5(3)

C010	2826.1(9)	2642.2(6)	3689.7(7)	15.1(3)
C011	5156.6(9)	2635.0(6)	3606.1(7)	14.5(3)
C012	3909.9(9)	3416.2(6)	3264.5(6)	13.8(3)
C013	4027.1(9)	3870.2(6)	7030.7(7)	17.1(3)
C014	3142.9(9)	2946.7(6)	3173.0(6)	13.9(3)
C015	5559.0(9)	3701.4(6)	3858.1(7)	15.1(3)
C016	2287.6(9)	2261.9(6)	5734.4(7)	15.1(3)
C017	1999.9(9)	3378.4(6)	5791.1(7)	16.0(3)
C019	2693.8(9)	2844.1(6)	2537.7(7)	15.0(3)
C01A	3368.5(10)	4180.3(7)	4021.6(8)	19.9(3)
C01B	2139.7(10)	1924.2(6)	2905.3(7)	16.7(3)
C01D	3319.2(9)	4471.0(6)	2919.9(7)	19.4(3)
C01E	6541.7(6)	4204.3(4)	3101.6(4)	18.4(3)
C01T	7125.3(7)	4585.6(4)	3507.8(4)	22.3(3)
C01Z	7761.0(6)	4947.9(4)	3253.1(5)	28.3(3)
C027	7813.1(7)	4929.0(4)	2592.1(5)	31.9(4)
C026	7229.4(8)	4547.7(5)	2185.9(4)	30.9(4)
C01V	6593.7(7)	4185.4(4)	2440.7(4)	23.5(3)
C01F	5093.6(10)	1330.4(6)	6420.9(7)	18.0(3)
C01G	776.9(6)	3728.1(4)	6436.0(5)	20.7(3)
C01J	733.4(6)	3782.2(4)	7095.9(4)	22.0(3)
C020	82.2(7)	4164.3(5)	7305.0(4)	27.2(3)
C028	-525.5(7)	4492.4(5)	6854.2(5)	33.3(4)
C02H	-482.0(7)	4438.3(5)	6194.3(5)	36.7(4)
C025	169.2(7)	4056.2(5)	5985.2(4)	30.1(4)
C01H	1961.9(6)	1435.6(3)	4942.9(5)	18.3(3)
C01P	2531.4(5)	1094.5(4)	4605.5(5)	23.1(3)
C022	2218.7(7)	544.7(4)	4352.2(5)	29.5(3)
C02C	1336.4(8)	335.9(4)	4436.3(6)	35.7(4)
C02F	766.9(6)	677.0(5)	4773.8(6)	39.4(4)
C024	1079.6(6)	1226.9(5)	5027.1(6)	28.5(3)
C01I	4370.8(10)	3757.9(7)	5958.7(7)	18.4(3)
C01K	4454.6(10)	4427.3(7)	7085.2(8)	21.6(3)
C01L	3010.6(10)	1543.2(7)	2884.6(8)	21.2(3)
C01N	4768.7(10)	4320.9(7)	5994.1(8)	22.2(3)
C01O	1234.7(10)	1575.3(7)	2773.1(8)	22.5(3)
C01Q	4802(1)	4657.9(7)	6555.2(8)	23.0(3)
C01R	2950.4(10)	5022.2(7)	3046.4(9)	26.8(3)
C01S	5942.8(11)	927.6(7)	6517.7(8)	24.7(3)
C01W	2829.4(11)	5160.7(7)	3671.3(10)	29.6(4)
C01X	3036.8(11)	4739.9(7)	4161.4(9)	25.9(3)
C021	4173.6(11)	993.5(7)	6246.6(8)	23.7(3)
C023	5199.6(12)	2191.8(8)	9104.0(8)	28.6(4)
C029	5735.7(14)	1329.6(9)	8522.8(9)	37.5(4)
C02A	3660.0(11)	2329.3(9)	1007.9(8)	32.4(4)
C02B	4362.4(13)	2577.8(8)	9162.8(8)	31.7(4)

C02D	1974.8(13)	2059.5(9)	845.9(10)	36.8(4)
C02E	2498.9(12)	3002.2(9)	413.2(8)	31.1(4)
C02G	3227.3(15)	3484.8(9)	531.5(9)	38.4(4)
C02I	4091.2(14)	1394.3(9)	8724.8(10)	37.4(4)
C02J	4335.7(12)	2840.4(11)	1100.9(9)	40.3(5)
C02K	3307.2(14)	1820.4(11)	8807.0(11)	44.4(5)

Table S7. Anisotropic Displacement Parameters (Å^2×10^3) for 15b. The Anisotropic displacement factor exponent takes the form: $-2\pi^2[h^2a^{*2}U_{11}+...+2hka\times b\times U_{12}]$

Atom	U_{11}	U_{22}	U_{33}	U_{23}	U_{13}	U_{12}
S00	27.94(17)	16.01(16)	12.55(15)	-0.41(12)	3.74(13)	4.43(13)
S1	22.94(17)	19.04(17)	16.73(16)	2.55(13)	5.04(13)	-5.38(13)
S2	26.42(18)	23.19(18)	16.68(16)	-3.81(13)	-2.84(13)	-5.14(14)
S3	28.48(18)	20.09(18)	22.53(18)	6.68(14)	6.00(14)	4.35(14)
O005	19.3(5)	15.5(5)	17.4(5)	-1.4(4)	4.5(4)	-5.0(4)
O006	23.1(5)	16.8(5)	16.5(5)	-0.5(4)	1.1(4)	3.7(4)
O007	18.7(5)	17.3(5)	15.8(5)	-0.3(4)	-1.0(4)	-4.5(4)
O008	20.0(5)	16.8(5)	18.9(5)	-0.6(4)	6.3(4)	2.1(4)
O009	27.4(5)	22.6(5)	13.1(5)	-0.7(4)	2.7(4)	4.7(4)
O00A	21.9(5)	19.9(5)	12.2(4)	2.0(4)	0.5(4)	-2.8(4)
O00B	25.3(5)	20.5(5)	15.6(5)	0.5(4)	6.3(4)	1.8(4)
O00C	28.6(5)	20.9(5)	12.4(5)	-0.2(4)	4.0(4)	-7.7(4)
O00D	19.7(5)	33.8(6)	24.8(6)	-9.3(5)	7.3(4)	-2.4(4)
O00E	23.1(5)	31.7(6)	26.3(6)	9.3(5)	4.3(4)	-3.2(5)
O00F	29.5(6)	36.8(7)	18.3(5)	-9.6(5)	-1.8(4)	1.2(5)
O00G	29.7(6)	31.1(6)	32.3(6)	17.4(5)	-10.1(5)	-5.9(5)
N00H	18.9(5)	13.5(6)	13.5(5)	-0.2(4)	3.7(4)	1.1(4)
N00I	16.4(5)	15.6(6)	18.4(6)	-3.6(5)	2.2(4)	-2.8(4)
N00J	20.3(6)	15.0(6)	15.0(6)	-0.4(4)	3.2(4)	-4.8(5)
N00K	18.1(6)	19.2(6)	23.3(6)	4.0(5)	4.7(5)	2.7(5)
O00L	39.4(7)	46.9(8)	32.5(7)	-16.4(6)	18.0(6)	-14.5(6)
N00M	18.4(6)	20.7(6)	19.6(6)	-7.2(5)	1.2(5)	4.9(5)
N00N	27.0(6)	24.6(7)	15.2(6)	1.7(5)	2.0(5)	-2.0(5)
N00O	22.1(6)	18.9(6)	26.1(7)	11.0(5)	-2.9(5)	-7.2(5)
O00P	38.5(7)	66.9(10)	28.1(7)	3.6(6)	13.1(5)	-10.5(7)
N00Q	20.2(6)	34.3(8)	17.6(6)	-5.8(5)	-0.8(5)	3.9(5)
C00R	14.7(6)	14.1(6)	18.3(7)	-1.9(5)	2.0(5)	-1.5(5)
C00S	20.0(6)	12.3(6)	13.3(6)	-0.8(5)	-0.2(5)	-0.1(5)
C018	20.7(7)	16.9(7)	15.9(6)	1.9(5)	2.1(5)	0.0(5)
C01C	22.4(7)	22.9(7)	19.2(7)	-0.6(6)	3.2(5)	4.5(6)
C01U	32.2(8)	13.4(7)	30.4(8)	-0.1(6)	5.4(6)	6.2(6)
C01Y	36.1(9)	14.3(7)	36.0(9)	6.2(6)	11.3(7)	-1.6(6)
C01M	23.4(7)	17.5(7)	25.8(7)	1.6(6)	8.7(6)	-1.0(6)
C00T	15.5(6)	13.2(6)	14.1(6)	-1.4(5)	2.0(5)	-1.3(5)
C00U	14.5(6)	13.4(6)	12.6(6)	-2.4(5)	1.8(5)	-0.9(5)
C00V	12.0(6)	14.0(6)	18.1(7)	-2.0(5)	-1.5(5)	1.2(5)

C00W	15.7(6)	12.9(6)	12.4(6)	-1.0(5)	1.0(5)	0.0(5)
C00X	11.8(6)	12.4(6)	22.9(7)	1.8(5)	0.0(5)	-1.9(5)
C00Y	15.9(6)	12.8(6)	12.6(6)	-0.6(5)	1.4(5)	-3.8(5)
C00Z	16.7(6)	14.9(6)	17.3(7)	-1.2(5)	1.5(5)	-1.4(5)
C010	15.5(6)	12.4(6)	17.4(7)	-1.0(5)	2.7(5)	-1.1(5)
C011	15.1(6)	12.4(6)	15.3(6)	-1.0(5)	-0.1(5)	-1.7(5)
C012	15.0(6)	12.5(6)	13.5(6)	1.3(5)	0.5(5)	-1.2(5)
C013	12.9(6)	17.0(7)	20.0(7)	-3.6(5)	-0.9(5)	3.2(5)
C014	15.8(6)	11.9(6)	13.9(6)	0.5(5)	2.1(5)	-1.1(5)
C015	14.3(6)	11.8(6)	18.2(6)	-0.6(5)	-0.5(5)	2.8(5)
C016	14.6(6)	14.6(6)	15.3(6)	1.4(5)	-0.1(5)	-2.0(5)
C017	15.5(6)	15.3(6)	15.7(6)	-1.4(5)	-2.0(5)	-3.3(5)
C019	14.3(6)	13.7(6)	16.8(6)	-1.4(5)	2.3(5)	0.1(5)
C01A	18.3(6)	15.5(7)	26.6(7)	-0.5(6)	5.0(5)	-1.0(5)
C01B	20.1(7)	13.9(6)	15.9(6)	-1.2(5)	1.7(5)	-3.2(5)
C01D	13.7(6)	15.7(7)	27.6(8)	5.1(6)	-0.7(5)	-2.9(5)
C01E	13.9(6)	13.5(6)	28.8(8)	-0.8(5)	6.1(5)	1.1(5)
C01T	17.9(7)	16.8(7)	31.3(8)	-1.5(6)	0.7(6)	-1.1(5)
C01Z	18.0(7)	19.1(7)	46.8(10)	-2.3(7)	2.5(7)	-3.5(6)
C027	25.3(8)	22.5(8)	51.6(11)	0.4(7)	17.6(7)	-4.9(6)
C026	32.1(8)	26.8(8)	38.6(9)	-2.9(7)	20.2(7)	-4.1(7)
C01V	23.7(7)	18.4(7)	30.8(8)	-5.8(6)	11.8(6)	-2.5(6)
C01F	21.6(7)	15.0(7)	17.4(7)	-1.3(5)	3.0(5)	1.2(5)
C01G	14.9(6)	19.0(7)	29.1(8)	1.3(6)	5.8(6)	-0.7(5)
C01J	16.9(6)	21.4(7)	28.7(8)	4.8(6)	6.8(6)	-1.8(5)
C020	21.5(7)	28.0(8)	34.3(9)	-2.1(7)	11.3(6)	-0.7(6)
C028	21.7(8)	31.3(9)	48.8(11)	-1.4(8)	11.4(7)	6.3(7)
C02H	21.8(8)	42.5(11)	45.6(11)	10.7(9)	5.0(7)	12.5(7)
C025	20.6(7)	39.1(10)	30.2(8)	4.4(7)	2.6(6)	6.8(7)
C01H	23.5(7)	14.6(7)	16.3(6)	-1.0(5)	1.6(5)	-3.1(5)
C01P	24.7(7)	24.2(8)	20.5(7)	-3.9(6)	3.7(6)	-2.5(6)
C022	40.8(9)	22.1(8)	25.9(8)	-6.8(6)	5.9(7)	1.6(7)
C02C	50.7(11)	18.8(8)	38.3(10)	-10.2(7)	9.0(8)	-11.8(7)
C02F	40.1(10)	31.1(10)	49.7(11)	-12.8(8)	15.1(9)	-19.3(8)
C024	27.5(8)	23.8(8)	36.1(9)	-9.8(7)	10.7(7)	-7.9(6)
C01I	17.5(6)	17.1(7)	20.1(7)	-2.7(5)	1.5(5)	-1.5(5)
C01K	17.2(6)	16.0(7)	29.3(8)	-8.8(6)	-4.1(6)	3.2(5)
C01L	21.9(7)	15.0(7)	26.8(8)	-3.1(6)	4.3(6)	-1.2(5)
C01N	17.8(7)	18.7(7)	29.9(8)	1.6(6)	3.4(6)	-2.0(5)
C01O	19.9(7)	19.5(7)	27.4(8)	-2.6(6)	1.3(6)	-6.6(6)
C01Q	15.7(6)	12.8(7)	38.6(9)	-3.6(6)	-1.2(6)	-0.6(5)
C01R	17.7(7)	16.5(7)	45(1)	10.8(7)	1.0(6)	0.7(5)
C01S	22.7(7)	19.7(7)	31.8(8)	-0.5(6)	4.4(6)	5.7(6)
C01W	20.5(7)	13.3(7)	55.8(11)	-1.3(7)	8.1(7)	2.5(6)
C01X	21.9(7)	18.7(7)	38.5(9)	-6.0(6)	9.7(6)	-2.1(6)
C021	23.0(7)	16.9(7)	31.0(8)	-1.9(6)	3.2(6)	-1.6(6)

C023	31.8(8)	36.4(9)	17.2(7)	-4.0(6)	2.3(6)	-11.4(7)
C029	42.3(10)	43.2(11)	25.0(8)	3.0(8)	-0.8(7)	15.6(8)
C02A	23.0(8)	48.9(11)	24.5(8)	-0.1(7)	1.4(6)	14.5(7)
C02B	42.1(9)	30.3(9)	24.9(8)	-7.0(7)	12.6(7)	-8.5(7)
C02D	31.9(9)	40.5(10)	36.5(10)	-17.3(8)	1.3(7)	-6.3(8)
C02E	31.3(8)	44.3(10)	16.1(7)	-0.7(7)	-1.2(6)	12.3(7)
C02G	52.9(11)	42.3(11)	20.8(8)	5.5(7)	8.7(8)	1.5(9)
C02I	49.2(11)	31.7(9)	34.9(10)	-8.6(7)	17.3(8)	-20.1(8)
C02J	20.7(8)	73.3(15)	26.9(9)	3.5(9)	3.6(7)	-0.2(8)
C02K	33.1(9)	58.9(13)	44.2(11)	-24.5(10)	15.3(8)	-19.6(9)

Table S8. Bond Lengths for adduct 15b.

Atom	Atom	Length/Å	Atom	Atom	Length/Å
S00	C011	1.6665(14)	C00U	C00V	1.5177(18)
S1	C016	1.6682(14)	C00U	C00Y	1.5591(17)
S2	C015	1.6574(14)	C00V	C013	1.3995(19)
S3	C017	1.6606(14)	C00V	C01I	1.401(2)
O005	C010	1.3861(16)	C00W	C011	1.5340(18)
O005	C01B	1.4271(16)	C00W	C012	1.5573(17)
O006	C00Z	1.3687(17)	C00W	C015	1.5448(18)
O006	C01F	1.4371(17)	C00X	C012	1.5221(19)
O007	C019	1.3702(16)	C00X	C01A	1.399(2)
O007	C01B	1.4401(17)	C00X	C01D	1.4032(19)
O008	C00R	1.3909(17)	C00Y	C016	1.5330(18)
O008	C01F	1.4281(17)	C00Y	C017	1.5353(19)
O009	C00Z	1.2519(18)	C010	C014	1.4091(19)
O00A	C019	1.2503(17)	C012	C014	1.5169(18)
O00B	C00R	1.2360(18)	C013	C01K	1.391(2)
O00C	C010	1.2318(17)	C014	C019	1.3974(19)
O00D	N00M	1.2307(17)	C01A	C01X	1.392(2)
O00E	N00O	1.2269(18)	C01B	C01L	1.524(2)
O00F	N00M	1.2284(16)	C01B	C01O	1.5080(18)
O00G	N00O	1.2299(17)	C01D	C01R	1.389(2)
N00H	C00S	1.4287(13)	C01E	C01T	1.3900
N00H	C011	1.3368(18)	C01E	C01V	1.3900
N00I	C015	1.3349(19)	C01T	C01Z	1.3900
N00I	C01E	1.4180(14)	C01Z	C027	1.3900
N00J	C016	1.3307(18)	C027	C026	1.3900
N00J	C01H	1.4266(13)	C026	C01V	1.3900
N00K	C017	1.3344(19)	C01F	C01S	1.5086(19)
N00K	C01G	1.4245(14)	C01F	C021	1.519(2)
O00L	C02B	1.424(2)	C01G	C01J	1.3900
O00L	C02K	1.426(2)	C01G	C025	1.3900
N00M	C013	1.4750(19)	C01J	C020	1.3900
N00N	C023	1.498(2)	C020	C028	1.3900
N00N	C029	1.484(2)	C028	C02H	1.3900
N00N	C02I	1.490(2)	C02H	C025	1.3900

N00O	C01D	1.476(2)	C01H	C01P	1.3900
O00P	C02G	1.431(3)	C01H	C024	1.3900
O00P	C02J	1.425(2)	C01P	C022	1.3900
N00Q	C02A	1.4964(19)	C022	C02C	1.3900
N00Q	C02D	1.490(2)	C02C	C02F	1.3900
N00Q	C02E	1.489(2)	C02F	C024	1.3900
C00R	C00T	1.4106(19)	C01I	C01N	1.385(2)
C00S	C018	1.3900	C01K	C01Q	1.381(2)
C00S	C01M	1.3900	C01N	C01Q	1.386(2)
C018	C01C	1.3900	C01R	C01W	1.375(3)
C01C	C01U	1.3900	C01W	C01X	1.389(2)
C01U	C01Y	1.3900	C023	C02B	1.505(3)
C01Y	C01M	1.3900	C02A	C02J	1.498(3)
C00T	C00U	1.5189(18)	C02E	C02G	1.502(3)
C00T	C00Z	1.4016(19)	C02I	C02K	1.510(3)

Table S9. Bond Angles for adduct 15b.

Atom	Atom	Atom	Angle/°	Atom	Atom	Atom	Angle/°
C010	O005	C01B	116.77(11)	C019	C014	C012	116.97(12)
C00Z	O006	C01F	115.67(11)	N00I	C015	S2	127.60(11)
C019	O007	C01B	116.01(10)	N00I	C015	C00W	112.59(12)
C00R	O008	C01F	116.90(11)	C00W	C015	S2	119.81(10)
C011	N00H	C00S	126.94(11)	N00J	C016	S1	125.85(11)
C015	N00I	C01E	134.61(12)	N00J	C016	C00Y	112.46(12)
C016	N00J	C01H	127.89(12)	C00Y	C016	S1	121.65(10)
C017	N00K	C01G	130.02(12)	N00K	C017	S3	125.78(11)
C02B	O00L	C02K	109.23(13)	N00K	C017	C00Y	115.11(12)
O00D	N00M	C013	119.56(12)	C00Y	C017	S3	119.08(10)
O00F	N00M	O00D	123.09(13)	O007	C019	C014	119.67(12)
O00F	N00M	C013	117.30(12)	O00A	C019	O007	114.86(12)
C029	N00N	C023	111.90(13)	O00A	C019	C014	125.45(13)
C029	N00N	C02I	112.43(15)	C01X	C01A	C00X	121.74(15)
C02I	N00N	C023	109.89(13)	O005	C01B	O007	109.48(11)
O00E	N00O	O00G	123.54(14)	O005	C01B	C01L	110.90(11)
O00E	N00O	C01D	119.13(12)	O005	C01B	C01O	106.78(11)
O00G	N00O	C01D	117.23(13)	O007	C01B	C01L	110.02(12)
C02J	O00P	C02G	109.68(13)	O007	C01B	C01O	106.30(11)
C02D	N00Q	C02A	111.71(14)	C01O	C01B	C01L	113.18(12)
C02E	N00Q	C02A	109.77(13)	C00X	C01D	N00O	122.38(13)
C02E	N00Q	C02D	111.73(13)	C01R	C01D	N00O	114.51(13)
O008	C00R	C00T	116.74(12)	C01R	C01D	C00X	122.96(15)
O00B	C00R	O008	114.74(12)	C01T	C01E	N00I	125.79(8)
O00B	C00R	C00T	128.45(13)	C01T	C01E	C01V	120.0
C018	C00S	N00H	122.22(7)	C01V	C01E	N00I	114.19(8)
C018	C00S	C01M	120.0	C01Z	C01T	C01E	120.0
C01M	C00S	N00H	117.67(7)	C01T	C01Z	C027	120.0
C01C	C018	C00S	120.0	C026	C027	C01Z	120.0

C01U	C01C	C018	120.0
C01Y	C01U	C01C	120.0
C01U	C01Y	C01M	120.0
C01Y	C01M	C00S	120.0
C00R	C00T	C00U	125.28(12)
C00Z	C00T	C00R	118.83(13)
C00Z	C00T	C00U	115.65(12)
C00T	C00U	C00Y	112.00(11)
C00V	C00U	C00T	113.18(11)
C00V	C00U	C00Y	110.81(11)
C013	C00V	C00U	124.98(13)
C013	C00V	C01I	115.88(13)
C01I	C00V	C00U	119.11(12)
C011	C00W	C012	109.91(11)
C011	C00W	C015	108.64(11)
C015	C00W	C012	112.50(11)
C01A	C00X	C012	120.27(12)
C01A	C00X	C01D	115.88(13)
C01D	C00X	C012	123.79(13)
C016	C00Y	C00U	109.00(11)
C016	C00Y	C017	112.27(11)
C017	C00Y	C00U	112.28(11)
O006	C00Z	C00T	119.85(12)
O009	C00Z	O006	115.01(12)
O009	C00Z	C00T	125.12(13)
O005	C010	C014	117.26(12)
O00C	C010	O005	115.42(12)
O00C	C010	C014	127.26(13)
N00H	C011	S00	125.78(11)
N00H	C011	C00W	113.39(12)
C00W	C011	S00	120.82(10)
C00X	C012	C00W	111.51(11)
C014	C012	C00W	112.54(11)
C014	C012	C00X	112.82(11)
C00V	C013	N00M	122.47(13)
C01K	C013	N00M	114.43(13)
C01K	C013	C00V	123.10(14)
C010	C014	C012	123.88(12)
C019	C014	C010	119.03(12)
C027	C026	C01V	120.0
C026	C01V	C01E	120.0
O006	C01F	C01S	106.27(12)
O006	C01F	C021	110.38(12)
O008	C01F	O006	109.35(11)
O008	C01F	C01S	106.58(12)
O008	C01F	C021	111.06(12)
C01S	C01F	C021	113.00(13)
C01J	C01G	N00K	116.46(8)
C01J	C01G	C025	120.0
C025	C01G	N00K	123.53(8)
C020	C01J	C01G	120.0
C01J	C020	C028	120.0
C020	C028	C02H	120.0
C025	C02H	C028	120.0
C02H	C025	C01G	120.0
C01P	C01H	N00J	117.48(8)
C01P	C01H	C024	120.0
C024	C01H	N00J	122.40(8)
C01H	C01P	C022	120.0
C01P	C022	C02C	120.0
C02F	C02C	C022	120.0
C024	C02F	C02C	120.0
C02F	C024	C01H	120.0
C01N	C01I	C00V	121.88(14)
C01Q	C01K	C013	118.87(14)
C01I	C01N	C01Q	120.19(15)
C01K	C01Q	C01N	119.92(14)
C01W	C01R	C01D	119.34(15)
C01R	C01W	C01X	119.77(15)
C01W	C01X	C01A	120.19(16)
N00N	C023	C02B	109.94(13)
N00Q	C02A	C02J	109.98(16)
O00L	C02B	C023	111.10(15)
N00Q	C02E	C02G	109.57(13)
O00P	C02G	C02E	111.13(16)
N00N	C02I	C02K	110.33(15)
O00P	C02J	C02A	110.76(15)
O00L	C02K	C02I	110.95(16)

Table S10. Hydrogen Bonds for adduct 15b.

D	H	A	d(D-H)/Å	d(H-A)/Å	d(D-A)/Å	D-H-A/°
N00N	H00N	O009	0.95(2)	1.66(2)	2.6135(16)	176(2)
N00Q	H00Q	O00A	0.97(2)	1.69(2)	2.6476(17)	170(2)
N00J	H00J	O00C	0.88(2)	1.92(2)	2.7963(16)	174(2)

Table S11. Hydrogen Atom Coordinates (Å×10^4) and Isotropic Displacement Parameters (Å^2×10^3) for adduct 15b.

Atom	*x*	*y*	*z*	U(eq)
H018	6802.27	1891.1	3619.92	21
H01C	7523.98	964.88	3709.72	26
H01U	6892.55	198.85	4252.16	30
H01Y	5539.41	359.03	4704.81	34
H01M	4817.69	1285.25	4615.03	26
H00Y	3035.63	2942.51	5397.71	17
H012	4150.11	3444.03	2850.18	17
H01A	3477.4	3897.43	4351.02	24
H01T	7090.47	4598.24	3949.97	27
H01Z	8151.49	5203.01	3524.83	34
H027	8238.39	5171.4	2421.73	38
H026	7264.27	4535.04	1743.75	37
H01V	6203.24	3930.27	2168.88	28
H01J	1140	3562.68	7397.48	26
H020	53.07	4200.5	7746.46	33
H028	-961.24	4748.07	6994.07	40
H02H	-888.64	4657.81	5892.69	44
H025	198.27	4019.98	5543.7	36
H01P	3121.69	1234.2	4549.2	28
H022	2599.75	316.48	4126.43	35
H02C	1127.21	-31.94	4266.86	43
H02F	176.6	537.36	4830.07	47
H024	698.53	1455.08	5252.85	34
H01I	4376.14	3531.85	5585	22
H01K	4505.72	4640.69	7471.98	26
H01B	3563.48	1786.45	2979.8	32
H01D	2981.62	1372.27	2459.21	32
H01E	3035.42	1230.96	3201.65	32
H01N	5014.16	4473.11	5640.44	27
H01F	1233.04	1275.06	3101.08	34
H01G	1183.37	1389.69	2353.57	34
H01H	711.61	1839.07	2780.36	34
H01Q	5057.86	5038.82	6574.69	28
H01R	2786.81	5294.97	2711.72	32
H01L	6499.81	1160.99	6645.49	37
H01O	5988.18	723.99	6118.31	37
H01S	5880.82	641.46	6850.98	37
H01W	2609.32	5534.97	3765.52	36
H01X	2953.91	4832.3	4584.28	31
H02A	4116.78	705.09	6578.55	36
H02B	4165.44	794.5	5837.6	36
H02D	3657.11	1268.06	6213.43	36
H02E	5706.73	2435.41	8991.21	34
H02G	5418.93	2000.56	9517.75	34

H02I	6232.37	1557.19	8383.31	56
H02J	5530.99	1026.81	8206.78	56
H02K	5961.75	1146.7	8934.44	56
H02R	3741.4	2110.06	619.15	39
H02S	3786.25	2061.01	1376.35	39
H02L	4538.15	2874.43	9498.43	38
H02M	4165.7	2784.35	8754.95	38
H02T	2040.79	1848.48	454.47	55
H02U	1350.08	2219.31	809.51	55
H02V	2084.58	1791.72	1209.27	55
H02W	1876.37	3172.61	397.08	37
H02X	2528.85	2811.4	-1.13	37
H02Y	3118.06	3773.45	181.24	46
H	3171.37	3688.48	934.64	46
H02N	4253.65	1159.53	9117.33	45
H02O	3882.19	1124.56	8367.52	45
H02Z	4275.87	3046.35	1502.04	48
HA	4974.3	2691.46	1136.03	48
H02P	3118.13	2036.42	8404.36	53
H02Q	2767.58	1597.75	8902.61	53
H00N	4755(16)	1938(11)	8193(11)	43(6)
H00Q	2640(15)	2779(10)	1338(11)	40(6)
H00I	5592(14)	3602(9)	2987(10)	27(5)
H00J	2557(14)	2221(10)	4877(10)	30(5)
H00H	5096(14)	2339(9)	4450(10)	29(5)
H00K	1615(14)	3037(10)	6522(10)	31(5)
H00W	4565(11)	3201(7)	4195(8)	12(4)
H00U	3226(11)	2827(7)	6763(8)	10(4)

Figure S74. HRMS data for compound 15a' (Table 1, entry 1)

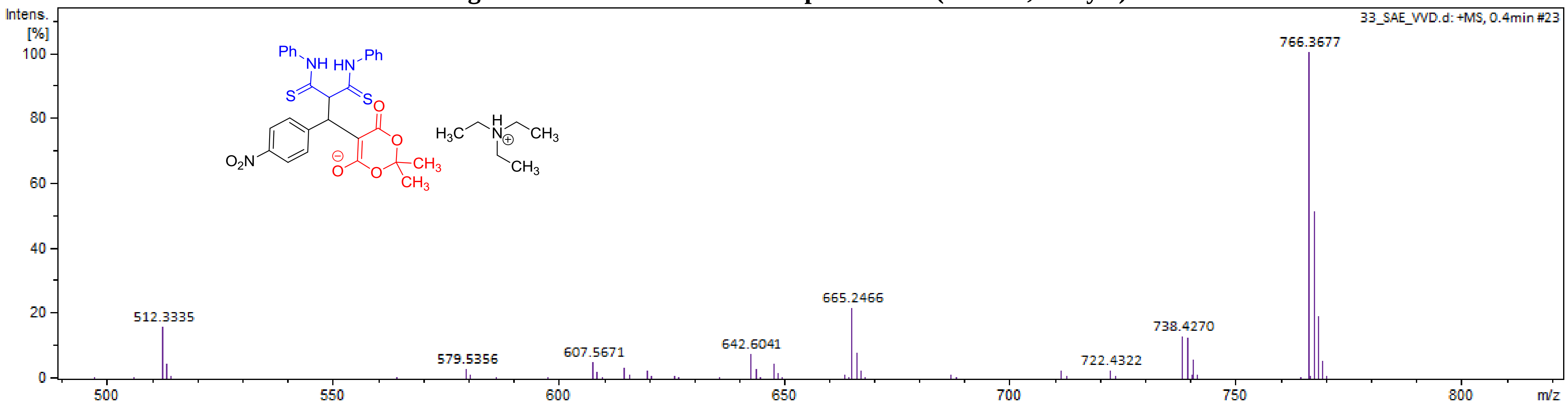

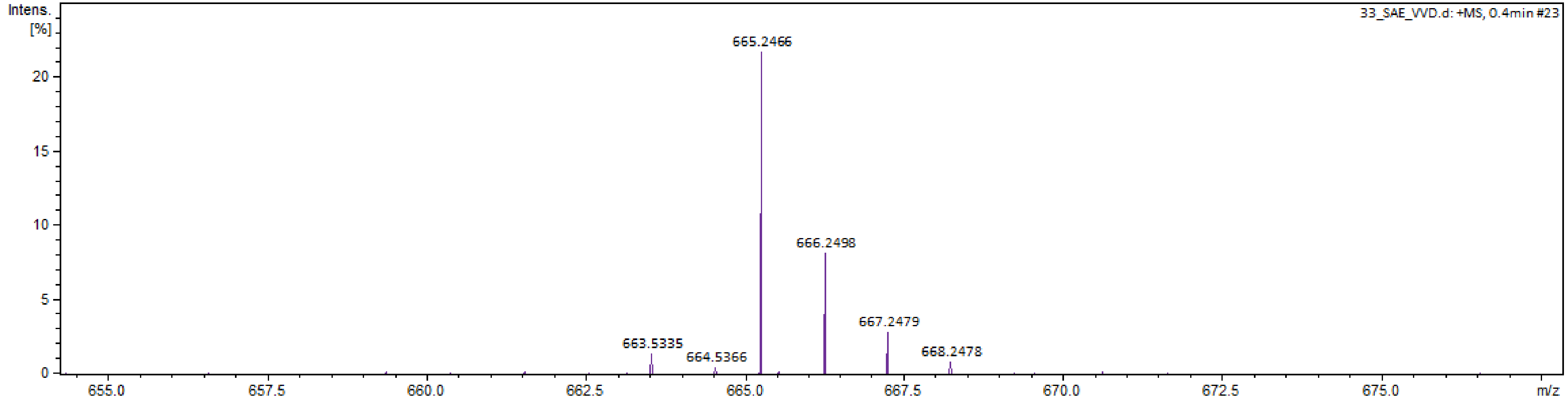

Figure S75. HRMS data for a mixture of Michael adduct 15a and dithiolopyridine 17a (Table 1, entry 5)

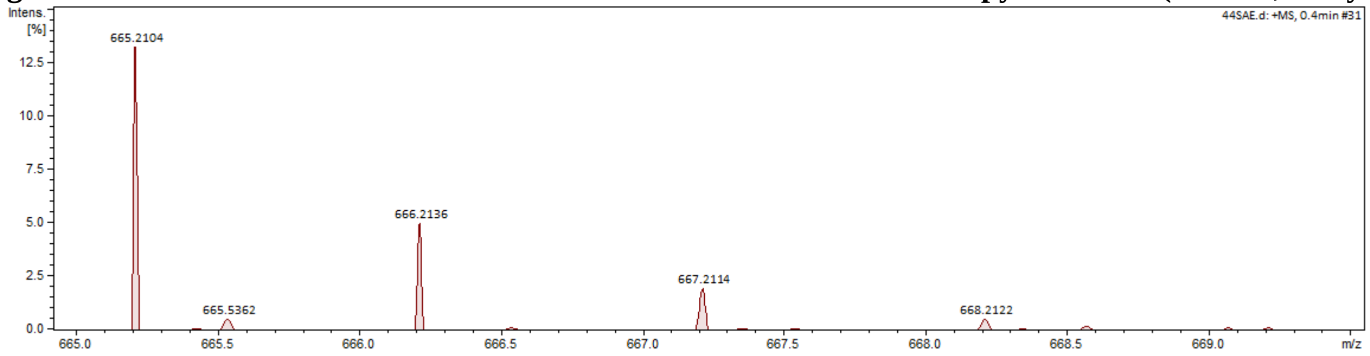

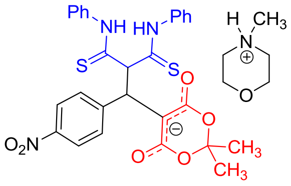

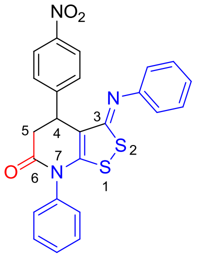

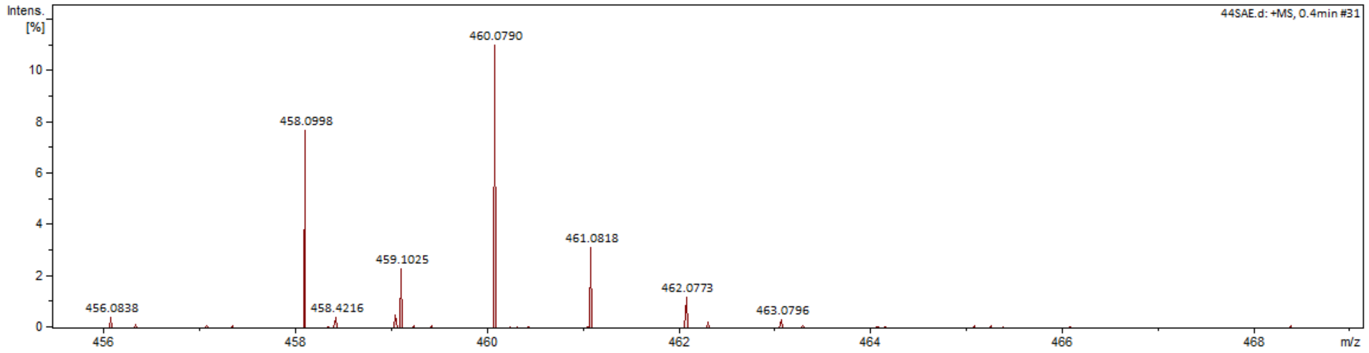

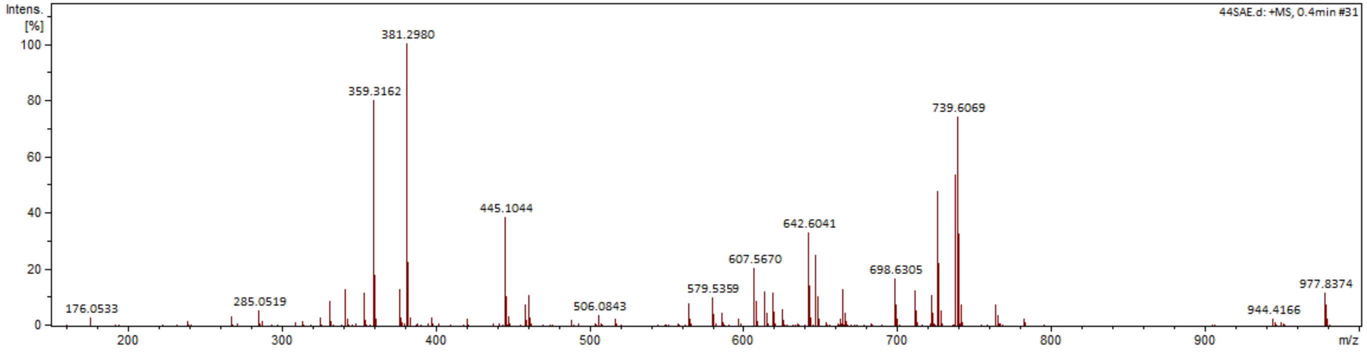

Figure S76. HRMS data for compound 15b (Table 1, entry 6)

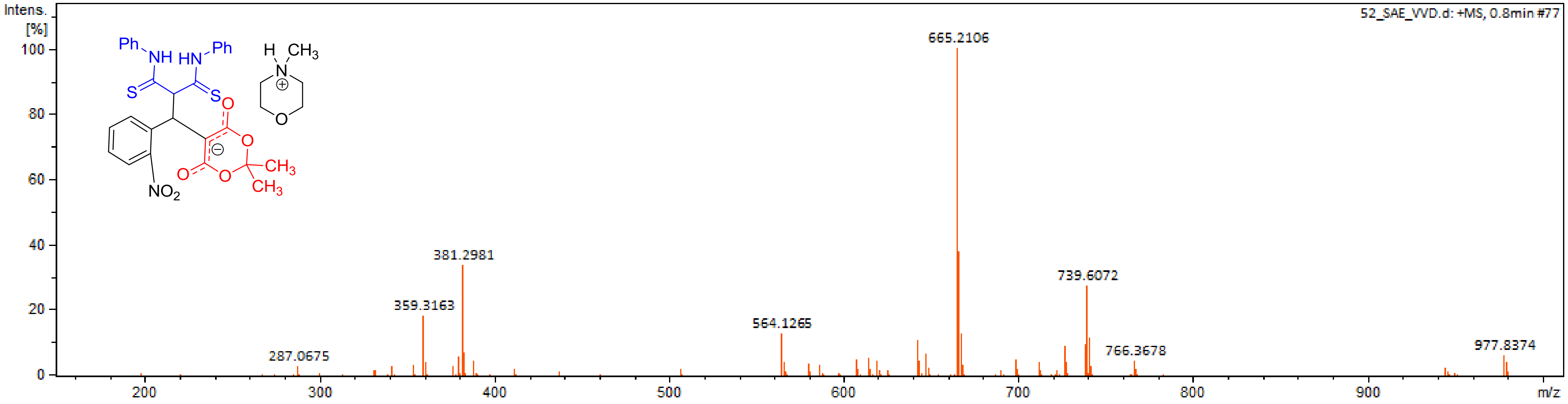

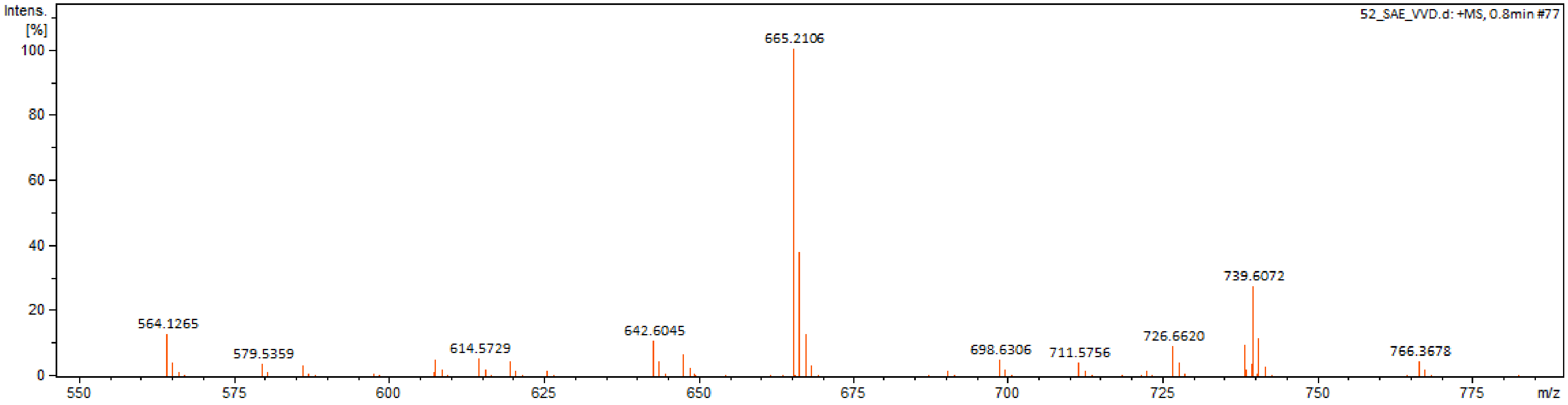

Figure S77. HRMS data for Michael adduct 15d and cyclization product 16d (Table 1, entry 11)

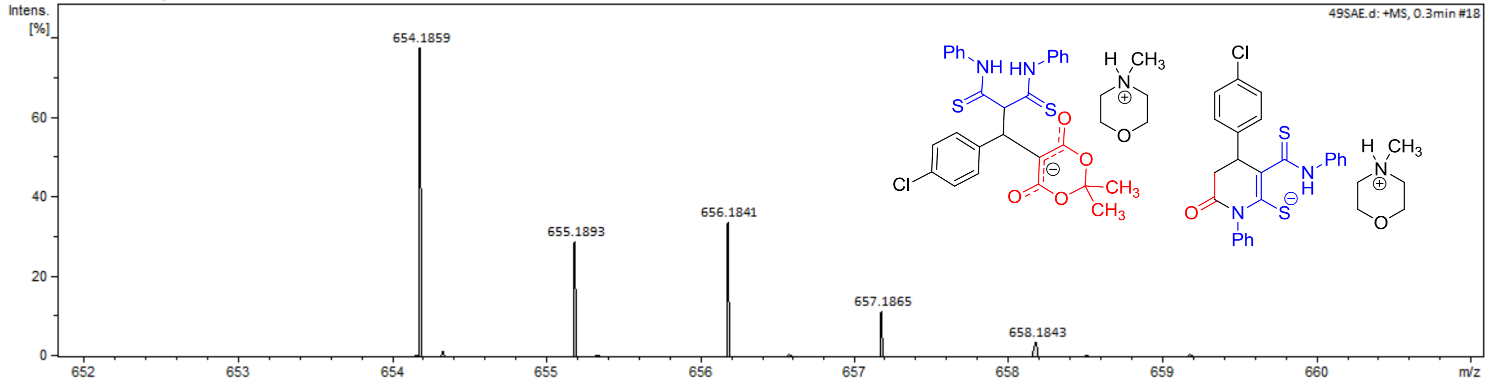

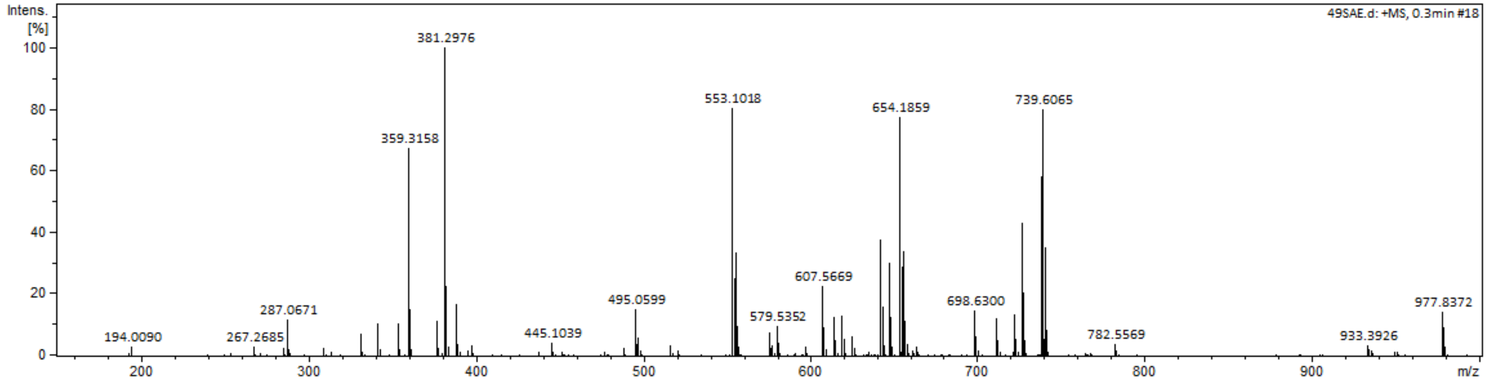

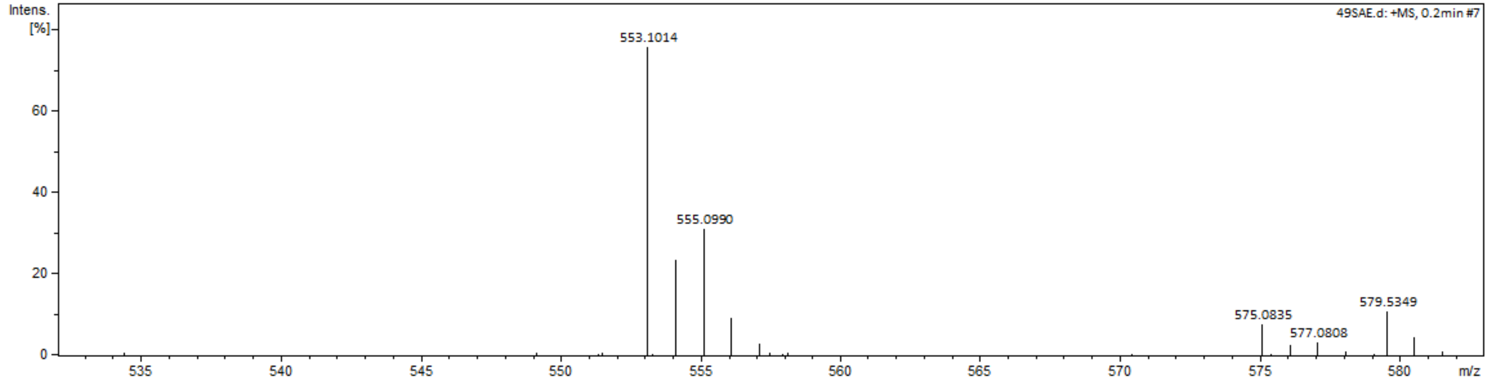

Figure S78. HRMS data for a mixture of Michael adduct 15e and cyclization product 16e

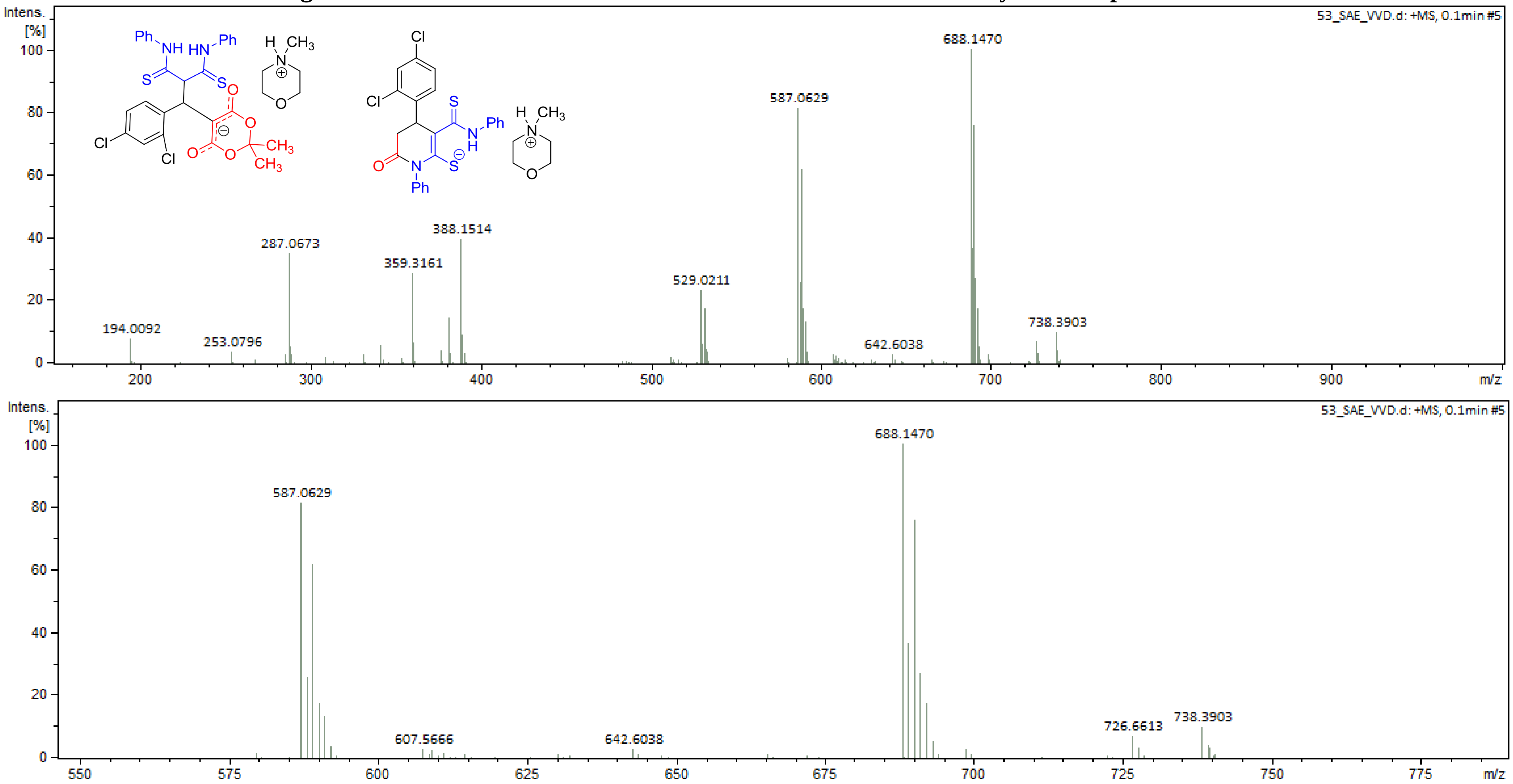

Figure S79. HRMS data for a mixture of Michael adduct 15f and by-products 16f,17f (Table 1, entry 14)

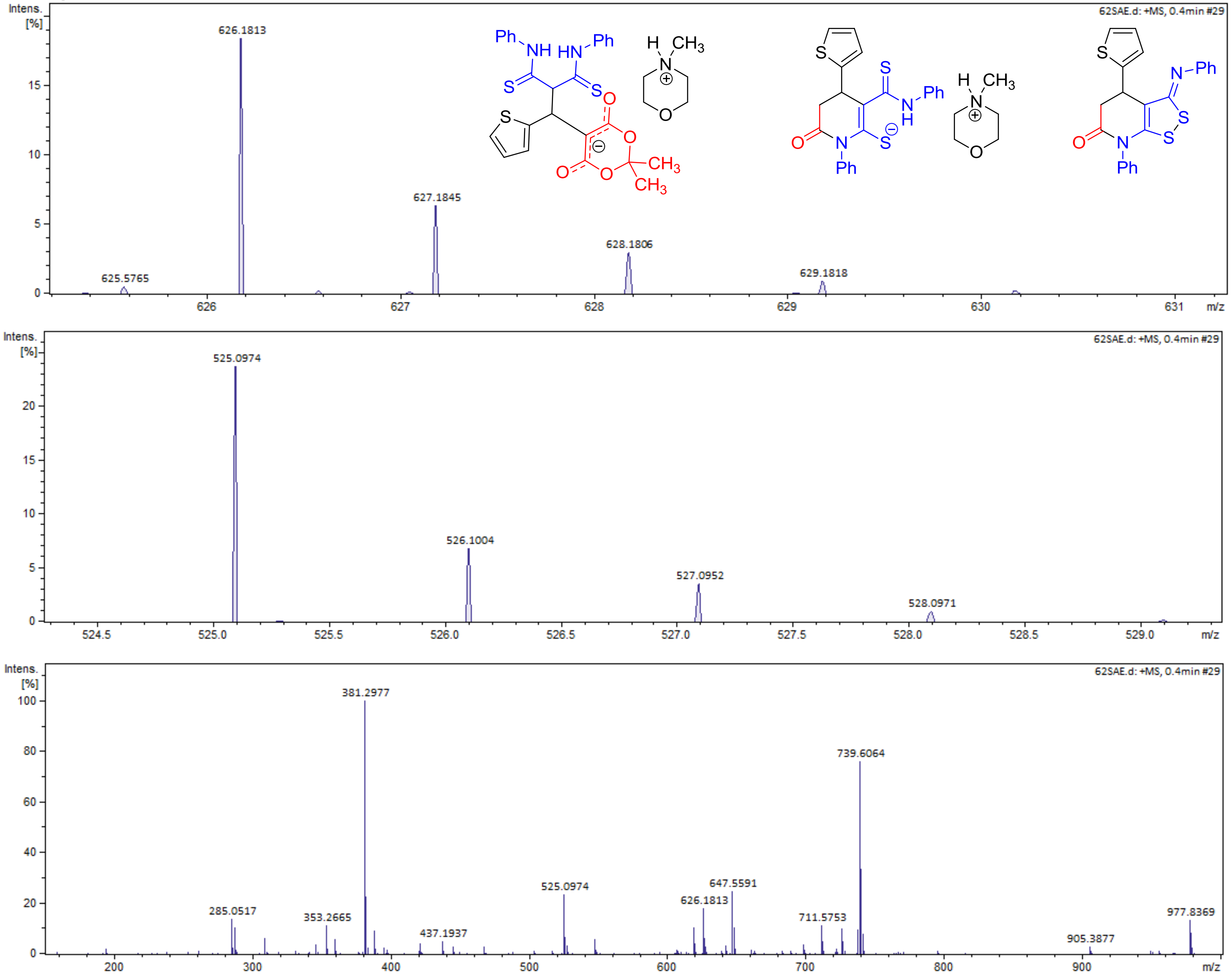

Figure S80. HRMS data for a mixture of Michael adduct 15h and by-products 16h,17h (Table 1, entry 17)

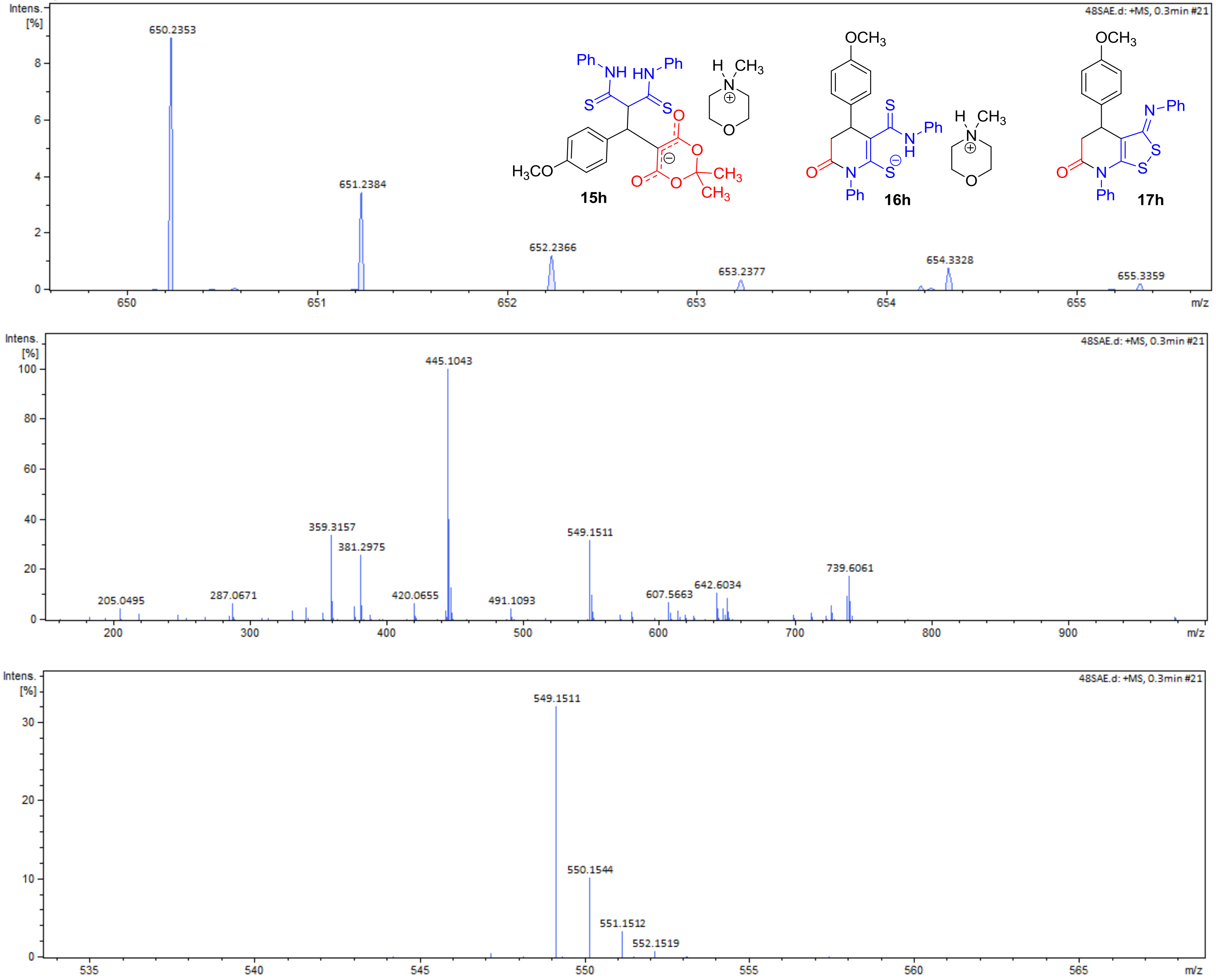

Figure S81. HRMS data for a mixture of Michael adduct 15i and by-products 16i and strating dithiomalondianilide (Table 1, entry 18)

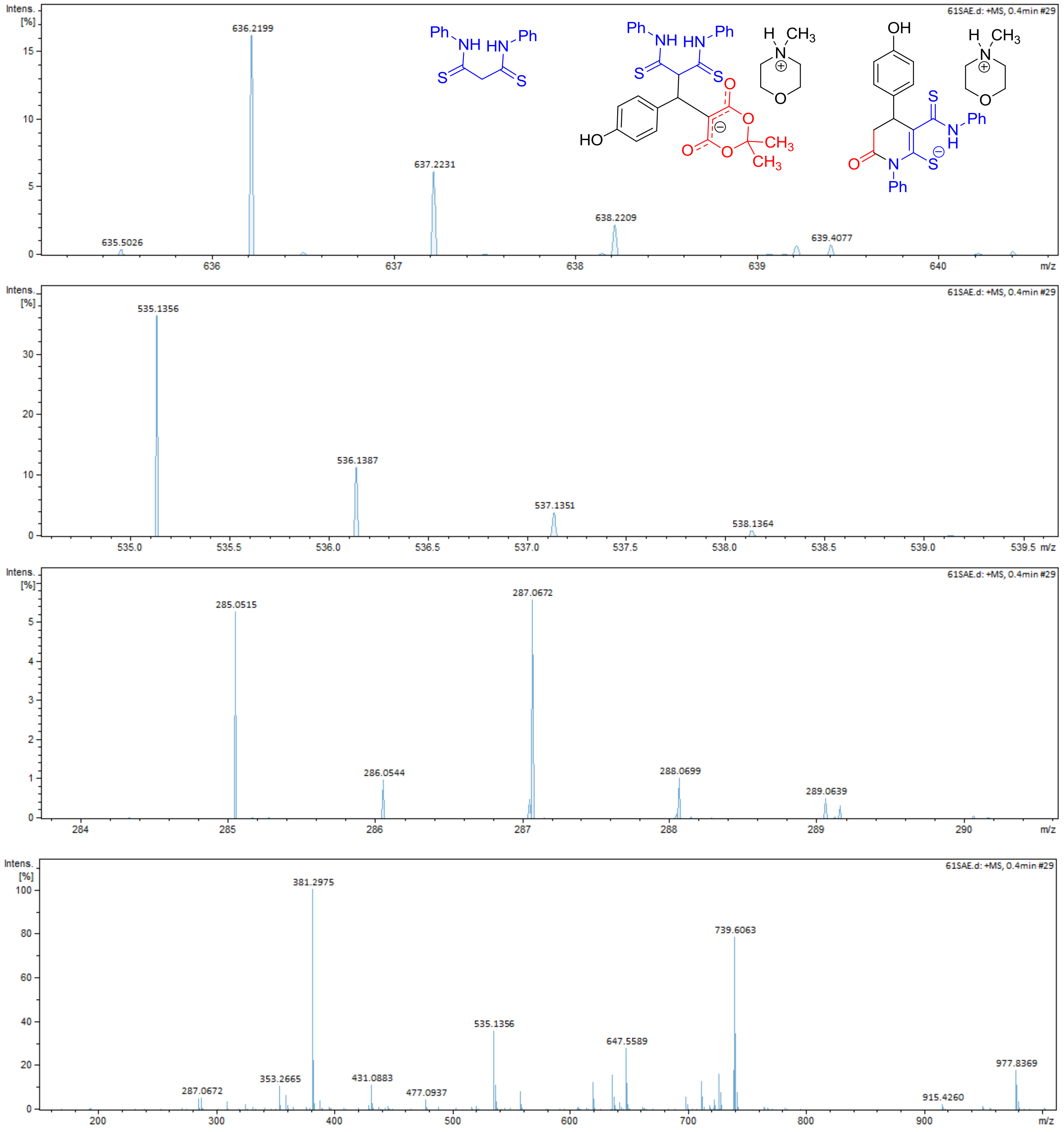

Supplement: Supplementary file 1 [file ijms-23-15997-s001.zip › ijms-2055720-supplementary.pdf]
